# Co-opting the bacterial lipoprotein pathway in the biosynthesis of a lipidated macrocyclic peptide

**DOI:** 10.1101/2025.10.31.685832

**Published:** 2025-11-01

**Authors:** Jeff Y. Chen, Lingyang Zhu, Kevin Y. Zhang, Deborah A. Berthold, Wilfred A. van der Donk

**Affiliations:** 1 Department of Chemistry and Howard Hughes Medical Institute, 600 South Mathews Avenue, University of Illinois at Urbana–Champaign, Urbana, Illinois 61801, USA.; 2 Carl R. Woese Institute for Genomic Biology, University of Illinois at Urbana-Champaign, 1206 West Gregory Drive, Urbana, Illinois, 61801, USA.; 3 School of Chemical Sciences NMR Laboratory, University of Illinois at Urbana-Champaign, Urbana, 61801, IL, USA.

**Keywords:** Lipopeptide, acylation, metalloenzyme, biocatalysis, Bacteroidetes, secondary metabolites, RiPPs, DUF692, UPF0276, Biochemistry, Biosynthesis, Natural Products, Peptides, Lipids

## Abstract

A family of bacterial multinuclear non-heme iron dependent oxidative enzymes (MNIOs) are involved in diverse transformations during the biosynthesis of ribosomally synthesized and post-translationally modified peptides (RiPPs). ChrH from the genus *Chryseobacterium* catalyzes a remarkable backbone rearrangement that involves macrocyclization, heterocyclization, and *S*-methylation. ChrH is part of a larger subfamily that includes members from other bacterial phyla. By leveraging comparative genomics, we characterize other products produced by this enzyme subfamily, which includes unmethylated macrocyclic congeners as well as C-terminally modified proteins of >30 kDa. We show that this MNIO subfamily recognizes substrates by their conserved C-terminal motif, allowing for structural diversification at their N-termini. For instance, the N-termini of some substrates contain a signal peptide for downstream maturation by the ubiquitous bacterial lipoprotein biosynthetic pathway. We demonstrate that, like bacterial lipoproteins, such peptides are modified by addition of diacylglycerol (DAG) groups to the N-terminal Cys residue along with acylation of the N-terminal amino group. Genome mining reveals examples of additional predicted RiPP-lipoprotein hybrids, which we term DAG-RiPPs. These results lay the foundation for the discovery of other RiPP-lipoproteins hybrids.

## Introduction

Ribosomally synthesized and post-translationally modified peptides (RiPPs) are a class of structurally diverse natural products possessing a wide range of biological activites^1–5^. A rapidly growing number of modifying enzymes have been shown to install various post-translational modifications onto their respective precursor peptides^6,7^. One such class of enzymes is the multinuclear non-heme iron-dependent oxidative enzyme family (MNIO, formerly DUF692/UPF0276), whose members catalyze diverse and challenging peptide modifications^8–10^. MNIOs can act on different amino acid residues, such as cysteine, asparagine, aspartate, and phenylalanine. In the biosynthesis of the copper-binding peptide methanobactin, MbnB (with its partner protein MbnC) converts a cysteine residue to an oxazolone-thioamide^11^, and HvfBC installs the same modification on the conserved EGKCG motif of copper-binding oxazolins^12^ (Supplementary Fig. 1). In alternative strategies, BufBC converts a cysteine residue to a 5-thiooxazole in the biosynthesis of the widespread bufferin peptides^13^, and TglH (with its partner protein TglI), excises the β-carbon from a C-terminal cysteine to form norcysteine^14^. ChrH performs another remarkable reaction in which a conserved motif featuring two cysteines is converted to a product that contains a macrocycle and a hydantoin heterocycle, and that is *S*-methylated^15^ (Supplementary Fig. 1). In a diversion of reactions involving Cys residues, three other MNIOs, ApyH, PflD and MovX, act on aspartate and asparagine residues^16–18^. Recently, PbsC was shown to perform *ortho*-hydroxylation of phenylalanine in the biosynthesis of the biphenomycin class of antibiotics^19^. While MNIOs catalyze widely diverse reactions, they share a common TIM-barrel fold that houses two to three irons in its active site^20–22^, and use molecular oxygen to perform a four-electron oxidation on their substrate in most cases^8^.

The biosynthetic gene cluster (BGC) encoding ChrH is enriched in the genus *Chryseobacterium*, but this subfamily is widespread across Bacteroidota and Cyanobacteria^8^. We hypothesized that comparative genomics could facilitate the discovery of new products from this subfamily. Here, we identified ChrH homologs, showing a distribution across various gram-negative phyla including Bacteroidota, Cyanobacteria, and Myxococcota. Multiple sequence alignments show that the precursor peptides in the majority of other Bacteroidota genera contain an additional highly conserved cysteine residue. A representative member from this group was selected for analysis, DybA from *Dyadobacter crusticola*, which is modified by the MNIO DybH. Heterologous coexpression in *E. coli* showed production of a macrocyclic congener of the product from ChrA that contains a free, unmethylated thiol. Characterization of other homologs demonstrate a surprising substrate tolerance of the ChrH/DybH-subfamily, with homologs from Cyanobacteria modifying precursor proteins over 34 kDa in size. Additionally, bioinformatic analyses and biochemical experiments show that several of these RiPPs contain an N-terminal signal sequence, resulting in lipidation of the modified peptide via the ubiquitous bacterial lipoprotein biosynthesis pathway. We demonstrate that the RiPP product of the lipoprotein pathway contains the additional conserved Cys as the N-terminal residue, and that this Cys carries a diacylglycerol (DAG) group on its sulfur and an acyl group on its amino group. Bioinformatic analyses show that these RiPP-lipoprotein hybrids are widespread across other subfamilies of MNIOs. We suggest to call these products DAG-RiPPs to distinguish them from the growing group of lipidated RiPPs that are *N-*acylated^23^.

## Results

### Phylogenetic analysis of ChrH-like gene clusters

**Fig. 1.**
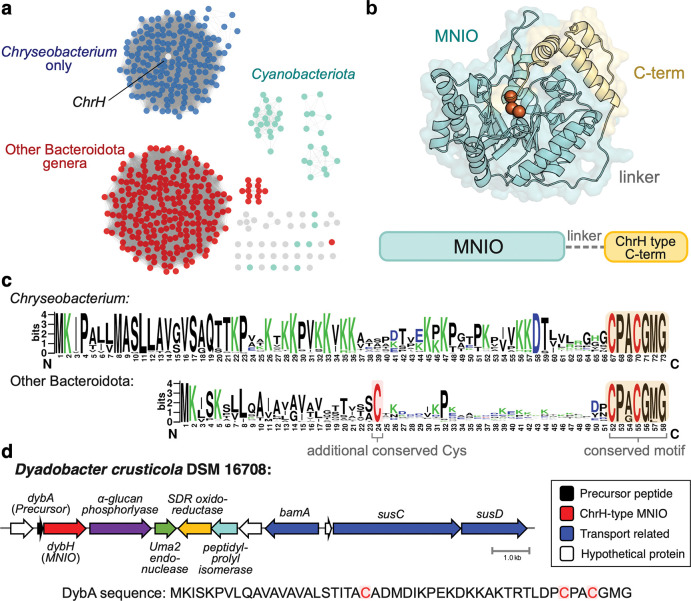
Bioinformatic analysis of the ChrH-like subfamily of MNIOs. **a** SSN of protein sequences in the ChrH subfamily, showing division into two clusters, one enriched specifically in *Chryseobacterium*, and one containing several Bacteroidetes genera. Smaller clades contain members from Cyanobacteria and other phyla. The SSN was generated using an alignment score of 110 and e-value of 10^−5^. The full list of proteins can be found in Supplementary Table 2, and the Cytoscape file as Dataset 1. **b** Structural model of ChrH predicted by Alphafold 3 showing the overall fold of ChrH-family enzymes. Three iron atoms are modelled and represented as orange spheres. ChrH family enzymes contain the core MNIO domain in addition to an approximately 7.8 kDa C-terminal helical bundle joined by a linker. In Cyanobacteria, the C-terminal domain is encoded as a separate protein. **c** Sequence logo of ChrA-like peptides from *Chryseobacterium* and other Bacteroidota genera, showing shared conservation of the CPACGMG motif. However, ChrA-like peptides from *Chryseobacterium* are longer and lack an additional conserved cysteine. **d** Biosynthetic gene cluster from a representative species from the Bacteroidetes-type precursors, *Dyadobacter crusticola* DSM 16708. The precursor peptide DybA is located upstream of the MNIO DybH. Below: the amino acid sequence of DybA with conserved cysteines shown in red.

To analyze the ChrH-like subfamily in detail, we first generated a sequence similarity network (SSN)^24^ of ChrH homologs using an alignment score of 110 (Fig. 1a). We observed a clear division of the subfamily into two main clusters. One cluster is populated by members of the *Chryseobacterium* genus, which includes the previously characterized ChrH^15^. The other cluster contains members of other Bacteroidota genera (mostly *Dyadobacter, Chitinophaga,* and *Hymenobacter*). Finally, smaller clusters are populated by other gram-negative bacterial phyla, including Cyanobacteriota, Myxococcota, and Planctomycetota.

Structural prediction of ChrH by Alphafold 3^25^ shows that in addition to the core TIM barrel that is present in all MNIOs, ChrH contains an additional C-terminal helical bundle (Fig. 1b). This additional C-terminal domain is present across all members of the ChrH family (in Fig. 1a), but is absent in other characterized MNIOs such as MbnB and TglH^21,22^. In the Cyanobacteriota MNIOs, this C-terminal domain is structurally conserved, but encoded as a separate polypeptide, suggesting fusion of the two domains at some point during evolution (Supplementary Fig. 2).

Analysis of the precursor peptides associated with these MNIOs showed that they contain the highly conserved CPxCGxG motif at the end of the peptide across the entire subfamily, with CPACGMG being the most frequently observed sequence (Fig. 1c). However, comparison of ChrA-like precursors from *Chryseobacterium* with precursors from other Bacteroidota clades show key differences. Precursor peptides from other Bacteroidota genera are shorter and contain an additional, completely conserved cysteine residue in the middle of the peptide. Given the proclivity of MNIOs to act on Cys residues^8^, we initially hypothesized that the additional cysteine could be modified or affect the reactivity of the MNIO.

Co-occurrence analyses also support differences between the BGCs of *Chryseobacterium* versus other Bacteroidota genera (Supplementary Table 1). Such analysis shows that whereas the ChrH-type enzymes are part of a more typical operon with highly conserved genes, the Bacteroidota enzyme families appear to be encoded in BGCs that minimally consist of the precursor peptide and MNIO, with other, mostly transport related genes at lower conservation. Notably, the BGCs do not encode an obvious conserved partner protein used for substrate binding that is encoded nearby for most characterized MNIOs^8^.

To characterize the modification performed by this divergent cluster in the ChrH subfamily, we selected a representative member, DybH, from *Dyadobacter crusticola* DSM 16708, isolated from soil crusts in Colorado^26^ (Fig. 1d). DybH has an amino acid sequence identity of 40% compared to ChrH, and the associated precursor peptide DybA contains the third conserved cysteine in addition to the CPxCGxG motif. The BGC also lacks a gene for an obvious partner protein.

### Coexpression of DybA and DybH yields a −4 Da modification

**Fig. 2.**
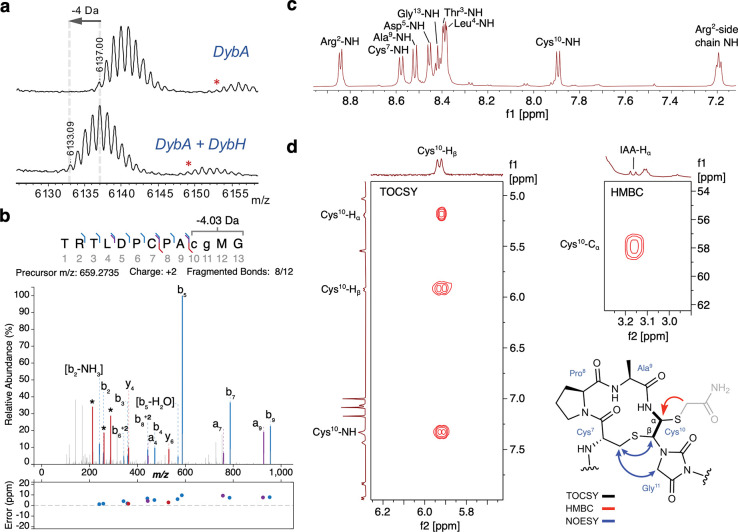
DybH installs a macrocycle and heterocycle onto DybA. **a** MALDI-TOF MS of DybA expressed alone or coexpressed with DybH in *E. coli*. M+H peaks are labelled. Expected monoisotopic [M+H]^+^ mass of unmodified peptide: 6137.12 Da (Supplementary Table 3). Red asterisks denote peptide with oxidized methionine (+16 Da). Experiments were repeated in triplicate with identical results (*n* = 3 biological replicates). **b** Annotated LC-MS/MS spectrum of LysC-digested, DybH-modified peptide. Asterisks denote modified y-ion fragmentations (see Supplementary Fig. 3). **c** ^1^H NMR spectrum showing eight amide N-H protons (and an N-H from an Arg side chain), indicating two missing signals. **d** Left: diagnostic TOCSY signals for the DybAH product showing a new modified spin system with three protons. Right: key HMBC correlation between the methylene proton of the carbamidomethylene group (in grey) and the α-carbon of the thio-hemiaminal. Proposed structure of the DybAH product (IAA-alkylated) based on diagnostic TOCSY, HMBC, and NOESY signals. Residues numbered (based on the fragment obtained after LysC cleavage) are shown in blue. Full spectra and assignments in Supplementary Figs 6-14 and Supplementary Tables 4-5.

We first heterologously co-expressed the genes encoding the His-tagged precursor peptide DybA with DybH in *E. coli*. Purification of the peptide (which we will refer to as DybAH heretoforth) and analysis by matrix-assisted laser desorption/ionization time-of-flight mass spectrometry (MALDI-TOF MS) showed a −4 Da mass shift relative to the unmodified precursor peptide (Fig. 2a). As forecast by the architecture of the BGC, DybH was active without the need for a partner protein. The −4 Da shift catalyzed by DybH is different from that observed for ChrH, which results in an overall +10 Da mass shift^15^.

Digestion of the full-length peptide to a 13-mer peptide by LysC protease, followed by LC-MS/MS, localized the −4.0313 Da modification to the C-terminal CPACGMG motif (Fig. 2b). This mass change is consistent with a loss of 4 hydrogens. Several observed fragments could be assigned to modified *y*-ions resulting from the final four amino acids (Supplementary Fig. 3). These fragments suggest that whereas the product is distinct from the product formed by ChrH (Figure S1), similarities in structure do exist in the DybH product. Reaction with the thiol-specific alkylating reagent iodoacetamide (IAA) showed one adduct to the 13-mer peptide, suggesting the presence of only one free thiol, with the second thiol that was present in the substrate now modified such that it is not reactive towards electrophiles (Supplementary Fig. 4).

UV-vis characterization of the modified peptide did not reveal an absorbance feature, suggesting the lack of formation of aromatic functional groups observed in other MNIO-modified peptides such as methanobactins or bufferins^11,13^ (Supplementary Fig. 5). Together, the presence of a free thiol, a −4 Da modification, and the observed fragments by tandem MS suggest that the product could be the non-methylated congener of modified ChrA.

### DybAH analysis by NMR spectroscopy shows macrocycle and heterocycle formation

We used NMR spectroscopy to determine the structure of the modified, LysC-digested DybA peptide co-expressed with DybH. The ^1^H NMR spectrum in 90% H_2_O:10% D_2_O showed the disappearance of the two amide N-H signals for methionine and glycine in the conserved CPACGMG motif (Fig. 2c, Supplementary Fig. 6). Furthermore, TOCSY and HSQC data showed a coupled spin system with three protons associated with the former Cys10 (numbering based on the peptide obtained by LysC digestion, Fig. 2d, Supplementary Fig. 7 and 8). The former Cys10 β-proton at 5.81 ppm is shifted far downfield from a typical Cys β-proton. We observed an NOE from this β-proton of former Cys10 to the β-protons of Cys7 (Supplementary Fig. 9). An NOE cross peak was also observed between the β-protons of Cys7 and the α-protons of Gly11. Collectively, these observations are similar to previous observations for the ChrA product and suggest formation of two thiohemiaminals^15^ (Fig. 2d). In H_2_O/ D_2_O, the observed peaks for both thiohemiaminal protons (Cys^10^ Hα and H_β_) were broad, which was also observed for modified ChrA. Therefore, we alkylated the peptide with IAA and recorded a spectrum in DMSO-d6. The broad peak corresponding to the former Cys^10^ Hα sharpened to a triplet, as expected by being split by the former Cys^10^ NH and H_β_ (Supplementary Fig. 10-13). These spectra also confirmed that the β-proton of former Cys10 is a CH group in the product instead of a CH_2_ group in the substrate (Supplementary Fig. 11). Analysis of the IAA-alkylated peptide also revealed the position of the free thiol in the enzymatic product because we observed an HMBC cross peak between the α-protons of the carbamidomethylene originating from IAA (3.16 ppm) and the α-carbon (57.3 ppm) of the former Cys10 (Fig. 2d, and Supplementary Fig. 14). These data together support the structure of modified DybA as having a macrocycle and hydantoin heterocycle (Fig. 2d). While the stereochemistry of the thiohemiaminals is predicted to be *trans* based on the J-coupling of 11.8 Hz, additional crystallographic studies of the modified peptide are needed to establish the absolute stereochemistry.

### DybH characterization and in vitro reconstitution

We purified His-tagged DybH aerobically. DybH was yellow in color, unlike ChrH, which is purple when purified aerobically^15^. DybH routinely co-purified with approximately 1.8–2.2 irons per protomer under the expression conditions used in this study. The UV-Vis spectrum showed a shoulder at 350 nm, which is also present in other MNIOs and non-heme iron-containing enzymes^12^ (Supplementary Fig. 15).

We tested whether the aerobically prepared enzyme was active, and observed the same modification of DybA in vitro as in *E. coli*. Incubation of DybA with DybH resulted in production of the −4 Da product and DybH performed multiple turnovers of substrate (Supplementary Fig. 16). These results confirm that a partner protein is not needed (as also observed with MovX^17^), and that DybH alone can perform the four-electron oxidation of its substrate. These data also suggest a mechanism consistent with that proposed for ChrH^15^. The isolation of a congener lacking *S-*methylation supports a stepwise mechanism in which heterocyclization and macrocyclization are not concerted with the final methylation step (Supplementary Fig. 17). Detailed mechanistic studies will be needed to understand the initial steps involving macrocycle and hydantoin formation, as well as the molecular basis for the differences in methylation.

### Modifications performed by homologs in Bacteroidota and Cyanobacteria

**Fig. 3.**
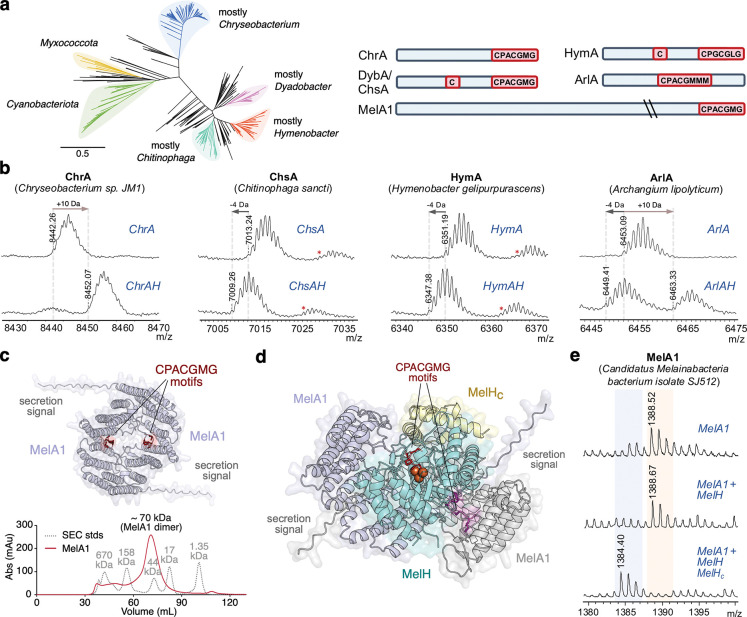
Characterization of ChrH homologs in Bacteroidota, Myxococcota, and Cyanobacteriota. **a** Left: Unrooted maximum-likelihood phylogenetic tree of the ChrH subfamily highlighting the major expanded clades. Scale bar for branch length indicates number of amino acid substitutions per site. Right: Diagrams of the representative diverse substrates (not to scale), showing conserved cysteines and the conserved CPxCGxG motif in different contexts. **b** MALDI-TOF MS of peptides coexpressed with their respective MNIO from representative members from Bacteroidota clades (*Chryseobacterium, Chitinophaga, Hymenobacter*) and a member from Myxococcota (*Archangium*). Expected monoisotopic [M+H]^+^ mass of peptides: ChrA, 8442.54 Da unmodified, 8452.52 Da modified; ChsA, 7013.45 Da unmodified, 7009.42 Da modified; HymA, 6351.22 Da unmodified, 6347.18 Da modified; ArlA, 6453.07 Da unmodified, 6449.04 Da modified. Experiments were repeated in triplicate with identical results (*n* = 3 different biological replicates). **c** Alphafold 3 model of a homodimer of MelA1 from a representative Cyanobacteria species (Candidatus *Melainabacteria bacterium* isolate SJ512), showing the C-terminal CPACGMG motif in red. Below: size-exclusion chromatography (SEC) analysis of MelA1 indicates dimer formation. **d** Alphafold 3 model of (MelA1)_2_ in complex with MelH and MelH_c_. The CPACGMG motif (shown as red sticks) from one copy of MelA1 is positioned next to the triiron active site (shown as orange spheres). The second copy of MelA1 is shown in grey. **e** MALDI-TOF MS of the C-terminal fragment of MelA1 containing the CPACGMG motif (obtained by LysC-digestion) from experiments in which MelH was expressed alone, with MelH, or together with MelH and MelH_c_. Expected monoisotopic [M+H]^+^ mass of C-terminal fragment: 1388.62 Da unmodified, 1384.58 Da modified.

The different reactivity at the same CPACGMG motif observed in DybA versus ChrA prompted us to explore homologs in other clades with different gene architectures to investigate whether they produce the *S-*methylated or unmethylated product. An unrooted maximum-likelihood phylogenetic tree of the ChrH subfamily was generated to gain more resolution into the different clades (Fig. 3a). The tree shows separation of distinct clades, predominantly expanded in the Bacteroidota genera *Chryseobacterium, Chitinophaga, Dyadobacter,* and *Hymenobacter*, as well as the phyla Cyanobacteriota/Myxococcota. The large diversity of gene architectures not only include ChrA-type and DybA-type precursor peptides, but also precursors that contain variations to the CPACGMG motif. For example, some precursors from Myxobacteria have a CPACGMMM motif that is not at the C-terminus, but instead embedded within the peptide (Fig. 3a). Furthermore, we observed that several Cyanobacteria BGCs encode putative precursor proteins over 30 kDa in size, which end with the CPACGMG motif at the C-terminus. These unusually large RiPP precursors are tetracopeptide repeat containing proteins^27^, but otherwise do not contain any other obvious predicted domains by sequence or structure.

Coexpression of ChrA with ChrH in *E. coli* resulted in formation of the +10 Da product even in the absence of ChrI (Fig. 3b), which was previously reported as required^15^. Coexpressions of ChsA with ChsH from *Chitinophaga sancti*, and HymA with HymH from *Hymenobacter gelipurpurascens* all showed the production of the −4 Da (unmethylated) product (Fig. 3b; for BGCs, see Supplementary Fig. 18). The C-terminal sequence of HymA ends with CPGCGLG, resulting in formation of a different macrocyclic congener. Coexpression of ArlAH, which contains an embedded CPACGMMM motif, showed a mixture of both the unmethylated and *S-*methylated product, further supporting that these two products are formed via the same route (Fig. 3b).

We next investigated the homologous gene clusters from Cyanobacteria that contain precursor proteins 30 to 40 kDa in size. One example is the *mel* cluster from *Melainabacterium bacterium* isolate SJ512 (Supplementary Fig. 18). The 34 kDa precursor protein MelA1 contains an N-terminal secretion signal peptide. The protein is predicted by Alphafold 3 to form a dimer, which was further validated by size-exclusion chromatography (Fig. 3c). In this gene cluster, the ChrH-like C-terminal domain is separately encoded as a small protein which we call MelH_c_. Coexpression of His_6_-tagged MNIO (MelH) pulled down untagged MelH_c_ during purification, confirming that MelH_c_ forms a stable heterodimeric complex with MelH (Supplementary Fig. 19). We structurally modelled this complex together with the substrate MelA1 with Alphafold 3 (Fig. 3d). Complexes of MelH/H_c_ with the MelA1 dimer or its monomer produced similar predicted local distance difference test (pLDDT) values (Fig. 3d and Supplementary Fig. 20) Interestingly, both models predict that the CPACGMG motif from MelA1 is positioned into the active site of MelH in the complex.

Coexpression in *E. coli* of the precursor protein MelA1 with the MNIO MelH did not yield any modification (Fig. 3e). However, coexpression of MelA1 with MelH and MelH_c_ (the separate protein homologous to the ChrH C-terminal domain, Fig. 1) led to production of the unmethylated (−4 Da) product at the CPACGMG motif, further demonstrating the diversity of substrates processed by this MNIO subfamily. In turn, it suggests that the product structure can fulfill its currently unknown physiological function both at the end of a > 30 kDa secreted protein or as a small peptide.

### Substrate recognition by the MNIO

**Fig. 4.**
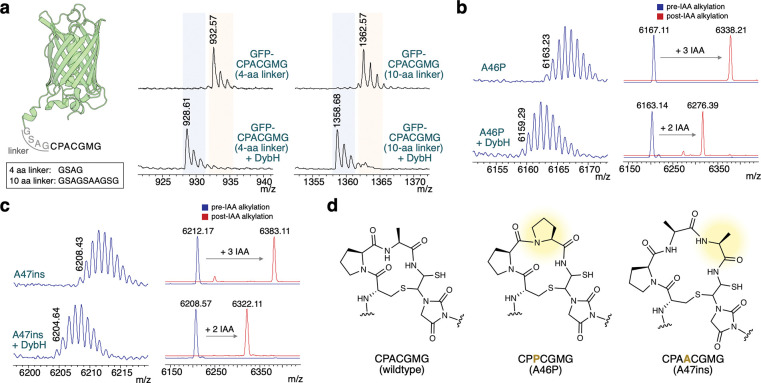
Substrate recognition and tolerance of DybH. **a** Diagram of the Green Fluorescent Protein-CPACGMG construct with a four amino acid linker (GSAG). Another construct with a ten amino acid linker (GSAGSAAGSG) was also tested. Right: MALDI-TOF MS of the C-terminal GFP-CPACGMG fragment (from LysC-digestion) expressed alone or together with DybH, with the 4- or 10-amino acid linker. Expected monoisotopic [M+Na]^+^ masses of C-terminal fragments, for the four-amino acid linker, 932.30 Da unmodified, 928.27 Da modified and for the ten amino acid linker, 1362.48 Da unmodified, 1358.45 Da modified. **b** MALDI-TOF MS of the DybA A46P (CPPCGMG) mutant expressed in the presence or absence of DybH showing the −4 Da modification. Left: Peptides before (blue trace) and after (red trace) IAA alkylation of the corresponding samples to determine the number of free thiols in the product. Three carbamidomethylations (each 57.05 Da) were observed for the unmodified peptide, and two for the product. Spectra collected in linear positive (LP) mode. **c** MALDI-TOF MS of the DybA A47ins (CPAACGMG) mutant expressed in the presence or absence of DybH showing the −4 Da modification. Left: Peptides before (blue trace) and after (red trace) IAA alkylation. **d** Chemical structures of the products obtained, showing the wild-type macrocycle in DybA, a macrocycle with two prolines (A46P mutant), and an expanded macrocycle (A47ins mutant).

Motivated by the natural diversity of precursor peptide and protein substrates in the ChrH subfamily (which appear to minimally share the conserved CPxCxMG motif), we tested whether the C-terminal 7-amino acid CPACGMG motif alone was sufficient for substrate recognition and modification. We appended the motif to the C-terminus of a globular protein, green fluorescent protein (GFP). Again, Alphafold 3 modelling of the GFP substrate and DybH predicted that the CPACGMG motif is inserted into the active site of DybH (Supplementary Fig. 21). Upon coexpression with DybH, we indeed observed the anticipated modification, showing that this motif is sufficient for substrate recognition and modification (Fig. 4a). Varying the linker length of the 7-amino acid tag to GFP still resulted in modified protein, further demonstrating that DybH solely recognizes the CPACGMG motif.

We hypothesized that the β-turn induced by the Pro-Ala in the CPACGMG sequence would be important for such a complex chemical transformation. Surprisingly, when additional conformational restraint was introduced by using two consecutive prolines in the macrocycle (CPPCGMG), we still observed formation of a −4 Da product (Fig. 4b). Alkylation with IAA showed one less adduct compared to the unmodified peptide, consistent with macrocyclization (Fig. 4b). Motivated by this tolerance, we also tested whether a larger macrocyclic ring could be formed by inserting an additional alanine to generate a C-terminal CPAACGMG sequence. Upon coexpression, the larger macrocycle could still be formed by the enzyme (Fig. 4c and d). Alphafold 3 structural prediction of the DybA and DybH complex shows that the side chains of the residues in the macrocycle point away from the active site, likely explaining why these sites are tolerant to substitutions (Supplementary Fig. 22).

### The product of the dyb BGC is a RiPP-lipoprotein hybrid

**Fig. 5.**
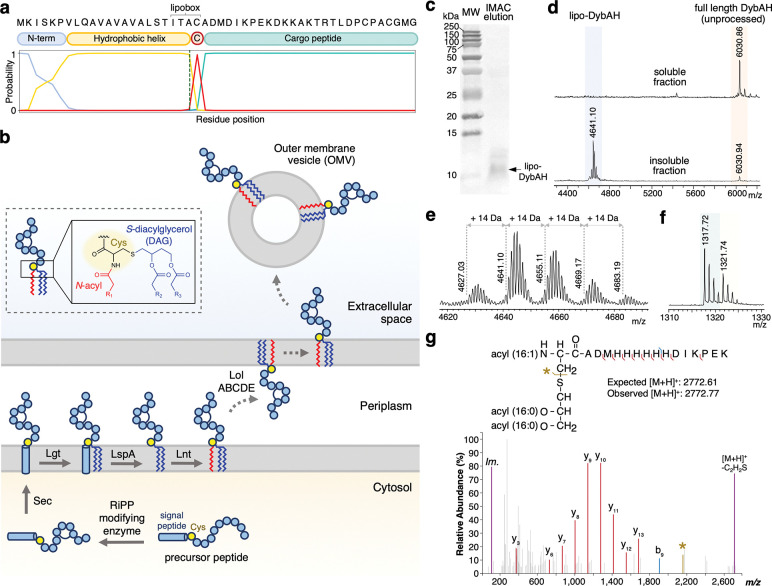
The product derived from *dybA* is a RiPP-lipoprotein. **a** Sequence and SignalP 6.0 prediction of DybA, showing the N-terminal positively charged sequence, the hydrophobic helix, the lipobox motif, the invariant internal cysteine, and the cargo peptide. The modified CPACGMG motif is bolded at the end of the peptide. **b** Proposed biosynthetic pathway for DybA maturation via the gram-negative bacterial lipoprotein pathway. DybA is first modified by cytosolic DybH before modification with a diacylglyceryl group (blue) by prolipoprotein diacylglyceryl transferase (Lgt), signal peptide cleavage by lipoprotein signal peptidase (LspA/SPII), *N*-acylation (red) by apolipoprotein N-acyltransferase (Lnt), and potential transport to the outer membrane and extracellular space. Inset: Structure of the modified N-terminal Cys in a triacylated lipoprotein containing an *S*-diacylglycerol and *N*-acyl group. **c** Ponceau S stained nitrocellulose membrane showing the eluate from lipo-DybAH IMAC purification, following transfer from a 16.5% tris-tricine gel. An arrow points to the band corresponding to lipo-DybAH, which was excised for LysC digestion and MS/MS identification. **d** MALDI-TOF MS of lipidated DybAH purified from *E. coli* using Ni-affinity chromatography. The eluate precipitated and both the soluble (upper trace) and insoluble fraction (lower trace) were desalted and analyzed by mass spectrometry. **e** Isolated lipo-DybA shows the presence of at least four differently lipidated species, with the major species containing C16:1, C16:0, C16:0 acyl chains. **f** LysC-digest of the lipoprotein-enriched fraction shows that lipidated DybA is partially unmodified (tan) and partially modified (teal) by DybH. **g** MALDI-TOF MS/MS tandem mass spectrum of the excised lipo-DybAH nitrocellulose band following LysC digestion, showing the triacylated form of lipo-DybAH [C16:1, C16:0, C16:0]. A fragmentation ion corresponding to loss of a C_2_H_2_S group, and the immonium ion (*Im.*) of histidine were also detected.

While we initially hypothesized that the internal cysteine in DybA and DybA-like precursor peptides could be a site for modification by the MNIO, we only observed modification at the CPACGMG motif. By analyzing the N-terminal sequence in more detail, we noticed a stretch of hydrophobic amino acids that precede the conserved cysteine. Indeed, the signal peptide prediction program SignalP predicts that DybA contains an N-terminal signal that is a substrate for signal peptidase II (SPII), which is involved in lipoprotein maturation^28^ (Fig. 5a). Conversely, when we analyzed the N-terminus of ChrA and MelA1 by SignalP, we observed that they contain secretion signals that are substrates for signal peptidase I (SPI), which does not result in lipidation (Supplementary Fig. 23). In peptides that contain a lipoprotein signal, the conserved cysteine and the three preceding residues constitute the lipobox motif^29^. The precursor peptides that contain the additional third Cys residue all contain canonical lipobox motifs, thus explaining why the cysteine is invariable at that position (Fig. 5a). Therefore, following DybA modification by DybH in the cytoplasm (since DybH does not contain any signal peptides), the modified peptide is predicted to be transported to the inner plasma membrane, where a diacylglycerol (DAG) group is appended to the Cys thiol (Fig. 5b). The signal peptide is then cleaved off by signal peptidase II, and another acyl chain is often added to the N-terminal amine of the Cys residue in gram-negative bacteria^29^. Depending on the specific sorting signal (largely determined by the identity of the residue immediately following the lipobox cysteine)^30^, the RiPP-lipoprotein could be transported to the outer membrane by the localization of lipoproteins (Lol) machinery, and further be secreted in the form of bacterial outer membrane vesicles (OMVs)^31^.

To probe the possibility that the product of the *dyb* BGC could be a RiPP-derived lipoprotein, we removed the N-terminal His-tag to expose the signal peptide for processing. To facilitate purification, the His-tag was inserted between Met26 and Asp27 within the peptide sequence, which is after the lipoprotein signal peptide, but before the C-terminal CPACGMG motif to prevent interference with the modification by DybH (Supplementary Fig. 24). Following heterologous expression in *E. coli*, membrane solubilization, and purification by nickel-affinity chromatography, a 16.5% tris-tricine gel showed the successful isolation of a small peptide (Fig. 5c). The IMAC eluate precipitated, which we reasoned could be due to the insoluble, highly non-polar lipoprotein. Indeed, analysis of the soluble fraction by MALDI-TOF MS showed enrichment of the DybH-modified full-length peptide (unprocessed by the lipoprotein machinery), whereas the insoluble fraction showed enrichment of a series of smaller peptide peaks that are consistent with lipidated forms of DybAH (Fig. 5d, lipo-DybAH). The observed peaks are separated by 14 Da (CH_2_), consistent with the attachment of lipids with varying chain lengths (Fig. 5e). Furthermore, digestion of the lipoprotein fraction by LysC confirmed that the majority of the lipidated product is modified by DybH, showing the anticipated −4 Da mass change (Fig. 5f).

To determine which lipids were attached to the *N*-acyl, *S*-diacylglyceryl-cysteine, the lipopeptide band from the 16.5% Tris-Tricine gel was transferred to a nitrocellulose membrane and excised for digestion with LysC^32^ (Fig. 5d). Elution in a 2:1 (v/v) mixture of chloroform and methanol resulted in the digested N-terminal lipoprotein fragment. Tandem MS analysis by MALDI-TOF/TOF confirmed the formation of a triacylated lipoprotein that primarily contained C16:1, C16:0, C16:0-derived lipids appended to the N-terminal cysteine via a glycerol linker (Fig. 5g, Supplementary Fig. 25). The lengths of the acyl chains are consistent with the lipids identified in previously characterized *E. coli* lipoproteins^33,34^. While these acyl chain lengths are affected by membrane lipid composition^35^ (which likely differs in the native host *D. crusticola*), these results clearly show that DybA contains a signal peptide for lipidation via the ubiquitous bacterial lipoprotein biosynthesis pathway.

### Bioinformatic mining for other RiPP-lipoprotein hybrids

**Fig. 6.**
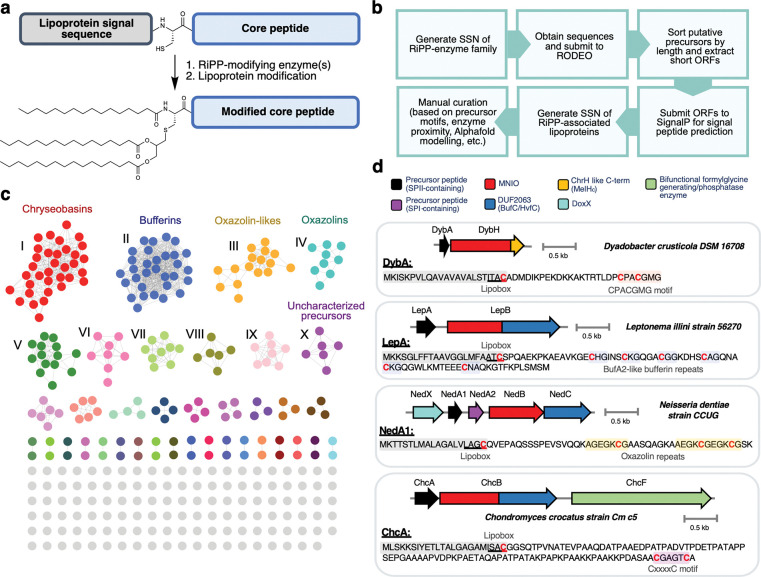
Genome mining for other RiPP-lipoproteins. **a** Diagram for RiPP-lipoprotein maturation. Shown here is an example of the triacylated form of the lipoprotein [C16:0, C16:0, C16:0]. **b** Bioinformatic pipeline for RiPP modifying enzyme directed discovery of RiPP-lipoproteins. During the SSN generation process, similar sequences can be filtered out using Uniref90 database or using a representative node network. **c** SSN of MNIO-associated lipoproteins (Cytoscape file provided as Dataset 2). Ten of the largest clusters are annotated I-X. Clusters V-IX correspond to putative non-RiPP lipoproteins that are found in (or adjacent to) MNIO-containing gene clusters. V: BON-domain containing protein in bufferin BGC. VI: LPS translocon maturation chaperone LptM in bufferin BGC. VII: Glycine zipper two transmembrane domain-containing protein in bufferin BGC. VIII: TssQ family T6SS-associated lipoprotein in bufferin BGC. IX: Hypothetical protein in bufferin BGC. The full list of sequences can be found in Supplementary Table 6. **d** Representative examples of predicted BGCs encoding RiPP-lipoproteins from the different precursor peptide clusters. The characterized RiPP families corresponding to chryseobasins, bufferins, and oxazolins all show examples of lipidation via the SPII pathway.

A subset of bufferins (a group of MNIO-modified RiPPs) have recently been predicted to contain a signal peptide like the one in DybA suggesting they are lipidated^10^. Therefore, we hypothesized that other bacterial RiPP-lipoprotein hybrids likely exist in nature that exploit the ubiquitous SPII pathway for lipidation (Fig. 6a). We developed a bioinformatic pipeline to facilitate the global discovery of RiPP-lipoproteins, which we used here for the MNIO family (Fig. 6b). Two possible approaches can be envisioned for discovery of these hybrid products: either starting with lipoprotein prediction followed by RiPP prediction, or starting with RiPP prediction followed by lipoprotein prediction. Starting with RiPP precursor peptide prediction ensures that the resulting peptides are within or adjacent to RiPP BGCs (thus RiPP-related) and narrows down the initial list of putative lipoproteins significantly. The list of precursor peptides can then be filtered based on the presence of a lipidation signal. Finally, a second SSN of the putative RiPP-lipoproteins can be used to group hits with similar gene contexts.

We utilized this approach to mine for precursor peptides that co-occur with MNIOs. From the local gene neighborhood of genes encoding MNIOs obtained from RODEO^36^, ORFs were extracted and sorted based on length. A length of 50–160 amino acids was used as the cutoff for peptide precursors. These peptides were then analyzed for a signal peptide using SignalP 6.0^28^. An SSN of the putative RiPP-lipoproteins was generated to group together related sequences (Fig. 6c). The clusters and corresponding BGCs were then inspected as potential RiPPs.

As expected, chryseobasin-like peptides (the products of *chr*-like BGCs from Bacteroidota) represented one of the largest clusters of precursor peptides predicted to be lipidated (Fig. 6c and d, Cluster I). Additionally, a subset of the previously characterized copper-chelating peptide families (both bufferins^13^ and oxazolins^12^) contained SPII lipoprotein signals. Interestingly, we also found a group of uncharacterized, putative precursor peptides that contain lipoprotein signals (Fig. 6c and d, Cluster X). These peptides contain a highly conserved CxxxxC motif that is the likely site for MNIO modification.

**Fig. 7.**
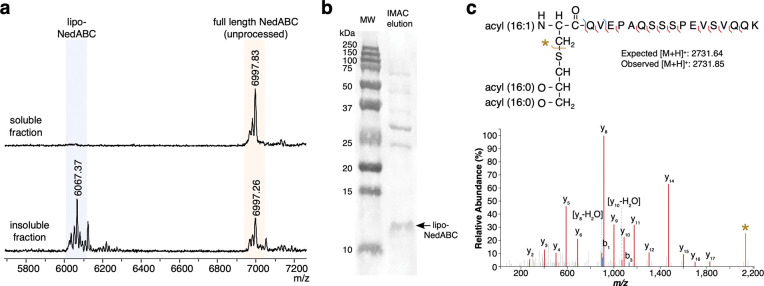
Heterologous production of lipo-NedABC in *E. coli*. **a** MALDI-TOF mass spectra of IMAC-purified C-terminally His-tagged NedA that was coexpressed with NedBC. Following Ni-affinity purification, the eluate precipitated, and both the soluble (upper trace) and insoluble pellet (lower trace) were desalted and analyzed by mass spectrometry. **b** Ponceau S stained nitrocellulose membrane with the arrow pointing to the band corresponding to lipo-NedABC, which was excised for LysC digestion and MS/MS identification. The IMAC eluate was first run on a 16.5% tris-tricine gel, then transferred to the nitrocellulose membrane. **c** MALDI-TOF MS/MS tandem mass spectrum of the excised lipo-NedABC nitrocellulose band following LysC digestion, showing the triacylated form of lipo-NedABC [C16:1, C16:0, C16:0].

NedA from *Neisseria dentiae* CCUG 53898 310003 (WP_085365104.1) was predicted to be a lipidated, copper-binding oxazolin as it contains the SPII lipoprotein signal in addition to the conserved repeated EGKCG motifs^12^ (Fig. 6d). To confirm that NedA is a RiPP-lipoprotein, we heterologously expressed in *E. coli* the C-terminally His-tagged NedA with its modifying enzymes (the MNIO NedB and its substrate-binding partner protein NedC). Using the same lipoprotein purification method as for lipo-DybAH, we successfully isolated tri-acylated lipo-NedABC from the insoluble membrane fraction (Fig. 7a and b). Digestion of this lipoprotein with LysC, and MS/MS analysis of the major N-terminally lipidated fragment showed that the attached lipids are derived from palmitic and palmitoleic acids [16:1, 16:0, 16:0] (Fig. 7c and Supplementary Fig. 26). In addition, MS analysis of the core peptide showed a −12 Da modification, which is consistent with a −4 Da modification at each of the three EGKCG motifs by NedBC (Supplementary Fig. 26). Whether the modification at the EGKCG motif corresponds to formation of a thioamide-oxazolone or 5-thiooxazole will require more investigation given the different assignments made by other groups^12,37^. Nevertheless, these experimental results confirm our initial bioinformatic prediction, showing that the product derived from NedA is a RiPP-lipoprotein. Since the related peptide MmrA from *Microbulbifer* sp. VAAF005 (which contains the conserved EGKCG repeats) is proposed to be involved in protection against copper toxicity^37^, it is possible that lipo-NedABC sequesters copper at the membrane to prevent entry into the cell. Furthermore, copper-bound lipopeptides could be secreted from the cell in the form of OMVs.

Here, we show application of the RiPP-lipoprotein mining approach for the MNIO family, although this method could be applied to any RiPP-modifying enzyme family. RiPPs most likely to be lipidated via this pathway involve short biosynthetic pathways that are leader-peptide independent. Conversely, RiPPs that rely on N-terminal cleavage or cyclization, such as lasso peptides and bottromycins, are likely incompatible with an N-terminal signal peptide^1^. Aside from the MNIO-modified precursors, our preliminary bioinformatic analyses also show that a subset of autoinducing peptides (AIPs)^38^ appear to contain canonical SPII signal peptides (Supplementary Fig. 27). In AIP biosynthesis, the thiol of a conserved internal cysteine typically cyclizes with the C-terminal carboxylate to generate a thiolactone macrocycle (catalyzed by the modifying enzyme AgrB)^39^. We found examples of unusual AIP precursors that contain two cysteines, suggesting that the first cysteine is used as the glycerolipid attachment site, and the second for macrocyclization (Supplementary Fig. 27, Supplementary Table 7). However, not all the predicted SPII-signal-containing AIPs contain two cysteine residues. Hence, whether these particular AIPs truly form lipoproteins (or whether the glycerolipid attachment site is blocked by formation of the thiolactone macrocycle) is presently not clear.

## Discussion

The MNIO family is a rich source for diverse post-translational modifications^8,10^. Here, we present a ChrH-like modification that is performed by several members of Bacteroidota MNIOs. The diverse substrates of this subfamily include secreted peptides, secreted proteins, and lipoproteins that share a C-terminal modification. The two previous examples of thiohemiaminals formed by MNIOs involved alkylation reactions of the thiol. In one example, the alkylation involved carboxymethylation by a dedicated carboxy-*S*-adenosyl methionine dependent enzyme^14,40^. In the second example, the MNIO itself appears to methylate the thiol of the thiohemiaminal as this product was obtained both in vitro and in *E. coli*^15^. DybH and its orthologs reported herein on the other hand form the thiohemiaminal as a stable product that is not further alkylated based on the genes in their BGCs. The function of the final products is currently still unclear.

Recognition of the minimal C-terminal motif likely provides relaxed purifying selection on the N-terminal sequence (which is typically constrained in RiPP biosynthesis by a mandatory leader peptide), thus explaining the broad structural diversity observed at the N-terminus of these substrates. This motif, when installed on the C-termini of wild-type (MelA1) or engineered (eGFP) proteins was successfully modified. Using DybA mutants, we also demonstrate that the DybH enzyme is unexpectedly tolerant to substitutions and expansions within the macrocycle.

As noted above, the biological function of the lipoprotein products is still unknown. In both Cyanobacteria and *Chryseobacterium*, these peptides are secreted via SPI, suggesting a periplasmic or extracellular function. In other Bacteroidota genera, the lipoproteins are membrane bound, but could also be liberated from the membrane by non-specific proteases, or released in outer membrane vesicles. The BGC in *Spirosoma endophyticum* encodes a prolyl oligopeptidase with a secretion signal, which could cleave the lipidated peptide (Supplementary Fig. 28). Furthermore, the gene clusters frequently co-occur with or contain genes encoding SusC and SusD type importers. The SusCD import machinery is involved in the uptake of oligopeptides or oligosaccharides, and are commonly found in polysaccharide utilization domains (PULs)^41^. We also note that the macrocyclic ring of these highly conserved MNIO products is structurally similar to the highly conserved B ring of lantibiotics such as nisin^42^, epidermin^43^, and mutacin 1140^42–44^(Supplementary Fig. 29). The B-ring of these lantibiotics is part of the binding site of the pyrophosphate of the lipid II target^45^. Future work will focus on elucidating the function of the lipoproteins.

The discovery of RiPP-lipoprotein hybrids demonstrates an example of specialized metabolism co-opting pathways involved in the general maturation of proteins. Our initial bioinformatic searches show that several products from other MNIO subfamilies are lipidated via the lipoprotein pathway. These results also suggests that additional RiPP-lipoproteins, beyond those modified by MNIOs, may exist in nature. The observation here that lipidated DybA and NedA are modified by their RiPP maturation enzymes demonstrates that their posttranslational modification (by DybH and NedBC respectively) is sufficiently fast to compete with export via the membrane bound lipoprotein pathway. We note that these DAG-RiPPs are distinct from other RiPP-derived lipopeptides, which are generated by acylation of the side chain(s) or N-terminus of various RiPPs^23,46^. While different lipidated forms of the N-terminus of bacterial lipoproteins have been observed (including triacylated, diacylated, lyso- and ‘peptidyl’-forms)^29^, their biosynthesis shares a common step involving the attachment of a DAG group from a membrane phospholipid to the side chain of cysteine.

Several characterized bacterial lipoproteins are immunogenic and potent activators of Toll-like receptors^35,47^. The use of RiPP-modifying enzymes could enable post-translational modifications of immunogenic lipoprotein adjuvants to expand their structural diversity beyond the canonical 20 amino acids and improve pharmacological stability.

## Methods

### Materials

Primers and gene fragments were ordered from Integrated DNA Technologies and Twist Biosciences, respectively. Antibiotics, IPTG, DTT, TCEP, and lysozyme were purchased from GoldBio. LysC endoprotease was ordered from New England Biolabs (NEB). Deuterated NMR solvents were sourced from Cambridge Isotope Laboratories. Q5^®^ High-Fidelity DNA Polymerase, NEBuilder^®^ HiFi DNA Assembly, and DpnI restriction enzyme were purchased from NEB. Pam2CSK4 and Pam3CSK4 synthetic lipoprotein standards were purchased from InvivoGen. The MALDI matrices super-DHB and CHCA were obtained from Millipore Sigma. All accession numbers for proteins in this study are provided in Supplementary Table 8.

### Cloning

All plasmid constructs were initially assembled by Gibson assembly of DNA fragments with PCR-amplified vector backbones (primers and gblocks listed in Supplementary Table 9). The genes *dybA* and *dybH* were directly amplified from the extracted gDNA of *Dyadobacter crusticola* strain DSM 16708. Prior to DNA extraction, *D. crusticola* was cultured in R2B media, shaking at 220 rpm at 28 °C to late mid-log phase. The other genes used in this study were obtained by synthetic gene fragments (Supplementary Table 9). Subsequent changes to the plasmids (site-directed mutagenesis, deletions, and short insertions) were performed by PCR with partially overlapping primers, and then digesting the template plasmid with Dpn1. The unpurified digestion reaction (2 μL) was directly used to transform *E. coli* 5α (NEB) chemically competent cells. The sequences of all plasmids were verified by Plasmidsaurus prior to transformation of the *E. coli* BL21 (DE3) expression strain.

### Heterologous expression and purification of peptides

*E. coli* BL21 (DE3) cells were transformed with the appropriate plasmids and plated on selective media (Supplementary Table 10). Transformants were inoculated in an overnight culture of LB with antibiotic(s). Antibiotics were used at the following concentrations: 33 μg/mL chloramphenicol, 50 μg/mL streptomycin, 50 μg/mL kanamycin. The overnight culture was used to inoculate either 200 mL of LB (small scale) or 4 L of terrific broth (TB) media (large scale). The cultures were grown shaking at 240 rpm, 37 °C until the OD_600_ reached 0.7–0.9. The flasks were cooled on ice for 20–30 min. For co-expressions with MNIOs, a freshly prepared 1000× stock solution of iron(II) sulfate heptahydrate and trisodium citrate was added to a final concentration of 350 μM and 1 mM, respectively. IPTG was added to 0.7 mM final concentration, and the flasks were placed back in the shaker at 18 °C, 220 rpm, for 18–20 h. The cells were harvested by centrifugation at 7000 g for 10 min, washed once with distilled (DI) water, then stored at −80 °C until further use.

Cell pellets were thawed at room temperature, then an equal volume of DI water was added (containing 0.5× Pierce EDTA-free protease inhibitor). After stirring at 4 °C for 30 min, four volumes of denaturing lysis buffer (6 M guanidine hydrochloride, 20 mM sodium phosphate dibasic, 500 mM sodium chloride, 10 mM imidazole, pH 7.5) was added. The cell solution was sonicated until clear (10 s on, 10 s off cycle, 30 min total). The lysate was clarified by centrifugation at 40 000 g for 30 min. The supernatant was decanted into 0.5 mL (small scale) or 5 mL (large scale) of equilibrated Ni-NTA agarose beads, and nutated at 4 °C for 30 min. The beads were transferred to a column and washed sequentially with 20 column volumes (CV) of 4 M guanidine hydrochloride, 20 mM sodium phosphate dibasic, 300 mM sodium chloride, 30 mM imidazole, pH 7.5 followed by 20 CV of 20 mM sodium phosphate dibasic, 500 mM sodium chloride, 30 mM imidazole, pH 7.5. The peptide was eluted using 10 CV of 20 mM sodium phosphate dibasic, 500 mM sodium chloride, 500 mM imidazole, pH 7.5.

For small scale purifications, the IMAC eluate was directly desalted with a C18 Ziptip. For large scale purification, the eluate was desalted using a 2 g C18 Hypersep SPE column, and eluted in 50–60% acetonitrile with 0.1% trifluoroacetic acid (TFA). The desalted peptide was concentrated to dryness under reduced pressure. To obtain full length peptide, the residue was directly resuspended in water and injected onto an Agilent HPLC equipped with an analytical Grace Vydac C18 column (250 mm × 4.6 mm, 300 Å pore size, 5 μm particle size) at a flow rate of 1 mL/min with the following gradient: 2% (H_2_O + 0.1% TFA:MeCN + 0.1% TFA) for 4 min, 2–50% for 30 min, 50–90% for 15 min). Fractions containing the desired peptide, judged pure by matrix-assisted laser desorption/ionization time-of-flight mass spectrometry (MALDI-TOF MS), were combined and lyophilized. The peptide was then stored at −20 °C and resuspended in water when needed.

### LysC digestion

To obtain digested peptide, the lyophilized, desalted full length peptide was first resuspended in 1 mL of 10 mM Tris-HCl buffer pH 8, with 2 mM TCEP, to which 20 μL of LysC (0.1 ug/μL, NEB) was added. The digest was incubated at 37 °C overnight. The digest was then injected onto HPLC using the same conditions as above with the following modification to the gradient: 2% B for 4 min, then 2–30% B for 30 min, 30–90% B for 15 min). Fractions containing the desired peptide fragment were lyophilized.

### Heterologous expression and purification of DybH

BL21 (DE3) cells were transformed with pET28a:His-TEV-DybH. Protein expression was carried out as with peptide expression described above. The cell pellet was resuspended in protein lysis buffer (50 mM HEPES, 200 mM NaCl, pH 7.6) containing 0.5x Pierce EDTA-free protease inhibitor, 2 mg/mL lysozyme, and 10 μg/mL DNAse I. Cells were stirred at 4 °C for 15 min, then lysed by sonication (10 s on, 10 s off cycle, 20 min total). Following centrifugation at 40,000 g for 40 min, the clarified lysate was loaded onto equilibrated HisPur^™^ Nickel-nitrilotriacetic acid resin, and washed with 20 CV of lysis buffer. The protein was eluted in the same buffer containing 300 mM imidazole. The fractions containing the most protein were pooled and immediately injected onto a HiLoad 16/60 Superdex 200 pg column equilibrated with storage buffer (25 mM HEPES, 200 mM NaCl, pH 7.5, 10% glycerol). Fractions containing purified protein, judged pure by SDS-PAGE, were pooled and concentrated to approximately 20 mg/mL, at which point they were flash frozen in liquid N_2_ and stored at −80 °C.

### In vitro assays

To 50 μL of assay buffer (25 mM HEPES, 200 mM NaCl, pH 7.6) was added DybA (75 μM), TCEP (1 mM) and DybH to a final concentration of 15 μM. The assay was incubated at room temperature for 1 h before the addition of acetonitrile to a final concentration of 10 % and TFA to 0.2 %. The reaction product was desalted by a C18 Zip tip and analyzed by MALDI-TOF MS.

### MALDI-TOF MS and MS/MS

Samples were desalted using a C18 Zip tip (0.6 μL bed volume) then eluted in 1 μL of 80% acetonitrile containing 0.1% TFA and 50 mg/mL of the MALDI matrix super-DHB. The eluate was spotted on a plate, dried, and analyzed on a Bruker UltrafleXtreme MALDI-TOF instrument using positive reflectron mode (RP) for peptides, or linear mode (LP) for proteins. Masses were calibrated using a set of peptide and protein standards (bradykinin, angiotensin II, ACTH clip 18–39, insulin, cytochrome C, and bovine serum albumin). MS/MS spectra were collected in LIFT positive mode.

### Iodoacetamide alkylation

The peptide was resuspended in 50 mM Tris, pH 8.3. Dithiothreitol was added to 6 mM final concentration and the reaction was incubated at 55 °C for 10 min. Iodoacetamide was added to 20 mM final concentration, and the reaction was placed at RT in the dark for at least 1 h. The alkylated peptide was purified by a C18 Ziptip, or by C18 HPLC as described previously.

### LC-MS/MS

Desalted samples were analyzed on an Agilent 6545B Q-TOF and 1290 Infinity II LC system using a Phenomenex Aeris C18-XB column (150 mm × 4.6 mm, 100 Å pore size, 2.6 μm particle size) at a flow rate of 0.6 mL/min, and the following gradient: 5% (H_2_O + 0.1% FA:MeCN + 0.1% FA) for 3 min, 5–70% for 12 min, 70–90% for 3 min. The collision energy was set to 25–30 mV and ions were detected in positive mode. Fragments were initially assigned by using the Interactive Peptide Spectral Annotator (IPSA)^48^ and manual curation.

### NMR spectroscopy

Lyophilized digested peptide was resuspended in either 280 μL of 90% H_2_O:10% D_2_O or 100% D_2_O and transferred to a D_2_O matched Shigemi tube, or in 100% DMSO-d6 in a DMSO-d6 matched Shigemi tube. The ^1^H NMR spectrum was collected on a 750 MHz Agilent VNMRS with a 5 mm HCN probe. HSQC, HMBC, and NOESY data was collected on a 600 MHz Bruker NEO spectrometer with a 5 mm broadband Prodigy CryoProbe. Mnova was used for data analysis and chemical shifts were referenced to the solvent.

### Ferene S iron quantification assay

Total iron in enzyme samples was quantified against a standardized iron solution (1000 mg/L Fe in 0.5 M HNO_3_, Certipur^®^ certified reference material) using the ferene S reagent, in a similar manner as described previously^49,50^. Briefly, to 300 μL of diluted enzyme sample and iron standards was added 300 μL of reagent A (155 mM SDS, 75 mM iron-free sodium acetate). The solution was thoroughly mixed, then 300 μL of reagent B (1 M sodium ascorbate, 8 mM sodium metabisulfite, 50 mM iron-free sodium acetate) was added. The solution was thoroughly mixed and incubated at 37 °C for 15 min. Then, 15 μL of reagent C (18 mM ferene S) was added, and the absorbance was read at 593 nm. The iron concentration in enzyme samples was determined using a standard curve of serial dilutions of the iron standard. The resulting iron concentration was normalized to the protein concentration (determined by the BCA assay) to yield the number of iron atoms per enzyme protomer.

### Lipoprotein expression and purification

Lipoprotein constructs were transformed and expressed in *E. coli* BL21 (DE3) cells as above, with the exception that LB was adjusted to pH 8, and at induction, only IPTG was added to 0.7 mM, without additional iron supplement. The cells were incubated shaking at 30 °C for 12 h at 220 rpm.

For purification, cells were first resuspended in lysis buffer (25 mM HEPES, 300 mM NaCl, pH 7.5). Lysozyme (2 mg/mL lysozyme) and DNAse I (10 μg/mL) were added, and the mixture was incubated at room temperature for 5 min, then sonicated (10 s on, 10 s off cycle, 15 min total). Unbroken cells were removed by centrifugation at 7000 g for 8 min. To collect membranes, the supernatant was subjected to ultracentrifugation in a Beckman TL-100 ultracentrifuge at 100 000 g for 20 min. The supernatant was discarded, and the membrane was resuspended in 0.5× initial volume of buffer by pipetting up and down to homogenize. To the mixture was added n-octyl-β-d-glucopyranoside (OG) and Zwittergent 3–14 to 1% (w/v) and 0.5% (w/v) respectively. The addition of Zwittergent 3–14 has been previously shown to help solubilize lipoproteins from membranes^51^. The mixture was incubated for 30 min nutating at RT. Insoluble precipitates that remained were removed by centrifugation at 100 000 g for 10 min. The supernatant was loaded onto 250 μL of Ni resin in a 5 mL tube and nutated at 4°C for 1 hr. The resin was transferred to a chromatography column and washed with lysis buffer containing 0.05% Zwittergent 3–14 and 30 mM imidazole. For the final 20 mL of wash and elution (containing 500 mM imidazole), no detergent was added. The elution was collected in 100 μL fractions, several of which formed precipitates (especially after freeze-thawing).

If the eluate precipitated (which can take several hours at 4°C or freeze-thawing), then a pellet was obtained by centrifugation at 16 000 g for 10 min. The supernatant was removed, and the pellet was gently washed with 50 mM ammonium bicarbonate pH 7.8 twice to desalt the sample. To solubilize the pellet, 100 μL of 0.1% TFA was added, and the mixture was sonicated in a water bath. The sample (1 μL) was mixed with 1 μL of 80% acetonitrile containing 0.1% TFA and 50 mg/mL of super-DHB then spotted on a MALDI target plate.

If the eluate did not precipitate, it was desalted on a PD MiniTrap G-10 column (Cytiva) into milliQ water. The sample was concentrated using a SpeedVac vacuum concentrator, then (1 μL) was directly mixed with 1 μL of 80% acetonitrile containing 0.1% TFA and 50 mg/mL of super-DHB then spotted on a MALDI target plate.

### Isolation of the N-terminally lipidated fragment for MALDI-TOF MS/MS analysis

The N-terminal lipid modification was confirmed by tandem mass spectrometry using a variation of a method described previously^32^. The IMAC eluate was first run on a 16.5% tris-tricine SDS-PAGE gel (BioRad). Proteins were transferred to a 0.2 μm nitrocellulose membrane using a wet transfer method for 1 h at 110 mV. Ponceau S was used to visualize the peptide bands, which were excised and transferred to a protein low-bind microfuge tube (Eppendorf). The membrane pieces were fully destained with two washes of water and three washes of 50 mM ammonium bicarbonate, pH 7.8. Endoprotease LysC (10 μL of a 20 μg/μL solution in ammonium bicarbonate) was added to cover the nitrocellulose pieces, and the digest was incubated for 16 h. At the final hour of digestion, DTT was added to 0.1 mM. The liquid was removed, followed by sequential washings with 10 uL of 10%, 20% MeCN + 0.1% TFA, and 5 uL of and 80% MeCN + 0.1% TFA. Finally, 5 μL of a mixture of CHCl_3_:MeOH (2:1 v/v) was used to elute the lipopeptides from the nitrocellulose membrane. The mixture (1 μL) was mixed with 1 μL of a 15 mg/mL α-cyano-4-hydroxycinnamic acid (CHCA) matrix dissolved in CHCl_3_:MeOH (2:1 v/v) and spotted on a MALDI target plate. The synthetic lipoproteins Pam2CSK4 and Pam3CSK4 were used as standards to validate the efficacy of the method.

### Phylogenetic and bioinformatic analyses

Protein sequences were uploaded to MEGA11^52^ and the MUSCLE algorithm was used to align sequences. Using MEGA11, the aligned sequences were used to build a maximum likelihood phylogenetic tree using the Jones-Taylor-Thornton model and 90% partial deletion.

For prediction of signal peptides in DybA or ChrA, individual sequences were submitted to the SignalP 6.0 server^53^. For genome mining of MNIO-RiPP-lipoproteins, an SSN for the MNIO family (PF05114) was first constructed using the EFI-EST program^24,54^, against the UNIREF90 database. The alignment score was set to 30, and enzymes were filtered based on length from 220 to 50,000 amino acids (default maximum length). The MNIO sequences retained from this filter totalled 8227 sequences. The Uniprot accessions were converted to RefSeq Protein accessions (using the ID Mapping tool from Uniprot), resulting in 4360 converted sequences, which were submitted to RODEO^36^. The predicted precursor open reading frames (ORFs) were sorted by length, and peptides between 50–160 amino acids in length starting with a methionine residue were selected. These peptides were submitted to SignalP 6.0 for signal sequence prediction^28^. To generate an SSN of putative lipidated RiPPs, a list of all peptides with a predicted SPII signal sequence of over 95% confidence (339 total) were submitted to EFI-EST, using an alignment score of 1. Cytoscape 3.10.3 was used for network visualization, using yFiles Organic layout^53^.

## Supplementary Material

Supplement 1

Supplement 2

## Data Availability

Raw data are deposited on Mendeley. Chen, J. Y; Zhu, L; van der Donk, W.A. (2025), Data associated with “Co-opting the bacterial lipoprotein pathway in the biosynthesis of a lipidated macrocyclic peptide”, Mendeley Data, V1, doi: 10.17632/n97sjjc2nm.1
