# Supplementary material for: Co-opting the bacterial lipoprotein pathway in the biosynthesis of a lipidated macrocyclic peptide
Source: bioRxiv. 2025 Nov 1:2025.10.31.685832. Preprint. [Version 1] doi: 10.1101/2025.10.31.685832 (PMC12636315; doi:10.1101/2025.10.31.685832)
Supplement: Supplement 1 [file media-1.pdf]

Supplementary Information

Co-opting the bacterial lipoprotein pathway in the biosynthesis of a lipidated macrocyclic peptide

Jeff Y. Chen,[1,2] Lingyang Zhu,[3] Kevin Y. Zhang,[1,2] Deborah A. Berthold,[1] Wilfred A. van der Donk[1-2,*]

[1] Department of Chemistry and Howard Hughes Medical Institute, 600 South Mathews Avenue, University of Illinois at Urbana−Champaign, Urbana, Illinois 61801, USA.
[2] Carl R. Woese Institute for Genomic Biology, University of Illinois at Urbana-Champaign, 1206 West Gregory Drive, Urbana, Illinois, 61801, USA.
[3] School of Chemical Sciences NMR Laboratory, University of Illinois at Urbana-Champaign, Urbana, 61801, IL, USA.

Corresponding author: Wilfred A. van der Donk
Corresponding author email: vddonk@illinois.edu

Supplementary Figures

Supplementary Fig. 1. Characterized reactions catalyzed by multinuclear non-heme iron dependent oxidative enzymes (MNIOs). 3
Supplementary Fig. 2. Alphafold3 models of ChrH, DybH and MelH with MelH$_c$. 3
Supplementary Fig. 3. Assignment of fragmentation ions from modified DybAH from LC-MS/MS. 4
Supplementary Fig. 4. IAA-alkylation of LysC-digested DybAH. 4
Supplementary Fig. 5. UV-Vis characterization of DybA and DybAH peptides. 5
Supplementary Fig. 6. ^{1}H NMR spectrum of LysC-digested DybAH in 90% H_2O: 10% D_2O. 5
Supplementary Fig. 7. 2D ^{1}H-^{1}H TOCSY spectrum of LysC-digested DybAH in 90% H_2O: 10% D_2O. 6
Supplementary Fig. 8. 2D ^{1}H-^{13}C HSQC spectrum of LysC-digested DybAH in D_2O. 7
Supplementary Fig. 9. 2D ^{1}H-^{1}H NOESY spectrum of LysC-digested DybAH in in 90% H_2O: 10% D_2O. 8
Supplementary Fig. 10. ^{1}H NMR spectrum of IAA-alkylated LysC-digested DybAH in DMSO-d6. 8
Supplementary Fig. 11. 2D ^{1}H-^{13}C HSQC spectrum of IAA-alkylated LysC-digested DybAH in DMSO-d6. 9
Supplementary Fig. 12. 2D ^{1}H-^{1}H TOCSY spectrum of IAA-alkylated LysC-digested DybAH in DMSO-d6. 10
Supplementary Fig. 13. 2D ^{1}H-^{1}H COSY spectrum of IAA-alkylated LysC-digested DybAH in DMSO-d6. 11
Supplementary Fig. 14. 2D ^{1}H-^{13}C HMBC spectrum of IAA-alkylated LysC-digested DybAH in DMSO-d6. 12
Supplementary Fig. 15. Heterologous expression and UV-Vis characterization of DybH. 13
Supplementary Fig. 16. In vitro activity assay of DybH. 14
Supplementary Fig. 17. Proposed mechanism for DybH catalysis. 15
Supplementary Fig. 18. BGCs and sequences of ChrA, ChsA, HymA, ArlA, MelA1. 16
Supplementary Fig. 19. MelH and MelH$_c$ form a stable complex. 17
Supplementary Fig. 20. Structural prediction of the MelA1, MelH and MelH$_c$ complex. 18
Supplementary Fig. 21. Structural prediction of GFP-CPACGMG complexed with DybH. 19
Supplementary Fig. 22. Alphafold3 model of DybA with DybH. 20
Supplementary Fig. 23. SignalP analysis of secreted peptides in the ChrA-like family. 21
Supplementary Fig. 24. Sequence of DybAH construct with 6xHis tag at residue 27. 21
Supplementary Fig. 25. Full structure of lipo-DybAH with embedded 6xHis. 22
Supplementary Fig. 26. MALDI-TOF MS analysis of lipo-NedABC. 22

Supplementary Fig. 27. SSN of predicted auto-inducing peptides (AIPs) with SPII signaling peptides. 23
Supplementary Fig. 28. *S. endophyticum* BGC contains a secreted prolyl oligopeptidase. 24
Supplementary Fig. 29. Structural comparison of the chryseobasin-type macrocycle to the B ring of nisin and mutacin. 25

Supplementary Tables

Supplementary Table 1. Co-occurring protein families with ChrH-like MNIOs. 26
Supplementary Table 2. Accession IDs (NCBI) and sequences of ChrH-subfamily of MNIOs used in the SSN in Fig. 1a. 27
Supplementary Table 3. Calculated $[M+H]^+$ masses for peptides characterized in this study. 44
Supplementary Table 4. Assignment of proton and carbon chemical shifts of LysC-digested DybAH in 90% D_2O and 10% H_2O. 46
Supplementary Table 5. Assignment of proton and carbon chemical shifts of LysC-digested, IAA-alkylated DybAH in DMSO-d6. 46
Supplementary Table 6. Protein sequences of predicted MNIO-modified RiPP-lipoproteins. Sequences are grouped by cluster as shown in the SSN in Fig. 6c. 47
Supplementary Table 7. Protein sequences of predicted autoinducing peptide-lipoproteins. 58
Supplementary Table 8. Sequences and accession IDs of proteins characterized in this study. 65
Supplementary Table 9. Primers and gblocks used in this study. 70
Supplementary Table 10. Strains and plasmids used in this study. 75

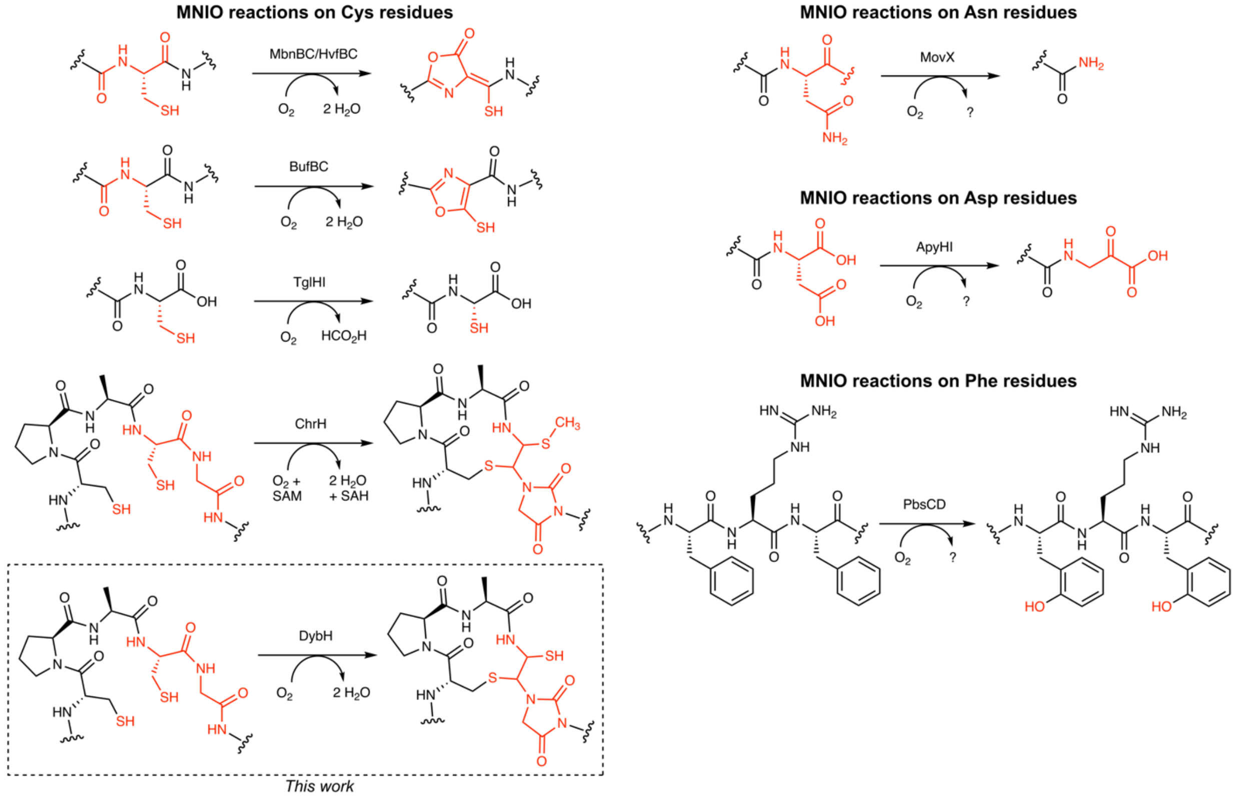

Supplementary Fig. 1. Characterized reactions catalyzed by multinuclear non-heme iron dependent oxidative enzymes (MNIOs). Currently characterized MNIOs act on cysteine, asparagine, aspartate, and phenylalanine residues within conserved motifs in a peptide or protein sequence[1-8].

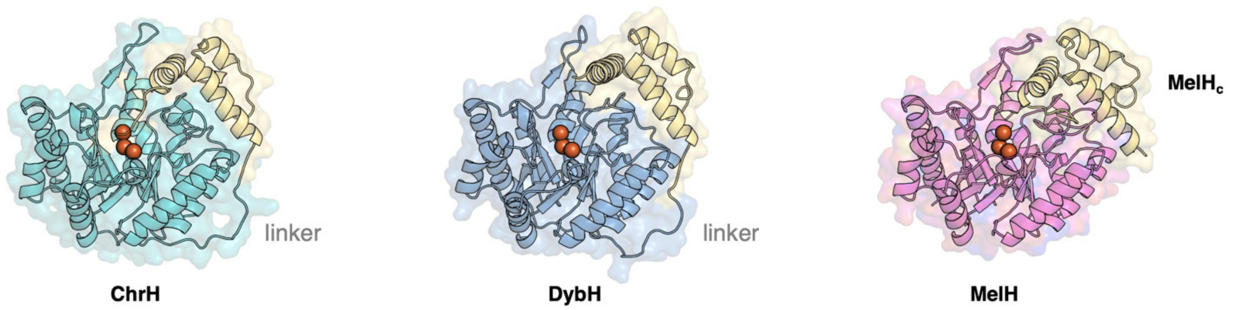

Supplementary Fig. 2. Alphafold3 models of ChrH, DybH and MelH with MelH$_c$. Predicted structures showing conservation of the core MNIO TIM-barrel in addition to the C-terminal domain (yellow). In MelH, the C-terminal domain is encoded as a separate polypeptide (MelH$_c$). All proteins were modelled with three Fe^{3+} ligands.

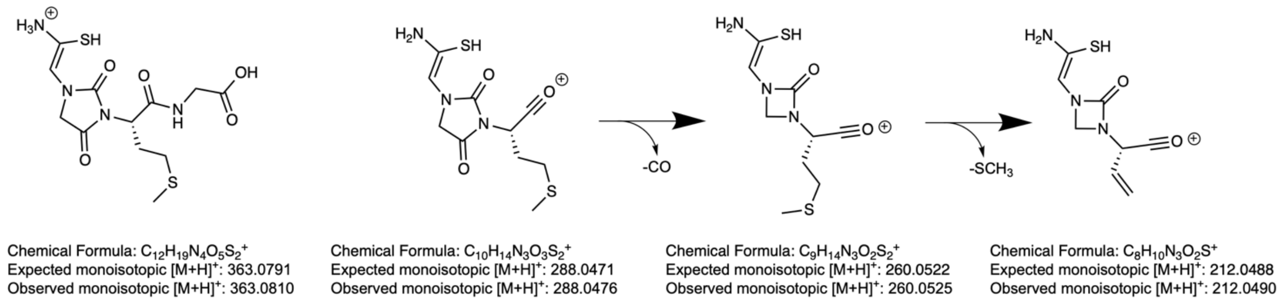

Supplementary Fig. 3. Assignment of fragmentation ions from modified DybAH from LC-MS/MS. Potential modified y-ions observed at 30 eV collision energy of LysC-digested DybAH (Figure 2b, main text). The exact mass and observed monoisotopic masses are shown below the structures.

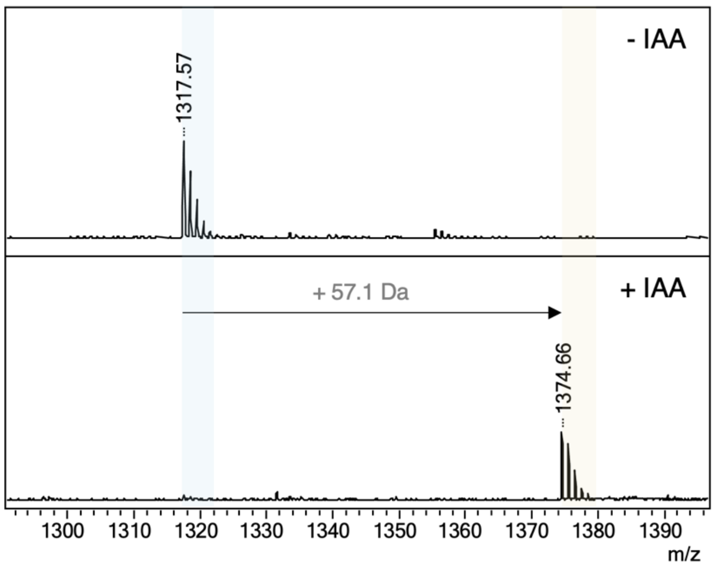

Supplementary Fig. 4. IAA-alkylation of LysC-digested DybAH. MALDI-TOF MS data of LysC-digested DybAH before and after alkylation with iodoacetamide (IAA), showing a mass increase of 57 Da, corresponding to one carbamidomethylene adduct, thus suggesting that one free thiol remains in the product. The C-terminal LysC fragment corresponds to a 13-mer peptide (sequence: TRTLDPCPACGMG) modified by DybH, expected monoisotopic $[M+H]^+$: 1317.54 Da. Expected monoisotopic $[M+H]^+$ of IAA-alkylated peptide: 1374.56 Da.

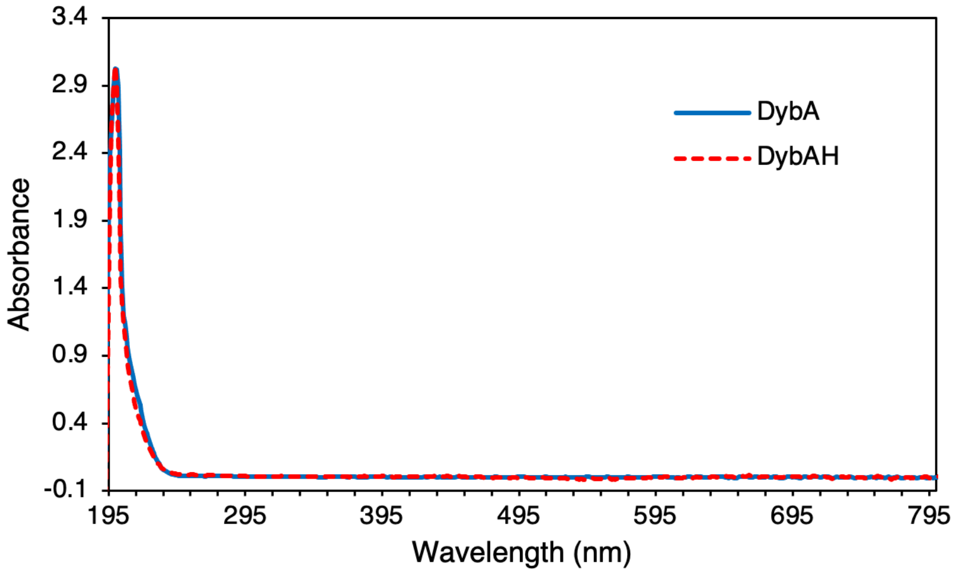

Supplementary Fig. 5. UV-Vis characterization of DybA and DybAH peptides. UV-Vis spectra of unmodified DybA, and DybAH (DybA modified by DybH) at 100 µM in H_2O, showing no change in absorbance, suggesting the lack of an aromatic feature associated with the DybH modification. DybA lacks any Trp and Tyr residues, hence the absence of a 280 nm peak.

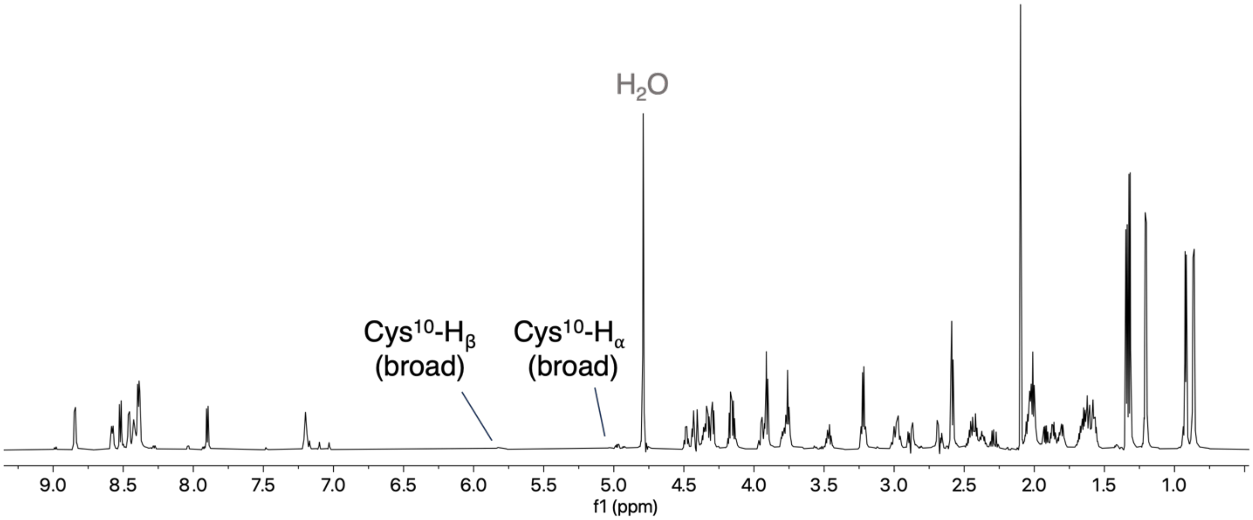

Supplementary Fig. 6. ^{1}H NMR spectrum of LysC-digested DybAH in 90% H_2O: 10% D_2O. Data collected at 25 °C on an Agilent VNMRS 750-MHz NMR spectrophotometer with water suppression.

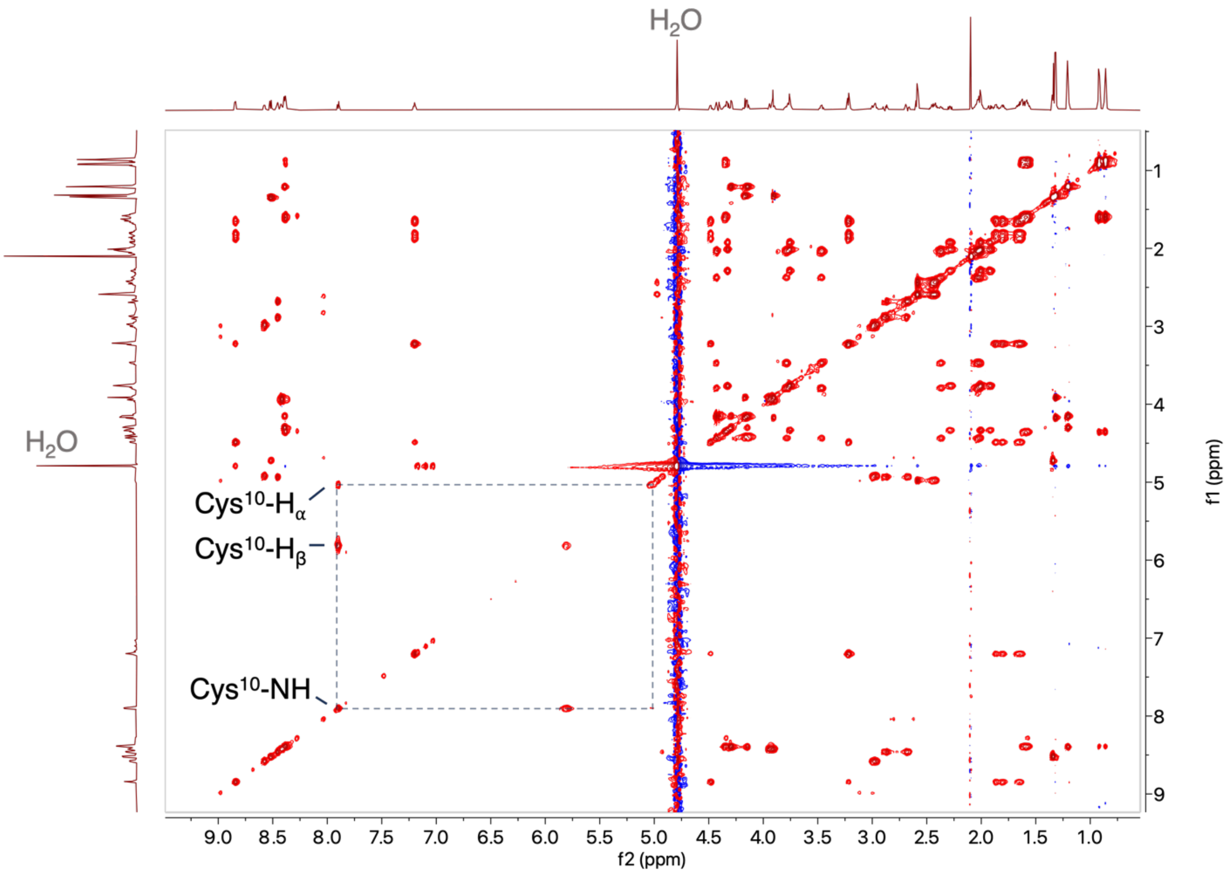

Supplementary Fig. 7. 2D ^1H-^1H TOCSY spectrum of LysC-digested DybAH in 90% H_2O:10% D_2O. Data collected at 21 °C on a Bruker 600-MHz, 5-mm spectrophotometer equipped with a Prodigy Probe. The signals of the Hα and Hβ of the former Cys10 that are shifted downfield compared to a normal Cys residue are indicated.

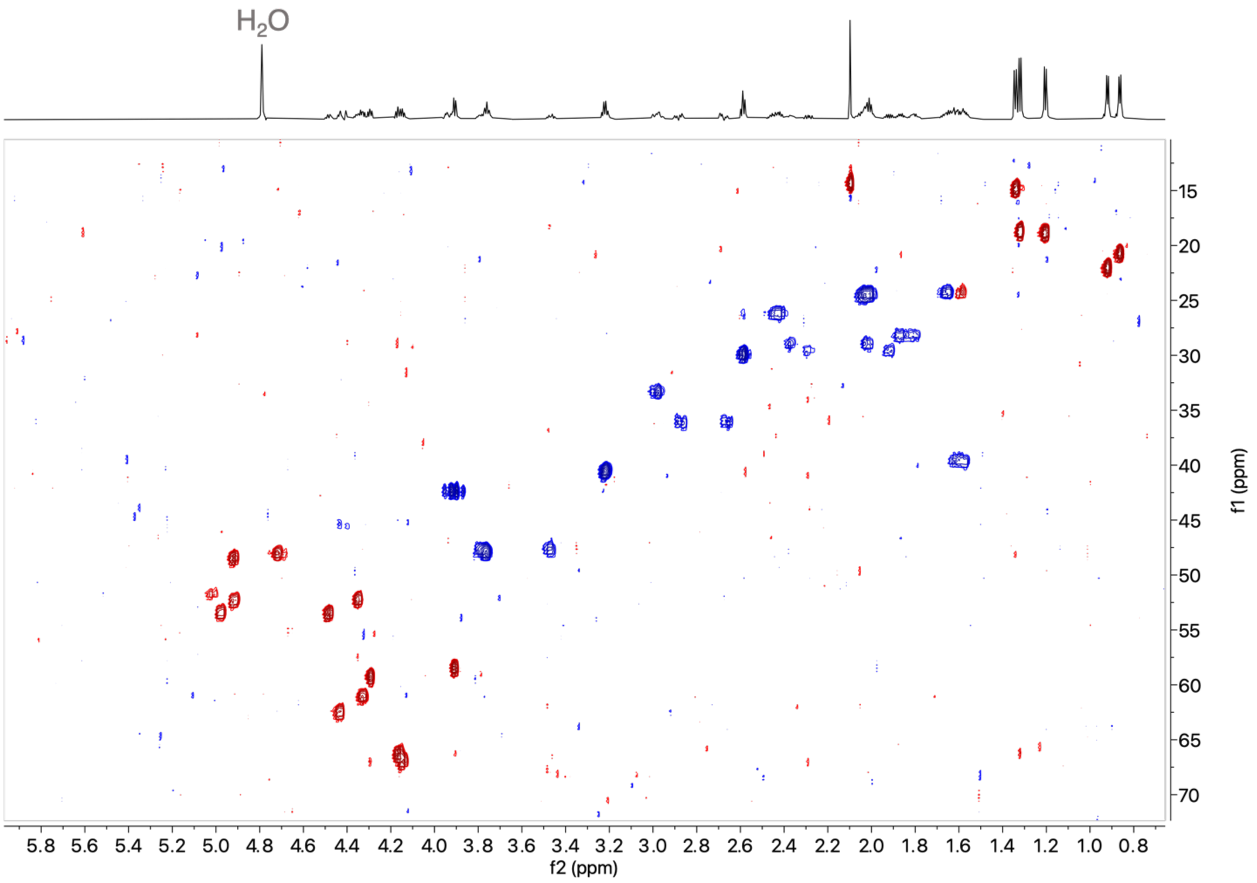

Supplementary Fig. 8. 2D ^1H-^{13}C HSQC spectrum of LysC-digested DybAH in D_2O. Data collected at 21 °C on a Bruker 600-MHz, 5-mm spectrophotometer equipped with a Prodigy Probe.

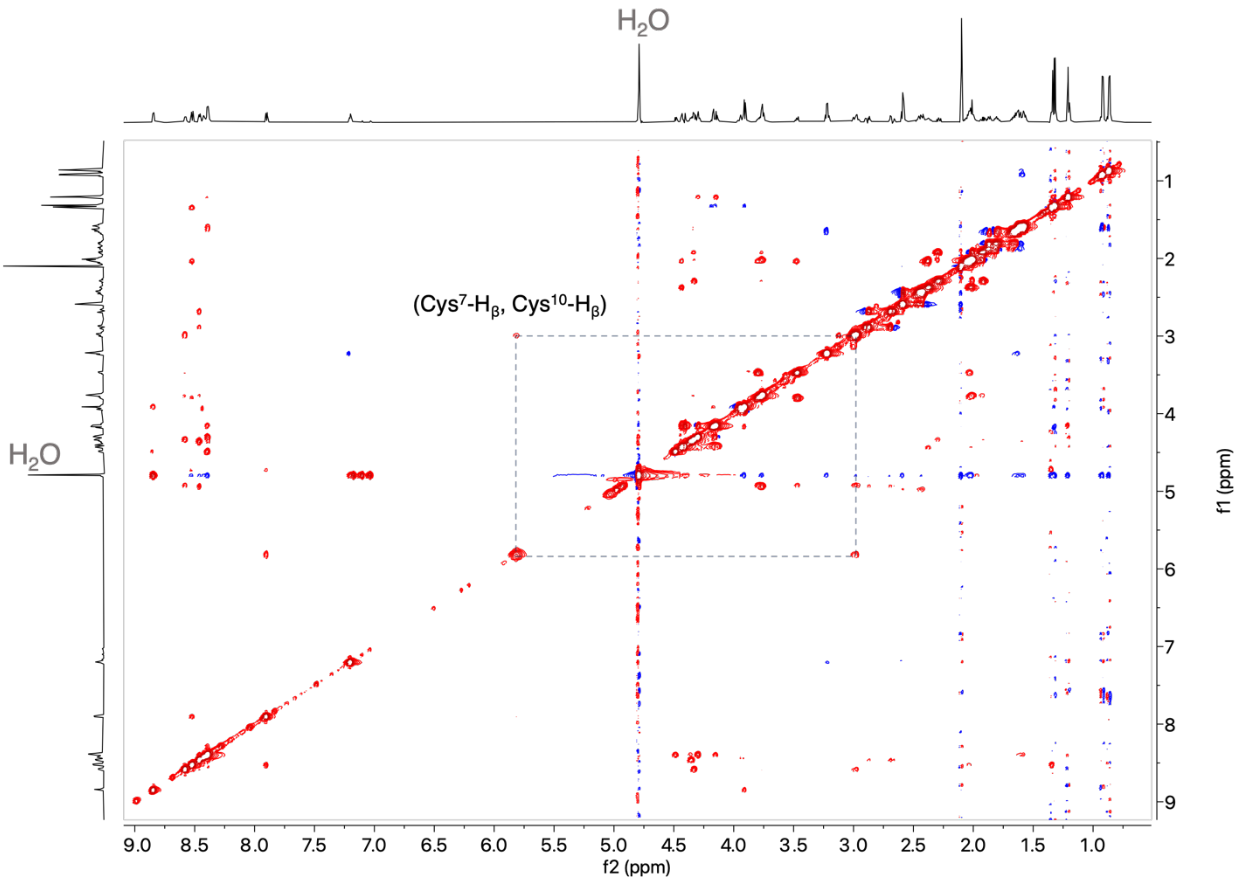

Supplementary Fig. 9. 2D ^1H-^1H NOESY spectrum of LysC-digested DybAH in in 90% H_2O: 10% D_2O. Data collected at 25 °C on an Agilent VNMRS 750-MHz NMR spectrophotometer with water suppression. The NOE between the Hβ of the former Cys10 (5.81 ppm) and the Hβ of Cys7 (2.98 ppm) is indicated.

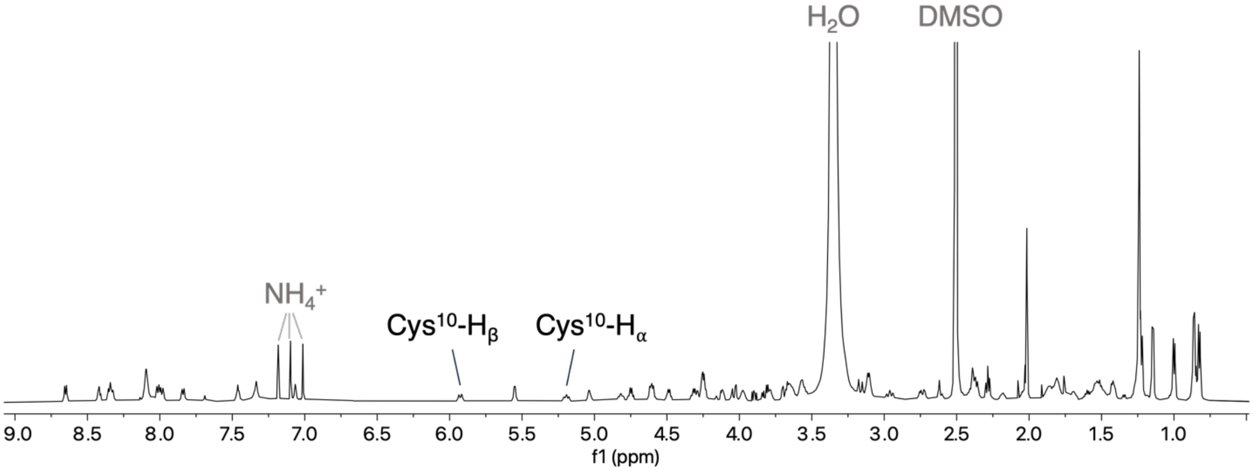

Supplementary Fig. 10. ^1H NMR spectrum of IAA-alkylated, LysC-digested DybAH in DMSO-d6. Data collected at 21 °C on a Bruker 600-MHz, 5-mm spectrophotometer equipped with a Prodigy Probe.

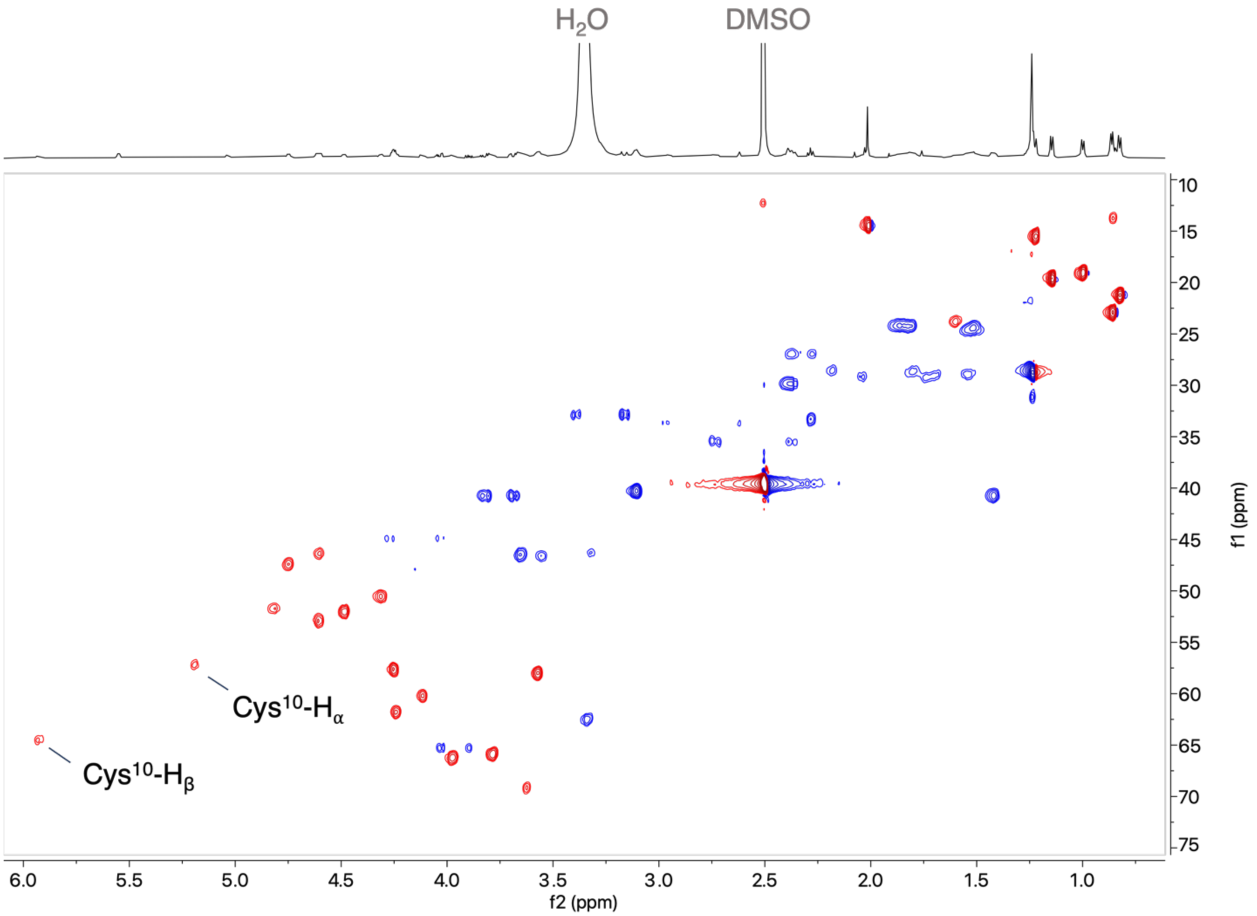

Supplementary Fig. 11. 2D ^1H-^{13}C HSQC spectrum of IAA-alkylated, LysC-digested DybAH in DMSO-d6. Data collected at 21 °C on a Bruker 600-MHz, 5-mm spectrophotometer equipped with a Prodigy Probe. Signals in red come from CH/CH_3 protons and signals in blue come from CH_2 groups.

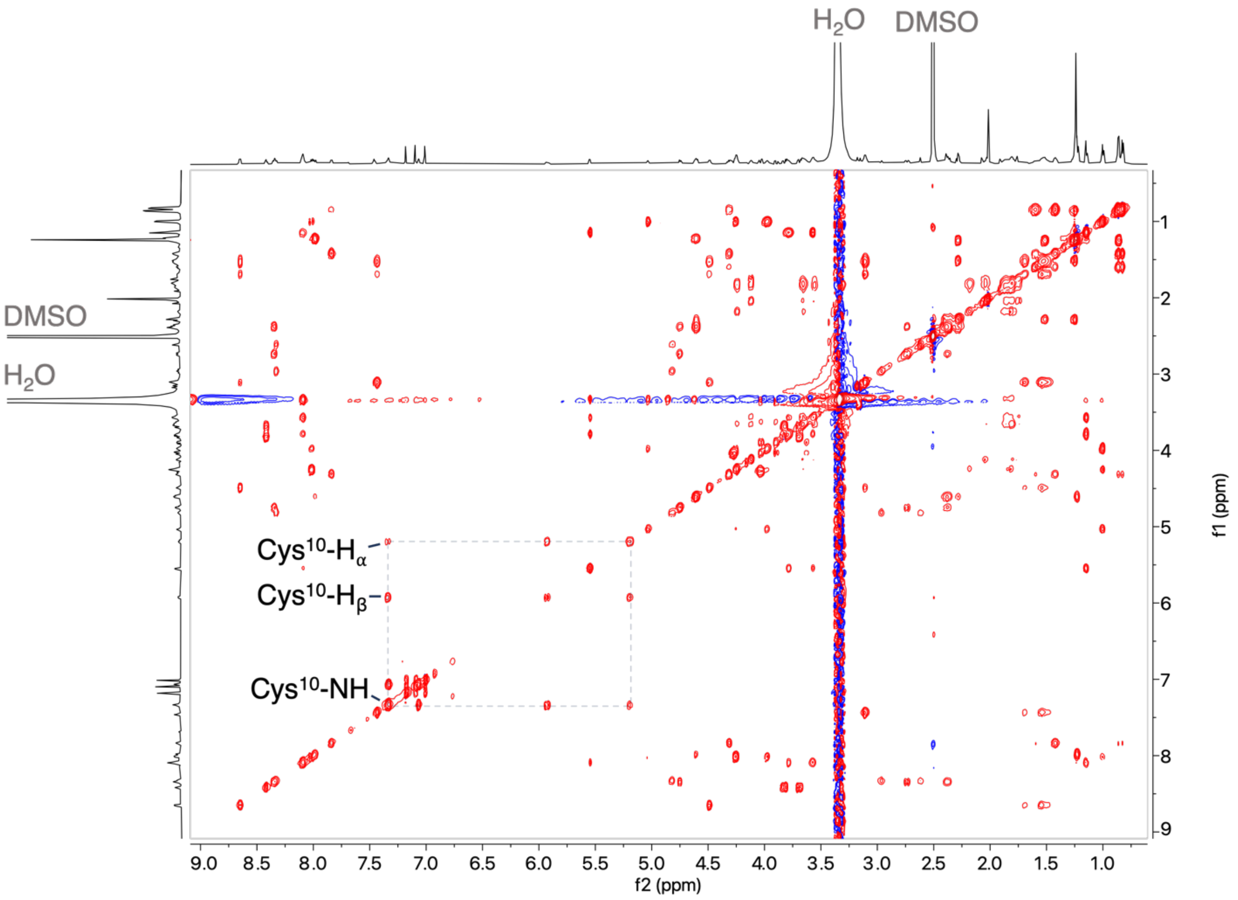

Supplementary Fig. 12. 2D ^{1}H-^{1}H TOCSY spectrum of IAA-alkylated, LysC-digested DybAH in DMSO-d6. Data collected at 21 °C on a Bruker 600-MHz, 5-mm spectrophotometer equipped with a Prodigy Probe.

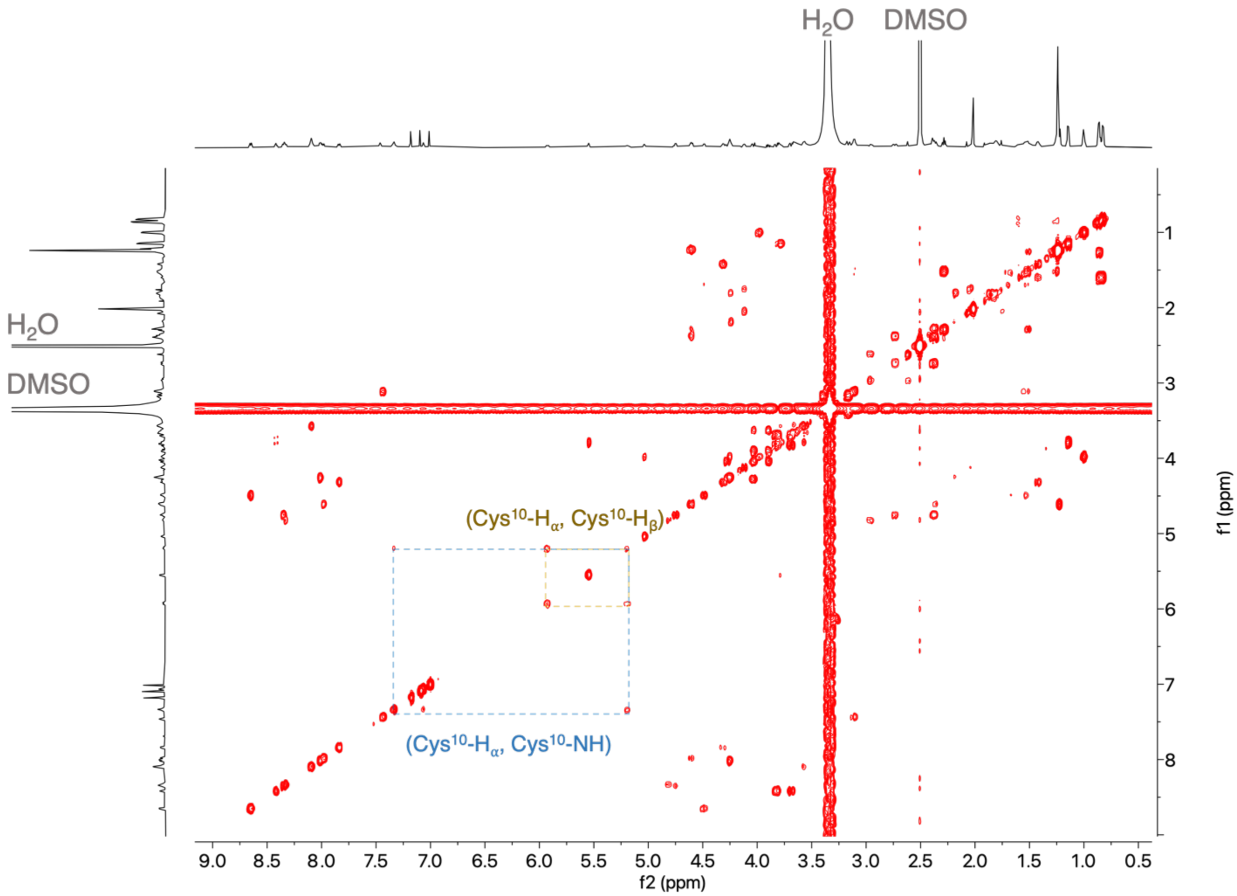

Supplementary Fig. 13. 2D ^{1}H-^{1}H COSY spectrum of IAA-alkylated, LysC-digested DybAH in DMSO-d6. Cross peaks observed between the Cys10-NH with the Cys10-Cα confirm the assignment of the α- and β-carbons. Data collected at 21 °C on a Bruker 600-MHz, 5-mm spectrophotometer equipped with a Prodigy Probe.

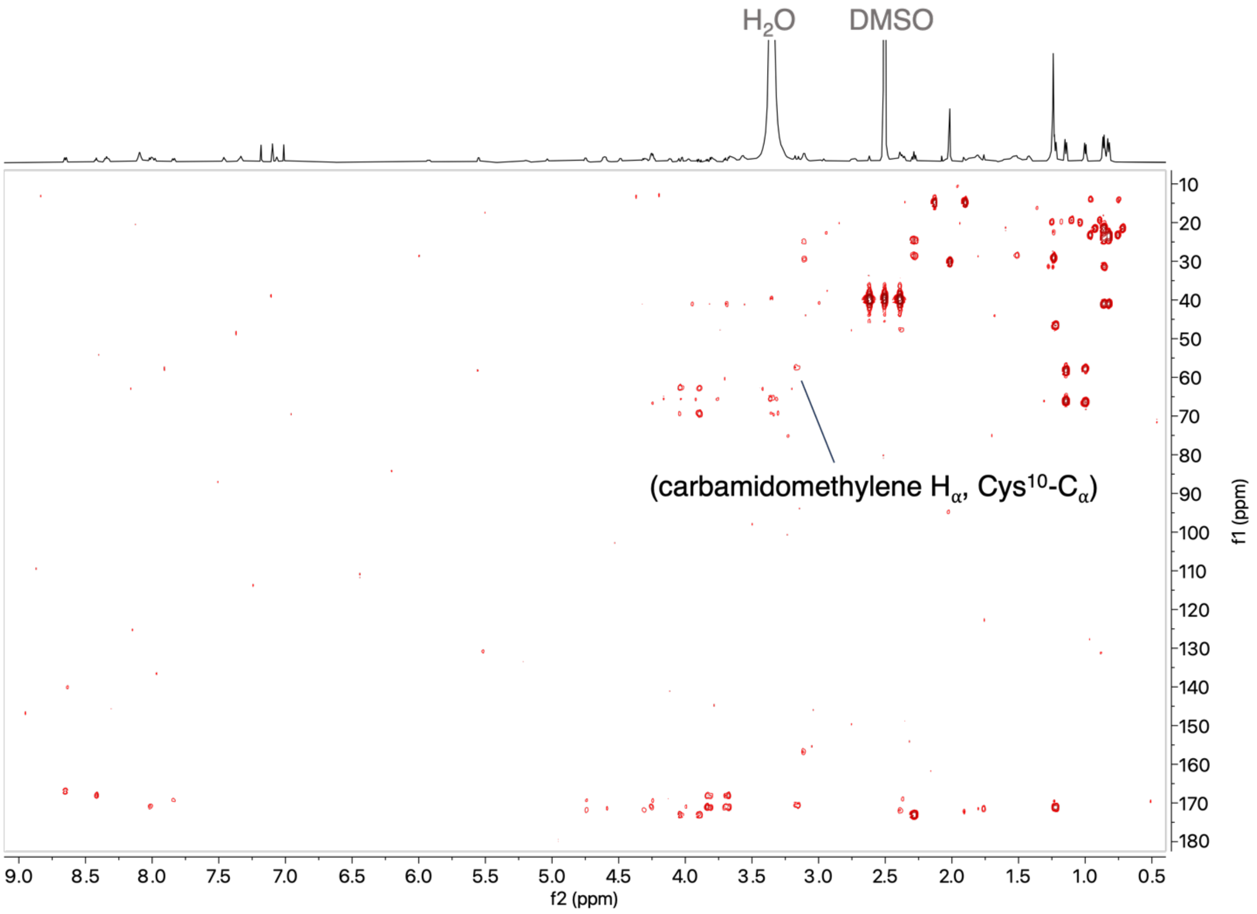

Supplementary Fig. 14. 2D ^{1}H-^{13}C HMBC spectrum of IAA-alkylated, LysC-digested DybAH in DMSO-d6. Data collected at 21 °C on a Bruker 600-MHz, 5-mm spectrophotometer equipped with a Prodigy Probe. The cross-peak between the carbamidomethylene protons originating from IAA and the Cα of the former Cys10 is indicated.

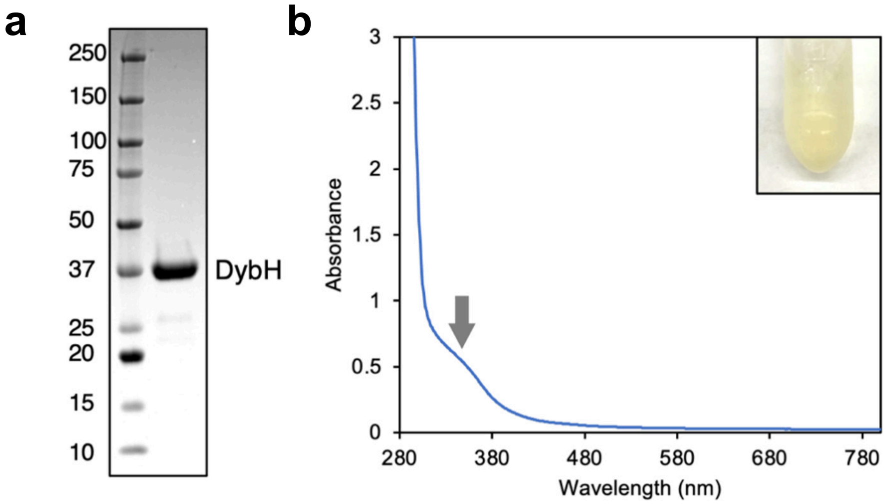

Supplementary Fig. 15. Heterologous expression and UV-Vis characterization of DybH. (a) His-TEV-DybH following IMAC purification and desalting. The molecular masses of the proteins in the MW ladder (Bio-Rad Precision Plus Protein™ Kaleidoscope™ Prestained Protein Standard) are shown in kDa. (b) UV-Vis spectrum of aerobically isolated DybH, with the arrow showing an absorbance feature at approximately 350 nm. Inset: DybH enzyme at 100 μM concentration.

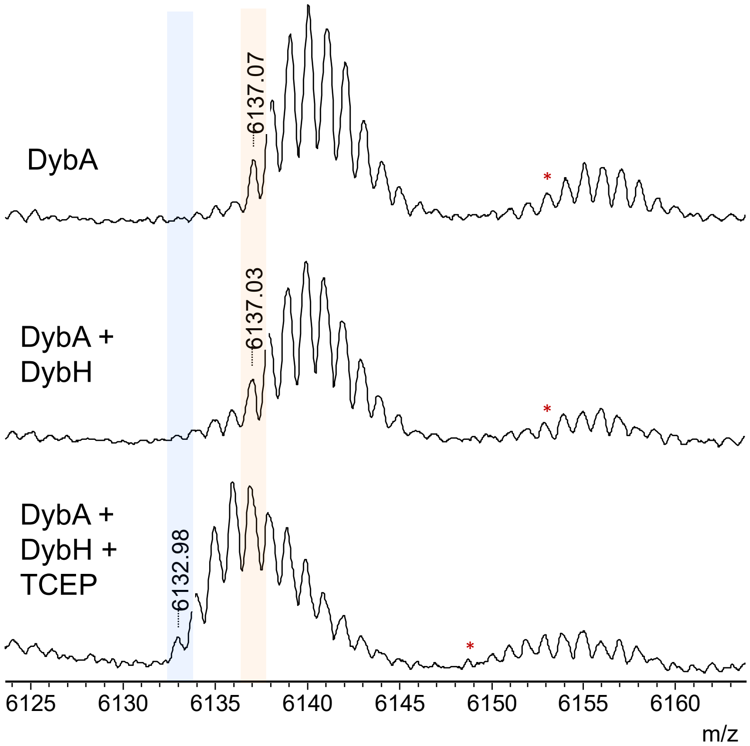

Supplementary Fig. 16. In vitro activity assay of DybH. MALDI-TOF MS spectra of DybA alone, DybA with DybH, and DybA with DybH and TCEP. The assay concentrations were as follows: DybA (75 µM), DybH (15 µM), and TCEP (1 mM) to keep the DybA thiols reduced. The reaction buffer was 25 mM HEPES, 200 mM NaCl, pH 7.6, and the reaction was incubated at room temperature for 1 h before quenching the reaction to a final concentration of 10% acetonitrile and 0.2% trifluoroacetic acid. The samples were desalted with a C18 Ziptip prior to mass spectrometry analyses. Red asterisks correspond to DybA peptides with an oxidized methionine residue.

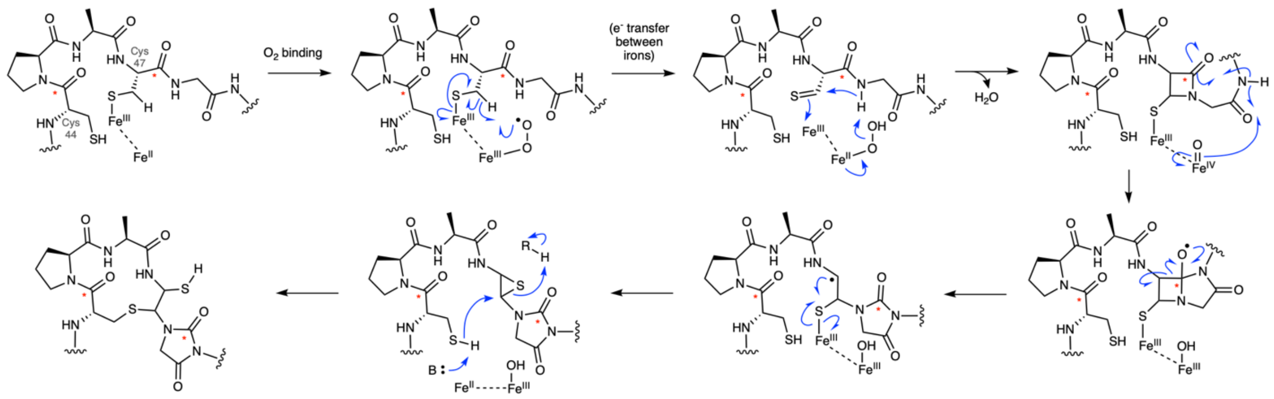

Supplementary Fig. 17. Proposed mechanism for DybH catalysis. Following substrate binding of DybA, the reaction is proposed to initiate via oxygen activation, and H-atom abstraction at the Cβ of Cys44, followed by a series of rearrangements. The mechanism is similar to that proposed for ChrH, with the exception of the final step, in which the ring opening of the episulfide results in addition of a proton instead of a methyl group from SAM (as in ChrH). Red asterisks show ^{13}C labelling studies done in ChrA[3].

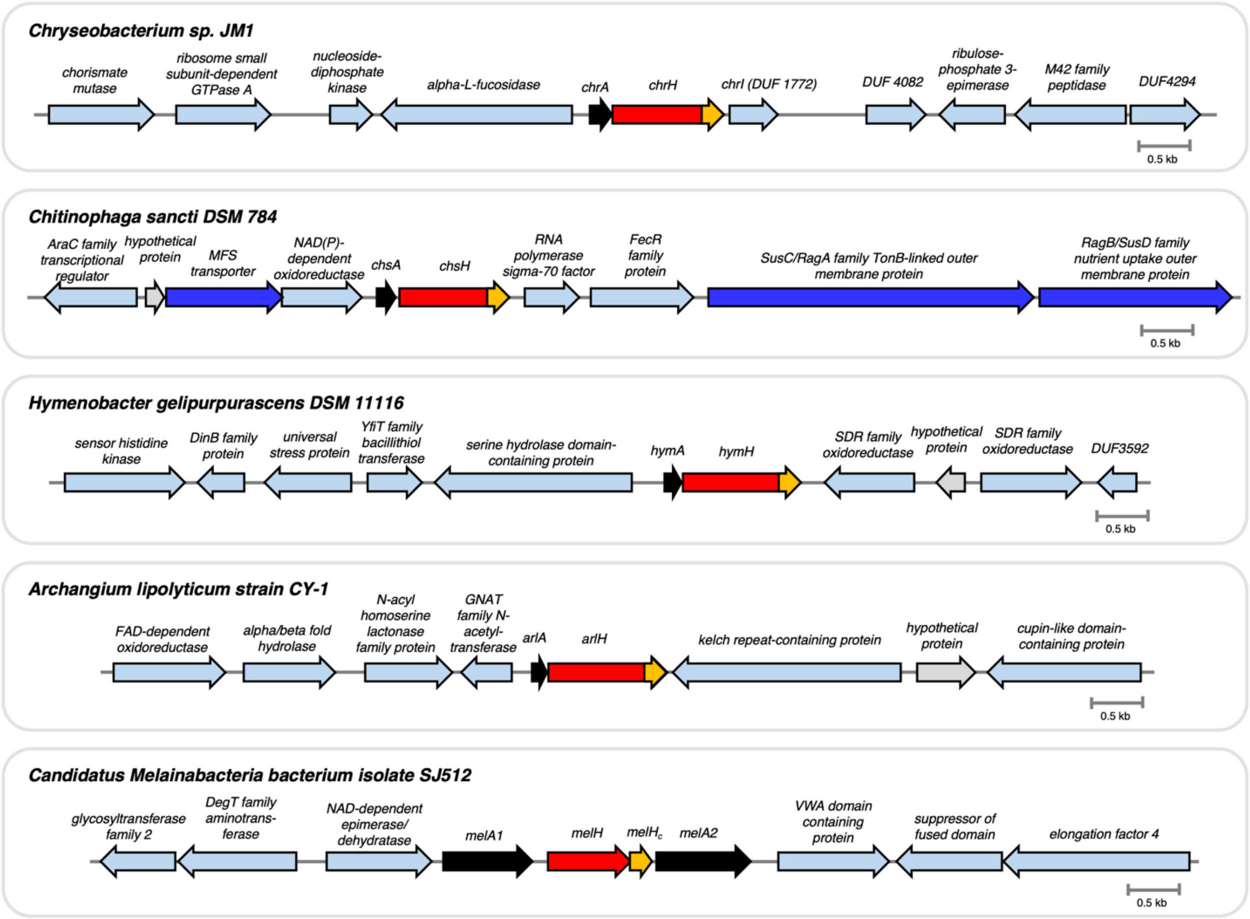

Precursor sequences:

chrA: MKIPALVMASLLAVSVSGQTTKPVKKGTKSVKKVKKMDSPKTVKAEPTKVVKRDTILKHGGGCPACGMG
chsA: MKLSKSLLSAIMIGIAVQTTVVSCSKDEQVKPKKADQANKQSESKPVDNPDSCPACGMG
hymA: MKLSQAILGAVLVGLTAQTTGCIKKSDPTPKEEQGKSGKKSPEIPYNCPGCGLG
arlA: MNQNLREKATQLLDVASNTQGMKSSSPLPCPACGMMMALPESSPQLVTPATR
melA1:
MKNKSAKVLTSLFALGLTTGAVPARADKAAVETAEASVKMFEQRSGTNNSSYTSLLMYLAGVYLANDREKDAEIIY
NKAIFNLKSKPDRKAEVPALMLNWAMLLASNKGASKEKAEKALSDGLKLANDLPAASKERINYLIGTINFYNVIGKPA
EKQARIKAADEHLATLEKNEKLNNEEITNVAANLVKLAEIQTFPMPVMRLRYQPPVFQVVPDNSPDKPNTVRAKEFK
SAEAFQLRAIKQYDRLPETVPWRIEAHRKLILWYRSLGQTKQEEFQIQQLGKIMHTTDRDKLFPQPAPCPACGMG

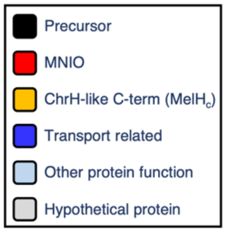

Supplementary Fig. 18. BGCs and sequences of ChrA, ChsA, HymA, ArlA, MelA1. Sequences of the precursor proteins/peptides are shown below.

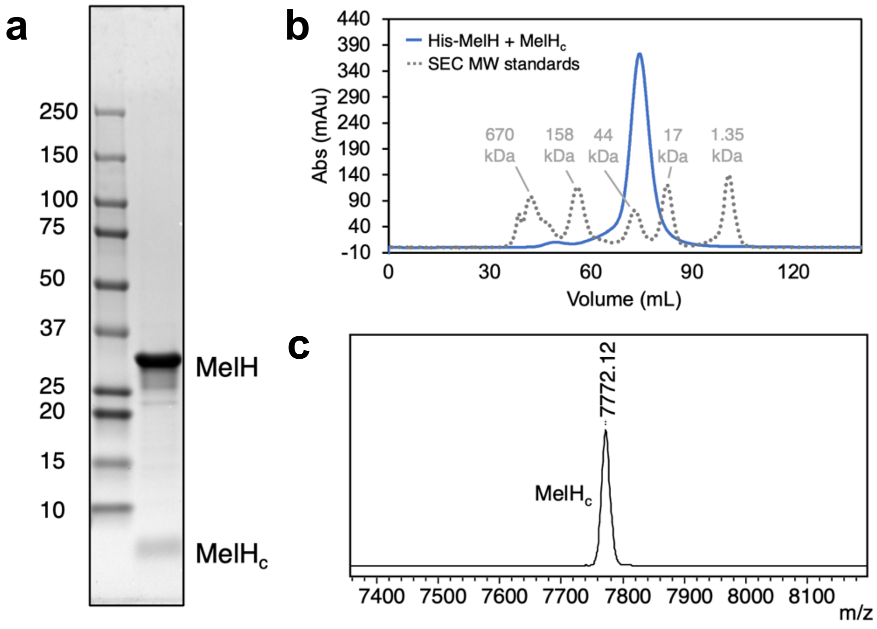

Supplementary Fig. 19. MelH and MelH$_c$ form a stable complex. (a) IMAC elution of His-TEV-MelH coexpressed with untagged MelH$_c$. (b) Size exclusion chromatography of and Bio-Rad SEC MW standards (dotted line). (c) MALDI-TOF MS spectrum (LP mode) of post-SEC purified MelH, showing that the additional gel band corresponds to MelH$_c$ (7761.60 Da expected).

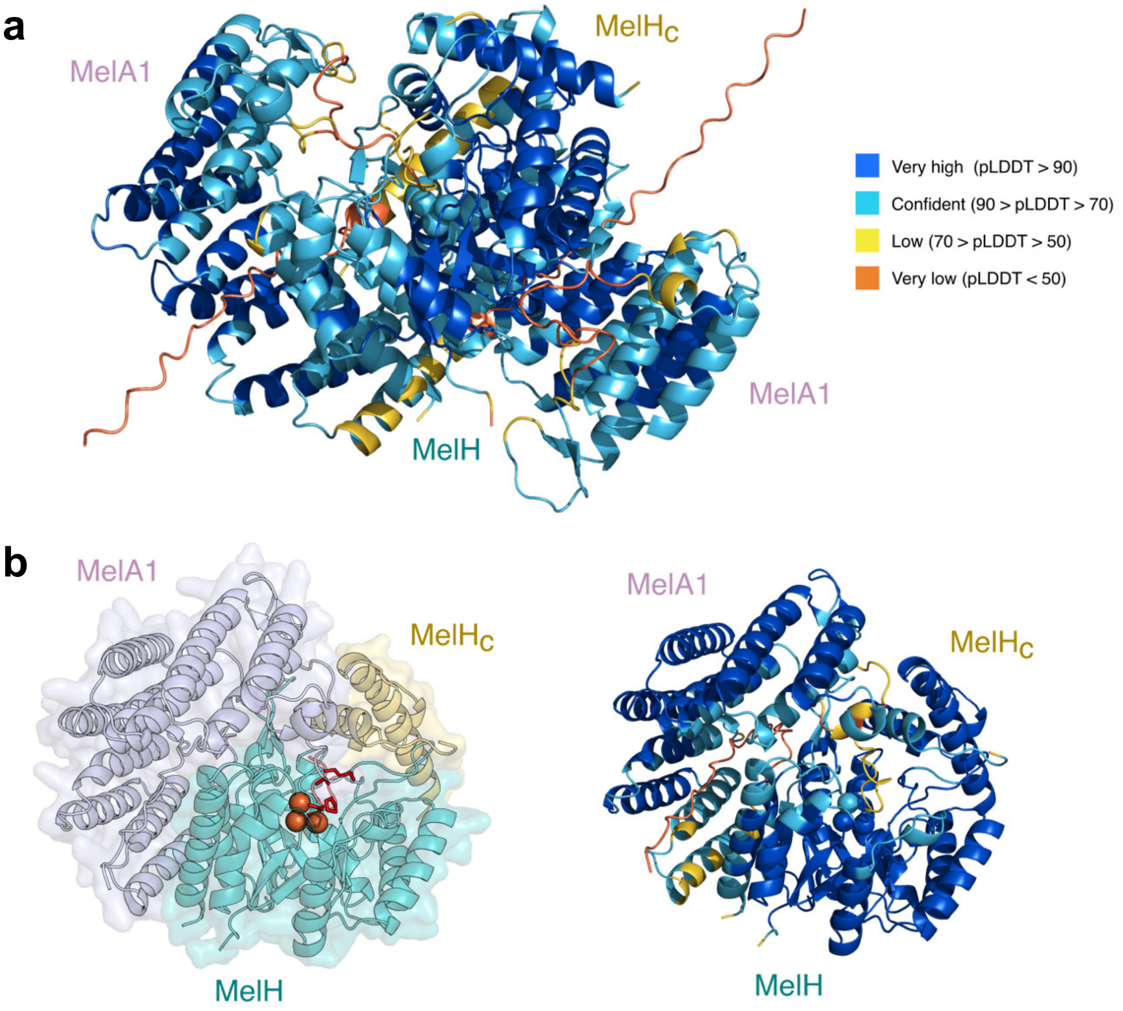

Supplementary Fig. 20. Structural prediction of the MelA1, MelH and MelH$_C$ complex. (a) Alphafold3 predicted model of the MelA1 dimer complexed with MelH and MelH$_C$ in Fig. 3 of the main text, colored by predicted local distance difference test (pLDDT) values. Three Fe^{3+} ions were modelled for the MelH cofactor. (b) Alphafold3 predicted model of the MelA1 monomer complexed with MelH and MelH$_C$. Right: the structure colored by pLDDT values.

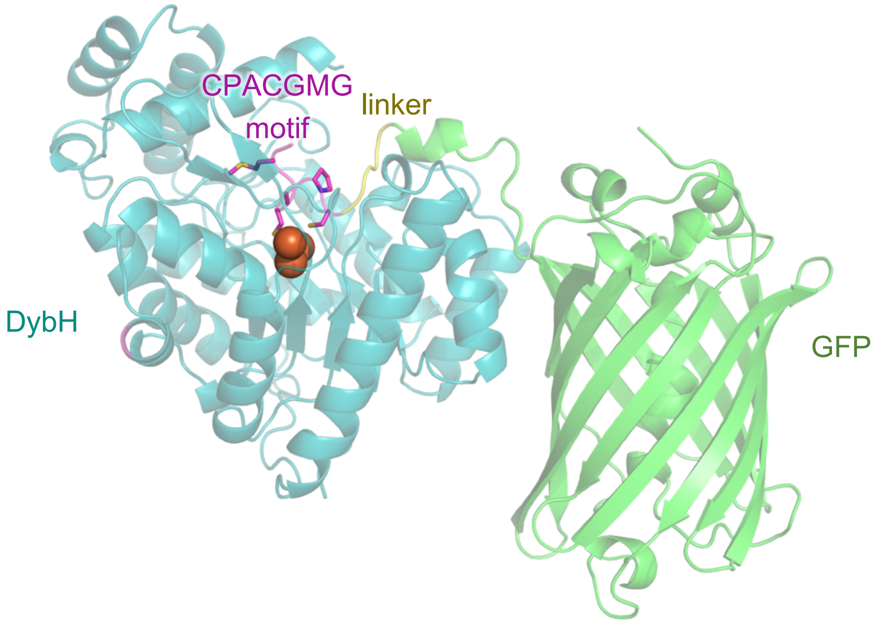

Supplementary Fig. 21. Structural prediction of GFP-CPACGMG complexed with DybH. Alphafold 3 was used tomodel the engineered GFP with a C-terminally appended CPACGMG motif with DybH. Three Fe^{3+} ions were also modelled (for DybH). GFP is shown in green, the linker is shown in yellow, and the CPACGMG motif is shown in magenta with side chains as sticks. DybH is shown in teal.

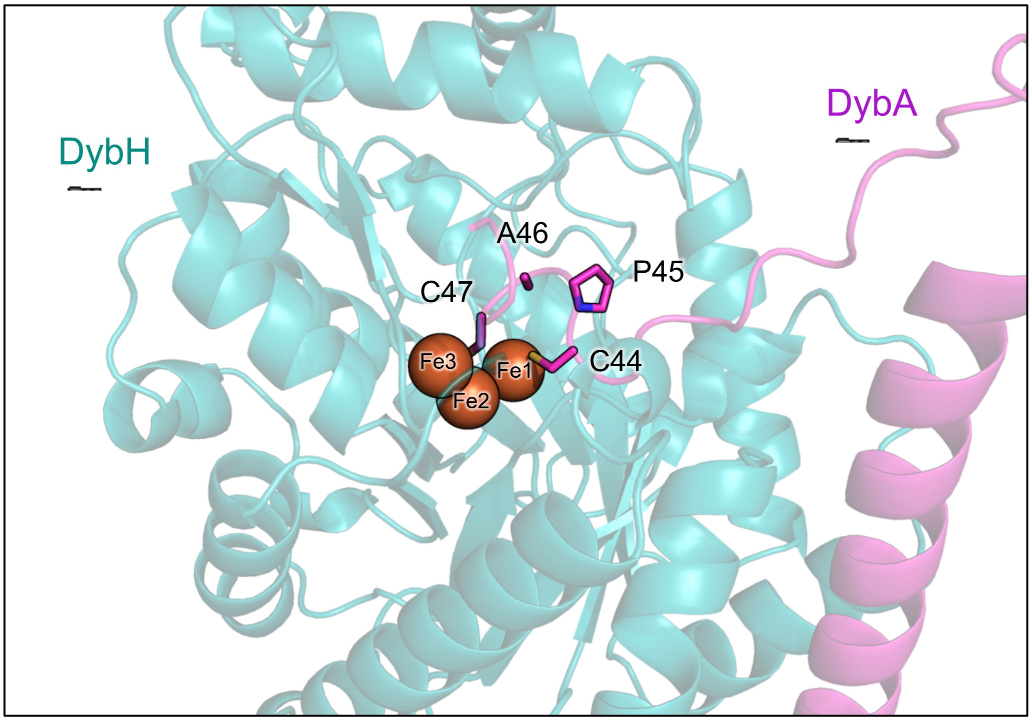

Supplementary Fig. 22. Alphafold3 model of DybA with DybH. DybA (magenta) is modelled with DybH (teal) containing three irons shown as orange spheres. The side chains of residues 44-47, ‘CPAC’ (in the CPACGMG motif of DybA), are shown as sticks. Notably, the model predicts that the thiols are coordinated to the irons while Pro45 and Ala46 point away from the active site.

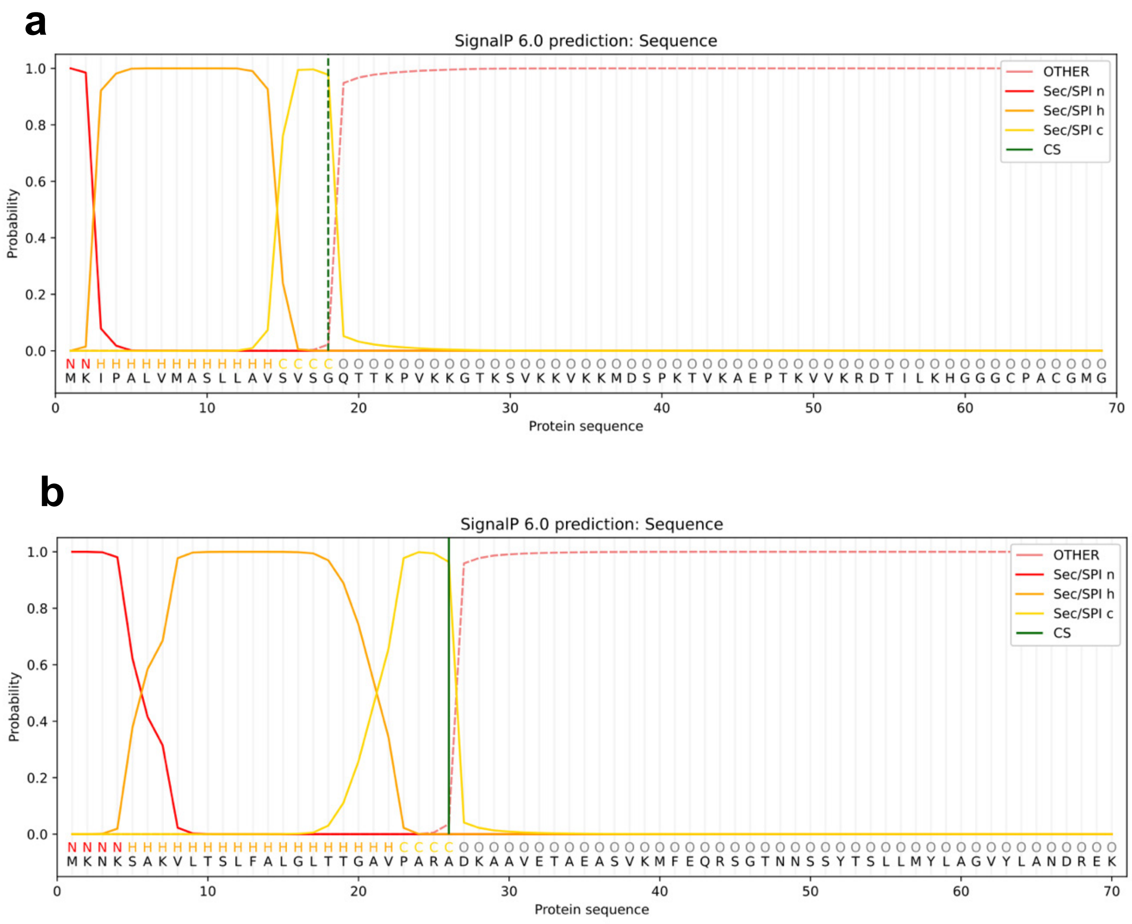

Supplementary Fig. 23. SignalP analysis of secreted peptides in the ChrA-like family. SignalP 6.0[9] graphs showing predicted signal peptidase I signal peptides for (a) ChrA and (b) MelA1. Only the first 70 amino acids are shown for MelA1.

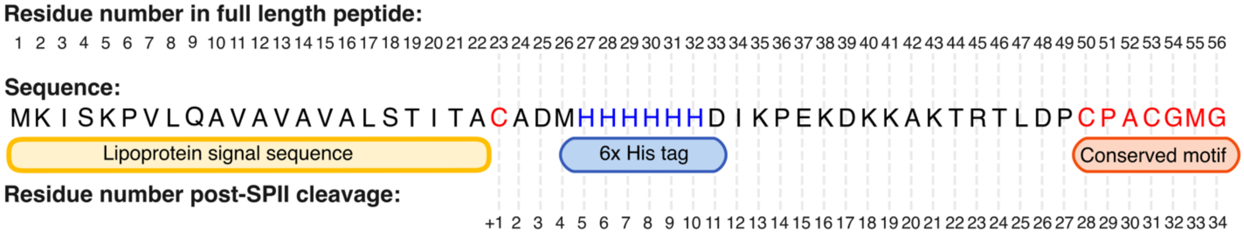

Supplementary Fig. 24. Sequence of DybAH construct with 6xHis tag at residue 27. The predicted lipoprotein signal sequence is shown in addition to the conserved CPACGMG motif. Following SPII cleavage, the His tag is at the +5 position.

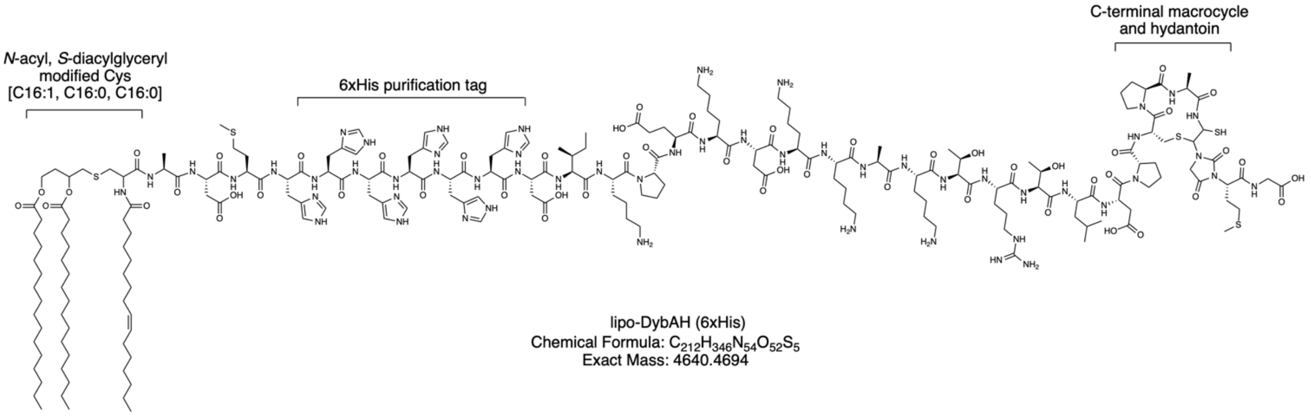

Supplementary Fig. 25. Full structure of lipo-DybAH with embedded 6xHis. The 6xHis tag is inserted within the full length DybA sequence, corresponding to the +5 position after lipid modification (Cys is +1).

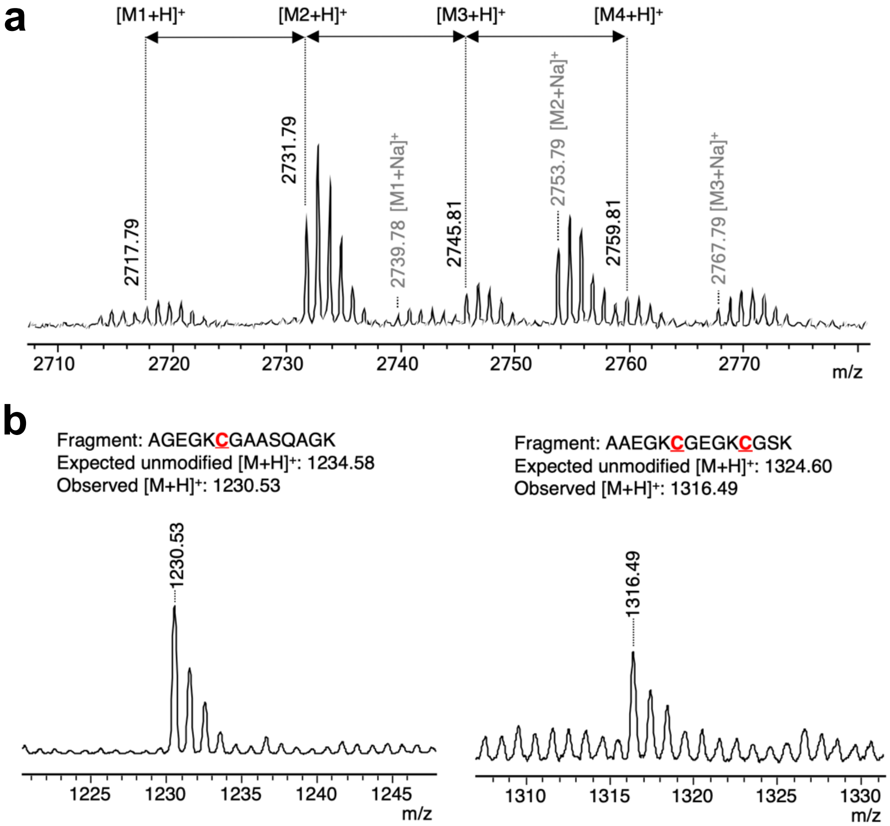

Supplementary Fig. 26. MALDI-TOF MS analysis of lipo-NedABC. (a) Isolated lipo-NedA post-LysC digest showing varying lipid lengths separated by 14 Da (CH_2) with the major lipidated species containing C16:1, C16:0, C16:0 acyl chains. (b) LysC-digest of the lipoprotein fraction showing that lipidated NedA is modified by NedBC, resulting in a -4 Da modification at each of the conserved EGKCG motifs.

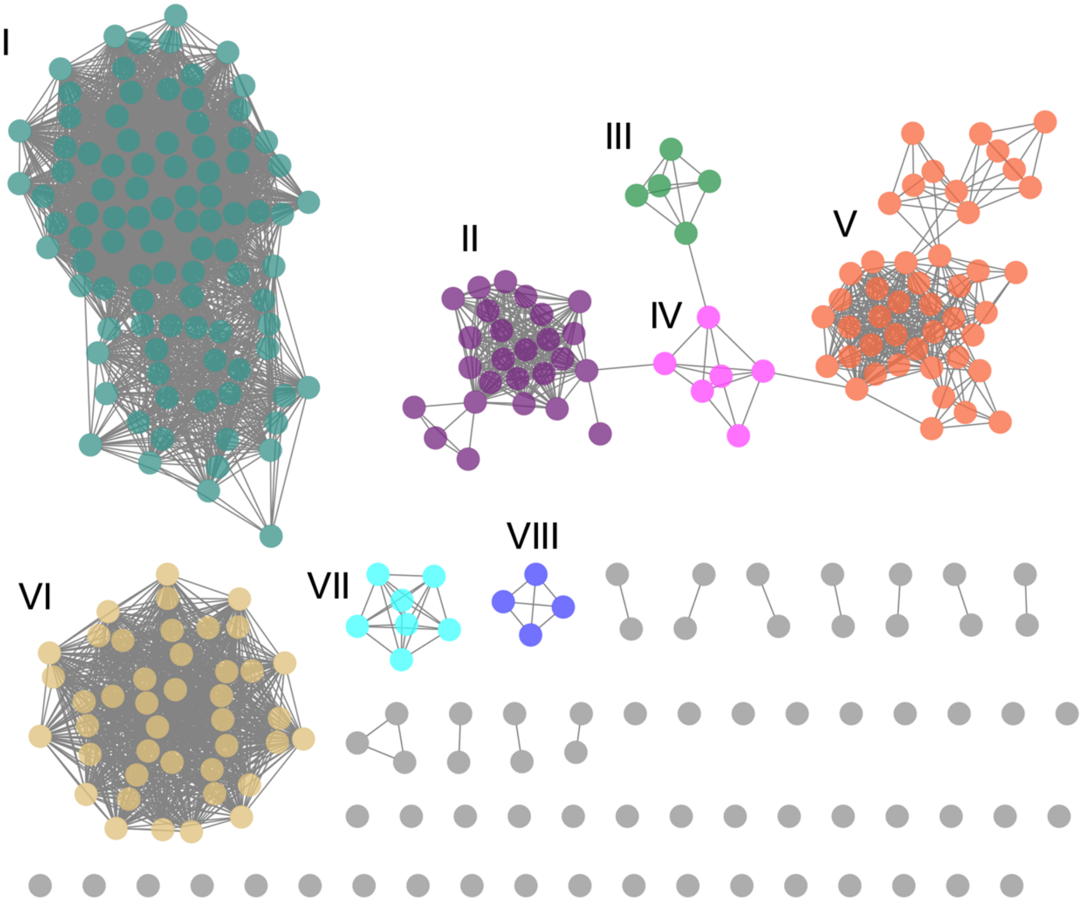

Cluster	Annotation	Representative sequence
I	AIP	MKKALAIALSAIGAVLGTVATTGCVMVLFDEPDMPKSLID
II	AIP	MKNLMKIARKTSSVLAAMALVVATSSVASCCYHWFAQPVEPEELRKFVGEN
III	AIP	MKYRILNSMVKVLTLVAFVCAASPSHSNCYEPEKPASLR
IV	AIP	MKKKILMGIAAVATVFASVISTSACYFSFYQPEEPECLREE
V	AIP	MKNCKQYFLKTFAFLLSFLAISSVNSACAVLFGQEKEPDSLARYKKC
VI	LptM chaperone	MKKVLSILLLGAFCTLATGCGVKGPLYFPEKEQPQNTQKTQ
VII	TolC-subunit	MNNMNTSKIIACGATLLLSSCGIYTSYEPQTSVPENLYGEEVTEAVSK
VIII	AIP	MKKIMYQVCTKLAACAFLAAIVSVGTASCNGLYQPKMPDKLVK

Supplementary Fig. 27. SSN of predicted auto-inducing peptides (AIPs) with SPII signaling peptides. Representative sequences from the top 8 clusters are shown below, with cysteines in red. Clusters VI/VII correspond to lipoproteins predicted not to be AIPs. The full list of sequences in the SSN can be found in Supplementary Table 7. Initial peptide sequence list of predicted AIPs obtained from Zhang *et al.* (2025)[10]. The SSN was generated by EFI-EST[11]. The Cytoscape file is provided as Dataset 3.

a

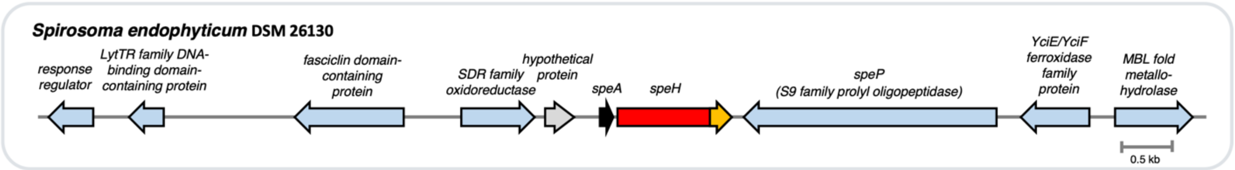

speA: MKLSKALLQTIAVAVAVTTLSSCDKGKVIDPKKDDTKQQIPYDCPGCGLG

speP:
MLHNLTTIRQSRWILLISWLAFSVASNAQDVPTYQTPPKALADLVTMPPTPTVRMSSKGDMMLIIEQPTVT
TIDELSQPELKLAGLRLNPANNGPSRVFYITGLKLKNLTDKEAKPIAGLPAKPQLSFIQWSPDQTKVAFAN
STDTQIEFYIVEVATATAKKVGSLALNATLGAPFQWLPDSKALIVKAVPAGRGPAPQLSRVPAGPTTQEN
VKGSRGQAPTFQDLLKNPVDEQQFAYYTTAQVVRMGLDGVATNIGPAGIILSAEPSPDGQYVMIETVHT
PFSYLVPVRRFPIKTEVYAVGGSLVKTLNDSPLQESVPYSRDGVATGPRDFNWRADTPASIYYTVAQDN
GDPKTKADIRDKVFLIDAPFSAQPKEIYAAQFRFETFDWGNESMALATERWWQNRKTITKTVNPTNWQT
ATLFDRSYEDRYTNPGQPDTKHNQYGREVLNLLPNNEILMVNGQGSSPEGDRPFVSLLNLKTKQTREL
WRSTAPLFERPIAVLDAAKQVILTTQETTEESPNYFVRNLKARIAPVQVTFFPHPYPQLKGIQKKQLRYKR
TDGVELTAMLYLPAGYKKEQGPLPTFLWAYPAEFKSKDAASQISGSPYQFGWNRPFWGAAAFVTMGYA
ILDNASIPIVGEGDKEPNDTYVEQLVSSAKAAIDEGVRLGVVDSSRVGVGGHSYGAFMTANLLTHSKLFK
GGIARSGAYNRTLTPFGFQNEQRSYWQAPDVYNNMSPFMNADKMKTPLLLVHGEADNNTGTFPIQSER
YYNALKGFGATTRLVFMPYESHHYDAKESLLHMLWEMNGWLDKYVKNPTAAGQANKAGKLGSGK

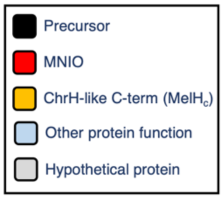

b

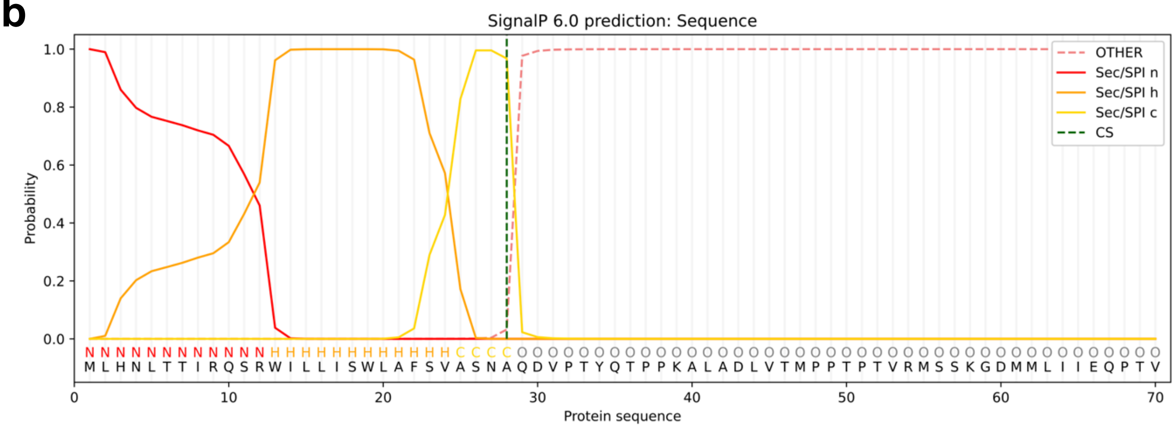

Supplementary Fig. 28. *S. endophyticum* BGC contains a secreted prolyl oligopeptidase. (a) BGC of *S. endophyticum.* SpeA contains a SPII signal peptide for lipidation. SpeH is the ChrH/DybH-type MNIO and SpeP is a S9 family prolyl oligopeptidase. (b) SignalP 6.0 analysis of SpeP shows that the first 28 amino acids constitute a SPI secretion signal.

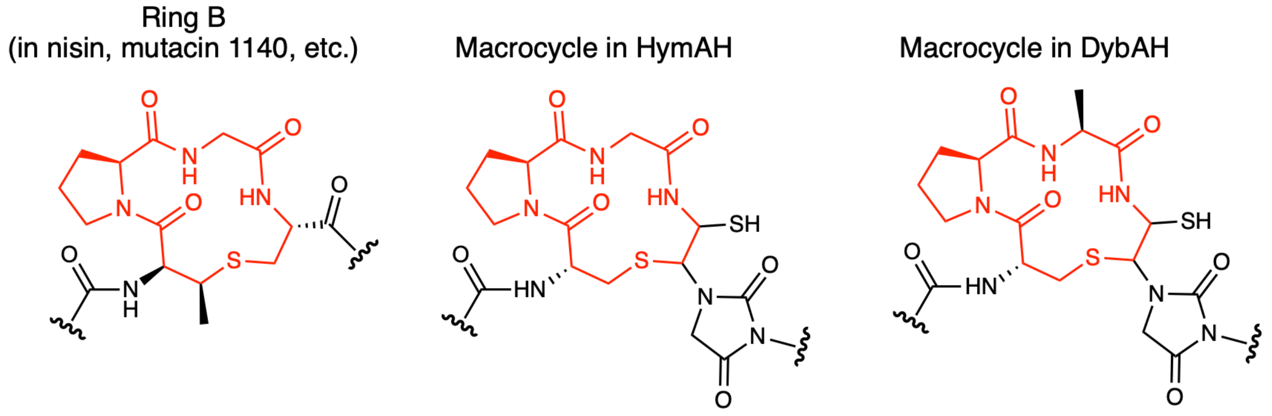

Supplementary Fig. 29. Structural comparison of the chryseobasin-type macrocycle to the B ring of nisin and mutacin. Structural similarities between the modified HymA and DybA macrocycle and the lantibiotics nisin and mutacin 1140 are shown in red bonds.

Supplementary Table 1. Co-occurring protein families with ChrH-like MNIOs. The EFI-Genome Neighborhood Network Tool (GNN) was used to analyze frequency of co-occurrence (10 genes upstream and downstream) of the ChrH-like MNIOs. The top 10 co-occurring PFAM domains with corresponding SwissProt annotations are shown. Frequencies greater than 1 indicate multiple copies of the same PFAM within the gene neighborhood window.

**ChrH-type BGCs**		
**Frequency**	**PFAM**	**Description**
1.52	PF00254	FKBP-type peptidyl-prolyl cis-trans isomerase
0.95	PF00834	Ribulose-phosphate 3 epimerase family
0.95	PF05343	M42 glutamyl aminopeptidase
0.93	PF00440	Bacterial regulatory proteins, tetR family
0.93	PF00293	NUDIX domain
0.93	PF00334	Nucleoside diphosphate kinase
0.93	PF14127	Domain of unknown function (DUF4294)
0.92	PF00793	DAHP synthetase I family
0.92	PF01063	Amino-transferase class IV
0.92	PF01817	Chorismate mutase type II
**DybH-type BGCs**		
**Frequency**	**PFAM**	**Description**
0.41	PF07715	TonB-dependent Receptor Plug Domain
0.38	PF13715	CarboxypepD_reg-like domain
0.3	PF00072	Response regulator receiver domain
0.26	PF00593	TonB dependent receptor-like, beta-barrel
0.2	PF08281	Sigma-70, region 4
0.2	PF12833	Helix-turn-helix domain
0.19	PF04542	Sigma-70 region 2
0.18	PF07980	SusD family
0.18	PF14322	Starch-binding associating with outer membrane
0.16	PF02518	Histidine kinase-, DNA gyrase B-, and HSP90-like ATPase
**MelH-type BGCs**		
**Frequency**	**PFAM**	**Description**
0.42	PF02574	Homocysteine S-methyltransferase
0.28	PF01370	NAD dependent epimerase/dehydratase family
0.28	PF00535	Glycosyl transferase family 2
0.28	PF02321	Outer membrane efflux protein
0.28	PF01041	DegT/DnrJ/EryC1/StrS aminotransferase family
0.28	PF02219	Methylenetetrahydrofolate reductase
0.28	PF05076	Suppressor of fused protein (SUFU)
0.28	PF00009	Elongation factor Tu GTP binding domain
0.28	PF00679	Elongation factor G C-terminus
0.28	PF03144	Elongation factor Tu domain 2

Supplementary Table 2. Accession IDs (NCBI) and sequences of ChrH-subfamily of MNIOs used in the SSN in Fig. 1a.

NCBI ID	Organism	Sequence
ACU59980.1	Chitinophaga pinensis DSM 2588 DSM 2588	MEESRIEAIEWSFDALYKVKEVPDWFRELLTAFSDENRLIGHGVFFSLFSGKWLPEQENWLKHLSQTADAFQFDHITEHFGFMTGKDFHQGAPLNIPYTAITLNIGRDRLKRIYEACKRPVGLENLAFSYSLDEVKRHGAFLEQLLEPVNGFIILDLHNLYCQLRNFDVTFEEMIALYPLDRVREIHISGGSWEDSVATPSRSIRRDTHDDAVPTEVFQLLEMTIPRCPQLKYVVLEQLGNGLETETSRQYFYNDFLRMQEIIQQHNDQADDTSTDPFLPLIPFSTDAVVEDMMLYQQQLELSAIFESSASYGEAMQSLSTSSLANSDWRIEQWEPHMIETAVKIAQKWMK
WP_034736621.1	Chryseobacterium indologenes	MMRKPLLGLSMMPEAEFVSAALPLLQNNSVEVLEWSFDTLFNTNEPDWLRDLLNFYAENNRLIGHGVYYSLFDAQWTDRQEIWLSRLKDEVSKRNYNHITEHFGFMNTENFHQGVPLPVPLHPITLQIGQDRLQRLQDAVNIPVGIENLAFSFCLDDVKEQGVFLEKLIDNSGGFLILDLHNMYCQSCNFDVDIQEIIKLYPLQKVKEIHLSGGSWQESVYGKKMIRRDTHDHTIPEEIFAILPFVLSHCENLEYIIIERLGHTIKTKEEEDDFFSDFMRVKTIIDSSAGHERQGDLWIKKEFELSKYPVEDPVLYEEQSRLTKLLFDGTHVDSIKNENFHYFNTAGWDPEMIFTAQEIIKKWNPY
WP_027375286.1	Chryseobacterium	MRKPLLGLSMMPEAEFVSAALPLLQNNSVEVLEWSFDTLFNTNEPDWLRDLLNFYAENDRLIGHGVYYSLFDAQWTDRQEIWLSRLKDEVSKRNYNHITEHFGFMNTENFHQGVPLPVPLHPITLQIGQDRLQRLQDAVNIPVGIENLAFSFCLDDVKEQGVFLEKLIDNSGGFLILDLHNMYCQSCNFDVDIQEIIKLYPLQKVKEIHLSGGSWQESVYGKKMIRRDTHDHTIPEEIFAILPFVLSHCENLEYIIIERLGHTIKTKEEEDDFFSDFMRVKTMIDSSAGHERQGDLWIKKEFELSKYPVEDPVLYEEQSRLTKLLFDGTHVDSIKTENFHYFNTAGWDPEMIFTAQEIIKKWNPY
WP_012927702.1	Spirosoma linguale	MSRLYSSLACNLDTNSLQASLPLFEAEKVQAIEWSFDTLFALGEMPDWFAELLRAYSQEGRLIGHGVFFSLFSGKWTPDQQQWLKQLKALSADFTFDHLTEHVGFMTGEDFHKGAPISIPFTTSTLAIGRDRLLRLQDAGNCPVGLENLAVAYSLDDVKRQGDFLAQLLEAVNGFILLDLHNLYCQSQNFDLGIADIQALYPLDRVREIHISGGSWVPSTVNPTKQIRRDTHDESVPAAVFHALQQVIGQCPNLKYVVLEQLGTGLTTDVSRQHFREDFYTMDALIEATNQLNSHSPINSFLPLSETSIPETPMENPLLNQQQTELSAILETATDYGQAQLFLNASSLANSDWNIENWQPEMLETALAIAQKWKDGLV
WP_013552756.1	Cellulophaga algicola	MGNKKPKLGIAIAPSQKFLEAALPLFAEEKIEIIEWSFDTLKDEADEPAWLSLLLKEYGEKNRLLGHGVFYALLDANWSSRQENWLKKVRQETLSHKYCQISEHFGLMSSANAHSGFPLPIPLSNPVLQIGIDRLKRLQATAQVDVGIENLALTANVADILEQGEFLFKLVNPVNGFVILDLHNIYCQSENFNIDMMTIIKSYPLSLVKELHISGGSWDTDPTLSRKIRRDTHDGRIPEVILEILPEVLKICPFLAFIIFEKIEDSFLTEKDGIDFRADFQKIREIIDATSFSVTPKEKKQVAIILGPPVIDVELLQAQVALRESIALDTYQTNSEWDKDMWKVATKLYEKWNN
WP_013687393.1	Fluviicola taffensis	MSKEPLKIQSGVSCNLDNSLLQATLPLFAAEKVEAIEWSFDTLYKVREIPEWFVELLHAFGNEGRLIGHGVYFSLFSGKWTAEQQRWLDHLRKVSSNFTFNHITEHFGFLTGEDFHKGAPISVPYTTETLNIGIDRLKRIQEACSCPVGLENLAFSYSLEEVQRQGEFLERLIEPVNGFIILDLHNLYCQLQNFSLDFETLIQAYPLDRVREIHISGGSWESSGIHPQRTIRRDTHDDAVPEAVFELLKQTLPLCRNLQFVVLEQLGVALHTLESQQQFQTDFNLMQSIVNEFNAQNQSISNADFLPKEIFQLNEQIPESVTLHQQQLELSSILEHAPNYEEATRLLSNSSLAYSAWKTEAWSPYMLETALRIAQKWKQGW
WP_010467436.1	Acaryochloris sp. CCMEE 5410	MTPQIGLSLMPQPDFWQAAQPLFDASEVEVVEWSFDMGWGGVSLPDPLCSVLRQFSQRNGLLGHGVSYSLLSAQLDRRHWLACLQAECRDYSYRHISEHFGWMATRNFARSAPLPMPMLPETLKLGCDRIQQFAEIAQVPVGLENLAFAFGLQDVRQQGEFLDQLLESVDGFLLLDLHNLYCQMHNFQLSATEILNLYPLARVRELHLSGGSWSHHGTATIRRDTHDQAVPELVFELLKLALQRCSSVEAIILERMGNTLNSEQEQIQFRQDFTHIRQITDRFTIEART
XP_004338586.1	Acanthamoeba castellanii str. Neff Neff	MEAEQAGGPWVGVSLMPHPQYRRAIQPLLDQHLIDAIEWSCDIGWGMGGRLPAWVVDLLDQFGRAGRLFGHGVMYSPTSAIWERRQAAWLRWLERECEERRFGHITEHFGFMTAGGALEGCPLPLPFTPGAVEVGVDRLRRMHRAAGGATPVGLENLALAMGPADAADQGPFLAAVLERLGDQGVLLLDLHNLHCQIVNFSLSAQDAIKRLACDPEGRPFRRDTHDGDVPPEIIDELLPLALRLCPNLRVVVLERLGETIRSEADAERLREDFCRIRSVVRDFASAQRAQSTAGAAAAADESAGGRATAEADHHNNINATTATDDDDGGGDEAALESPPFEDEELGRYQAAFLRLFVRDLSPEQLLHQLRAQPEFAPFQKYVEGFDVRCVEVASVLVRKWVRFARPKAYESRWRRPRPDSEGEEDEEEEEEEEYAEEDGEGGDGGECDVYESSEADE
WP_052346243.1	Hymenobacter swuensis	MPTPTFPLPVEHPPVLATLACNLDADILAAAFPLLEAGRVAALEWSFDTLFAVEQVPEWFMALLRSYSTQGRLIGHGVYFSLLSGRWTAEQQQWLEQLRQLSAQVAFDHVTEHFGFFTGENFHSGAPLPIPYTSSTLRLGQDRLRRVQAACGCPVGLENLAFAYSLAEVQRHGEFLAKLVAPVNGFLILDLHNVYCQLHNFSLSTESLLSAFPLELVREIHISGGSWQPSGQVPGRQIRRDTHDEAVPEEVFQLLEYALPLCPNLKFVVLEQLGNALHTEASRTRFRADFSRMETLVQQRPQRPQNPIYQSFLPGPAGPLTAAAAEDEQLHTQQRHLAHILETATSFAEARHQLRASPLAHTAWKIEDWEPHMLETAISIAQKWKQ
EFK35127.1	Chryseobacterium gleum ATCC 35910 ATCC 35910	MRKPMLGVSMMAEADFVSAILPLLQNNSIDVLEWSFDTLYHPNEPDWLRDLLNFYAENGRLIGHGVYYSLFDARWTERQDEWLKKLKEEVRLRNYNHITEHFGFMNTENFHQGVPLPVSLHPKTLQIGKDRLCRLQEAVDIPVGIENLAFSFSVDDVKEQGVFLDQLTEDTNGFLILDLHNIYCQSCNFGVEMKEIVDLYPLNKVKEIHLSGGSWQESAYGKKQVRRDTHDDVIPQDILSVLPSVMEQCQNLEYIIIERLGHTVKTEEEKVSFLNDFNTVRKIIEISDLKLAERNWMKKEMILPEKPLEDRVLYEEQSELTKLLFENADAAVIKNHDFHYFKTENWDSEMILTAQNIIKKWNPY
WP_031530742.1	Dyadobacter crusticola	MSQIFSTVACNLDAHILQASIPLFEMERVEAIEWSFDALFKVDVIPEWFTDFIKEFSDQNRLIGHGVYFSLFSGKWSAEQQKWLDQLSKLCDQFQFDHITEHFGFMTGSDFHKGAPLGIPFNASTLAIGKDRLARIQNACNCPVGLENLAFSYSLDEVKRHGDFLAQLVESVNGFIILDLHNLYCQCHNFELDFIDFLSFYPLERVREMHISGGSWDTQIPGRVVRRDTHDEAVPSEVFEYLRQAIPRCPNLKFVVMEQLGTALDTAEKQAGFQKDFLEMDRIVKSVGATLPDSVKNFQPLSQQVQIGPPIEDLTLHQQQMQLSEILEKARDLHEAKNLLLASDLSNTDWHIERWEPHMLQTAMNIAQKWKAGF
WP_034727520.1	Chryseobacterium sp. JM1	MKKPLLGLAMMPEADFVSAILPLLQTQSVDVLEWSFDTLYDVEEPEWLSGLLDFYSDNSRLLGHGVYYSLFDARWMERQEIWLKKLKEEVKRRKYNHITEHFGFMNTENFHQGVPLPVPLLPKTLQIGKDRLSRLQDAVEIPVGVENLAFSFSMDDVKEQGEFLDRLVEDIDGFLILDLHNIYCQSCNFKVDMMEIINLYPLEKVNEIHLSGGSWQESAYGKKPVRRDTHDDRIPEEILNLLPEVLIQCHPEYVIIERLGHTLNTEVAKQIFFDDFNRVKKILELSDYPVGEEKIWNRQNTVHSKPVEDLLLYAEQTELTRLLFDGNTTESIKNRDFHYFKPESWDEEMITTAQQIIKKWNPY
WP_050021162.1	Chryseobacterium sp. P1-3	MEKPFLGISMMAEADFLTAILPLLRDGSIDVLEWSFDTFYHAEEPDWLTRLLNFYSQNNRLLGHGVYYSLFDAKWTERQSMWLQKLKEEVQQRKYNHITEHFGFMNTENFHQGVPLPVSLHPKTLEIGKDRLCRLQEVLEIPVGIENLAFSFSIDDVKQQGIFLEKLIEDTDGFLILDLHNIYCQACNFGLGIEEIVRLYPLEKVREIHLSGGSWQTSPYGKRRIRRDTHDDTVPAEILAVLPSVLSQCENLEYVIIERLGHTIQTETEAKDFLDDFLKVKAIIEASEWNSRHKEIKIKKKPECAGRPVEDEGLYREQTILTKLLFDNVKADHFKDFDFQYFKTENWDPEMIRTAQNIIKKWNPY
KGP62404.1	Legionella norrlandica	MPEQDYMDASRPLFESGDVDIIEWSFDMCWGRKIPQPCLDLLTKYSSQGCLLGHGVSFSILTAKRTKRQELWLQYLQNEVNQLNYQRISEHFGFLSTNTFIQGPPLSMPFIPEMLEVGIKNLQQIKQIAKIPIGLENLGFAFCKQDVIEQGKFLTALLEAVDGFLLLDIHNIYCHMINFDMSLDEILDSLPLDRAKEMHISGGSFRSIEQNNRNIYCDSHDAAIPQDLFLLLEKNLSRFSQIDSLIFERLGNTLSNHYEAAQFREDFMTLKSLIRR
WP_034751030.1	Chryseobacterium vrystaatense	MKKPLLGLSMMPEADFVSAIVPLLQSNSVEVLEWSFDTFYDAEEPEWLSGLLNFYAGNERLIGHGVYYSLFDARWNDRQENWLQKLKEECKRRKYNHITEHFGFMNTENFHQGVPLPVPLLPKTLQIGKDRLRRLQDAVEIPVGVENLAFSFSIDDVREQGHFLDMLVEDIDGFLILDLHNIYCQACNFQIDMQEIINLYPLGKVREIHLSGGSWQESAYGRRPIRRDTHDESIPDEILKILPKVLSQCYNLEYIIIERLGNTLNNDTEKQIFFSDFNHVKEIIEVSVYPDIIRQDWKRTEKIYSEPVEDLLLHEEQTRLTKLLFDGNNVESVKNGKFLYFKPENWDEEMIMTAQQIIRKWNPY
WP_045496050.1	Chryseobacterium sp. StRB126	MGKPLLGLSMMAEAEFVSAVLPLLQSNSVEVLEWSFDTFYNTEEPDWLSELLHFYSENNRLIGHGVYYSLFDARWTERQEIWLKKLKEEFRKRNYNHITEHFGFMNTENFHQGVPLPVSLHSKTLEIGKDRLYRLQDAVQVPVGIENLAFSFSLDDVMEQGIFLNKLTEDTNGFLILDLHNIYCQSCNFGVEMQEIIKLYPLDKVKEIHLSGGSWQESVYGKKLIRRDTHDDAVPEDILSVLPSVLSQCKNLEYIIIERLGNTLDAVEKKESFLDDFTQIKKIIDSSGAGTQNKDLWTKKEIRFSENPLEDMILHEEQTLLTKLLFENAGPASIKSHEFHYFKTKNWDPEMILTAQNIIKKWNPY
WP_044220505.1	Phaeodactylibacter xiamenensis	MEYSGLQRPRATLACNLDTNLLRAAHPLLTAGQVEALEWSFDALFSFPEIPDWFTALIQAYSEEGRLVGHGVFFSLFSGRWSAEQQQWLDRLKALSEVYRFDHVTEHFGFMTGADFHKGAPLGIPFTPQTLQLGRDRLQRISAAAGCPVGLENLAFAYTLDEVKRHGDFLEQLLEPINGFIILDLHNLYCQLHNFRQSAEELLRLMPLHRVREIHLSGGSWEPAAGQRNRQIRRDTHDGTVPQAVFDLLEKALPKCPNLKYVVLEQLGGALETPEQQRAFQADFRTMHGILKRQNHSGKTSAPANDFQPPAFTPSATPRSDEALHQQQQALSEILENAASLRDARTQLSASPLAETAWNVENWEDDMLETARRIAQKWKGGWG
WP_040999004.1	Chryseobacterium oranimense	MNKPLLGLAMMPEADFVSAILPVLQNNSVDVLEWSFDTFYDVEEPEWLSGLLDFYAENDRLLGHGVYYSLFDAQWTSRQKDWLKKLKEETKCRKYNHITEHFGFMNTENFHQGVPLPVPLHAKTLQIGKDRLKRLQDTVEVPVGVENLAFSFSMDDVKEQGEFLEKLVEDIDGFLILDLHNIYCQSCNFEIDMQEIIRMYSLERVKEIHLSGGSWQESAYGNKPVRRDTHDDNIPVEITDVLPEVFSQCPNLEYVIVERLGHTLDTEVKKQLFFDDFNKVKEIIEVSDFPAGITKAWNKRKGESSEPVEDLLLYEEQTKLTKILFEGNTVQSVKNETFHYFNPENWDKEMITTAQQIIKKWNPY
WP_027385220.1	Chryseobacterium gregarium	MNDKPLLGLSMMADADFVSAVIPLLESDEIDILEWSFDTSYGADEPQWLTDLLNFYSENRRLLGHGVYYSLFDAKWTERQDYWLQQLKIETEKRNYNHITEHFGFMNTENFHQGVPLPVPLHSEILKIGKDRLLRLQDVVEIPIGIENLAFSLSANDVREQGDFLSRLIEDIDGFLILDLHNLYCQALNFNMDIIELVNLYPLEKVKEIHLSGGSWENSVYTKNKRIRRDTHDEAIPLELFSVLPEVMARCHHLQYIIIERLGHTLEGESDKLNFFTDYKKVRGIIYNSKRKKPSPHQWSKKDIVLADPLENLLLFNDQKNLNKLLFDSEDPERITNYPFNYFKTENWDLEMIDTAVQIVKKWNPY
WP_053328730.1	Chryseobacterium gallinarum	MEKPFLGISMMAEADFLTAILPLLRDGSIDVLEWSFDTFYHAEEPDWLTRLLNFYSQNNRLLGHGVYYSLFDAKWTERQSMWLQKLKEEVQQRKYNHITEHFGFMNTENFHQGVPLPVSLHPKTLEIGKDRLCRLQEVLEIPVGIENLAFSFSIDDVKQQGIFLEKLIEDTDGFLILDLHNIYCQACNFGLGIEEIVRLYPLEKVREIHLSGGSWQTSPYGKRRIRRDTHDDTVPAEILTVLPSVLSQCENLEYVIIERLGHTIQTETEAKDFLDDFLKVKAIIGASEWNSRHKEIKIKKKPECAGRPVEDEGLYREQTILTKLLFDNVKADHFKDFDFQYFKTENWDPEMIRTAQNIIKKWNPY
WP_034698536.1	Chryseobacterium sp. S0630	MRKPMLGVSMMAEADFVSAILPLLQNNSIDVMEWSFDTLYHTNEPDWLRDLLNFYAENNRLIGHGVYYSLFDAKWTERQEKWLKKLKEEVSLRKYNHITEHFGFMNTENFHQGVPLPVSLHSKTLQIGKDRLYRLQDAVNIPVGIENLAFSFSIDDVKEQGIFLDKLTEDTNGFLILDLHNIYCQSCNFELGMQEIIALYPLEKVKEIHLSGGSWQDSVYGKKQVRRDTHDDIIPQDILSVLPSVLTQCGNLEYIIIERLGHTVKTEQERDDFLNDFNKVKDIIETSDFNGKEKSSWSKMEMKLSERPLEDLVLYEEQSRLTRLLYDNANIETIKDLDFHYFKTEKWDSEMILTAQNIIKKWNPY
WP_046369149.1	Flavihumibacter petaseus	MTTLAAVALNPDSNLLTAALPLLEEEMVDAIEWSFDAVDTTGRLPAYFHDLLQFYASHHRLIGHGIYFSAFSGKWTPDQQEWLSRLAAVCRQFPLEQVTEHFGFMTGKDFHKGAPLNIPRNAATLAIGRDRLARIRQACNCPVGLENLAFSYSLEETRHHGAFLAELVEPVNGFIILDLHNCYCQAMNFSVDADRLLDTYPLHLVREIHISGGSWEPFPDAPGGMVRRDTHDDSVPQEVFDLLQLALRRCPNVRYVTLEQLSTALETTSAQEQFCKDFRRMRGMMAIPEGERQQAASGVPQVDFHRPIPLLTGQIPVEDMLLYQQQRELAGILESSEDLATAQKLLRNSSLAASDWKIENWAPYMVETARQIARKWK
WP_046244672.1	Hymenobacter terrenus	MEPTPAILASLACNLDSDILTAAFPLLEDGQVEALEWSFDALFRTQELPEWFVELLQTYSRRQRLVGHGVFFSLLSSKWTPEQQQWLAQLRQVSLQFAFDHITEHFGFFTGQNFHSGAPLPVPYTNTTLRIGQDRLRRISDACQCSVGLENLAFAYSLAEVRQHGEFLEKLIAPVNGFIILDLHNLYCQLHNFSVPYENLIGLYPLDCVREIHISGGSWEDSGVLPHKKVRRDTHDDAVPAEVFHLLELTMPKCPNLKYVVLEQLGTGLRSDSSKAQFRRDFQKMASMVQQHRHGPHRQPAHEFRPLQLPVLGTAIEDEQLQTQQIQLSHILENASTFEAAHRQLLSSSLANSAWEIENWEPHMLETAVRIAQKWKK
WP_044218508.1	Chitinophaga pinensis	MPKVLSAVACNLDANILAACLPLMEESRIEAIEWSFDALYKVKEVPDWFRELLTAFSDENRLIGHGVFFSLFSGKWLPEQENWLKHLSQTADAFQFDHITEHFGFMTGKDFHQGAPLNIPYTAITLNIGRDRLKRIYEACKRPVGLENLAFSYSLDEVKRHGAFLEQLLEPVNGFIILDLHNLYCQLRNFDVTFEEMIALYPLDRVREIHISGGSWEDSVATPSRSIRRDTHDDAVPTEVFQLLEMTIPRCPQLKYVVLEQLGNGLETETSRQYFYNDFLRMQEIIQQHNDQADDTSTDPFLPLIPFSTDAVVEDMMLYQQQLELSAIFESSASYGEAMQSLSTSSLANSDWRIEQWEPHMIETAVKIAQKWMK
WP_034713482.1	Chryseobacterium soli	MGKPLLGLSMMAETEFVSAVLPVMQTNSIEVLEWSFDTFYHTEEPEWLSELLDFYAESNRLIGHGVYYSLFDARWTGRQEMWLEQLKKETQKRNYNHITEHFGFMNTDNFHQGVPLPVSLNSKTLQVGKDRINRLQDAVDIPIGIENLAFSFSIDDVKEQGEFIHKLIEDIDGFIILDIHNLYCQACNFETDIWELIQLYPLEKVKEIHLSGGSWQKSAYGRNPVRRDTHDDSIPEEIFAIIPQIFSQCTHLEYVIVERLGHTIKTQKEKQCFFNDFNKVKEIIDASDDRLDNKKVWHKKEINLSAPVEDLFLYEDQTKLTKLLFDAVDETAIKRERFHYFEPEKWDLEMINTAQAIIKKWNPY
WP_062699011.1	Chryseobacterium indologenes	MKKPLLGLSMMPEADFVSAILPLLQDQSVAVLEWSFDTVEHVLEPEWLRGLLDFYAENSRLLGHGVYYSLFDARWTERQEVWLEKLKKETQRRKYNHITEHFGFMNTENYHQGVPLPVPLHSRILQIGKDRLAHLQAAVEIPVGVENLAFSFSADDVREQGIFLEKLIEDVEGFLILDLHNIYCQSCNFDIDIVDIVAMYPLEKVKEIHLSGGSWQESVYRKEPVRRDTHDDAIPEEVLAVLPQVLSRCPNLEYIIIERLGHTLNTTMEKEAFFNDFVRTKKIIEDSWFPAGNKTVWNRKECISSVPLEDHLLYHEQTKLTRLLYGNSDITSFKNENFSYFDVQKWDAEMIETARNIIKKWNPY

WP_051884865.1	Chryseobacterium luteum	MSKPLLGLSMMPEADFVSAILPLLQNNSVDVLEWSFDTFYDTQEPEWLSGLLDFYAENGRLLGHGVYYSLFDARWTERQEGWIEKLKEEFRHRKYNHITEHFGFMNTENFHQGVPLPVPLHSKTLQIGKNRLRRLQDAVEIPVGVENLAFSFSINDVKEQGEFLDKLIEDIDGFLILDLHNIYCQSCNFEINMQEIIRLYPLEKVKEIHLSGGSWQESAYGEKTVRRDTHDDSIPDEILNILPEVLSRCHNLEYVIIERLGHTLNSEAKKQAFFDDFNRVKEIIEISDPFTGNKKVWNKKNGQYSEPVEDLLLHNEQTKLTKMLFEGDSIQSVKNKPFHYFKPGKWDEEMITAAQQIIKKWNPY
WP_083997424.1	Chryseobacterium angstadtii	MKKPLLGLSMMPEADFVSAILPLLQDHSVEVLEWSFDTFFDEKEPEWLSGLLDFYSANNRLLGHGVYYSLFDARWTGRQEIWLEKLKEECKHRKYSHITEHFGFVNTENFHQGVPLPVPLHPQILQIGKDRLKRLQDAVEVPVGVENLAFSFSIEDVKEQGEFLYRLVEDIDGFLILDLHNIYCQSCNFEKDMQEIIRQYPLEKVKEIHLSGGSWQESAYGKKLIRRDTHDDRIPDEILLVLPEVLSQCPSPEYVIIERLGHTLNTEAERHIFLEDFKKVKDIIEAFDGYSDENKPGSRMTGKYSEPVEDFLLYDEQTKLTKLLFDENNVEFIKNQKFHYFKPENWDEEMINTAQQIIKKWNPY
WP_083996892.1	Chryseobacterium sp. BLS98	MKKPLLGLAMMPEADFVSAILSILQNNSVDVLEWSFDTFYDVEEPEWLSGLLDFYAENDRLLGHGVYYSLFDARWTSRQKDWLQKLKEEFRHRKYNHITEHFGFMNTENFHQGIPLPVPLHSKTLQIGKDRLKRLQDTVEVPVGVENLAFSFSMDDVKEQGEFLEKLIEDIDGFLILDLHNIYCQSCNFKIDMQELIRMYPLEKVKEIHLSGGSWQESSYGNKPVRRDTHDDDIPDEIIDVLPKVLSQCLNLEYVIIERLGHTLDTEGKKQLFFNDFNKVKEIIEVSDFPTGTTKPWNKRKRESSEPVEDLLLYEEQTKLTKILFEGNAAQSVKNEAFHYFKPENWDKEMIATAQQIIKKWNPY
KQM24483.1	Chryseobacterium sp. Leaf201	MMAEADFVSAILPLLESSGIDVLEWSFDTFYTVDEPQWLADLLNFYSDHKRLLGHGVYYSLFDAKWTERQDVWLQKLKAETDRRSYNHITEHFGFMNTENFHQGVPLPVPLHPELLKIGKDRLLRLQDVVEVPIGIENLAFSFSSDDVSEQGEFLYRLIEDIDGFVILDLHNIYCQASNFNMEIMELVNLYPLEKVKEIHLSGGSWENSIYTKNKRIRRDTHDDEIPQELFDILPDVISRCSHLEYIIIERLGHTIKTESDKQNFLRDFKKVKQIIDNSDPKENLQHQWKKKNRVLTDPMENLLLFDDQKNLNKFLFESQDPETIRNYAFHYFKMHNWDPEMIHTASQIVKKWNPY
WP_054508689.1	Chryseobacterium sp. ERMR1:04	MRKALLGLSMMAEPDFVTAILPLLENSEIEVLEWSFDTFYGVEEPQWLSNLLNFYAENNRLIGHGVYYSLLNAVWTDRHHEWLENLKTETQKRRYTHITEHFGFMNTENFHQGIPLPLPLNAVTLQTGKDRLQRLQNVLDVPVGIENLAFSFSKQDVEEQGEFLSRLIEDVDGFLILDLHNIYCQAHNFEIDMKELIRLYPLDQVKEIHLSGGSWQESIYSKDKYVRRDTHDDCIPEDIFQILPYVLKQCKNLEYVIIERLGNTIGAEKDKASFIKDFRRAKKIIDAIKLPLLTNHWIKKEKKIVEPFEDHLLFEDQKKLTKLLCESNTPEDIMTEKLHYFKSEDWNKEMINTAKNIIKKWNSY
WP_059069066.1	Solirubrum puertoriconensis	MEAAPPLYASVACNLDADLLAAAYPLLEEGRVEALEWSFDALYWAEQVPEWFTNLLHTYAGAGRLVGHGVFFSLLSGKWSPEQQQWLAHLGQLAEQFRFDHITEHFGFFTGQNFHHGAPLPIPYTATALRIGQDRLRRIYDACCCPVGLENLAFAYTLDEVKRHGNFLEELLLPVNGFVILDLHNLYCQLHNFGVPYDELIALYPLERVREIHISGGSWHDSEQAPSQRIRRDTHDDAVPEEVFQLLELTMPRCPNLKFVVLEQLGHALHAEASRTQFREDFGRMESLVARHRAALRTLPTNDFRPQQTWPPSTAAEDEHLHRQQRQLSDILENAPSYAEAQRLLQASGLAHSDWRIEQWEPHMLETALHIARKWKT
KXH81096.1	Chryseobacterium kwangjuense	MPEPEFVSAVLPILQDHSVDVLEWSFDTLYDLEEPEWLSGLLDFYAENNRLLGHGVYYSLFDARWTERQETWLEKLKKEVKHRNYNHITEHFGFMNTENFHQGVPLPVPLHSKTLQIGKDRLGRLQDAVEIPVGIENLAFSFSVNDVKEQGEFLDKLIEDIDGFLILDLHNIYCQSCNFETDMLEMIRMYPLEKVKEIHLSGGSWQESAYGKKPVRRDTHDDCIPDDILKILPEVLSLCPNLDYIIIERLGHTLKTESEQKIFFDDFESVKKIISKASYPSGQSKIWLKKKIGAYSEPVEDQLLYDEQTKLTQLLFNGTDIQSIKDQDFHYFKPENWDDEMIFSAQQIIKKWNPY
WP_162265596.1	Legionella santicrucis	MTPPKMGLSLMPEQDYLDASLPLFESGQVEVIEWSFDMCWGKKIPQRCHDLLEKYSDDNCLLGHGVSFSILTAKRTKRQEIWLQYLQKEMSHLHYQNISEHFGFLTTDTFIQGPPLSMPFIPELVEVGINNLQEIKQIAQIPIGLENLGFAFSRQDVIDQGKFITALLDAVDGFLLLDIHNIYCHMLNFNMSFEEILETLPRHRLKEMHISGGSFRDICYQNKTIYCDSHDAAIPEDLFLLLEKNLPYLLPMESIIFERMGNTFSSPYEVSRFQDDFANLKSIVQKASDEMFK
WP_059013965.1	Flavobacterium psychrophilum	MKKPQLGISLMTEPAFLEACFPLFAEGEIEVLEWSFDTIQTIEDEPLWLNELLSTYSRENKLLGHGVYYSLFDAYWGDKQDLWINKIKKETERYKYNHISEHFGFMSSENYHKGVPLPIPLSDSTLNIGIDRLKRIQNAVDLPVGIENLAFSFSKKDVFEQGVFIEKLIDSVNGFLVLDLHNIYCQSHNFEIDMMELINYYPLSKVKEIHISGGSWQDSIYKEKQIRRDTHDGNIPLEILSILPLVLSKCVNTDFVIFERLGNTFNNSYDVTEYQKDYKELKKIVQQIEYKSNQESWRKEYKISEKPLVNFELKKEQQKLSNFLLTEDYNEIKNKEFKYWDVNSWDIEMINTAIEISKKWNQ
WP_063962962.1	Chryseobacterium kwangjuense	MKKPLLGLSMMPEPEFVSAVLPILQDHSVDVLEWSFDTLYDLEEPEWLSGLLDFYAENNRLLGHGVYYSLFDARWTERQETWLEKLKKEVKHRNYNHITEHFGFMNTENFHQGVPLPVPLHSKTLQIGKDRLGRLQDAVEIPVGIENLAFSFSVNDVKEQGEFLDKLIEDIDGFLILDLHNIYCQSCNFETDMLEMIRMYPLEKVKEIHLSGGSWQESAYGKKPVRRDTHDDCIPDDILKILPEVLSLCPNLDYIIIERLGHTLKTESEQKIFFDDFESVKKIISKASYPSGQSKIWLKKKIGAYSEPVEDQLLYDEQTKLTQLLFNGTDIQSIKDQDFHYFKPENWDDEMIFSAQQIIKKWNPY
WP_056291594.1	Dyadobacter sp. Leaf189	MPQIFSTIACNLDAHILQSSIPLFEMEKVEAIEWSFDALFKADVVPEWFTDLIGEFSDQGRLICHGVYFSLFSGKWSAEQQHWLDQLSKLCGQFHFDHITEHFGFMTGSDFHKGAPLGIPFNASTLAIGKDRLARIQNACNCPVGLENLAFSYSLDEVKLHGDFLGQLVESVNGFIILDLHNLYCQCHNFELDFTEFLSFYPLDRVREMHISGGSWDAQIPGRIVRRDTHDEAVPPEVFEYLRQAIPRCPDLKFVVMEQLGTALDTAEKQADFQKDFLQMDHIIKSVGETGRISANNFLPFSPTSPAGLPVEDLALYQQQMQLSNILEIAGDLKEAQRLLNASDLSDTDWHIEHWEPHMLQTAMNIAQKWKGGF
WP_062670360.1	Chryseobacterium cucumeris	MRKPILGVSMMAEPDFVSAILPLLHNNSIEVMEWSFDTLYHTNEPDWLRDLLNFYAENNRLIGHGVYYSLFDARWTARQEEWLKKLKEEVSLRKYNHITEHFGFMNTENFHQGVPLPVSLHSKILQIGKDRLYRLQDAVNIPVGIENLAFSFSIEDVKEQGVFLDKLTEDTNGFLILDLHNIYCQSCNFELEMQQIIDLYPMDKVKEIHLSGGSWQDSVYRKKQVRRDTHDDVIPKDILSVLPSVMKKCPNLEYIIIERLGHTLKTEEKRNSFMEDFTKVRTIIETSDLKLKQQNGWCKEEIKLPAKPLEDLLLYEEQSRLTRLLYENANVENIKDLDFHYFKTENWDAEMILTAQNIIKKWNPY
WP_056076704.1	Chryseobacterium sp. Leaf394	MNNKPLLGTAMMAETEFVSAVLPLLENNEIDILEWSFDTFYHADEPKWLSGLLDFYSENNRLIGHGVYYSLFDAKWTDRQEHWIEKLKSETKKRNYNHITEHFGFMNTENFHQGIPLPVPLHSKILQLGKDRLLRLQDALEIPIGIENLAFSFSADDVKEQGEFLEKLIEDIDGFLILDLHNLYCQAVNFNIDILELVAMYPLEKVREIHLSGGSWENSSYTEEKRIRRDTHDDSIPREILEVLPKVLSVCHQLEYIIIERLGHTLTEKSDTTNFLNDFKKVRQIVNASEVNSFLKKNWKKKNQITGKPLEDLLLYEDQTKLTKLLFDSVNPEIIKNYHFNYFEIEKWDLEMIDTASKIIKKWNPY
WP_065397536.1	Chryseobacterium arthrosphaerae	MKKPLLGLSMMPEADFVSAILPLLQSNSVEVLEWSFDTFYEAKEPEWLDELLNFYSENQRLIGHGVYYSLFDALWTERQENWLKKLKEEVRKRKYNHITEHFGFMNTENFHQGVPLPVPLHPKTLLIGKDRLYRLQDAVQLPVGIENLAFSFSIEDVKEQGVFLDNLIADINGFLILDLHNIYCQACNFEVEMEDIIRLYPLDKVREIHLSGGSWQESVYARKMIRRDTHDDLIPEEIFSVLPAVITQCNNLEYVIIERLGHTIKTDQEKRAFLDDFTRARRIIDDSEIKTGKKIRWGQKEMGFSNSPVEDDALYEEQSRLTKLLFDSSGAEMIKEQRFHYFKTENWDPDMILTAQNIIKKWNPY
WP_065394721.1	Chryseobacterium artocarpi	MKKPLLGISMMAESEFVSAILPLLQSSSVEVLEWSFDTFYNAKEPVWLSDLLNFYSENNRLIGHGVYYSLFDAKWSDRQEVWLERLKQEVHKRQYNHLTEHFGFMNTENFHQGVPLPVSLHSQTLQIGKDRLHRLQDAVEIPVGIENLAFSFSVDDVKEQGLFLDKLIEEIDGFLILDLHNIYCQACNFDVDMLEIIQSYPLDKVKEIHLSGGSWQESVYGKKSIRRDTHDDDIPDEIWAVLPSVLLQCKNVQYVIIERLGHSIKTERDKQNFLSDFVKAQTIINVLDWSGRENRFWSKKEVNISKKPLEDVILYEEQTRLTKLLFDSIDPVLVKKQDFQYFNTETWCEEMIMTAQNILKKWNPY
WP_066690840.1	Chryseobacterium contaminans	MGKPLLGLSMMADVEFLSTILPLLQSNSVEVLEWSFDTFYNAQEPDWLHDLLNFYSESNRLVGHGVYYSLFDALWTERQEVWLKKLREEFRKRKYNHITEHFGFMNAENFHQGVPLPVSLHAKTLQIGKDRLYRLQDAVEVPVGIENLAFSFSVDDVMEQGIFLDKLTEDTNGFLILDLHNIYCQSCNFEVEMSEIIQLYPLDKVKEIHLSGGSWQESVYRKKPVRRDTHDDAIPGSIFSVLPSVLSQCKNLEYIIIERLGDTIDTEDKKKSFLDDFIQVKTLIDSSGREIQNKNSWTKKEVSFPENALEDLLLHEEQTLLTRLLFDNTEATYIKSHEFHYFKTENWDPEMILTAQNIIKRWNPY
SEH31972.1	Chryseobacterium culicis	MMAEADFVSALLPLLQSNSVDVLEWSFDTLYHNHEPVWLCDLLNFYADNERLIGHGVYYSLFDARWTERQEDWLKKLKEEVALRRYNHITEHFGFMNTENFHQGVPLPVSLHAKTLEIGKDRLYRLQDAVNIPVGIENLAFSFGIDDVKEQGAFLDKLIEDTDGFLILDLHNIYCQACNFGIEIQEIMDWYPLDKVKEIHLSGGSWQESVYGKRPVRRDTHDDSIPKELFSVLPSVVSQCQHLEYVIIERLGHTLKTDEEKNDFLTDFNRVKKIIEASDWKGEQKKNWKKKEIKLTEKPLEDRVLFEEQSRLTQLLFDHVDAAVIKDVDFHYFKTENWDLEMILTAQNMIRKWNPY
WP_068943005.1	Chryseobacterium	MRRPLLGLSMMAEAEFISAIMPLLQSNSVEVLEWSFDTFYNMEEPSWLGDLLNFYAENNRLIGHGVYYSLFDAQWSERQEMWLSQLKQEFQKRKYNHITEHFGFMNTENFHQGVPLPVSLHSKTLEIGKDRLYRLQDAVQVPVGIENLAFSFSLDDVMEQGAFLDQLTEDVDGFLILDLHNIYCQSCNFEVEMQEIIKLYPLNKVREIHLSGGSWQESAYGKKPVRRDTHDDSIPEEILLILPSILSQCENLEYMIIERLGHTINTEEKRNRFLDDFDKVRALIDSSDLEIQNKNPWSKKEVQFPQKPLEDMILHEEQTLLTKLLFDSVEAVSIQNHEFHYFKTENWDPEMILTAQNIIKKWNPY
WP_245705439.1	Chitinophaga filiformis	MPKVLSAVACNLDANVLAACLPLMEESRVEAIEWSFDALYKVKEVPSWFRELLTAFSQEKRLIGHGIFFSLFSGKWLPEQENWLKHLAQTAAEFQFDHITEHFGFMTGKDFHHGAPLNIPYTTTTLNIGRDRLKRIYEACGRPVGLENLAFSYSLDEVKRHGAFLEQLLEPVNGFIILDLHNLYCQLRNFDISFDEMIALYPLERVREIHISGGSWDDSNADPKRSIRRDTHDDAVPPEVFQLLEMTIARCPHLKYVVLEQLGNGLATEESRRCFYNDFLQMEEIVRRNNNENGTDASDPFIPLFPLSTGTVVEDMTLYQQQLELSSILESSASYTEAIYALTQSSLANSDWRIEQWEPYMIETAVKIAQKWKK
WP_090148480.1	Dyadobacter soli	MSEIYASVACNLDAHILQASLPLFEQEKIEAIEWSFDTLFKFDEIPAWFTDLVSEFSNHNRLIGHGVYFSLFSGKWSPAQQQWLDKLKRLSAEFRFDHITEHFGFMTGEDFHKGAPIGIPRTARTLALGQDRLRRIQDACQCPVGLENLAFSYSVDEVKKHGEFLDQLVETVNGFIILDLHNVYCQVHNFDIAFDDIIKLYPLNKVREIHISGGSWQQTEAIPGKSVRRDTHDESVPEAVFEFLGKAIPECPYLKFVVLEQLGTALDTPSSQAAFQADFLRMDYIIKSLAHGYPQAAVYPFAPLPGLAPPVPLEDPDLHKQQVQLSRILETAGSVSEATAMLQSSDLRNTAWEVEKWAPYMLETAISIAQKWKDGFV
WP_090332217.1	Dyadobacter koreensis	MPKILSSIACNLDTHILLAALPLFEQQKIEAIEWSFDTLFKYEEIPEWFTDLISEFSNQGRLVGHGVFFSLFSGKWLPEQDIWLKKLTAFSERFKFDHITEHFGFMTGSDFHKGAPIGIPYTKSFLDIGIDRLKRIQDACQRPVGLENLAFSYSLEEVGNHGDFLSKLVESVNGFIILDLHNLYCQIHNFRMDFEDIIVLYPLEKVRELHISGGSWDERKTATGEKIRRDTHDDRVPEEVFNLLSATIPKCPNLKFVVLEQMGTALTSEENQLAFQRDFLKMDAIVKSINYAFPQSLPNNFLPVNRAILSRFPLENLALFEQQQQLSYILETAENFGHAQQLLAKSALHQTEWKVENWEPYMLETAMAIAQKWRNGF
WP_218144591.1	Hymenobacter actinosclerus	MASTPSSATTPASPVLAALACNLDADLLTAAFPLLEQGRVAALEWSFDALFAVEQVPEWFQELLRAYSAQGRLLGHGVYCSLLSGRWTHAQQQWLTQLRTLARQLPFDHVTEHFGFFTGANFHAGAPLPIPYTSATLRLGQDRLQRVQAACECPVGLENLAFAYSLDEVRRHGEFLEKLVEPVNGFLILDLHNVYCQLHNFRLPADELLALYPLHRVREIHLSGGSWAESGQEPGRQIRRDTHDDAVPAEVFQLLKWALPRCPNLKYVVLEQVGGALRTARSQAQFRVDFGRMEALVQEYQKGSARQPGNAFRPRLPLAPPGPVIEDELLHTQQRQLGHILETAASFEQARQRLQASSLAHTAWQIEHWEPAMLETAISIAQKWQ
ASS50882.1	Candidatus Fluviicola riflensis	MIKQQHILASVACNLDQHLLQATLPLFIDEQVEAIEWSFDALYKVRNIPDWFVELLTTFGKEGRLIGHGVFFSLFSGKWSVEQERWLNHLKKVSGNFTFNHITEHFGFMTGEDFHKGAPIGIPYNSTTLGIGIDRLKRIQDTCQCPVGLENLAFSYSLEEVKRHGEFLNELIEPVNGFIILDLHNFYCQLHNFSLDAEELLELYPLDRVREIHISGGSWEQTRLEPQRTIRRDTHDDAVPEAVFELLKLVIPRCPNLKFVVLEQLGIALDSTEKQQQFQADFSRLKSIVSTFDSPNPVNDFLPKSTFVLSSSVAEDSFLASQQQELSDILENAINHENAVVLLAKSGLANSDWKVEEWSPYMLETAMRIAQKWKNGW
WP_245752765.1	Chitinophaga arvensicola	MPEILSAAACNLDSNILAACLPLMEESRLEAIEWSFDALFNVKDIPSWFLELLTAYSHEKRLTGHGVFFSLFSGKWRQEQADWLKRLKKINSTFRFDHVTEHFGFMTGKDFHHGAPLNIPYTPTTLRIGQDRLKRIFEACECPVGLENLAFSYSLEEVKKHGAFLEQLLEPVNGFIILDLHNLYCQIHNFNIPFEEILALYPLHKVREIHISGGSWQPAPDNPVKKIRRDTHDDAVPEEVFALLEHTIARCPHLKYVVLEQLGNALYTANSKHLFYTDFGRMEKIVQANNKIHKAVSLQTFLPLSPIINGAVIEDETLNAQQLELSRILESAHSHHEAIYRLSQSSLAHSAWQVEEWEPDMIATAVSIAQKWKK
WP_090381944.1	Dyadobacter sp. SG02	MSEIYSSIACNLDAHILQASLPLFEQERVEAIEWSFDTLFRFDEIPAWFTDLVSEFSNGNRLIGHGVYFSLFSGKWSSGQQEWLDKLKKLSDEFLFDHITEHFGFMTGEDFHKGAPIGIPFTSQTLALGKDRLRRIQDACRCPVGLENLAFSYSMDEVKKHGDFLRELVESVNGFIILDLHNIYCQVHNFDIAFDDIIKLYPLDKVREIHISGGSWEHTLMNSGRPVRRDTHDDAVPEVVFEFLGSAIPKCPNLKYVVLEQLGTALDNESSQAAFQSDFLRMDSIIKSLTPKSPQIPADTFAPLPANANTTPLEDPRLHRQQVQLSRILETASDVNEARTMLLSSDLHNTAWNVENWAPYMLETAIAIARKWRNGFE
SFN39809.1	Chryseobacterium oleae	MMPEAEFVSAILPLLQTQSVDVLEWSFDTFYDAEEPEWLSGLLDFYAENNRLLGHGVYYSLFDGRWANRQEIWLKKLKEEVKRRKYNHITEHFGFMNTENFHQGVPLPVPLLPKTLQIGKDRLSRLQDAVEIPVGVENLAFSFSMDDVKEQGEFLDKLVEDIDGFLILDLHNIYCQSCNFEVDMMEIINLYPLEKVKEIHLSGGSWQESAYGKKPVRRDTHDDRIPEEILNLLPEVLILCHPEYVIIERLGHTLNTEAEKQIFFDDFNRVKKIIELSDYPVDEEKIWNRQNTVHSKPVEDLLLHAEQTELTRMLFDGNTTESIKNRNFHYFKPESWDEEMITTAQQIIKKWNPY
WP_093832332.1	Spirosoma endophyticum	MTPIRSTLACNLDTNLLSACLPLFEAGSVQAIEWSFDTLYQHRQSPDWFTELLTTFSDAGCLIGHGVFFSLFRGQWTPDQQAWLDQLKDLASTYQFNHITEHFGFMTGADFHKGAPLSIPLNASTLAIGQDRLRRIQDACGCPVGLENLAFAYSVDEVKRQSDFLNRLLEPINGFLILDLHNLYCQSQNFGMSGADLLDLYPLDRVREIHISGGSWSTSTIEPERIIRRDTHDDSVPAEVFAWLEQAINRCPNLRYVVLEQLGPALATEQERHFFQDDFRRMDQLVQAANQTLSVRETNTFLPANSSANPSVPVEDTFLFAQQRTLATILETAIDTRQAQTLLATSSLANSDWAIEQWEPAMLETALAIAQKWRNGFN
WP_089733898.1	Chryseobacterium jejuense	MGKPLLGLSMMAEAEFVSAVLPLLQSNSVEVLEWSFDTFYNVEEPDWLSELLDFYSENNRLIGHGVYYSLFDARWTDRQEIWLKKLQKEFRKRNYNHITEHFGFMNTENFHQGVPLPVSLHPKTLKIGKDRLYRLQDAVQVPVGIENLAFSFSLDDVMEQGVFLDKLTEDTDGFLILDLHNIYCQSFNFEVEMQEIIKLYPLDKVKEIHLSGGS

		WQESVYGKTLVRRDTHDDIIPEDILFVLPSILSQCKNLEYVIIERLGNTINTDEKKKNFLDDFTKVKNLIDSSDFGTENKDPWTKKEVRFSENPLEDLMLHEEQTLLTKLLFDNAGPESIKNHDFHYFKTKNWDPEMILTAQNIIKKWNPY
WP_255373497.1	Chitinophaga sp. CF118	MPKILSAVACNLDANILSACMPLMEESRVEAIEWSFDALYKVKEVPSWFRELLTAFSNEGRLIGHGVFFSLFSGKWLPEQESWLKHLEQTAAEFRFDHITEHFGFMTGKDFHHGAPLNIPYNTTTLNIGRDRLKRIYEACRRPVGLENLAFSYSLDEVKRHGAFLEQLLEPVNGFIILDLHNLYCQLRNFDIAFDEMIALYPLDRVREIHISGGSWDDSAADPNRSIRRDTHDDAVPPEVFRLLEMTITQCPHLKYVVLEQLGNGLITETSKRCFYNDFLQMEEIVRKNSNESDAGSPDPFLPLSPLSTGIAVEDMTLYQQQLELSSILESSASYAEAMNVLSQSSLANSDWRIEQWEPYMIETAVKIAQKWKK
WP_072362160.1	Chitinophaga sancti	MPKILSAVACNLDANILAACLPLFEESRVEAIEWSFDALYKVKEVPGWFRELLSAFSDENRLIGHGVFFSLFSGKWLPEQEAWLSHLQQTATEFSFDHITEHFGFMTGEDFHHGAPLNIPYSSSTLNIGKDRLKRIYHACGRPVGLENLAFSYSLDEVKRHGTFLEQLLEPVNGFIILDLHNLFCQLKNFDLDFNTLISLYPLDKVREIHISGGSWDDSAAAPDRSIRRDTHDESVPPEVFQLLEMTIPQCPNLKYVVLEQLGNGLQSTASKQGFYNDFLQMQEIAQQHNYKEGNPFLPLESLPIITGAAIEDLRLYQQQLELSAILENAGTYAEAMQQLQQSSLAHSDWKIEEWEPYMIETAVKIARKWKK
WP_073063557.1	Chryseobacterium oranimense	MNKPLLGLAMMPEADFVSAILPALQNNSVDVLEWSFDTFYDVEEPEWLSGLLDFYAENDRLLGHGVYYSLFDAQWTSRQKDWLKKLKEETKRRKYNHITEHFGFMNTENFHQGVPLPVPLHAKTLQIGKDRLKRLQDTVEVPVGVENLAFSFSMDDVKEQGEFLEKLVEGIDGFLILDLHNIYCQSCNFEIDMQEIIRMYPLERVKEIHLSGGSWQESAYGNKPVRRDTHDDNIPVEIIDVLPEVFSQCPNLEYIIIERLGHTLDTEVKKQLFFDDFNKVKEIIEVSDFPAGITKAWNKRKGESSEPVEDLLLYEEQTKLTKILFEGNTVQSVKNEAFHYFNPENWDKEMITTAQQIIKKWNPY
WP_217646801.1	Pontibacter chinhatensis	MPGILATVACNLDADILSATYPLLEEGRVEAMEWSFDTLFQVEQLPDWFREMLLAYSREGRLIGHGVFYSLISGRWSREQAQWLKNLRQLSAQFHFDHITEHFGFFTGQNFHHGAPLSIPYTSTTLRIGRDRLLRIQDACQRPVGLENLAFSYSLDDVKQHGEFLEKLIAPANGFIILDLHNLYCQLHNFSVSYKELIRLYPLERVREIHISGGSWEEAPEAPGQKVRRDTHDDAVPAAVFGLLEQTMPLCPNLKYVVLEQLGNGLKTESSRAGFQRDFRQLEQLVQQANAVRTGHQPANLFLPLNALPGSAVEEDEALHQQQVLLSQILENAPSYQEAQRLLQTSSLAHSDWRIENWQPYMLETAIRIAQKWKK
WP_255373162.1	Chitinophaga sp. YR627	MPKVLSAVACNLDANILAACLPLMEESRIEAIEWSFDALYKVKEVPAWFQELLTAFSDENRLIGHGVFFSLFSGKWLPEQENWLKHLSQTANAFQFDHITEHFGFMTGKDFHHGAPLNIPYTATTLNIGRDRLKRIYEACKRPVGLENLAFSYSLDEVKRHGAFLEQLLEPVNGFIILDLHNLYCQLRNFDVTFEEMIALYPLDRVREIHISGGSWEDSVATPSRTIRRDTHDDAVPAEVFQLLEMTIPRCPQLKYVVMEQLGNGLETAESKQYFYNDFLRMQEIIQQHSDQTASPVTDHFLPLLSFSTDTIVEDMTLYQQQLELSAIFESAASYDEAIQALSASSLANSDWRIEQWEPHMIETAIKISQKWMK
OIN58769.1	Arsenicibacter rosenii	MHILQAGLPLFASEQVAALEWSFDSLYAQTELPDWFDELLLTYGQANRLVGHGVFFSIFAGRWRPEQQTWLNQLADRANRYRFDHVTEHFGFMTGASFHAGAPMSVPLNTTTLRIGQDRLHRIQQAAGCAVGLENLALAYSLDDVKRQGDFLHQLLAPVDGLLIMDLHNLYCQSHNFGVDWRELLALYPLDRIREIHLSGGSWEDSISEPGRIIRRDTHDEAVPGLLFDWLPVAIDQCPNLKYVVLEQLGRGLVTDAARLQYQADFTRIANSVAAHPKASVQGPAPAFRPETPVELPAQPLEDETLAWQQQHLTTILETAGSAHEARQQLAASPLAHSDWGTERWQPAMLETALRIAQKWTNGFSGLQ
SEW45833.1	Chitinophaga sp. YR573	MEESRVEAIEWSFDALYKEKEVPSWFRELLTAFSNEKRLIGHGVFFSLFSGKWLPEQEAWLKQLEQTAAEFRFDHITEHFGFMTGKDFHHGAPLNIPYTTTTLNIGRDRLKRIYEACRRPVGLENLAFSYSLDEVKRHGTFLEQLLEPVNGFIILDLHNLYCQLRNFDIAFDEMIALYPLDRVQEIHISGGSWDDSAADPNRSIRRDTHDDSVPPEVFQLLEMTIAQCPHLKYVVLEQLGNGLVTEMSKRCFYNDFLQMEEIVRKNNNENDAGASDPFLPLFPLSTGTAVEDMMLYQQQLELSSILESSASYAEAMHVLSQSSLANSDWRIEQWEPYMIETAVKIAQKWKK
WP_073174028.1	Chryseobacterium vrystaatense	MKKPLLGLSMMPEADFVSAIVPLLQSNSVEVLEWSFDTFYDAEEPEWLSGLLNFYAGNERLIGHGVYYSLFDARWNDRQENWLQKLKEECKCRKYNHITEHFGFMNTENFHQGVPLPVPLLPKTLQIGKDRLRRLQDAVEIPVGVENLAFSFSIDDVREQGHFLDMLVEDIDGFLILDLHNIYCQACNFQIDMQEIINLYPLGKVREIHLSGGSWQESAYGRRLIRRDTHDDSIPDEILKILPKVLSQCYNLEYIIIERLGNTLNNDTEKQIFFSDFNHVKEIIEVSVYPDIIRQDWKRTEKIYSEPVEDLLLHEEQTRLTKLLFDGNNVESVKNGKFLYFKPENWDEEMIMTAQQIIRKWNPY
WP_291042127.1	Dyadobacter sp. 50-39	MSEIYSSIACNLDAHILQAALPLFEQEKVEAIEWSFDTLFKFDEIPAWFTDLVGEFSNHDRLIGHGVYFSLFSGKWSREQQEWLDKLRKLSGEFRFAHITEHFGFMTGEDFHKGAPISIPFTSRTLALGKDRLERIQDACQCPVGLENLAFSYSIDEVKKHGDFLGQLVGSVNGFIILDLHNVYCQTHNFNIDFDDIMRLYPLDRVREIHISGGSWDDILAAPGRPVRRDTHDERVPEAVFDFLSKAIPKCPNIRFVVLEQMGTALETPERQAAFQSDFLRMDGIVRASASRSAHAPAYAFAPAGTLPNDMPLEDPDLYNQQVQLSRILETASDAYEARALLLASDLRNTAWQVENWAPYMLETAVSIARKWKDGFIWK
WP_073335882.1	Chryseobacterium carnipullorum	MSKPFLGLSMMPEADFVSAILPLLQNNSVDVLEWSFDTFYDVNEPEWLSGLLDFYAANNRLLGHGVYYSLFDARWNERQEIWLKKLKKECANRKYNHITEHFGFMNTENFHQGVPLPVPMHPLTLQIGKDRLKRLQDAVEIPVGVENLAFSFSIDDVKEQGVFLDQLVEDIEGFLILDLHNIYCQSCNFGVSMQEIIKLYPLEKVKEIHLSGGSWQESAYAKKPVRRDTHDDRIPDEIVEMLPEVLSQCQNLEYIVIERLGHTLSAESDKLFFFADFNRVKEIISKSEDRSEDKKVWNRRIGVDSKPVEDMVLYDEQTKLTKMLFDESSVAKVKNEAFQYFKPENWDEEMIMTAQQIIKKWNPY
OJY27962.1	Myxococcales bacterium 68-20	MTLAPELVSRDHRGRMHPWIGLSLMLEDDFRLAAGPLFAEGVVDVLEWSFDTGWGRAIPEWADALLDHYGEAGRLLGHGVHYSAFSARWEERQARWLERLAGEVARRTYVHVSEHYGFMTAAPFMRGAPLPVPRGEASRSVGRDRLERMRAVLAAGRNDACPIGLENLALAWNRDEALAHGAFLGEVLDHAEDFIVLDVHNLHCQIENFDIDPDELLDSFPAARIRELHVSGGSFLPAWPHHPEETVRCDTHDGDVPAPVFDLLERALDRFPDVRAVILERLGGTLRTQADIDTFRRDYLRVKQIVDHSRDGEGARDGDG
WP_072923384.1	Chryseobacterium sp. OV279	MKKPLLGLAMMPEADFVSAVLPLLQTQSVDVLEWSFDTFYDAEELEWLSGLLDLYAENNRLLGHGVYYSLFDARWADRQEVWLKKLKEEVKRRRYNHITEHFGFMNTENFHQGVPLPVPLLPKTLQIGKDRLSRLQDAVEIPVGVENLAFSFSMDDVKEQGEFLDKLVEDIDGFLILDLHNIYCQSCNFEVDMREIINLYPLEKVKEIHLSGGSWQDSAYGKKPVRRDTHDDRIPEEILNLLPEVLIQCHPEYVIIERLGHTLHTEAEKQIFFDDFNRVKKIIELSDYPVGEEKIWNRKKTVHSQPVEDLLLYAEQTELTRLLFDGNTTESIKNRDFHYFKPESWDEEMITTAQQIIKKWNPY
WP_073852115.1	Pontibacter flavimaris	MPGILATVACNLDADILSATYPLLEGGSVEAMEWSFDTLFQVEQLPDWFREMLQAYSQEGRLIGHGVFYSLISGRWTEKQAQWLKNLRQLSAAFHFDHITEHFGFFTGQNFHYGAPLSIPYTSTTLRIGRDRLLRIQDACQRPVGLENLAFSYSLDEVKQHGEFLEKLIEPANGFIILDLHNLYCQLHNFSVSYEELILLYPLERVREIHISGGSWEEAQEAPGHKVRRDTHDDAVPAEVFQLLKQTMPLCPNLKYVVLEQLGNGLKTESSRAGFQRDFRQMELLVQQVNAARTGLLPDNLFLPLSAFPDSFVEEDEALHQQQVLLSQILENAPSYQEAQRLLQTSSLAHSDWRIENWQPYMLETAIRIAQKWKK
SIO13075.1	Chitinophaga niabensis	MQESRVEALEWSFDALYKVKDIPSWFIELLTAYSDEKRLIGHGVFFSLFSGKWLKEQENWLKQLKQTCTEFQFDHITEHFGFMTGKDFHHGAPLNIPYTSTTLNIGRDRLKRIYDVCRCPVGLENLAFSYSLDEVKRHGDFLEQLLSPVNGFIILDLHNLYCQIHNFNISFDDIIALYPLDRVREIHISGGSWEGSLVDPPRQIRRDTHDDAVPEEVFHLLENTIERCPNLKYIVLEQLGNGLSTDESKTTFYQDFLKMESIAGRFSSQGKQTFVPLSPWQTGYPVEDETLYQQQRELSSILESAASYETAVNALSVSSLAQSDWGIEHWEPYMIETAIKIAQKWKK
WP_074240080.1	Chitinophaga niabensis	MPKILSAVACNLDNDILAACLPLMQESRVEALEWSFDALYKVKDIPSWFIELLTAYSDEKRLIGHGVFFSLFSGKWLKEQENWLKQLKQTCTEFQFDHITEHFGFMTGKDFHHGAPLNIPYTSTTLNIGRDRLKRIYDVCRCPVGLENLAFSYSLDEVKRHGDFLEQLLSPVNGFIILDLHNLYCQIHNFNISFDDIIALYPLDRVREIHISGGSWEGSLVDPPRQIRRDTHDDAVPEEVFHLLENTIERCPNLKYIVLEQLGNGLSTDESKTTFYQDFLKMESIAGRFSSQGKQTFVPLSPWQTGYPVEDETLYQQQRELSSILESAASYETAVNALSVSSLAQSDWGIEHWEPYMIETAIKIAQKWKK
WP_076552343.1	Chryseobacterium ureilyticum	MKKPLLGISMMAESEFVSAILPLLQSSSIEVLEWSFDTFYNAKEPVWLSDLLNFYSENSRLIGHGVYYSLFDAKWNDRQKIWLEKLKKEVHKRQYNHLTEHFGFMNTENFHQGVPLPVSLHSQTLQIGKDRLHRLQDAVDISVGIENLAFSFSVDDVKEQGLFLDQLIEDIDGFLILDLHNIYCQACNFEVDMQEIIQSYPLDKVKEIHLSGGSWQESIYGKKSIRRDTHDDDIPDEIWAVLPSVLLQCKNVQYVIIERLGHSIKTERDKQNFLSDFIKAQTIINDLDWSGRENRFWSKKEVNISKKPLEDIVLYEEQTRLTKLLFDNVEPSLIKKENFQYFNTKIWNEEMIMTAQNILKKWNPY
OJV53697.1	Bacteroidetes bacterium 43-16	MEQMIEGIIPSIACNLDADMLSASLPLFEAGVVGGLEWSFDALHARNYQVPDWFHELLLAYSDAERLVGHGVFFSLFSGRFSEAQQAWLKRLSALTKVYQFDHITEHFGFMTGQDFHKGAPLSVPYTPSTLAIGRDRISRIADAAQCPVGLENLAFAYDLEEVKIQGRFLDELLTPVNGFIILDLHNLYCQLHNFNLDIATLLALYPLDKVREIHISGGSWESHPQAPFMKVRRDTHDDAVPAAVFELLRMALAQCPNLKFVVLEQLGIALSSPAQRVQFQQDFNTMKTIIEQYNRQQQWPRDIAAFRADQIQLAPAALSDEALFAQQQIISHILETASDHKDVIDKMQHTSLAGSDWHIENWHPAMVDTAIHIAQKWK
WP_170872041.1	Chryseobacterium sp. RU33C	MLGVSMMAEADFVSAVLPLLQNNSIDVMEWSFDTLYHANEPDWLRDLLNFYAENNRLIGHGVYYSLFDARWTERQEEWLKKLKEEVNLRKYNHITEHFGFMNTENFHQGVPLPVSLHPKTLQIGKDRLYRLQEAVNIPVGIENLAFSFSIDDVKEQGVFLDKLTEDTNGFLILDLHNIYCQSCNFELEMQEIIDLYPLDKVKEIHLSGGSWQDSVYGKKQVRRDTHDDVIPRDILSVLPSVLTQCENLEYIIIERLGHTLKTDEERDHFLDDFSKVKTIIETSELNGKEKSNWSKMKVELSEKPLEDPVLYEEQSRLTQLLYDNADVETIKDLDFYYFKTENWDSEMILTAQNIIKKWNPY
WP_084180480.1	Chryseobacterium joostei	MRKPLLGLSMMAETEFVSAILPLLQSNSVDVLEWSFDTLYHTKEPDWLGDLLNFFSENNRLIGHGVYYSLFDARWTERQNIWLNKLKKEVKKRKYNHITEHFGFMNTENFHQGVPLPVSLHSKTLQIGKDRIFRLQEALDIPVGIENLAFSFSIDDVREQGLFLDKLIEDTDGFLILDLHNVYCQSCNFEVEMREIINSYPLDKVKEIHLSGGSWQNSVYGEKLIRRDTHDDVIPEEILSVLPWVFSQCQNLEYVIIERLGHSIHLESEKDDFLKDFAKVKKLISESDWRDQEKNNWNKKVLKLSESPLEDTLLHKEQTMLTRLLFDSADVSLIKNQEFHYFKTDTWDTEMILTAQNIIKKWNPY
SIS50444.1	Chryseobacterium shigense	MMPEADFVSAILPLLQNNSVDVLEWSFDTFYDVNEPEWLSGLLDFYAQNSRLLGHGVYYSLFDARWTARQEVWLEKLKKECSQRKYNHITEHFGFMNTENFHQGVPLPVPMHSKTLQIGKDRLKRLQDAVEVPVGVENLAFSFSMDDVKEQGVFLDQLVEDINGFLILDLHNIYCQSCNFEVGMQEIIQLYPLEKVKEIHLSGGSWQESAYAKKPIRRDTHDDRIPDEILDLLPEVLSQCQNLEYVIIERLGHTLSAESDKLSFFADFNRVKEMISNSEDRSEGKKVWKRRIEEDSKPLEDLVFYNEQTRLTKMLFDESDVAKVKDEGFQYFKPENWDEEMIITAQQIIKKWNPY
WP_076510010.1	Chryseobacterium shigense	MRRPFLGLAMMPEADFVSAILPLLQNNSVDVLEWSFDTFYDVNEPEWLSGLLDFYAQNSRLLGHGVYYSLFDARWTARQEVWLEKLKKECSQRKYNHITEHFGFMNTENFHQGVPLPVPMHSKTLQIGKDRLKRLQDAVEVPVGVENLAFSFSMDDVKEQGVFLDQLVEDINGFLILDLHNIYCQSCNFEVGMQEIIQLYPLEKVKEIHLSGGSWQESAYAKKPIRRDTHDDRIPDEILDLLPEVLSQCQNLEYVIIERLGHTLSAESDKLSFFADFNRVKEMISNSEDRSEGKKVWKRRIEEDSKPLEDLVFYNEQTRLTKMLFDESDVAKVKDEGFQYFKPENWDEEMIITAQQIIKKWNPY
OMP75747.1	Flexibacter sp. ATCC 35208	MDANILAACLPLLQESRIEAIEWSFDALYKIKEVPDWFRELLTAFSDENRLIGHGVFFSLFSGKWLPEQEAWLSHLKQTSTAFNFDHITEHFGFMTGKDFHHGAPLNIPYSASTLAIGKDRLKRIYNACGRPVGLENLAFSYSLDEVKRHGDFLDQLLEPVNGFIILDLHNLYCQLHNFDLAFEELIALYPLDKVREIHISGGSWDDVGNRTIRRDTHDEGVPAEVFQLLEMTMPKCPHLKYVVLEQLGNGLVTEASRLGFYNDFLKMQEIVHKSSNEIEANLFQMQEIVPRSNHDTGGNLLQTQEIVHKSNRETEVNPFHPLLPLPIGPAVEDLHLYEQQMELSSILETAPSNAAAMQLLQHSSLAHSDWKIEQWDPYMIETAVKIARKWKK
WP_077414519.1	unclassified Chryseobacterium	MKKPLLGISMMAEADFVSAVLPLLQNNTIDVLEWSFDTLYHANEPDWLRDLLNFYAENNRLIGHGVYYSLFDARWTERQEDWLKKLKEEVGLRKYNHITEHFGFMNTENFHQGVPLPVSLHPKTLQIGKDRLYRLQETIDIPVGIENLAFSFSIDDVKEQGIFLDKLTEDTNGFLILDLHNIYCQSCNFGVKMQEIIDLYPLDKVKEIHLSGGSWQESVYGKKQVRRDTHDDIIPEEIFSVLPSVLAKCQNLEYIITERLGHTLKTEEEKENFLNDFNKIKTIIEASDWKGRKISSWNKRQIQLPEKPVEDLALFEEQSRLTRLLFDNAGAAVIKDQDFHYFKTENWDPEMILTAQNIIKKWNPY
WP_235015951.1	Chitinophaga ginsengisegetis	MPEILSAAACNLDANILAACLPLMEESRLEAIEWSFDALFKVQQIPSWFFELLTAYSNEQRLIGHGVFFSLFSGKWRQEQADWLKQLKKVREEFRFDHVTEHFGFMTGRDFHHGAPLNIPYTKTTLDIGRDRLKRIYEACECPVGLENLAFSYSLEEVKRHGGFLEQLLEPVNGFIILDLHNLYCQIHNFNIPFEEIIRHYPLDKVREIHISGGSWENAPNSPLKKIRRDTHDDAVPAEVFHLLENTIARCPHLKYVVLEQLGNALTTEESRTIFYNDFGKMENIVKKKNTPGISSYQSFLPSSPVITGKIIEDEILHHQQLELSAILESATSCGEAIQSLTRSSLAHTAWQIENWEPYMINTAIQIAQKWKK
WP_079241387.1	Chryseobacterium	MRKPMLGVSMMAEADFVSAILPLLQNNSIKVMEWSFDTLYHTNEPDWLRDLLNFYAENDRLIGHGVYYSLFDARWTERQEEWLKKLTEEVSLRKYNHITEHFGFMNTENFHQGVPLPVSLHSKTLQIGKDRLCRLQDAVNIPVGVENLAFSFSIDDVKEQGVFLDKLTEDTNGFLILDLHNIYCQSCNFELGMQEIIDLYPLDKVKEIHLSGGSWQDSVYGKKQVRRDTHDDIIPQDILSVLPSVLTQCGNLEYVIIERLGHTMKTEQERDDFLNDFNQVKAIIETSDFNGKEKSSWNKMEVEHSEKPLEDLVLYEEQSRLTRFLYDNSNVETIKDLDFHYFKTEKWDSEMILTAKNIIKKWNPY
WP_078013439.1	Hymenobacter sp. CRA2	MHSEQAPIYSAVACNLDANILGAAFPLLEDGRIEALEWAFDTLFWAEQIPDWFTNLLDAYSQAGRLVGHGVFFSLLSGRWTPEQQQWLAHLRQVAARFGFDHITEHFGFFTGQNFHHGAPLPVPYSRTALRLGHDRLGRIAEACRCPVGLENLAFAYSLDEVKRHGTFLDELLAPVNGFVILDLHNLYCQLHNFAVPYDELLALQPLARVREIHISGGSWEDSALVPGRRIRRDTHDEAVPEEVFELLAWTLPRCPNLKYVVLEQLGSGLTTEQSRAQFRHEFERMAALVEQHRRARPATNAFRPPLPGLSSPAAEDAALHEQQRQLSHILETATSCEQAREQLQASALAHTDWRIEQWEPHMLETAVRIAQKWKE
WP_077921573.1	Spirosoma sp. 209	MTDIRSSVACNLDSHILRAALPLFADEKVGAIEWSFDALVNPRSMPDWFAELLRTYSGAGRLLGHGVFYSLFSGRWTGAQQQWLDQLKSLSRTYRFDHVTEHFGFMTGADFHKGAPMSISFTESTLAIGRDRLLRLQDACHCPVGLENLAFAYTVDEVDRHGAFLNQLIEPVNGFIILDLHNLYCQSRNFGVDPDGLLSLYPLYRVREIHLSGGSWAPSAVEPGRTIRRDTHDDAVPADVFRLLKQAIDLCPNLQYVVLEQLGAGLQTDGQQSQFQQDFLWMDHIVQAANQIRNPLINHSFLPRRFHVPDPSPLEDPLLYAQQLELSDILETSVSYAQAQQRLAGSGLARSDWAVETWQPAMLETALAIAQTWKNGFD

WP_080778923.1	Chryseobacterium phocaeense	MKKPLLGLSMMPEPDFVSAILPILQDHSVDVLEWSFDTLYDLEESEWLSGLLDFYAENNRLLGHGVYYSLFDARWTERQEIWLEKLKKEVQHRNYNHITEHFGFMNTENFHQGVPLPVPLHPKTLQIGKDRLLRLQDAVEIPVGIENLAFSFSVNDVKEQGEFLDKLIEDIDGFLILDLHNIYCQSCNFETDMLEIIRMYPLEKVKEIHLSGGSWQESAYGKKPVRRDTHDERIPEDILNILPEVLSLCPNLDYIIIERLGHTLKTEAEQRIFFDDFERVKKIISKAGYPSGQNKIWRKKKIREHSEPVEDHLLYDEQTKLTQLLFNGTDTHSIKDQDFYYFKPENWDEEMIITAQKIIKKWNPY
WP_235021434.1	Chitinophaga eiseniae	MPELISTVACNLDNNILAACLPLMEAARVEAIEWSFDALYAVENVPAWFEELLTAFSQENRLIGHGVFFSLFSGRWLPEQQQWLDHLKTVSRHYRFDHVTEHFGFMTGKDFHHGAPLNIPYTPVTLNIGRDRLKRMQDACECPVGLENLAFSYSLEEVKKHGDFLNELLEPVNGFIILDLHNLYCQAHNFDLSFDALKALYPLHRVREIHISGGSWHESEAAPGKTVRRDTHDDAVPEVVFNYLDKAIDLCPSLKYVVLEQLGTGLATEESRSAFGNDFLRMDQLLKNKNTNTAPLNTFLPQALRIPAQAREDETLYRQQLELSQILETAASFEATMKALQASSLAHSAWQIENWDPAMIETAAKIAQKWKRQTPDRRIT
SMC33782.1	Chryseobacterium sp. YR221	MMAEADFVSAILPLLQDNAVDVVEWSFDTFFAAEEPSWLKDLLNFYAGNGRLLGHGVYYSLFDARWTERQEMWLQKLKHEVQKRNYNHITEHFGFMNTENYHRGVPLPLSLHAKTLEIGKDRLYRLQDAVPIPIGVENLAFSFSVDDVREQGVFLDKLVDDIDGFLILDLHNIYCQSCNFDVSIQEIVSLYPLEKVKEIHLSGGSWQDSVYGKKMIRRDTHDDVIPEEIFSVLPSVLSQCHNLEYVIIERLGYTIHTEEEKQRFFNDFMRVKSILNDSDGMKRKKEKWMKREMLLPGPVEDSILYEEQILLTKLLFDHIDPTLIKNQNFHYFKTENWDPEMILTAQNIIKKWNPY
WP_081964927.1	Legionella norrlandica	MDLPKLGLSLMPEQDYMDASRPLFESGDVDIIEWSFDMCWGRKIPQPCLDLLTKYSSQGCLLGHGVSFSILTAKRTKRQELWLQYLQNEVNQLNYQRISEHFGFLSTNTFIQGPPLSMPFIPEMLEVGIKNLQQIKQIAKIPIGLENLGFAFCKQDVIEQGKFLTALLEAVDGFLLLDIHNIYCHMINFDMSLDEILDSLPLDRAKEMHISGGSFRSIEQNNRNIYCDSHDAAIPQDLFLLLEKNLSRFSQIDSLIFERLGNTLSNHYEAAQFREDFMTLKSLIRR
WP_082215810.1	Dyadobacter psychrophilus	MPEIYSSIACNLDAQILQASLPLFEQEKVEAIEWSFDTLFKFNEIPVWFTDLVSEFSDHSRLIGHGVYFSLFSGKWTSGQQEWLDKLQGLSLQFQFDHITEHFGFMTGEDFHKGAPISVPFTPQTLALGKDRLQRIQDACKCPVGLENLAFSYSMDEVKKHGDFLAQLVESVNGFIILDLHNIYCQVHNFNIDFDEIIKLYPLGRVREMHISGGSWDETLVGPGRQVRRDTHDEAVPEAVFELLQKAIPKCPNLKYVVMEQMGTALDTAERQAVFQSDFLRMDNIIKEIKTDFDPKITNTFGLLNRSAGTIPLEDQELHRQQLQLSHILETAGSVSEAQAMLRSSDLHDTAWEVDKWAPYMLQTAISIAQKWKNGFA
WP_082007927.1	Cellulophaga baltica	MRKRIMGNKKPKLGIAIAPSLKFLEAALPLFAGEKIEIIEWSFDTLKDAADESAWLSLLLKEYGEKNRLLGHGVFYSLLDANWSSRQENWLKKVRQETLSHKYCQISEHFGLMSSANAHSGFPLPIQLSNPVLQIGIDRLKRLQATAQVDVGIENLALTANVADILEQGEFLFKLVNPVNGFVILDLHNIYCQSENFNIDMMTIIKSYPLSLVKELHISGGSWDTDSTLSRKIRRDTHDGRIPEVILEILPEVLKICPFLAFIIFEKIEDSFLTEKDGIDFRADFQKIREIIDATAFSVTPKEKKQVAAILGPPVIDVELLQAQVALRESIALGTYHTNTKWDKDMWKVATKLYEKWNN
WP_082474654.1	Chryseobacterium sp. Leaf201	MKRQLLLGVSMMAEADFVSAILPLLESSGIDVLEWSFDTFYTVDEPQWLADLLNFYSDHKRLLGHGVYYSLFDAKWTERQDVWLQKLKAETDRRSYNHITEHFGFMNTENFHQGVPLPVPLHPELLKIGKDRLLRLQDVVEVPIGIENLAFSFSSDDVSEQGEFLYRLIEDIDGFVILDLHNIYCQASNFNMEIMELVNLYPLEKVKEIHLSGGSWENSIYTKNKRIRRDTHDDEIPQELFDILPDVISRCSHLEYIIIERLGHTIKTESDKQNFLRDFKKVKQIIDNSDPKENLQHQWKKKNRVLTDPMENLLLFDDQKNLNKFLFESQDPETIRNYAFHYFKMHNWDPEMIHTASQIVKKWNPY
WP_081145449.1	Niastella vici	MTKLLATAACNLDADMLNACYPLFLEEKVQAIEWSFDALFDHGALPQWFGELLQAYSQAKRLTGHGVFFSLFSGKWLPEQVQWLASLRDTIQRFPLEQVTEHFGFMTGKDFHEGAPLAIPYTATTLRIGRDRLRRIQDAAQCPVGLENLAFAYTPDEVKRHGDFLYKLIEPVNGFIILDLHNVYCQLKNFDLDANELLSLYPLHKVREVHISGGSWERVESEPGRQIRRDTHDDVVPAEVFLLLDKALDRCPECKYVVLEQLGNGLKSHTSRQQFYHDFVRMDNILQQKNWFRKGESQNDFMPLQLFDNGPAPEDELLFNQQRELSQILESGMSYSEAMQVLNRSSLANTPWDIEHWDPAMLETAMQIARKWKKQV
WP_084084025.1	Chryseobacterium sp. YR221	MGKPLLGLSMMAEADFVSAILPLLQDNAVDVVEWSFDTFFAAEEPSWLKDLLNFYAGNGRLLGHGVYYSLFDARWTERQEMWLQKLKHEVQKRNYNHITEHFGFMNTENYHRGVPLPLSLHAKTLEIGKDRLYRLQDAVPIPIGVENLAFSFSVDDVREQGVFLDKLVDDIDGFLILDLHNIYCQSCNFDVSIQEIVSLYPLEKVKEIHLSGGSWQDSVYGKKMIRRDTHDDVIPEEIFSVLPSVLSQCHNLEYVIIERLGYTIHTEEEKQRFFNDFMRVKSILNDSDGMKRKKEKWMKREMLLPGPVEDSILYEEQILLTKLLFDHIDPTLIKNQNFHYFKTENWDPEMILTAQNIIKKWNPY
ASE60851.1	Chryseobacterium indologenes	MMRKPLLGLSMMPEAEFVSAALPLLQNNSVEVLEWSFDTLFNTNEPDWLRDLLNFYAENNRLIGHGVYYSLFDAQWTDRQEIWLSRLKDEVSKRNYNHITEHFGFMNTENFHQGVPLPVPLHPITLQIGQDRLQRLQDAVNIPVGIENLAFSFCLDDVKEQGVFLEKLIESSGGFLILDLHNMYCQSCNFDVDIQEIIKLYPLQKVKEIHLSGGSWQESVYGKKMIRRDTHDHIIPEEIFAILPFVLSHCENLEYIIIERLGPTIKTKEEEDDFFSDFMRVKTMIDSSAGHERQGDLWIKKEFELSKYPVEDPVLYEEQSRLTKLLFDGTHVDSIKNENFHYFNTAGWDPEMIFTAQEIIKKWNPY
OUJ69468.1	Hymenobacter crusticola	MEQEPTMLASIACNLDADILSAAFPLLEEGQVEALEWSFDTLFWAEQVPSWFTELLTTYSNHNRLVGHGMFFSLLSGKWTLEQQQWLRQLHQLAAQFRFDHITEHFGFFTGQNFHYGAPLHVPYCEATLRIGQDRLRRIYEACQCPVGLENLAFSYSLEEVKQHGAFLEKLIKPVNGFLILDLHNVYCQLHNFSVAFEELVMLYPTDRVREIHISGGSWEGSVSAPGGRVRRDTHDEAVPEEVFQLLDATLPRCPNVKYVVLEQLGNGLKTEPSKIQFRQDFLRMQALVAKHRDHTSRPLTNTFLPLHPLPPSSIIEDEELYKQQLQLSHILETASSSDEAQRLLHASTLAKTAWKIEQWEPHMLQTAVDIAQKWKK
WP_088841642.1	Hymenobacter gelipurpurascens	MRTVAPPDNANERAIYSSIACNLDANILTAAFPLLEQGKVEALEWSFDALFWAEQVPDWFTELLQAYSAQHRLVGHGVYFSLLSGRWTAEQQQWLQHLKELTRRFSFDHVTEHFGFFTGQNFHAGAPLPIPYGSNTLRIGQDRLRRMQEACGCPVGLENLAFAYSLEEVKRHGEFLEQLVEPVNGFLILDLHNVFCQLHNFSVPYEELMALYPLDRVREIHISGGSWEASGQVPGLQIRRDTHDEAVPEEVFQLLYNTMPRCPNLKYVVLEQLGTGLQTEDSRAHFQSDFHRMQALVNQHRSTTSIRAVQPFLPIHSAVAGPVAEDAQLHEQQQQLSHILETAPSYEDAQRQLQASALAYTAWKVEEWEPHMLETAISIAQKWKPQPR
WP_083729470.1	Flexibacter sp. ATCC 35208	MPKVLSAVACNLDANILAACLPLLQESRIEAIEWSFDALYKIKEVPDWFRELLTAFSDENRLIGHGVFFSLFSGKWLPEQEAWLSHLKQTSTAFNFDHITEHFGFMTGKDFHHGAPLNIPYSASTLAIGKDRLKRIYNACGRPVGLENLAFSYSLDEVKRHGDFLDQLLEPVNGFIILDLHNLYCQLHNFDLAFEELIALYPLDKVREIHISGGSWDDVGNRTIRRDTHDEGVPAEVFQLLEMTMPKCPHLKYVVLEQLGNGLVTEASRLGFYNDFLKMQEIVHKSSNEIEANLFQMQEIVPRSNHDTGGNLLQTQEIVHKSNRETEVNPFHPLLPLPIGPAVEDLHLYEQQMELSSILETAPSNAAAMQLLQHSSLAHSDWKIEQWDPYMIETAVKIARKWKK
WP_083421972.1	Arsenicibacter rosenii	MAIYPALACNLDMHILQAGLPLFASEQVAALEWSFDSLYAQTELPDWFDELLLTYGQANRLVGHGVFFSIFAGRWRPEQQTWLNQLADRANRYRFDHVTEHFGFMTGASFHAGAPMSVPLNTTTLRIGQDRLHRIQQAAGCAVGLENLALAYSLDDVKRQGDFLHQLLAPVDGLLIMDLHNLYCQSHNFGVDWRELLALYPLDRIREIHLSGGSWEDSISEPGRIIRRDTHDEAVPGLLFDWLPVAIDQCPNLKYVVLEQLGRGLVTDAARLQYQADFTRIANSVAAHPKASVQGPAPAFRPETPVELPAQPLEDETLAWQQQHLTTILETAGSAHEARQQLAASPLAHSDWGTERWQPAMLETALRIAQKWTNGFSGLQ
WP_090024778.1	Chryseobacterium oleae	MKKPLLGLAMMPEAEFVSAILPLLQTQSVDVLEWSFDTFYDAEEPEWLSGLLDFYAENNRLLGHGVYYSLFDGRWANRQEIWLKKLKEEVKRRKYNHITEHFGFMNTENFHQGVPLPVPLLPKTLQIGKDRLSRLQDAVEIPVGVENLAFSFSMDDVKEQGEFLDKLVEDIDGFLILDLHNIYCQSCNFEVDMMEIINLYPLEKVKEIHLSGGSWQESAYGKKPVRRDTHDDRIPEEILNLLPEVLILCHPEYVIIERLGHTLNTEAEKQIFFDDFNRVKKIIELSDYPVDEEKIWNRQNTVHSKPVEDLLLHAEQTELTRMLFDGNTTESIKNRNFHYFKPESWDEEMITTAQQIIKKWNPY
WP_097124551.1	Spirosoma fluviale	MSRLYSSIACNLDTNSLQAALPLFESEKVQAIEWSFDTLFALGEMPDWFAELLRAYSQEGRLIGHGVFFSLFSGKWTSDQQQWLNQLKITSADFTFDHLTEHFGFMTGEDFHKGAPVSIPFTASTLAIGRDRLQRLQDAGSCPVGLENLAVAYSLDDVKRQGDFLAQLLEPINGFIILDLHNLYCQSYNFAIEITDLQALYPLDRVREIHISGGSWVPSAVNPTKQIRRDTHDDSVPAAVFHALERVMGQCPNLKYVVLEQLGTGLTTEASRRHFQEDFLRMDALVQAAHRADSPPMLNSFLPLNPPSIPEIAPENPLLYKQQTQLSAILETATDCAQAQLFLKASSLANSDWEIENWHPEMLETAIAIARKWKDGFVQ
OYV03992.1	Verrucomicrobiales bacterium VVV1	MWKGLIDMIRSGLSLMLEDEFLQAALPLFECGSVEALEWSFDVGWSLPSLPEWVTALLREYGEKGALVGHGVTFSALSGEWGDRAQNWSDQLREELNQRKYLHLSEHFGFMAGGNFHQGTPLPVPLTEKTLRLGQSRLKLLSDVAGAPVGLENLALALSPRDVADQGRFLGELLAPSDGFLVLDLHNIYCQMMNFDCTADSILASYPLERVREMHISGGSWMEAVTARGTPIRRDTHDEAVPETVFQMLDAVLPRCPGTQVVIFERLGHTLGGEGESETFRDDFLRMKSIVSSHAG
WP_099766995.1	Chryseobacterium sp. 52	MKKPLLGLSMMPEADFVSAILPILQNNAVEVLEWSFDTFYDLEEPEWLSGLLDFYAENNRLLGHGVYYSLFDARWTVRQDEWLKKLKKEVNQRKYNHITEHFGFMNTENFHQGVPLPVPLVSKTLQIGKDRLRRLQDAVEIPVGVENLAFSFSVDDVKEQGEFLNRLVEDIDGFLILDLHNIYCQSCNFEIDMQEIIRWYPLEKVKEIHLSGGSWQESAYGKKPVRRDTHDDRIPEEILNILPGVLSQCHHLEYTIIERLGHTLKTEEEKQTFLDDFKQVKEIIETSECFTGEEKKWNKTEEVYSEPVEDHLLYDEQTKLTKLLFDSNGVASVKEQEFHYFKPENWDEEMITTAQQIIRKWNPY
WP_101237823.1	Chryseobacterium sp. PMSZPI	MKKTLLGLSMMAEADFVSAILPLLETHSVDVLEWSFDTFYDVEEPDWLSGLLNFYAENNGLIGHGVYYSLFDARWTDRQEAWLEKLKKEIDKRRYKHITEHFGFMNTENFHQGVPLPVSLNPQTLQIGKDRLYRLQDILQIPVGVENLAFSFCTDDVKEQGEFLDKLVEDIDGFLILDLHNIYCQSCNFGVDMENIIRLYPLEKVKEIHLSGGSWQESAYGKSPIRRDTHDDAIPKEILNILPIVFSKCENLEYVIIERLGNTIKTDKEKKSFFDDFVQVKTILESSSRKSRKKSSWAKKDLEFSEVPLEDLILYEEQTMLTKLLFDNTDVTSIKNYDFKYFKTEKWDPEMILTAQNIIKKWNPY
ATN04960.1	Chryseobacterium indologenes	MMRKPLLGLSMMPEAEFVSAALPLLQNNSVEVLEWSFDTLFNTNEPDWLRDLLNFYAENNRLIGHGVYYSLFDAQWTERQEIWLSRLKDEVSKRNYNHITEHFGFMNTENFHQGVPLPVPLHPITLQIGQDRLQRLQDAVNIPVGIENLAFSFCLDDVKEQGVFLEKLIDNSGGFLILDLHNMYCQSCNFDVDIQEIIKLYPLQKVKEIHLSGGSWQESVYGKKMIRRDTHDHTIPEEIFAILPFVLSHCENLEYIIIERLGHTIKTKEEEGDFFSDFMRVKTIIDSSAGHERQGDLWIKKEFELSKYPVEDPVLYEEQSRLTKLLFDGTHVDSIKNENFHYFNTAGWDPEMIFTAQEIIKKWNPY
PWN62849.1	Chryseobacterium viscerum	MPGLRNGIKMRKPLLGVSMMAEADFVSAILPLLQNNSIEVLEWSFDTLYHTHEPDWLCDLLNFYAENNRLIGHGVYYSLLDARWTARQEQWLEKLKEEVQQRKYNHITEHFGFMNTDNFHQGVPLPVSLHPKTLQIGKDRLNRLQEAVDIPVGIENLAFSLSVDDVKEQGMFLDKLTEDSNGFLILDLHNIYCQSCNFGVEMQEIINLYPLDKVKEIHLSGGSWQESVYGKMPVRRDTHDDVIPEDIFSVLPAVLTQCQSLEYVIIERLGHTLKTEEEKSNFLHDFNKVKTIIEASDWKNRRENNWTKKEIQLAEKPVEDLVLYEEQSRLTRLLFDNVGAAAIKDQDFHYFKTENWDPEMILTAQNIIKKWNPY
WP_099149092.1	Flavilitoribacter nigricans	MPKIYSSIACNLDNHLLSAALPLFAEESVEAIEWSFDTLYKQRNIPDWFIELLTAFGQENRLVGHGVYFSLFSGKWTPEQEAWLQQLEKVSGHFQFDHISEHFGFMTGANFHAGAPIGIPFTRRTLQLGQDRLQRIFQACRCPVGLENLAFAYSLDEVKAHGQFLDELLEPVNGFIILDLHNLYCQLQNFTIPFEALLELYPLHRVREIHISGGSWEDSALHPRQKIRRDTHDDAVPDEVFQMLSAVVPRCPHLKFVVLEQLGIGLHTPASQEAFREDFRKLKRTVQSLSTPPPETVANDFLPKELCPLGPAPEDLSLHRQQQQLSRILETAPDLSAAREELQSSDLAGSAWKVEEWEPAMLETAMQIAQKWKNGFA
AUC75689.1	Olleya sp. Bg11-27	MWYGIMKINKPKLGLSIMPNPEFIAAALPLFESAQVEVIEWSFDTILDKKYQPEWLPLLLKEYGDNNRLLGHGVYYAPLDANWGANQDNWLQKAKLETLAYNYNHLSEHFGVMSSANAHHGFPLPFDLSNTILNIGIDRLKRLQNTVQLDIGIENLALASNVSDILKQGAFIEKLVAPINGFVILDLHNIYCQSINFDLDLLTIITSYPLHLVKEIHLSGGSWDHDHNLTKPIRRDTHDGRIPEVLLDILPEVLQRCPVLEFVIFERLGDTFQNKNDGLEFRTDFNKIQAIIDHTAFSSNARQWTLKKHDLGPPLIDLPLLNKQQELREHIRLETANNHAEWDTNMWITATKLYKKWNA
WP_104421012.1	Neolewinella xylanilytica	MTYPSLACNLDHQLLSTALPLLEAGTVSGLEWSYDAVYQYETLPEWFAALLKAFAAEGRLIGHGIFYSVCSGAWSEDQRNYLDRLAHTVEAFPFDHVTEHFGFLTGANFHRGAPIAPPLTQSTLRIGQDRLHRLHDAARCPVGLENLAFAYSADDVFRQYAFLDQLVQPIDGLLILDLHNLYCQLHNFSLDLEELLRHIPLERVREMHLSGGSWDFHHDAPTGRVRRDTHDERVPDEVFHLLHEIAPRCSNLKFVVLEQLGSALRETEAQLGFQADFDRMTSVCTAMVQQPVNDRPAPCLPNTSPITHPPLQDRALANEQQELSTILETSSTLAEVQDRLSNSSLANSAWQMENWSPYMLETAWKIARKWR
WP_170061848.1	Hymenobacter chitinivorans	MSTPAAAAEPGILATLACNLDADMLSASFPLLEQGRVEALEWSFDALFWAEQIPEWFTELLRAFAAQNRLVGHGVYFSLLSGRWTPEQQQWLSQLRKLAQQFRFDHITEHFGFFTGQNFHAGAPLPIPYTSTALRLGQDRLCRIQEACGCPVGLENLAFAYSLDEVRRHGEFLAQLVEPVNGFLILDLHNLFCQLHNFGVSYEELIQLYPLERVREIHISGGSWEESGQEPGRQVRRDTHDGAVPDEVFELLSRTLPRCPNLKFVVLEQLGNALQTEPSRAQFRRDFGRLEAMVHEHRAQAPAGGSPLFLPQRPLPTGPVAEDARLHEQQQQLTHILETAPSYDEARQRLAGSSLAHSDWQIEQWAPHMLETALRIAQKWK
MAR09898.1	Blastopirellula sp.	MLEADFAAATQPLFATGDVEVLEWSFDVGWGRSVPPWAEQLVSFYSQHQQLLGHGVSFSPLSAEWQTRQTDWLRQLGQEVARRDYQHVSEHFGFMTAGDFHQSAPLPVPRTPATLAIGRDRLARLADTCPCSVGLENLAFAFGQRDVASQGDFLDALLEPVDGFLLLDLHNIYCQSCNFDVDPWSLIESYPLDRVRELHVSGGSWTEARDGQAVRRDTHDGSVPALLFELLPRVLDRCPQVQYVILERLGGTFTNAEAEFQADFRRLRHTVETWGEWATSSSANSKHQP
WP_105041844.1	Rubritalea profundi	MPHVGLSLMPETQFLQASMPLFEAGDVGAVEWSFDTGWKNGVSEWLTSLIGTYSEAGRLTGHGVHFSLLSADWTQRQQGWLEKFKEEVTRHQYLHHTEHFGFMTAGDFHRSAPLPVPLLESTLKLGRERFAQLAAVAPCPMGLENLALALSVDDALEQHDFLEKIVSPSNGFLLLDLHNIYCQLCNFNLSPAELLDNYPFHLVREIHVSGGSWEPTASSKEPIRRDTHDEDVPAEVFSLLDYVLPRCPNLEVVFLERLGNTLHEHQFVSFRKDFHTLKSIVANG
WP_105702996.1	Chryseobacterium sp. MYb7	MDKPLLGISMMAETEFVSALLPLLQSNSVDILEWSFDTLYHSNEPGWLCDLLNFYAENDRLIGHGVYYSLFDARWTGRQEEWLKKLKEEVSLRKYNHITEHFGFMNTENFHQGVPLPVSLNPKTLEIGKDRLYRLQEAVNIPVGIENLAFSFSVDDVKEQGVFLDKLTEDTDGFLILDLHNIYCQSYNFEVEMQEIIDLYPLGKVKEIHLSGGSWQESAYGKKQVRRDTHDDVIPKEIFSVLPSVLTQCQNLEYIIIERLGHTLKTDEEKDNFLKDFNSVKGTIEASDWKREKKKNWNKKDVKIAEKPLEDLVLFEEQSRLTQLLFDNIDAAVIKDLDFHYFKTKNWDPEMILTAQNIIRKWNPY
WP_103293743.1	Chryseobacterium lactis	MGKPLVGLSMMAEAEFVSAILPLLQNEEVAVLEWSFDSLYQTDEPDWLCDLLNFFAKNNRLIGHGVYYSLLDAKWTGRQEIWLKKLKEEVCKRKYNHITEHFGFMNTENFHQGIPLPVSLHPTTLKIGKDRLSRLQDAVEIPVGIENLAFSFSIDDVKEQGVFLDRLIEDIDGFLILDLHNIYCQACNFGIDMHEIIGLYPLEKVKEIHLSGGSWQES

		VYGKRLIRRDTHDDVIPEEIFAVLPAVVSLCPNLEYIIIERLGHTLKSDEEKRSFFNDFMKVKSIIGSLEGCYLQKRDWVKKENNLLDAPVEDLLLYEEQSKLTKLLFDSAEIETVKDQDFHYFNTKNWDPEMILTAQNIIKKWNPY
PQJ27362.1	Rubritalea profundi	MPETQFLQASMPLFEAGDVGAVEWSFDTGWKNGVSEWLTSLIGTYSEAGRLTGHGVHFSLLSADWTQRQQGWLEKFKEEVTRHQYLHHTEHFGFMTAGDFHRSAPLPVPLLESTLKLGRERFAQLAAVAPCPMGLENLALALSVDDALEQHDFLEKIVSPSNGFLLLDLHNIYCQLCNFNLSPAELLDNYPFHLVREIHVSGGSWEPTASSKEPIRRDTHDEDVPAEVFSLLDYVLPRCPNLEVVFLERLGNTLHEHQFVSFRKDFHTLKSIVANG
WP_106522985.1	Taibaiella chishuiensis	MTPILSSIACNLDAGILSASLPLFAEERVEAIEWSFDTLFNYKEIPSWFLELLHTYSDAGRLVGHGVFFSLFSGRFLPEQKAWLEALRKMAGTFRFDHITEHFGFMTGKDFHSGAPLNIPYSDVTLRIGRDRLMRIQDAAQCPVGLENLAFSYSADEVKRHGEFLEMLVKPVNGFIILDLHNLYCQLHNFSMPYEQLIGLYPLHRVREIHISGGSWEDAASGQGRTIRRDTHDDAVPTVVFDLLRRTLPLCPNLKYVVMEQLGTGLQTEGSKQGFYNDFITMQEIVQAYRPQPQQAALNAFLPVQAVSIAGRPEEDEILYAQQLELSGILEQASSCREAQDTLATSGLAGTAWHTERWDADMLETAIRIAQKWKRQQA
WP_105682666.1	Chryseobacteriu m culicis	MRKPMLGVSMMAEADFVSAILPLLQNNSIEVMEWSFDTLYHAHEPDWLHDLLNFYAENDRLIGHGVYYSLFDARWTERQEEWLKKLTEEVSLRKYNHITEHFGFMNTENFHQGVPLPVSLHPQTLEIGQDRLYRLQEAVNIPVGIENLAFSFSIDDVKEQGVFLDKLTEGTNGFLILDLHNIYCQSSNFEVPIQEIINLYPLDKVKEIHLSGGSWQDSVYGDKQVRRDTHDDVIPQDILSVLPSVLTQCQNLEYIIIERLGDTVKTEEERDNFLNDFNTVSTIIETSDWKVKGKSGWSKKEMKLSEKPLENLVLYEEQSRLTKLLFDNVEVKAIKDQDFYYFKTENWDAEMILTAQNIIKKWNPY
PSK92621.1	Taibaiella chishuiensis	MPTIPVLVVAWANRPAGIQGGPDIKHLKLFVDHEQMTPILSSIACNLDAGILSASLPLFAEERVEAIEWSFDTLFNYKEIPSWFLELLHTYSDAGRLVGHGVFFSLFSGRFLPEQKAWLEALRKMAGTFRFDHITEHFGFMTGKDFHSGAPLNIPYSDVTLRIGRDRLMRIQDAAQCPVGLENLAFSYSADEVKRHGEFLEMLVKPVNGFIILDLHNLYCQLHNFSMPYEQLIGLYPLHRVREIHISGGSWEDAASGQGRTIRRDTHDDAVPTVVFDLLRRTLPLCPNLKYVVMEQLGTGLQTEGSKQGFYNDFITMQEIVQAYRPQPQQAALNAFLPVQAVSIAGRPEEDEILYAQQLELSGILEQASSCREAQDTLATSGLAGTAWHTERWDADMLETAIRIAQKWKRQQA
PSL33846.1	Dyadobacter jiangsuensis	MLLDQMAKKPPKIKRWTLVQPVEWADSYVSMSEIYSSIACNLDTHILQAALPLFEQEKVEAIEWSFDTLYRFDEVPAWFTDLVSEFSSHNRLVGHGVYFSLFSGKWSTGQQEWLDKLKKLSGEYNFDHITEHFGFMTGEDFHKGAPIGIPLTSRTLALGNDRLRRIQDACQCPVGLENLAFSYSIDEVRKHGTFLDQLVESVNGFIILDLHNLYCQVHNFNIDFADILRLYPLNKVREIHISGGSWEDTMTPPHMPVRRDTHDESVPEAVFEYLGKVIPQCPNLKFVVLEQMGTALATPASQAAFQSDFLRMDRIVKSLARKSLPIPAHLFAPLAGRANPVPLEDPDLHKQQMQLSHILETAANVSEAKAMLRSSDLHNSAWEVENWAPYMLETAMAIAQKWKNGFV
WP_106930259.1	Adhaeribacter arboris	MPEILPAIACNLDADILAAALPLFGESRIEAIEWSFDTLFRTAQIPSWFLELLKAFSNEKRLVGHGVFFSLFSGKWSPNQQNWLKQLRQLSSEFDFDHITEHFGFMTGQNFHYGAPLPIPFSKTTLAIGQDRLARIYDACHCPVGLENLAFSYSLDEVKQHGEFLEQLITPINGFIILDLHNLYCQIYNFSIAYEDIINLYPLHRVREIHISGGTWQDSNVAPARKIRRDTHDEAVPKEVFQLLDWTIDKCPNLKYVVLEQLGNALKTKESKKIFYQDFLKMEVIVQSKSNSLAPQPTNLFLPDLPNLPTTIIEDENLHQQQLQLSAILEKAPTYTEAFELLFSSSLAHSDWKIESWEPYMLETARSIAQKWKK
WP_245901666.1	Chitinophaga ginsengisoli	MPKVLSAVACNLDANILAACLPLMEESRIEAIEWSFDALYKVKEVPAWFRELLTTFSNEKRLIGHGVFFSLFSGKWLPEQENWLKHLEQTAAEFQFDHITEHFGFMTGKDFHRGAPLNIPYTTATLNIGRDRLKRIYEACKRPVGLENLAFSYSLDEVKRHGAFLEQLLEPVNGFIILDLHNLYCQLQNFNISFDEMIALYPLDRVREIHISGGSWDDSAAAPDRSIRRDTHDDTVPPEVFQLLEMTIARCPHLKYVVLEQLGNGLVTEESKRCFYDDFLQMEEIVRKNSDENDADSSDPFLPLSSLLTGTAVEDMTLYQQQLELSAILESSASYTEAMHALRQSSLANSDWRIEQWEPYMIETAVKIAQKWKK
WP_106915993.1	Chryseobacteriu m aurantiacum	MGKPLLGLSMMAEAEFVSAVLPLLQSNSVDVLEWSFDTLYHTEEPDWLGDLLDFYSENNRLIGHGVYYSLFDARWTERQSIWLDKLKEEVKKRKYNHITEHFGFMNTENFHQGVPLPVSLHPKTLQIGKDRLFRLQEALDIPVGIENLAFSFSVDDVREQGLFLDKLIEDTHGFLILDLHNIYCQSCNFEIEMQEIINSYPLDKVKEIHLSGGSWQDSVYGKKHIRRDTHDDIIPEDILSVLPWALSQCHNLEYIIIERLGHSIQQESEKNDFLEDFAKVKKLVSESDWKNQQEVHWNKKEIKLPETPLEDMLLHEEQTLLTRLLFDNADVSLIKNQEFHYFKTKNWDREMILTAQNIIKKWNPY
WP_109619450.1	Chryseobacteriu m oncorhynchi	MGKPLLGLSMMAEAEFVSAILPLLQSNSVDILEWSFDTLYNTKEPDWLEDLLNFYSENNRLIGHGVYYSLFDARWTERQDVWLDKLKEEVKKRKYNHITEHFGFMNTENFHQGIPLPVSLHHKTLEIGKDRLYRLQKAVDIPVGIENLAFSFSVDDVKEQGIFLDKLIEDTDGFLILDLHNIYCQSCNFEVEMREIINSYPLNKVKEIHLSGGSWQNSVYGKKNIRRDTHDDVIPEEILSVLPWVFSQCQNLEYVIIERLGHSIHGESEKNDFLEDFVKVKELMSASDWKDQQKNHWNKNEIKLSENPLEDMLLYEEQTVLTRLLFDSADIALIKSKEFHYFKTENWDPEMILTAQSIIKKWNPY
WP_110011639.1	Chryseobacteriu m sp. AG844	MRKPMLGVSMMAEADFLSAVLPLLQDNAIEVMEWSFDTLYHTNEPDWLRDLLNFYAENNRLIGHGVYYSLFDAIWTARQEEWLKKLKEEVSLRKYNHITEHFGFMNTENFHQGVPLPVSLGPETLQIGKDRLYRLQETVDIPVGVENLAFSFSIDDVKEQGVFLDKLIEDTDGFLIFDLHNIYCQSCNFEVGLQEIINLYPLHKIKEIHLSGGSWQESAYGKKQVRRDTHDDVIPEEILSVLPSVLTQCPNLEYIIIERLGHTLKTEEERNDFLDDFNKVRTIIETSDLKFKENKRWCKKEVKLSEKPLEDLLLYEEQSKLTRLLYDNSDVKAIQDADFHYFKTQHWDAEMILTAQNIIKKWNPY
PWW16924.1	Chryseobacteriu m sp. AG844	MLGVSMMAEADFLSAVLPLLQDNAIEVMEWSFDTLYHTNEPDWLRDLLNFYAENNRLIGHGVYYSLFDAIWTARQEEWLKKLKEEVSLRKYNHITEHFGFMNTENFHQGVPLPVSLGPETLQIGKDRLYRLQETVDIPVGVENLAFSFSIDDVKEQGVFLDKLIEDTDGFLIFDLHNIYCQSCNFEVGLQEIINLYPLHKIKEIHLSGGSWQESAYGKKQVRRDTHDDVIPEEILSVLPSVLTQCPNLEYIIIERLGHTLKTEEERNDFLDDFNKVRTIIETSDLKFKENKRWCKKEVKLSEKPLEDLLLYEEQSKLTRLLYDNSDVKAIQDADFHYFKTQHWDAEMILTAQNIIKKWNPY
WP_109709735.1	Chryseobacteriu m phosphatilyticum	MEKPLLGISMMGEAEFLSAVLPLLQNNLVDVLEWSFDTFFNAKEPDWLDSLLNFYAENNRLIGHGVYYSLFDAKWTERQEIWLEKLKEEICKRKYNHITEHFGFMNTENFHQGVPLPVSLHPKTLQIGQDRLQRLQNVINLPVGIENLAFSFCLDDVREQGVFLQQLIKDVDGFLILDLHNIYCQACNFEIGMQEIIDLYPLDKVKEIHLSGGSWEESVYGRKMIRRDTHDHAIPEDIFSILPFVLSHCKNLEYVIIERLGSTIRTADEKKKFLDDFTRVKAMIDSSVWNERQKDSWTKKEYNLSKTPVEDVMFYEEQSRLTKILFDNTAVDALKNEKFHYFNTVNWEPEMIRTAQNIIKKWNPY
WP_233259989.1	Chitinophaga sp. S165	MPKILSAVACNLDTNILAACLPLMEESRVEAIEWSFDALYKVKEVPSWFRELLTAFSDEKRLIGHGVFFSLFSGKWLPEQENWLKQLEQTAAEFRFDHITEHFGFMTGKDFHHGAPLNIPYTATTLNIGRDRLKRIYNACRVPVGLENLAFSYSLDEVKRHGAFLEQLLEPVNGFIILDLHNLYCQLRNFDIAFDEMIALYPLDRVREIHISGGSWEDSAAAPERSIRRDTHDDAVPSEVFQLLEMTIGKCPQLKYVVMEQLGNGLVTDASRRAFYDDFLQMEEIVRKNSDESNTGSSDLFLPLSSFSTDIAVEDMALYQQQLELSSILESAVSYEDAMQVLTHSSLANSDWQIEQWEPYMIETAVKIAQKWKK
PTT33357.1	Chryseobacteriu m sp. HMWF028	MSKPLLGISMMAEAEFVSAVLPLLQDNSVDVLEWSFDTFYHAHEPDWLRDLLNFYAENERLIGHGVYYSLLDARWTERKEEWLKKLKEEVTLRKYNHITEHFGFMNTENFHQGVPLPVSLHPKTLEIGKDRLYRLQEAVNIPVGIENLAFSFSIDDVKEQGVFLDKLIEDTDGFLILDLHNIYCQSCNFGVRLQEIIDLYSLDKVKEIHLSGGSWQDSVYGKKPVRRDTHDDVIPEEIFSVLSSVLTQCQNLEYVIIERLGHTLKTHEEKDNFLSDFNNVKAIIEASDWKKRGKKIRNKKEIKLSVKPLEDLILFEEQSRLTKLLFDNADAKVIKDLDFHYFKTENWDLEMILTAQNIIKKWNPY
WP_106593918.1	Dyadobacter jiangsuensis	MSEIYSSIACNLDTHILQAALPLFEQEKVEAIEWSFDTLYRFDEVPAWFTDLVSEFSSHNRLVGHGVYFSLFSGKWSTGQQEWLDKLKKLSGEYNFDHITEHFGFMTGEDFHKGAPIGIPLTSRTLALGNDRLRRIQDACQCPVGLENLAFSYSIDEVRKHGTFLDQLVESVNGFIILDLHNLYCQVHNFNIDFADILRLYPLNKVREIHISGGSWEDTMTPPHMPVRRDTHDESVPEAVFEYLGKVIPQCPNLKFVVLEQMGTALATPASQAAFQSDFLRMDRIVKSLARKSLPIPAHLFAPLAGRANPVPLEDPDLHKQQMQLSHILETAANVSEAKAMLRSSDLHNSAWEVENWAPYMLETAMAIAQKWKNGFV
RPD50464.1	Hymenobacter sediminis	MAEGRVEALEWSFDALFWAEQVPDWFSELLTTYSAANRLLGHGVYFSLLSGRWTPEQQQWLEQVRQVVAQYPLAHLTEHFGFFTGQNFHSGAPLPVPYTPATLRLGQDRLSRIYEACRCPVGLENLAFAYSLDEVKRHGEFLGRLLEPVNGFLILDLHNLYCQIHNFSVSYEELIGLYPLERVREIHISGGSWENSELAPGRKIRRDTHDEGVPEEVFQLLELTIPRCPRLKYVVLEQLGPSLKTEESRIRFRHDFGRMEEVVHRHRAGRLGYADAFFLPPQPLQTGPALEDAQLHAHQQQLSHILETAPSFAEAQRQLHASALASSDWKLEQWAPYMLETAVSIAQKWK
WP_181416153.1	Chryseobacteriu m sp. CBTAP 102	MLGVSMMAEADFISAVLPLLQNNSIDVMEWSFDTLYYANEPDWLRDLLNFYAENNRLIGHGVYYSLFDARWTERQEEWLKKLKEEVNLRKYNHITEHFGFMNTENFHQGVPLPVSLHPKTLQIGKDRLYRLQEAVNIPVGIENLAFSFSIDDVKEQGVFLDKLTEGTNGFLILDLHNIYCQSCNFELEMQKIIDLYPLDKVKEIHLSGGSWQDSVYGKKQVRRDTHDDVIPRDILFVLPSVLTQCENLEYIIIERLGNTLKTDEERDHFLDDFSKVKTIIETSELNGKEKSNWSKMKVELSEKPLEDPVLYEEQSRLTQLLYDNADVETIKDLDFYYFKTESWDAEMILTAQNIIKKWNPY
PZD74685.1	Acaryochloris thomasi RCC1774 RCC1774	MPQADFWQTAQPLLESSAVEVLEWSFDMGWGKVGLPTWVPPLLDQFSQQDRLLGHGVSYSLLSAQLDRSHWLACLEAERRQYHYQHISEHFGWLATETFRQSAPLPMPLCPETLQLGRERLQRLAEVAQVPVGLENLAFAFSQQDVREQGIFLEKLLESVDGFLLLDVHNLYCQVCNFQISATELLALYPLHRVREIHISGGSWSKGTGGQIRRDTHDQPVPEAVFELLTLALQQCLNVQFVIFERLGNTLGPASEQSQFRQDFARIQAIVQADREG
PZM82676.1	Candidatus Melainabacteria bacterium	MPTKPKLGLSLMPTEDFEQATVPLFQNNQVEAIEWSFDFSWNGAHVGEWCNKILDEFSQKGALTGHGVNLSPLSARFSQRQQKWLDNAKEEFKARKYIHASEHFGFSEAGPIAQGAPLAVPMNAESLRKGKEMLKRYADATQCPVGLENLAFAFSIEDVKRQGDFIDRLISSVDGFLLLDLHNIYCQIANFGVSELELLNSYPLAKVREIHLSGGTWTHSISGKRAAVRRDTHDDAMPQEVFNLATLALKLCPNIEFVIMERLGYTMMELEEQQEFREDFETMEEILEFCYAN
WP_199474262.1	Adhaeribacter pallidiroseus	MPEKVLAAVACNLDVDILAAALPLFAAGQVEAIEWSFDTLFRTPQMPDWFAELLQTYSQQNRLIGHGVYFSLFSGQWSVNQQNWLQHLRELSKLYTFDHITEHFGFMTGQNFHHGAPLPIPYSETTLAIGQDRLARIQDACQCPVGLENLAFSYSLDEVKQHGQFLEQLITPVNGFLILDLHNLYCQLHNFQASYNEIINLYPLDRVREIHVSGGSWESSAVEPDRKIRRDTHDEAVPEEVLELLAQIIEKCPNLKYVVLEQLGNALKSEKSKQLFYADFLKMEAILRQKNQKSRTNAGKTFLPAPFKLPEQPIEDQTLAAQQIYLSEILENAASAEEAIHLLQTSPLAHSDWQIETWEPYMIETAHNIARKWKK
WP_111952938.1	Chryseobacteriu m lathyri	MKKPLFGLSMMPEADFVSAILPILQNNSVDVLEWSFDTFYDAQEPEWLSGLLDFYSANNRLLGHGVYYSLFDAKWTERQEGWLKKLKEEFRHRKYNHITEHFGFMNTENFHQGVPLPVPLHPKTLQIGKDRLKRLQDAVEIPVGVENLAFSFSIDDVKEQGDFLDKLIEDIDGFLILDLHNIYCQSWNFEISMQEIIRLYPLEKVREIHLSGGSWQDSAYGKKPVRRDTHDESIPEEILNILPEVLSQCHNLEYIIIERLGHTLNTDSEKDAFFDDFNRVKKIIQASDSLPGNKRVWHKKNEESSVPLEDLLLYDEQTELTKMLFDGIQIHSVKKHDFHYFKPENWDEEMITTAQQIIKKWNPY
WP_114789236.1	Niabella yanshanensis	MPEIFSSIACNLDNDILAACLPLFESEKVEAIEWAFDTIFSKEVIPAWFTELLQAYSSQGRLIGHGVFFSLFSGNWSKEQENWLLKLKALSKQFRFDHITEHFGFMTGSDFHKGAPLSVPLTASTMRIGIDRLQRIQDACNCPVGLENLAFACSAEEVKRHGSFLLELIESVNGFIILDLHNFYCQSHNFNIPFDTLIKYYPLDKVREIHISGGSWEPVSVQPARTIRRDTHDDAVPETVFGFLEKTLPRCPNLKFVVLEQLGNGLTTPESRLQFQKDFYRMRAIVQSAQKNKKDGSPDPFLPPMGQRVFSTPTEDPLLGLQQQELSDIFENAESVNDAIRLLKRSSLAQSAWKIEYWDEAMLETAINIAQKWKNGFD
RBL88330.1	Chitinophaga flava	MEASRVEAIEWAFDTLFNVTEVPSWFEELLTAYSQENRLIGHGVFFSLFSGKWLPEQESWLKHLRKVSNDFHFDHVTEHFGFMTGKDFHHGAPLNIPYTTATLDIGRDRLKRICEACSCPVGLENLAFSYSPEEVKKHGAFLDQLLEPVNGFIILDLHNLYCQVHNFNLSFDDISMLYPLHRVREIHISGGSWENSIATPGRNIRRDTHDDAVPEEVFAYLEKAIDLCPHLKYVVMEQLGTGLYTDASKIAFRNDFLRMDDIIKNKNKTIAVANSFLPETLIVPPEVKEDLSLYQQQLELSNILETALSYETAMTALSSSSLAHSAWQIEQWDPAMIETAVNIAQKWKKKT
WP_123881242.1	Chryseobacteriu m carnipullorum	MSKPLLGLSMMPEADFVSAILPLLQNNSVDVLEWSFDTFYDVNEPEWLSGLLDFYAANNRLLGHGVYYSLFDARWNERQEIWLKKLKKECANRKYNHITEHFGFMNTENFHQGVPLPVPMHPLTLHIGKDRLKRLQDAVEIPVGVENLAFSFSIDDVKEQGVFLDQLVEDIEGFLILDLHNIYCQSCNFGVSMQEIIKLYPLEKVKEIHLSGGSWQESAYAKKPVRRDTHDDRIPDEIVEMLPEVLSQCQNLEYIVIERLGHTLCAESDKLFFFADFNRVKEIISKSEDRSEDKKVWNRRIGVDSKPVEDMVLYDEQIKLTKMLFDESSVAKVKNEAFQYFKPENWDEEMIMTAQQIIKKWNPY
WP_114822528.1	Chryseobacteriu m sp. KLBC 52	MRKPMLGVSMMAEPDFVSAILPLLQDNSIEVMEWSFDTLYHTNEPDWLRDLLNFYAENNRLIGHGVYYSLFDARWTERQEEWLKKLKEEVRLRKYNHITEHFGFMNTENFHQGVPLPVSLHSKTLQIGKDRLYRLQDAVSIPVGIENLAFSFSIEDVKEQGVFLDKLTEDTHGFLILDLHNIYCQSCNFELEMHEIIDLYPLDKVKEIHLSGGSWQDSVYGKKQMRRDTHDDVIPKDILSVLPSVMKKCSNLEYIIIERLGHTLKTEEERNNFLEDFNKVRTIIETSDLKLREQNGWCKEKLKFSAKPLEDLLLHEEQSKLTRLLYDNANVETIKDLDLHYFKTENWDAEMILTAQNIIKKWNPY
REC44108.1	Chryseobacteriu m pennipullorum	MKKPLLGLSMMAEAEFVSAILPLLQSNLVDVLEWSFDTFFHAEEPDWLNDLLDFYSDSRRLTGHGVYYSLFDAKWTERQEVWLDTLKKEVQTRTYNHITEHFGFMNTENFHQGVPLPVSLHPVTLQIGKDRLFRLQDAVNIPVGIENLAFSFGIDDVKEQGVFLDKLTEDTNGFLILDLHNIYCQSCNFEIPMEEIINSYPLDKVREIHLSGGSWQESAYGKKQIRRDTHDDAIPESLLDVLPSVLLKCKNLQYVIIERLGHTISTEKEKKRFTEDFQKVKKMLEAPGLLTENTTSWIKKGVVIPDVPLEDLALAEEQSRLTRLLFEGADAAAFKKENFHFFNTRNWDSEMIRTAQNIIKKWNPY
WP_110984643.1	Acaryochloris thomasi	MATTTTTRPSLGLSLMPQADFWQTAQPLLESSAVEVLEWSFDMGWGKVGLPTWVPPLLDQFSQQDRLLGHGVSYSLLSAQLDRSHWLACLEAERRQYHYQHISEHFGWLATETFRQSAPLPMPLCPETLQLGRERLQRLAEVAQVPVGLENLAFAFSQQDVREQGIFLEKLLESVDGFLLLDVHNLYCQVCNFQISATELLALYPLHRVREIHISGGSWSKGTGGQIRRDTHDQPVPEAVFELLTLALQQCLNVQFVIFERLGNTLGPASEQSQFRQDFARIQAIVQADREG
WP_115833250.1	Dyadobacter luteus	MREIYSSLACNLDANILLASIPLFEQENIEAIEWSFDALYKYDQIPPWFADLVSEYARNNRLVGHGIYFSIFSGKWLPEQQQWLNKLAVFCKEFRFDHISEHFGFMTGADFHKGAPISIPFTSKIVELGADRLARIQEICQCPVGLENLAFAYSFNEVKKQGEFLDCLIEKVNGFLILDLHNLYCQLHNFSISARELLKAYPLERVREIHISGGSWEQ

		GLASDRKIRRDTHDDAVPEEVFALLNDTIPLCPNLKFVVLEQLGTGLTTDESRAQFQTDFNRMDSIVRLHRTNNADSLDNDFIPKVPLHINTTPVEDVLLYQQQQELSHILETASDLNHAQQLLLASSLNRSAWHIDQWTPEMLHTAISIAQKWKNGF
RDV15053.1	Pontibacter diazotrophicus	MEEGRVEAIEWSFDTLYWAAQVPYWFTDLLLAYSQQSRLIGHGVFFSLLSGKWTKEQQHWLEQLKQLSSQYSFDHITEHFGFFTGQNFHSGAPLYVPYTSTTLRIGQDRLSRIYDACQCPVGLENLAFSYSLDEVKQHGEFLEKLVAPVNGFLILDLHNLYCQLHNFSITYEDLITLYPLNRVREIHISGGSWEASVLEPEKKVRRDTHDDAVPEEVFHLLELTVPKCSNLKYVVLEQLGNGLKTESSRNRFRQDFLKMEALVQKSRSSLSRQDDNLFLPVQPLQTGPVVEDDMLHDQQLQLSHILETAPSYEEARRLLFTSSLAGSDWKIENWAPHMLETAVSIAQKWKR
WP_115919746.1	Chryseobacterium rhizosphaerae	MGRPLLGLSMMAEADFVSAILPLLQDNAVDVVEWSFDTFFAAEEPSWLKDLLNFYAENGRLLGHGVYYSLFDARWTERQEMWLQKLKHEVQKRNYNHITEHFGFMNTENYHQGVPLPLSLHPKTLEIGKDRLYRLQDAVPIPIGVENLAFSFSVDDVREQGVFLDKLVDDIDGFLILDLHNIYCQSCNFDVSIQEIVSLYPLEKVKEIHLSGGSWQDSVYGKKMIRRDTHDDVIPEEIFSVLPSVLSQCRNLEYVIIERLGHTIHTEEEKQRFFNDFVRVKSILNDSDGMKRKKEKWVKREMLLPEPVEDSILYEEQIRLTKLLFDHTDPTLIKNQNFHYFKTENWDPEMILTAQNIIKKWNPY
WP_115959376.1	Chryseobacterium flavum	MERPLVGVSMMAEADFVSAVLPLLQDNAIDVLEWSFDTFFETEEPDWLCDLLNFYAGNDRLIGHGVYYSLFEAKWTDRQEIWLEKLKKEVCKRKYNHITEHFGFMNTENYHQGIPLPVPLHPKILEIGKDRLNRLQDAVQLPVGVENLAFSFSVEEVKEQGIFLDKLIEEMEGFLILDLHNIYCQACNFGVEMQEIIGSYPLHKVKEIHLSGGSWQNSVYRKKPVRRDTHDDAIPEELFAVLPSVLSQCSNLDYVIVERLGHTINTEDLKQNFLDDFIKVKTIVEKSGCQKQNTKDWFPKKNILAKPVEDAVLYEEQVRLTKLLFNHTDPIIIQHEDFHYFNTGNWDHEMILTAQNIIKKWNPY
WP_116014710.1	Chryseobacterium elymi	MGKPLLGLSMMPEADFVSAILPILQNNSVDVLEWSFDTFYDAQEPEWLSGLLDFYSEHNRLLGHGVYYSLFDARWTERQEGWLKKLKEEFRHRKYNHITEHFGFMNTENFHQGVPLPVPLHFKTLQIGKDRLKRLQDAVEIPVGVENLAFSFSIDDVKEQGEFLDKLLEDIDGFLILDLHNIYCQSWNFDISMQEIIRLYPLEKVKEIHLSGGSWQESAYGKKPVRRDTHDGSIPEEILNVLPEVLPQCHNLEYIIIERLGHTLNTGSKKEAFFHDFNRVKEITQVSDSHTKNQKVWHKKNGECVEPVEDLLLYEEQTKLTRMLFDGTDIQSVKDHGFHYFKPENWDEEMITTAQQIIKKWNPY
WP_116105833.1	Lewinella sp. IMCC34191	MVYPSVACNLDRHLLATVLPLLEEGEVAGMEWSYDAIYRYESLPDWFGQLLTAFAREGRLVGHGIFYSVCAASWTEDQKNYLDQLSSVARTYPFDHVTEHFGFLTGKDFHRGAPLSPPLTAATLRIGRDRIRRLRDAANCPVGLENLAFAYRVEDVYRQYAFLDQLVEPVDGLLILDLHNLYCQLHNFSLDLEDLLRHIPLERVREMHVSGGSWESHSSAPTGRVRRDTHDERVPNEVFHLLREIGPRCPRLKFVVLEQLGTALHDLTAQDGFRSDFQRVAAICETIHSTVPADDRDKPLSADPIPEPPLQDRQLAAEQRELSDILETSSSYSEAHDRLARSLLANSEWHVESWAPHMVETAWKISRKWR
WP_273008157.1	Chryseobacterium sp.	MRKPLLGLSMMAETEFVSAILPLLQSNSVDVLEWSFDTLYHTKEPDWLGDLLNFFSENNRLIGHGVYYSLFDARWTERQNIWLNKLKKEVKKRKYNHITEHFGFMNTDNFHQGVPLPVSLHSKTLQIGKDRIFRLQEALDIPVGIENLAFSFSIDDVREQGLFLDKLIEDTDGFLILDLHNVYCQSCNFEVEMQEIINSYPLDKVKEIHLSGGSWQDSVYGKKLIRRDTHDDVIPEEILSILPWVFSQCQNLEYVIIERLGHTIHLESEKDDFLKDFAKVEKLISESDWRNQKKINWNKKELKLSESPLEDTLLHKEQTMLTRLLFDSADVSLIKNQEFHYFKTDTWDPEMILTAQNIIKKWNPY
WP_115969000.1	Chryseobacterium pennae	MGKPLLGLSMMAEAEFLSAVLPLLQSNSIEVLEWSFDTFYNTEEPDWLSDLLNFYSENNRLIGHGVYYSLFDARWTKRQELWLQKLKEEFHKRKYNHITEHFGFMNTENFHQGVPLPVSLHSKTLAIGKDRLYRLQDAVQVPVGIENLAFSFSLDDVLEQGVFLDKLTDDTDGFLILDLHNIYCQSCNFEVQMQDIIKLYPLDKVKEIHLSGGSWQESAYGKKWVRRDTHDDVIPEDILSVLPSVLSQCRNLEYIIIERLGHTINTEEKKKSFLDDFAKVKMLIDSSDWHAQNKENRNRKEIQFPEHPLEDRILHEEQTLLTKLLFDNVEPESVNHHTFQYFKTEKWDPEMIITARNIIKKWNPY
HAY78875.1	Planctomycetaceae bacterium	MLEADFAAATQPLFATGDVEVLEWSFDVGWGRSVPPWAEQLVSFYSQHQQLLGHGVSFSPLSAEWQTRQTDWLRQLGQEVARRDYQHVSEHFGFMTAGDFHQSAPLPVPRTPATLAIGRDRLARLADTCPCSVGLENLAFAFGQRDVASQGDFLDALLEPVDGFLLLDLHNIYCQACNFDVDPWSLIESYPLDRVRELHVSGGSWTEARDGQAVRRDTHDGSVPALLFELLPRVLDRCPQVQYVILERLGGTFTNAEAEFQADFRRLRHTVETWGEWATSSSANSKHQP
RFM37115.1	Chitinophaga silvisoli	MEESRVEAIEWSFDALYKVKQVPDWFRELLTAFSDENRLIGHGVFFSLFSGKWLPEQQAWLDHLKHTSTEFSFDHITEHFGFMTGKDFHHGAPLNIPYSAATLSIGRDRLKRIYNACGRPVGLENLAFSYSLDEVKRHGAFLDQLLAPVNGFIILDLHNLYCQLRNFDLAFDDLISLYPLDKVREIHISGGSWDDSAASPDRSIRRDTHDEAVPPEVFQLLEWTLPKCPHLKYVVLEQLGNGLVSQESKAAFYNDFLHMQEIVSQSNNKYSDNSFLPLLPTTTGPAVEDLALYQQQLELSAILETAASYDSAMHLLAQSSLANSDWRIEQWEPYMIETAVKIAGKWKK
WP_291873038.1	Chryseobacterium sp.	MKKPLLGLAMMPEADFVSAILPLLQTQSVDVLEWSFDTFYDLEEPEWLSGLLDFYSDNNRLLGHGVYYSLFDARWMERQEIWLKKLKEEVKRRKYNHITEHFGFMNTENFHQGVPLPVPLLPKTLQVGKDRLSRLQDAVEIPVGVENLAFSFSMDDVKEQGEFLDKLVEDIDGFLILDLHNIYCQSCNFEVDMREIINLYPLEKVKEIHLSGGSWQESAYGKKPVRRDTHDDRIPEEILNLLPEVLIRCHPEYVIIERLGHTLHTEAEKQIFFDDFNRVKKIIELSDYPVDEEKIWNRKKTVHSKPVEDLLLHAEQTELTRLLFDGNTTESIKNSDFHYFKPESWDEEMITTAQQIIKKWNPY
HCN49489.1	Chryseobacterium sp.	MEKPLLGLSMMAEAEFVSAILPLLQNNSVEVLEWSFDTFYNAEEPDWLNELLQFYSENNRLIGHGVYYSLFDARWTERQEIWLKKLKEEVRKRNYNHITEHFGFMNTENFHQGVPLPVSLHPKTLQIGRDRLYRLQDAVDVPVGIENLAFSFSIDDVKEQGMFLDQLVEDTNGFLILDLHNIYCQSYNFEVNIQDIISLYPLDRVREIHLSGGSWQESVYGKKPIRRDTHDDRIPKELLAILPSVLSQCQNLEYVIIERLGHTIKTEEQKKYFFDDFNKVKAIIEVSDTRSRKVNKWTGKEAVHTDHPEEDLALYEEQSRLTRLLFDQAGTEGIKEEKFHYFNTESWDPEMILTAQNIIKKWNPY
WP_276729926.1	Chryseobacterium carnipullorum	MSKPLLGLSMMPEADFVSAILPLLQNNSVDVLEWSFDTFYDVIEPEWLSGLLDFYADNNRLLGHGVYYSLFDARWNERQGIWLKKLKKECANRKYNHITEHFGFMNTENFHQGVPLPVPMHPLTLQIGKDRLKRLQDAVEIPVGVENLAFSFSIDDVKEQGVFLDQLVEDIEGFLILDLHNIYCQSCNFGVSMQEIIKLYPLEKVKEIHLSGGSWQESAYAKKPVRRDTHDDRIPDEIVEMLPEVLSQCQNLEYIVIERLGHTLSAESDKLFFFADFNRVKEIISKSEDRSEDKKVWNRRIGVDSKPVEDMVLYDEQIKLTKMLFDESSVAKVKNEAFQYFKPENWDEEMIMTAQQIIKKWNPY
WP_118972670.1	Taibaiella koreensis	MEYEPLRSTIACNLDAGILSASLPLFEEARVEAIEWAFDTLFRLETIPVWFEELLEAFSKEKRLIGHGVFFSLFSGRWQPEQQRWLDQLRTLCRKYHFDHITEHFGFMTGADFHHGAPLGIPFTAETLALGRDRLARIYDACQCPVGLENLAFSYSLEEVERHGAFLEELLQPLNGFIILDLHNLYCQLHNFSLDFDTLIKRYPLHRVREIHISGGSWEPSALFEGKSVRRDTHDDTVPEEVFVLLEKTIPLCPCLKYVVMEQLGTGLATEPSRQAFRNDFIRMQQIVSAATAQPAAFLPEAFLPPDILPAGPVVESATLHNQQLELSAILESSAGYEDARQRLAHSSLAGTGWQVEAWQPHMLETAVHIAQKWRKKNT
WP_119445210.1	Hymenobacter oligotrophus	MQAAPAIYASVACNLDADLLAAAFPLLEEGRVEALEWSFDALYWAEQVPEWFTALLHTYAEAGRLVGHGVFFSLLSGKWSPEQQQWLAHLGQLAEQFRFDHVTEHFGFFTGQNFHHGAPLPIPYTRHALRIGQDRLRRIYEACRCPVGLENLAFAYTPDEVKRHGAFLEELLLPVNGFLILDLHNLYCQLHNFGLPYDELIALYPLERVREIHISGGSWHDSALAPGRRIRRDTHDDAVPAEVFQLLEWTMPRCPNLKFVVLEQLGHALHAEASRAQFRADFARMESLVAHHRATWRPQPANAFRPLQAWLPGPAAEDEHLHQQQRQLSDILENAGSYTEAQHLLQTSSLAHSDWCIEQWEPHMLETALHIARKWKK
AXY78851.1	Paraflavitalea soli	MEESRIEALEWSFDTLYKVQEIPTWFQELLTAFSKEKRLIGHGVFFSLFSGKWLPEQEAWLKALQQTCSEFQFDHITEHFGFMTGKNFHQGAPLNIPYTPTTLAIGQDRLKRIYAACQCPVGLENLAFSYSLDEVKRHGRFLDKLLEPVNGFIILDLHNLWCQLHNFSIALEELIGLYPLDRVREIHISGGSWDHSQIEKGRTIRRDTHDDAVPAAVFQLLETVIPACPHLKYVVLEQLGNGLESIESKQQFYADFLEMESIIRRQSQPAYLTNLASFLPLSPIVMGPPVEDEGLYEQQAALSTILETSHSYEEARTLLGQSSLACSEWQVENWDPAMIETAMKIAQKWKAGFVGFIS
RKE81000.1	Chryseobacterium sp. AG363	MMAEPDFVSAILPLLHNNSIEVMEWSFDTLYHTNEPDWLRDLLNFYAENNRLIGHGVYYSLFDARWTARQEEWLKKLKEEVSLRKYNHITEHFGFMNTENFHQGVPLPVSLHSKILQIGKDRLYRLQDAVNIPVGIENLAFSFSIEDVKEQGIFLDKLTEDTNGFLILDLHNIYCQSCNFELEMQQIIDLYPLDKVKEIHLSGGSWQDSVYRKKQVRRDTHDDVIPKDILSVLPSVMKKCPNLEYIVIERLGHTLKTEEKRNSFMEDFTKVRTIIETSDLKLKQQNGWCKEEIKLPAKPLEDLLLYEEQSRLTRLLYENANVENIKDLDFHYFKTENWDAEMILTAQNIIKKWNPY
WP_121487547.1	Chryseobacterium sp. 7	MNKPLLGISMMAETDFVSAVLPLLQSNSVDVLEWSFDTLYHSHEPDWLRDLLNFYAENNRLIGHGVYYSLFDARWTGRQEEWLKKLKEEVSLRKYNHITEHFGFMNIENFHQGVPLPVSLNPKTLEIGKDRLYRLQEAVNIPVGIENLAFSFSVDDVKEQSVFLDKLTEDTNGFLILDLHNIYCQSYNFEVEMQEIIDLYPLEKVKEIHLSGGSWQESAYGKKQVRRDTHDDVIPKEIFSVLPSVLTQCQNLEYVIIERLGHTLKTEEEKDDFLNDFNQVKGIIEASDWKENEKKSWNKKEIKLSEKPLEDLVLFEEQSRLTRLLFDNVDAETIKDQDFHYFKTEKWDPEMILTAQNIIKKWNPY
GDY10763.1	Planctomycetia bacterium	MTAPQVGLSLMLEPDFVAAALPLLQAGRVDVLEWSFDIGWSQAAMPEWADELLDHFASQDRLLGHGVKYSLLSADESPARDAWLERLSAECRRRHYRHVSEHFGFMAAGRFHEGAPLPVPLTEFTVRLGRERLQRLADAARCPVGLENLAFAFGPRDVQDQGRFLDELLTPLDGFLLLDLHNIDCQCCNFDVSGAELLATYPLHRVREIHVSGGSWSSVKVPSPKQLIRRDTHDAAVPREVFDLVRWAIPRCPNLEAVILEQLGGTLLEANAAEQFRTDFQTLRQVVSEVSNDRV
WP_119080626.1	Chitinophaga alhagiae	MPRLLSTVACNLDPHLLRACLPLFESGEADAIEWSFDALYRLDALPDWFHGLINAFSEGNRLIGHGIFFSLFSGKWHPEQETWLRELSAVCRKYRFDHITEHFGFMTGQDFHHGAPLNIPYTDAALRIGRDRLLRIADACGRPVGIENLAFSWSVDEVRQHGDFLDQLLEPVNGFIILDLHNLYCQVANFNLPFETLAALYPLHRVREIHISGGSWELSEDQSRSIRRDTHDDSVPEEVFRYLENIIPRCPHLRYLVMEQLGSGLETEDSKNAFRADFRRMAAIARQWNLTKGDEILHPFSPPANPPQGAPLEDLALYQQQTELSSILENAPSFEAATRALQASSLAQSHWQVERWSPFMLETAMHIAQKWMKKN
WP_120230832.1	Chryseobacterium sp. AG363	MRKPILGVSMMAEPDFVSAILPLLHNNSIEVMEWSFDTLYHTNEPDWLRDLLNFYAENNRLIGHGVYYSLFDARWTARQEEWLKKLKEEVSLRKYNHITEHFGFMNTENFHQGVPLPVSLHSKILQIGKDRLYRLQDAVNIPVGIENLAFSFSIEDVKEQGIFLDKLTEDTNGFLILDLHNIYCQSCNFELEMQQIIDLYPLDKVKEIHLSGGSWQDSVYRKKQVRRDTHDDVIPKDILSVLPSVMKKCPNLEYIVIERLGHTLKTEEKRNSFMEDFTKVRTIIETSDLKLKQQNGWCKEEIKLPAKPLEDLLLYEEQSRLTRLLYENANVENIKDLDFHYFKTENWDAEMILTAQNIIKKWNPY
WP_123278455.1	Chryseobacterium cucumeris	MRKPILGVSMMAEPDFVSAILPLLHNNSIEVMEWSFDTLYHTNEPDWLRDLLNFYAENNRLIGHGVYYSLFDARWTARQEEWLKKLKEEVSLRKYNHITEHFGFMNTENFHQGVPLPVSLHSKILQIGKDRLYRLQDAVNIPVGIENLAFSFSIEDVKEQGVFLDKLTEDTNGFLILDLHNIYCQSCNFGLEMQQIIDLYPLDKVKEIHLSGGSWQDSVYRKKQVRRDTHDDVIPKDILSVLPSVMKKCPNLEYIIIERLGHTLKTEEERNYFLADFNKVRTIIETSDLKLKQQNGWCKEEIKLPAKPLEDLLLYEEQSRLTRLLYENANVENIKDLDFHYFKTENWDAEMILTAQNIIKKWNPY
WP_123274716.1	Chryseobacterium sp. G0240	MRKPFLGISMMAEPDFLAAILPLLQDGSIDVLEWSFDTFYHAEEPDWLANLLNFYSQKDRLLGHGVYYSLFDARWTDRQEKWLEKLQQEVQQRRYAHITEHFGFMNTENFHQGVPLPVSLHPKTLEIGKDRLLRLQEVVDIPVGIENLAFSFSIDDVKEQGVFLEKLTADINGFLILDLHNIYCQACNFGVDMEELIRLYPLEKVREVHLSGGSWQTSSYGKRQIRRDTHDDSIPEEILTVLPSVLSQCKNLEYVIIERLGHTIQTEKEKKDFFDDFVRVKTIVGALAWKDYDKTCWTKKNIRLTGKPLEDEALYQEQTKLTKLLFDNVESGAFKEQDFQFFKTENWDPEMIRTAQAIIKKWNPY
WP_123319418.1	Chryseobacterium sp. BIGb0232	MGKPLLGLSMMAEAEFLSAVLPLLQSNSIEVLEWSFDTFYNTEEPDWLSDLLNFYSENNRLIGHGVYYSLFDARWTERQELWLQKLKEEFRKRKYNHITEHFGFMNTENFHQGVPLPVSLHSKTLAIGKDRLYRLQDAVQVPVGIENLAFSFSLDDVLEQGVFLDKLTDDTDGFLILDLHNIYCQSCNFKVEMQDIIKLYPLDKVKEIHLSGGSWQESAYGKKRVRRDSHDDVIPEDILSVLPSVLSQCRNLEYIIIERLGHTINTEEKKKSFLDDFAKVKMLIDSSDWQTQNKENRNRKEIQFPEHPLEDRILHEEQTLLTKLLFDNVEPESFNHHAFQYFKTEKWDPEMIITARNIIKKWNPY
AYY86288.1	Chryseobacterium indologenes	MMRKPLLGLSMMPEAEFVSAALPLLQNNSVEVLEWSFDTLFNANEPDWLRDLLNFYAENNRLIGHGVYYSLFDAQWTERQEIWLSRLKDEVSKRNYNHITEHFGFMNTENFHQGVPLPVPLHPITLQIGQDRLQRLQDAVNIPVGIENLAFSFCLDDVKEQGVFLEKLIDNSGGFLILDLHNMYCQSCNFDVDIQEIIKLYPLQKVKEIHLSGGSWQESVYGKKMIRRDTHDHTIPEEIFAILPFVLSHCENLEYIIIERLGHTIKTKEEEGDFFSDFMRVKTIIDSSAGHERQGDLWIKKEFELSKYPVEDPVLYEEQSRLTKLLFDGTHVDSIKNENFHYFNTAGWDPEMIFTAQEIIKKWNPY
WP_123899531.1	Chryseobacterium bernardetii	MGKPLLGLSMMAEEEFLSAVLPLLQGNSVEVLEWSFDTFYNSHEPDWLSDLLNFYAENDRLVGHGVYYSLFDARWTERQEIWLKKLKEEFRKRKYNHITEHFGFMNTENFHQGVPLPVSLHPEIVEIGKDRLYRLQDAVQVPVGIENLAFSFSLNDVMEQGIFLDRLTEGIEGFLILDLHNIYCQSCNFEVEMSEIIKLYPLDKVKEIHLSGGSWQESIYGKKLIRRDTHDDEIPEAILSVLPSVLLQCKNLEYVIIERLGNTINTREKKKRFLDDFLKVKALIDSSGLEIQNKNSWAKKELHFPESPLENIILHEEQTLLTKLLFDNVEAECIKNHKFHYFKTENWSPEMILTAQNIIKKWNPIKNDNPLVTTNQII
WP_123841941.1	Chryseobacterium arthrosphaerae	MKKPLLGLSMMPEADFVSAILPLLQSNSVEVLEWSFDTFYEAKEPEWLNELLNFYSENQRLIGHGVYYSLFDALWTERQENWLKKLKEEVRKRKYSHITEHFGFMNTENFHQGVPLPVSLHPKTLLIGKDRLYRLQDAVELPVGIENLAFSFSIEDVKEQGVFLDNLIADINGFLILDLHNIYCQACNFEVEMEDIIHLYPLDKVREIHLSGGSWQESVYARKMIRRDTHDDLIPEEIFSVLPAVITQCNNLEYVIIERLGHTIKTDQEKRAFLDDFTRVRSIIDDSEIKTGKKIRWGQKETGFPNSPVEDDALYEEQSRLTKLLFDSSGAEMIKEQRFHYFKTENWDPDMILTAQNIIKKWNPY
WP_124634758.1	Taibaiella sp. KBW10	MDTPKILPTVACNLDADILSASLPLLEEGRVAAIEWSFDTLFKRNHILPEWFQELLETYSQANRLIGHGVFFSLFSGRLLPEQKTWLQHLETVSKQYTFDHITEHFGFMTGADFHKGAPMSIPYSKSTVAIGRDRLSRIQQACQCPVGLENLAFSYSLEEVKKHGDFLNELIEPLNGFIILDLHNFYCQLHNFNIPFDTLIALYPLDRVREIHISGGSWQQSIVNEQKQIRRDTHDGAVPQMVFELLQKALPLCPNLKYVVMEQLGIGLQTEADRLQFYRDFLQMEYLVMQYNEGHVYQPHNTFMPSGSLDTTRVVEDEYLYAEQQILSFILEHAKDHEDALHQMQQTALRQSSWDIEHWQPEMIDAALRIAQKWK
WP_123939944.1	Chryseobacterium sp. G0186	MGKPLLGISMMAEAEFVSAILPLLQTNLVDVLEWSFDTMNTDEPDWLSNLLDFYAGNNRLIGHGVYYSLFDARWTERQRLWLEKLKNEVHRRNYNHITEHFGFMNTSNFHQGVPLPVSLHPKTLQIGKDRLYRLQEAVQIPVGIENLAFSFSIDDVMEQGVFLDKLTEDTNGFLILDLHNIYCQACNFEVEIEDIIKLYPLDKVREVHLSGGSWQ

		ESIYGKKMIRRDTHDDAIPEDILTVFSSVLSQCKNLQYVIVERLGHTIDTEEKKENFLNDFSKVKILIEASDQCIQQKKNWTKKEIRFPKAPLEDLVLYEEQVRLTKLLFDRAEVASIRNQEFYYFKTENWDQEMILTAQNIIKKWNPY
RTL44676.1	Candidatus Melainabacteria bacterium	MKPAQLKPMLGVSLMPSDDFRKASRSLFESNQIQVIEWSFDFSWLGNEIEPWCTEIIDRFSSSNNLIGHGVTLSPLSANFSKRQENWLARVRDEFKTRNYVHASEHFGFSEAGPISHGAPIAVPMTAESLRTGTEMLKRYAEATQCPVGLENLAFAFSLDDVKRQGEFIDRLISAVDGFLVLDVHNLYCQVANFDVPITDLLNSYPLTKVREMHLAGGSWSESESRKRFAVRRDTHDDSVPQDVFNFAALGLKLCPNIEFVILERLGWTMLDEEAQAGFREDFETLAEILDATYSQSPLLNAEALFSNPDWRPVLRESFLPGSTEKQCLPEYQNRLLELLSSNKPSAEIFELLKSDPAFENYQNYVSNFDPDMVAVACELMRKWARRTEC
WP_123907925.1	Chryseobacterium sp. G0162	MGRPLLGLSMMAESEFISAVLPLLQSNSIEVLEWSFDTFYNTEEPVWLSELLNFYSENNRLIGHGVYYSLFDALWTERQELWLEKLKEEFHKRKYKHITEHFGFMNTENFHQGVPLPVSLHSKTLEIGKDRLYRLQDAVEVPVGIENLAFAFSLDDVTEQGVFLDKLTEDIDGFLILDLHNIYCQSCNFNVDMRDIIKLYPLNKVREIHLSGGSWQESVYSKTPVRRDTHDDAIPKEILSILSSVVFQCKNLEYIIIERLGHTINTEEKKKNFLDDFAQVRTLIDSSDWEGADKDLWNKKKVQFQKNPLEDMILHEEQTLLTKLLFDNAGVASIKNHEFHYFKTKDWDPEMILTAQNIIKKWNPY
WP_124531036.1	Chryseobacterium sp. KBW03	MRKPLLGVSMMAEADFVSAILPLLQNNSIEILEWSFDTLYYTHEPDWLCNLLNFYAENDRLIGHGVYYSLLDARWTARQEKWLEKLKEEVQQRKYNHITEHFGFMNTDNFHQGVPLPVSLHPKTLQIGKDRLYRLQEAVDIPVGIENLAFSFSVDDVKEQGVFLDKLTEDSNGFLILDLHNIYCQSCNFGVEMQEIINLYPLDKVKEIHLSGGSWQESVYGKMPVRRDTHDDVIPEDIFSVLPAVLTQCQNLEYVIIERLGHTLKTEEERSNFLYDFNKVKTIIEASDWKNRRENSWTKKEIQLTTEKPVEDLVLYEEQSRLTRLLFDNVGVAVIKDQDFHYFKTENWDPEMILTAQNIIKKWNPY
WP_123870988.1	Chryseobacterium bernardetii	MGKPLLGLSMMAEAEFLSAVLPLLQGNSVEVLEWSFDTFYNSQEPNWLSDLLNFYAENDRLVGHGVYYSLFDARWTERQEIWLKKLKEEFRKRKYNHITEHFGFMNTENFHQGVPLPVSLHPEIVEIGKDRLYRLQDAVQVPVGIENLAFSFSLNDVMEQGIFLDRLTEDIEGFLILDLHNIYCQSCNFEVEMSEIIKLYPLDKVKEIHLSGGSWQESIYGKKLIRRDTHDDEIPEAILSVLPSVLLQCKNLEYVIIERLGNTINTREKKKRFLDDFLKVKALIDSSGLEIQNKNSWTKKELHFPESPLENIILHEEQTLLTKLLFDNVEAECIKNHKFHYFKTENWSPEMILTAQNIIKKWNPY
WP_123860487.1	Chryseobacterium nakagawai	MGRPLLGLSMMAESEFISAVLPLLQSNSIEVLEWSFDTFYNTEEPVWLSELLNFYSENNRLVGHGVYYSLFDALWTERQELWLEKLKEEFHKRKYKHITEHFGFMNTENFHQGVPLPVSLHSKTLEIGKDRLYRLQDAVEVPVGIENLAFAFSLDDVTEQGVFLDKLTENIDGFLILDLHNIYCQSCNFNVDMQDIIKLYPLDKVREIHLSGGSWQESVYSKTPVRRDTHDDAIPKEILSILSSVVFQCKNLEYIIIERLGHTINTEEKKKSFLDDFEQVRTLIDSSGWERENEDLWNKKKVQFQKAPLEDMILHEEQTLLTKLLFDNAGVASIKNHEFHYFRTKDWDPEMILTAQNIIKKWNPY
RTZ46797.1	Chryseobacterium arthrosphaerae	MKKPLLGLSMMPEADFVSAILPLLQSNSVEVLEWSFDTFYEEKEPEWLDELLNFYSENQRLIGHGVYYSLFDALWTERQENWLKKLKEEVRKRKYSHITEHFGFMNTENFHQGVPLPVPLHPKTLLIGKDRLYRLQDAVKLPVGIENLAFSFSIEDVKEQGVFLDNLIADINGFLILDLHNIYCQACNFEVEMEDIIRLYPLDKVREIHLSGGSWQESVYARKMIRRDTHDDLIPEEIFSVLPSVITQCNNLEYVIIERLGHTIKTDQEKRAFLDDFTRVRRIIDDSEIKTGKKIRWGQKETGFPNSPVEDDALYEEQSRLTKLLFDSSGAEMIKEQRFHYFKTENWDPDMILTAQNIIKKWNPY
WP_126650399.1	Chryseobacterium aureum	MSKPLLGISMMAEADFVSAVLPLLQTDSVDVLEWSFDTLYHSYEPDWLHDLLNFYAENNRLIGHGVYYSLFDGRWTGRQEEWLKKLQEEVSLRKYNHITEHFGFMNTENFHQGVPLPVSLHPQTLEIGKDRLYRLQEAVNIPVGIENLAFSFSVDDVKEQGVFLDKLTENTDSFLILDLHNIYCQSCNFQVGLQELIELYPLDKVKEIHLSGGSWQESAYGKKQVRRDTHDDVIPDEIFSVIPYVLMKCQNLEYVIIERLGHTLKTQEEKDNFVMDFNEVKNIIEASAWKKKEKKSWNKKEVRLSEKPLEDLVLYEEQSRLTRLLFDNIDAATFKGVDFHYFKTENWDPEMIVTAQNIIKKWNPY
NSL90190.1	Chitinophaga solisilvae	MEASKIEAIEWSFDALYKVKDIPDWFVELLTAFSNENRLIGHGIFFSLFSGRWRQEQEQWLQHLRKTSTHFRFDHITEHFGFMTGKDFHHGAPLNIPYTKSTLNIGVDRLKRIHDACRCPVGLENLAFSYSMDEVKRHGAFLDELLEPVNGFIILDLHNLYCQLHNFDITFGEMIILYPLHKVREIHISGGSWEASPAAPERMVRRDTHDDAVPEEVFQLLQKTIPLCPNLQYVVLEQLGTGLETDTSRTLFRQDFGRMQRIVHEQQPQTGSCPVNSFMPDNLLISAVPAADETLFQQQLELSAILESASSCEAAVHALQHSSLAHSDWLIEQWQPYMIETAVSIAQKWKKI
WP_129036615.1	Chryseobacterium sp. CH21	MSKPLLGISMMAEADFVSALLPLLQSNSVDVLEWSFDTLYHSNEPGWLRDLLNFYAENNRLIGHGVYYSLFDARWTSRQEEWLRKLKEEVALRKYNHITEHFGFMNTENFHQGVPLPVSLNPKTLEIGKDRLYRLQEVMNIPVGIENLAFSFSVDDVKEQGVFLDELTEDTNGFLILDLHNIYCQSCNFEIGLQEIIDLYPLDKVKEIHLSGGSWQESAYGKKQVRRDTHDDVIPDDIFAVLPSVLTQCHNLEYIIIERLGHTLKTDNEKDDFLNDFDKVKMIIAASDWKEKEKKNWNAKDIKLPEEPLEDLVLFEEQSKITQLLFENIDVAVIKDQDFHYFKTENWDAEMILTAQNIIKKWNPY
WP_126247406.1	Chitinophaga rhizosphaerae	MPRLLSTVACNLDPQLLGACLPLFESGEADAIEWSFDALYRLHELPEWFHGLIGAFSEGDRLIGHGIFFSLFSGKWHPEQQAWLKELSSICDRYRFDHITEHFGFMTGQDFHHGAPLNIPYTAATLRIGQDRLQRIAEACGRPVGLENLAFSYSADEVRRHGDFLDNLLAPVNGFLILDLHNVYCQVENFDIDFDTLAAMYPLHRVREIHISGGSWEPSADGSQNIRRDTHDGAVPEKVFQYLERIIPQCPHLRYVVMEQLGAGLQTEASKSAFRADFRRMADIAGTWNNPASDESIHPFTPPASRLQSAPAEDPDLHRQQTELSSILENAASLEDALRSLETSSLAQSDWQVEQWSPYMVETAMRIARKWMKKD
WP_126693540.1	Hymenobacter gummosus	METAPRICSAVACNLDADILAAVYPLLEEGRVEALEWAFDALYWAEQVPEWFEALLQAYAQAGRLIGHGVFFSLLSGRWTPEQQQWLQDLRQVASRYQFDHITEHFGFFTGQNFHHGAPLPIPYSAAALRIGQDRLLRIADACRCPVGLENLAFAYALDEVRRHGEFLDQLLQPVNGFIILDLHNLYCQLHNFEVPYEELIALYPLDRVREIHISGGSWEDSALTPARRIRRDTHDEAVPDEVFELLRLTLPRCPNLKYVVLEQLGPGLRTEASRLRFQADFGRMEALVQPQRAGLAAGPGNAFRPPLAPLTAPAAEDGQLHAQQRQLSDILETAGSYEEVQRRLHASALAGTDWRVESWAPHMLETAVNIARKWKQ
WP_129020704.1	Edaphocola flava	MSRTILPTIACNLDADILSASLPLLEEGVVDALEWSFDALFRQDHVIPEWFHDLLTAYSNEGRLIGHGVFFSLFSGRLLPEQAAWLQRLKQLSRQYRFDHITEHFGFMTGSDFHKGAPMSVPYTSGTVAIGRDRLQRIQDAAQCPVGLENLAFAYSLDEVKRQGAFLEQLIEPVNGFIILDLHNVYCQLHNFNIPFEVLIQAYPLDRVREIHISGGSWETAVTDQSRSIRRDTHDGRVPESVFELLEHTLPLCPQVKYVVMEQLGTGLQTEAAKISFRKDFQQMQKITEQFLEHHSRMDAVPNNFLPEYSLPPGQVIEDEYLYGEQQILSYILEHAAGHAEAMEQLQHTALRNSVWGIEKWQPDMLETAIRIAQKWK
QBA23550.1	Chryseobacterium indologenes	MRKPMLGVSMMAEADFLSAVLPLLQDNSIEVMEWSFDTLYHTNEPDWLRDLLNFYAENNRLIGHGVYYSLFDARWTARQEEWLNKLKEEVSLRKYNHITEHFGFMNTENFHQGVPLPVSLHSKTLQIGKDRLFRLQEAVNIPVGVENLAFSFSIDDVKEQGVFLDKLIDDTDGFLILDLHNIYCQSCNFEVGLQEIINQYPLDKIKEIHLSGGSWQESAYGKKQVRRDTHDDVIPEEILSVLSSVLTQCPNLEYIIIERLGHTLKTEEERNDFLDDFNKVRTIIETSDLKFKEKKRWCKKEVKLSEKPLEDLLLYEEQSELTRLLYDNADVKAIQDADFQYFKTQHWDAEMILTAQNIIKKWNPY
RYD58560.1	Sphingobacteriales bacterium	MHEIFSSIACNLDNDMLSAALPLLQEERVEAIEWSFDALYDHRQIPPWFDELLHAFSNEGRLVGHGVFFSLFSGGWSVGQQAWLDHLKMLSRTFRFDHITEHFGFMTGRDFHSGAPLNIPYTNTNLQIGQSRLKKIYDACECPVGLENLAFSYTLDEVKRHGAFLEELLRPINGFIILDLHNLYCQLHNFSIPFDELIDLYPLDRVREIHISGGSWEGSEIEPGRQVRRDTHDDAVPEEVFYLLKRTIDKCPHLKFVVMEQLGNALKTTESRQQFYADFVRMDTVVKAKNHSAISASVNNFLPVLPLPEFPVNGDANLYRQQLELSDILENASSYSDALGKLQQSSLAKTEWDIERWKPAMLETAISIAQKWKKT
WP_129606978.1	Spirosoma sordidisoli	MTDIRSSVACNLDSHILRAALPLFADEKVGAIEWSFDALVNPRSMPDWFAELLRTYSGAGRLLGHGVFYSLFSGRWTGAQQQWLDQLKSLSRTYRFDHVTEHFGFMTGADFHKGAPMSIPFTESTLAIGRDRLLRLQDACHCPVGLENLAFAYTVDEVDRHGAFLNQLIEPVNGFIILDLHNLYCQSRNFGVDPDGLLSLYPLYRVREIHLSGGSWAPSAVEPGRTIRRDTHDDAVPADVFRLLKQAIDLCPNLRYVVLEQLGAGLQTDGQQSQFQQDFLWMDHIVQAANQIRNPLINHSFLPRRFHVPDPSPLEDPLLYAQQLELSDILETSVSYAQAQQRLAGSGLARSDWAVETWQPAMLETALAIAQKWKNGFD
RYY55266.1	Chitinophagaceae bacterium	MQKPFSSIACNLDTDILTASLPLLEQSKVEGIEWSFDTLFGSTVIPEWFRELLLTYSRAGRLVGHGVFFSLFTGRWLHGQQQWLDQLRKIATEFPFDHVTEHFGFMTGRDFHQGAPINIPFTGSTLAIGRDRLKRIQDAAGCAVGLENLAFAYSLDEVKRHGDFLQQLLLPVNGFIILDLHNLYCQLHNFNMDFEDLIELYPLDKVREIHISGGSFEDSILKPVRSIRRDTHDDRVPEAVFQLLEQTIPRCPNLRYVVLEQLGTALNSAGSKSGFREDFDRMDRIVKSLPATSNPTDIFLPHKNGLPFSIAEDEVLYQQQRQLSEILETSTDGTEALARLRKSALAGTAWDFENWDPYMLETAVGIARKWK
RYZ55261.1	Sphingobacteriales bacterium	MKAGIAVNADADLLRAAFPLFEQEKVGVVEWAFDTLYDQRTLPPWFEELLGHYSSEGALLGHGVFFSLFSARWSPQQAAWLTALKALSRRFSFDHVTEHFGFMTGSDFHKGAPLNIPYNHTTLAIGQDRLARIQDACGCPVGLENLAFTWSADEAKKQGSFLEKLVTHVNGFIILDLHNLYCQLHNFKLGFEDMARFYPLHLVREIHISGGSWDSVAGRQIRRDTHDDQVPADVFDLLEMAIPLCPNLRFVILEQIGQGLVTGESQMQYQKDFLRLEMMLQHIPVDSGGRLDFSPLLPAFPSVPPEDILLAGQQSELCRMLESTESYEQLQHELKNSGICGTDWAIESWEPEMLETARRIAAKWKNGFFE
RZK63166.1	Hymenobacter sp.	MSTPPAATAAPELRATLACNLDADILTAALPLLQEGRVAAIEWSFDTLYWAEQVPDWFTELLHAYGSQHRLIGHGVYFSLLAGRWSAEQAQWLGQLRQLARQFTFDHITEHFGFFTGQNFHSGAPLPMPYTSATLRLGQDRLRRIQDACQCPVGLENLAFAYSLREVEQHGEFLAKLVEPVNGFLILDLHNLYCQLHNFGASCESLLACYPLDRVREIHISGGSWEDSVLEPTRKIRRDTHDEAVPEEVFQLLALTLPQCPNLKYVVLEQLGHGLKTAVSHERFRQDFGRLEALVQQHQAGRRGAAGQPFLPQRPWQPGPVVVDEQLHAQQQQLSQILETATSVAEAQQRLQASALARTDWHIERWEPHMLETALRIAQKWQQ
VFA40327.1	Chryseobacterium indologenes	MMRKPLLGLSMMPEAEFVSAALPLPQNNSVEVLEWSFDTLFNTNEPDWLRDLLNFYAENNRLIGHGVYYSLFDAQWTDRQEIWLSRLKDEVSKRNYNHITEHFGFMNTENFHQGVPLPVPLHPITLQIGQDRLQRLQDAVNIPVGIENLAFSFCLDDVKEQGVFLEKLIESSGGFLILDLHNMYCQSCNFDVDIQEIIKLYPLQKVKEIHLSGGSWQESVYGKKMIRRDTHDHIIPEEIFAILPFVLSHCENLEYIIIERLGPTIKTKEEEDDFFSDFMRVKTMIDSSAGHERQGDLWIKKEFELSKYPVEDPVLYEEQSRLTKLLFDGTHVDSIKNENFHYFNTAGWDPEMIFTAQEIIKKWNPY
WP_129056564.1	unclassified Chryseobacterium	MKRPLLGLSMMAEAEFVSAVLPLLQSNSIEVLEWSFDTFYNAEEPDWLSNLLDFYAENNRLIGHGVYYSLFDARWTERQEIWLNNLKEEFHKRNYNHITEHFGFMNTENFHQGVPLPVSLHSKTLEIGKDRLYRLQDAVQVPVGIENLAFSFSLDDVMEQGVFLDKLTEDTNGFLILDLHNIYCQSCNFGVEMQEIIKLYPLHKVKEIHLSGGSWQESVYGKNPVRRDTHDDAIPEQILSVLSSVLSQCENLEYIIIERLGNTIDTDEKKKSFLNDFETVRRLIDLSAGSGIENNNFWTKKDVLLSENPLEDLLLHEEQTLLTKLLFDNAGPELVKDQKFHYFKTKNWDSEMILTAQNIIKKWNPY
RYD95878.1	Sphingobacteriales bacterium	MGRRIEGIIPGIATNLDAAMLSASLPLLEAGKVGGLEWSFDALYARDYQVPDWFHELLLAYSEAQRLVGHGVFFSLFSGRFSAGQQAWLERLRALTAVYQFDHITEHFGFMTGRDFHKGAPLSVPYTANALAIGRDRISRIADAAQCPVGLENLAFAYDLDEVKIQGRFLDELLNPVNGFMILDLHNLYCQLHNFNMDITTLLDLYPLDKVREIHISGGSWEPHPGAHLQQVRRDTHDDAVPAAVFELLPIALRRCPNLKFVMLEQLGTALTTPVESTQFQEDFNTMQTIIEQYNREQSWPRNIAGFEPASLQLTPAALVDEQLFGHQQIISHILETAADYQDAMAKMQYTSLAGSDWHIEKWQPAMVDTAIHIAQKWK
RYF71102.1	Cytophagaceae bacterium	MIRSTLACNLETNLILASLPLFEAERVEALEWSFDTLYAHEQLPDWFIDLLTTYSEAGRLIGHGVFFSLLSGRWTRDQENWLERLRALTRDYQFDHITEHFGFMTGADFHKGAPINIPCTPAALALGQDRLKRIQDACGCPVGLENLAFAYTLDDVKRQGDFLRQLIQPVNGFIILDLHNLYCQSCNFSIMHDDLLALYPLDCVREIHISGGSWSKSQTQPDVLIRRDTHDDAVPDEVFNWLETTIDQCPNLRYVVLEQLGSGLTSNAQQQMFQADFHRMDTIVQDRNQTTTATTMNSFLPKLPLIINATPLENHRLHNQQVELSAILESSTDVGQAQVRLAQSSLANSEWEIESWEPSMFETAVAIARKWKDGFVSRK
RZK32697.1	Hymenobacter sp.	MSKEPSILAALACNLDADILAAAFPLLAEGRVEAIEWSFDTLYWTEQMPGWFAELLGAYSAQNRLLGHGVFFSLLSGKWTAEQQQWLGHLQQLTQQFSFAHITEHFGSFTGQNFHSGAPLPVPYTPATLRIGQDRLRRIYEACQCPVGLENLAFAYSLAEVQHHGEFLGKLLEPVNGFLILDLHNLYCQLHNFAVSEETLLALYPLDRVREIHISGGSWEESALAPGQKIRRDTHDAGVPEEVFHLLERSMPQCPHLKYVVLEQLGTGLKTEASRTQFRQDFRRMARLVKQCRKGWPTPPTQSFRPLHPHQPGPVAEDAQLHEQQTQLSHILESASSFAEAQRQLHNSALASTAWQLETWEPHMLETAIRIAQKWKG
TAL31200.1	Alphaproteobacteria bacterium	MPFAGLSIFGDARYLDAARPLFAAGAVDAIEWSVDSWSHEEAADEIDTLLDSYAKRGLLIGHGIHYPLLAVKADKLRANWLKQLKPVVQARKYAGVSVHFGFSTGWKLREGAPLPVPLCKEALDLGKKAMVALQKTVKVPVGIENLALAFSKQDVASQGVFIDEMLRAVDGYQLLDLHNIYCQSVNFGVPVLDLVKTYPLSRVLEIHVSGGSWSEHGKMKVRRDTHDGRVPQELFDALPAVLKLCPRARFVIFEKLPQSFKTKADTTGFRDDFMQLKALVPREKAAA
WP_131433417.1	Chryseobacterium gleum	MRKPMLGVSMMAEADFVSAILPLLQNNSIDVLEWSFDTLYHPNEPDWLRDLLNFYAENGRLIGHGVYYSLFDSRWTERQDEWLKKLKEEVRVRNYNHITEHFGFMNTENFHQGVPLPVFLHPKTLQIGKDRLCRLQEAVDIPVGIENLAFSFSVDDVKEQGVFLDQLTEDTNGFLILDLHNIYCQSCNFGVEMKEIVDLYPLDKVKEIHLSGGSWQESDYGKKQVRRDTHDDVIPQDILSVLPSVMEQCQNLEYIIIERLGHTVKTEEEKVSFLNDFNTVRKIIEISDLKQAERSWTKKEMILPEKPLEDRMLYEEQSELTKLLFENADAAVIKNHDFHYFQTENWDPEMILTAQNIIKKWNPY
WP_136580099.1	Niastella caeni	MTTLLATAACNLDSDILNACYPLFAEERVQAIEWSFDVLFNRNVLPPWFEELLHAYSQAKRLTGHGVFFSLFSGKWLPEQAQWLETLRATTKRFPLEQVTEHFGFMTGKDFHAGAPLTIPYSATTLRIGRDRLRRIQDAAQCPVGLENLAFAYTSDDVKRHGYFLHDLIEPVNGFIILDLHNVYCQLENFGIDANELLALYPLHKVREIHISGGSWEPVACDRDRRIRRDTHDDAVPAEVFSLLEMAIDRCAECKYVVLEQVGSGLKTPASRQQFYNDFLQMEKVLQQKNKSRHGNSQNNFLPLHVLNTNPPPEDEFLFQQQRELSRILESDIPYNEAVTVLRRSSLANSVWNIENWNPAMLETAMQIARKWKGE
TGE29932.1	Hymenobacter metallicola	MEVGRVEALEWSFDTLFWAEQVPEWFTELLLTYSQQQRLVGHGVYFSLLSGRWTAEQQQWLQQLQELTKRFAFDHITEHFGFFTGQNFHSGAPLPIPYTRSALHLGQDRLQRIQQACGCPVGLENLAFAYSLDEVQRHGEFLDELVEPVNGFLILDVHNLFCQLHNFAVPYEELIRRYPLDRVREIHISGGSWDDSGPTPGRPVRRDTHDGAVPEEVFQLLEQTMPRCPNLRYVVLEQLGNALKTDAQHTQFRADFCRMEALVTRHRSRAAGRAGQLFLPLQPGAAGPVAEDESLYAQQQQLSRILETAPTFEEARRQLQASPLAHSAWKIEEWEPHMLETARRIAQKWQ

WP_135531367.1	Hymenobacter wooponensis	MPNQPVKADTSEICASIACNLDANILTAAFPLLEEGRVEALEWAFDTLFWAEQMPEWFTELLQAYAAQHRLVGHGVYFSLLSGRWTAEQQQWLQHLRELSKQFAFDHVTEHFGFFTGQNFHSGAPLPIPYGSNTLRIGQDRLRRMQEACGCPVGLENLAFAYAPDEVKRHGEFLEKLVEPVNGFLILDLHNVFCQLHNFSLPYEELVTLYPLERVREIHISGGSWENSGQVPGRQIRRDTHDESVPEEVFQLLELTLPRCPNLKYVVLEQLGTGLRTELSQANFRADFHRMQELVNRHRSTTLRRTTNLFLSQQPLSSGPVAEDAQLHAQQQQLSHILENAPSFEAARQQLQTSSLAYSAWKIEEWEPHMLETAISIAQKWKD
WP_135435099.1	Hymenobacter fodinae	MRAPAQSAPAPEPGIFSALACNLDANILTAAFPLLEEGRVEALEWSFDTLFWTKQVPAWFSDLLQAYAAQHRLVGHGVYFSLLSGRWTAEQQQWLRHLRELTKQFAFDHITEHFGFFTGQNFHSGAPLPIPYGANTLRLGQDRLRRMQEACACPVGLENLAFAYSLEEVKRHGEFLDKLVEPVNGFLILDLHNVFCQLHNFSVPYEELMDLYPLERVREIHISGGSWEDSGQVPGRQIRRDTHDEAVPEEVFQLLERTLPRCPNLKYVVLEQLGTGLQTELSQAQFRADFSRMQALINQHRSTSSHPAPEGFWPQQLLPLGPVAEDAELEAQQQQLSNILETAPSFEAARQQLHASSLANTAWNIEGWEPHMLETAISIAQKWKRKPV
QCE40223.1	Psychroserpens sp. NJDZ02	MRYGIMKNNKPKLGLSLMPNPEFIAAVLPLFEAAQVEVIEWSFDTILDKTYQPEWLPLVLKEYGDNNRLLGHGVYYSLLDANWSANQDNWLKKAKQETLTYNYTHISEHFGLMSATNAHNGFPLPFDLSDTILNIGIDRLKKLQNTVQLDVGIENLALASNASDILKQGEFLNKLVTPVNGFIILDLHNIYCQSENFDIDMLTIITSYPLDLVKEIHISGGSWDQDTSLTKPIRRDTHDGCIPEAILNILPEVLQRCPVLEFIIFERIGDAFKDQKDGIDFRADFNKIKAIIDHTNFSTTPRHWSLNKHNLDQPLIDLELVNQQNTLRQNIQLDQPNAHPEWDTDLWKTATKLYKKWNK
THV62431.1	Chryseobacterium candidae	MKKTMLGVSMMAEADFVSAVLPLLQNNSIDVMEWSFDTLYHANEPDWLRDLLNFYAENNRLIGHGVYYSLFDARWTERQEEWLKKLKEEVNLRKYNHITEHFGFMNTENFHQGVPLPVSLHPKTLQIGKDRLYRLQEAVNIPVGIENLAFSFSIDDVKEQGVFLDKLTEDTNGFLILDLHNIYCQSCNFELEMQEIIDLYPLDKVKEIHLSGGSWQDSVYGKKQVRRDTHDDVIPRDILSVLPSVLTQCENLEYVIIERLGHTLKTDEERDHFLDDFSKVKTIIETSELNGKEKSNWSKMKVELSEKPLEDPVLYEEQSRLTQLLYDNADVETIKDLDFYYFKTESWDAEMILTAQNIIKKWNPY
QCR24856.1	Pontibacter sp. SGAir0037	MEEGRVEALEWSFDTLYWAEQVPDWFSELLHTYSGEERLVGHGVFFSLVSGKWTKEQQQWLEHLKQVCRQFHFDHITEHFGFFTGQNFHYGAPLNIPYTSSTLRIGQDRLMRISDACQRPVGLENLAFSYSLDEVKQHGHFLEKLVEPVNGFIILDLHNLYCQLHNFSVSFEELMQLYPLHRVREIHISGGSWESSVLDPAIRIRRDTHDDAVPAEVFQLLEYALPLCPELKYVVLEQLGNGLKLESSRIQFRQDFLKMEELVKRHTTTRKPAAKNLFLPSHPLQPGAAAEDKTLQAQQYTLSHILETATSYEEALQLLRSSSLANSEWQIESWAPHMLETAISIAQKWRK
WP_137903941.1	Chryseobacterium sp. 2VB	MRKPMLGVSMMAEADFLSAVLPLLQDNAIEVMEWSFDTLYHTNEPDWLRDLLNFYAENNRLIGHGVYYSLFDAIWTARQEEWLKKLKEEVSLRKYNHITEHFGFMNTENFHQGVPLPVSLGPETLQIGKDRLYRLQETVDIPVGVENLAFSFSIDDVKEQGVFLDKLIEDTDGFLIFDLHNIYCQSCNFEVGLQEIINLYPLDKIKEIHLSGGSWQESAYGKKQVRRDTHDDVIPEEILSVLPSVLTQCPNLEYIIIERLGHTLKTEEERNDFLDDFNKVRTIIETSDLKFKENKRWCKKKVKLFEKPLEDLLLYEEQSKLTRLLYDHADVKAIKDLDFHYFKTQNWDAEMILTAQNIIKKWNPY
WP_131957160.1	Dyadobacter psychrotolerans	MSTIFSSVACNLDANILLASLPLFESDQIEAIEWSFDALFKNDELPLWFNNLILEFSSHNRLVGHGVYFSLFSGKWMPEQHDWLKKLKVLAEKYRFDHITEHFGFMTGENFHKGAPMSIPLTAGTMSLGIDRLHRIQDACNCPVGLENLAFAYSIDEVKKQGDFIGKLVESINGFIILDLHNLYCQIHNFDVDYQNIIDLYPLGRVREIHISGGSWDKQLTRTGKPIRRDTHDDAVPEEVFRILSAVIPRCKNLKYVVLEQLGTALDSEQKRKAFRKDFLKMDAIVKESAFQFYNNPVNDFRPYQTIIPHPVPLEDLSFYNQQLQLSNILETAVDQENARHLLSKSSLRDSDWEVEKWESGMIQTAIAIAQKWKEGF
WP_138367034.1	Dyadobacter luticola	MSEIYASVACNLDIPILQASLPLFEEGKIEGIEWSFDALFKWQEIPGWFTDLVHEFSENNRLVGHGVYFSLFSGKWSPEQQQWLEKLRKLSGDFHFDHVSEHFGFLTGDDFHKGAPIGIPFTNSTLTIGRDRLRRIQDACQCPVGLENLAFSYSLEEVKRHGEFLDALVEEVNGFIILDLHNLYCQSHNFNIPFDQMMRLYPLDRVREIHISGGSWDDQLSGFEKIVRRDTHDESVPEEVFEYLKLAIPQCKNLKYVVLEQLGAALETSDQQETFRNDFLKMQAIIQHPGTKSAQSVSDFVTKYFSPLSPIPPEDPHLYRQQKQLSDILENAADSFEAFKMLQNSTLANSDWRVENWEPVMLETAIAIARKWKNGF
TLX25343.1	Chryseobacterium indologenes	MMRKPLLGLSMMPEAEFVSAALPLLQNNSVEVLEWSFDTLFNTNEPDWLRDLLNFYAENNRLIGHGVYYSLFDAQWTERQEIWLSRLKDEVSKRNYNHITEHFGFMNTENFHQGVPLPVPLHPITLQIGQDRLQRLQDAVNIPVGIENLAFSFCLDDVKEQGVFLEKLIDNSGGFLILDLHNMYCQSCNFDVDIQEIIKLYPLQKVKEIHLSGGSWQESVYGKKMIRRDTHDHTIPEEIFAILPFVLSHCENLEYIIIERLGHTIKTKEEEDDFFSDFMRVKTIIDSSAGHERQGDLWIKKEFELSKYPVEDPVLYEEQSRLTKLLFDGTHVDSIKNENFHYFNTAGWDPEMIFTAQEIIKKWNPY
TQM21133.1	Chryseobacterium aquifrigidense	MLGVSMMAEADFVSAILPLLQNNSIEVMEWSFDTLYHTHEPEWLRDLLNFYAENSRLIGHGVYYSLFDARWTERQEEWLKQLKEGVSLRKYNHITEHFGFMNTENFHQGVPLPVSLHSKTLQIGKDRLYRLQETVNIPVGIENLAFSFSIEDVKEQGTFLDQLTEDTNGFLILDLHNIYCQSCNFEAGMQEIIDLYPLDKVKEIHLSGGSWQESVYGKKQVRRDTHDDIIPEEILSVFPSVMAQCPNLEYIIIERLGHTLKTEEERNNFLEDFNKVRTIIETSDLKLKEKSNWCKKEMKLPEKPLEDLLLYEEQSKLTRLIYDNAEVETVKDQDFHYFKTENWDSEMILTAQNIIKKWNPY
WP_138991310.1	Larkinella sp. C7	MANLHSALACNLDANILQASLPLLEAEQVDAIEWAFDTLYRTNKLPDWFDQLLRIYSDAGRLVGHGVFFSLFSGHWTADQQAWLKHLKILSSHYRFDHVTEHFGFMTGADFHTGAPLSVPLNSSTLSIGKDRLKRIQEAARCPVGLENLAFAYSLDDVKRQGDFLHQLIYPVNGFLILDLHNLYCQSHNFRLGYEQLLRLYPLDRVREIHLSGGSWEPATSEPGRTVRRDTHDEAVPTELFGWLEKAIDQCPNLKYVVLEQVGSGLRTEQARQGFARDFRQMKAIVKGKSNGLPSKLNTFLPGEPIVINELPVEDAELFAQQRQLATILESANDYQHARQLLASSNLAGSDWLAEKWQPGMLETAIAIAQKWRKGFS
TVT40773.1	Hymenobacter setariae	MLAALACNLDADILGAALPLLEEGQVEALEWSFDALYWASEVPAWFVELLHAYSDQQRLLGHGVYFSLLSGRWTSEQQQWLNQLRELTQRYSFAHITEHFGAFTGQNFHAGAPLSVPYSPVSLRIGQDRLARIAEAARCPVGLENLAFAYSLEEVKRQGEFLGELLAPVNGFLILDLHNLYCQLHNFSIGYEELLECYALDRVREIHISGGSWENSQLLPTRKIRRDTHDEAVPVEVFNLLARTMPRCPNLKYVVLEQLGTSLKTEASHVQFRRDFVQMAALVQQHNHSPRSVPNAFLPRWPALPALAVQDDLLHEQQRQLSHILETAASLPEAQQRLRASALARSAWQIEHWEPYMLETALSIAQKWK
WP_138506478.1	Spirosoma lacussanchae	MIDIRSSIACNLDSHILRAALPLFADEKVGAIEWSFDALINQRSMPDWFGELLRTYSEAGCLVGHGVFYSLFSGRWTGAQQQWLDQLRSLSRTYRFDHVTEHFGFMTGADFHKGAPMSIPFTESTLAIGRDRLVRLQDACRCPVGLENLAFAYTIDEVNRHGAFLSQLIEPVNGFIILDLHNLYCQSRNFGVEPDALLSLYPLYRVREIHLSGGSWAPSAVEPGRTIRRDTHDDAVPADVFRLLKQAIDLCPNLRYVVLEQLGAGLQTDGQQSQFQQDFLWMDHIVQTANQIRNSLINHSFLPRRFHVPDSSPLEDPLLYAQQLELSDILETSVSCAQAQQRLAGSGLARSNWAVETWQPAMLETALAIAQKWKNGFD
WP_142716532.1	Chryseobacterium rhizoplanae	MKKPLLGISIMAEADFVSAVLPLLQNNAVDVLEWSFDTLYHTNEPDWLRDLLNFYAENERLIGHGVYYSLFDARWTERQEVWLQKLKEEVSLRKYNHITEHFGFMNTENFHQGVPLPVSLHPKTLQIGKDRLYRLQEAVDIPVGIENLAFSFSVDDVTEQGVFLDKLTEDTNGFLILDLHNIYCQSCNFGVEMQEIIDLYPLDKVKEIHLSGGSWQESVYGKKQVRRDTHDDVIPEEIFSVLPSVLAKCQNLEYSIIERLGHTLKTEEDKENFLNDFNKVKAIIEASDGKKKEKSSWNKWQIQLPEKPLEDLALFEEQSRLTRLLFDGAGAGVIKDQDFHYFKTENWDPEMILTAQNIIKKWNPY
WP_144334209.1	Fluviicola chungangensis	MNQLLPKIRSAVSCNLDNQLLQATLPLFAAEKIEAIEWSFDTLYKVREIPIWFVELLHAFGNEGRLVGHGVYFSLFSGKWTAEQQRWLDHLRKVSSNFRFDHITEHFGFMTGEDFHKGAPISVPYKKETLRIGIDRLQRIREACACPVGLENLAFSYSLEEVQRHGEFLERLIEPVNGFIILDLHNVYCQLQNFELDFETLIRAYPLDRVREIHISGGSWDVSGIQPQRTIRRDTHDDAVPEAVFELLRKTLSVCPNLKFVVLEQLGIALHTTESQELFQQDFDHMQAIVNEFNGRNNLIPDNDFLLKQAVSLNEPVPESLELYRQQLELSSILEQAANYEEATRLLNNSTLAHSAWKTESWSPYMLETALRIAQKWRSGW
WP_142722334.1	Chryseobacterium sp. ON_d1	MKRPMLGLSMMAEADFVSAILPLMQNNSIDVLEWSFDTLYHTHEPDWLRDLLNFYAENSRLIGHGVYYSLFDAKWTERQEEWLQKLKEEVRLRKYNHITEHFGFMNTENFHQGVPLPVPLHSRILQIGKDRLCRLQDAVNIPVGIENLAFSFSMDDVKEQGIFLDRLTEDTNGFLILDLHNIYCQSCNFEVGMQEIINLYPLDKVKEIHLSGGSWQESVYGKKQVRRDTHDGVIPQDILSVLPSVLKQCQNLEYIIIERLGHTLKTEQEKVDFLNDFNTVKMIIETSGLKLRQGGTWSKKEMKLSEKPLEDLVLYEEQSRLTRLLFDKMGAEAIQGQDFRYFKTENWDSEMILTAQNIIKKWNPY
WP_142015289.1	Chryseobacterium	MRKPMLGVSMMAEADFVSAILPLLQNNSIEVMEWSFDTLYHTHEPEWLRDLLNFYAENSRLIGHGVYYSLFDARWTERQEEWLKQLKEGVSLRKYNHITEHFGFMNTENFHQGVPLPVSLHSKTLQIGKDRLYRLQETVNIPVGIENLAFSFSIEDVKEQGTFLDQLTEDTNGFLILDLHNIYCQSCNFEAGMQEIIDLYPLDKVKEIHLSGGSWQESVYGKKQVRRDTHDDIIPEEILSVFPSVMAQCPNLEYIIIERLGHTLKTEEERNNFLEDFNKVRTIIETSDLKLKEKSNWCKKEMKLPEKPLEDLLLYEEQSKLTRLIYDNAEVETVKDQDFHYFKTENWDSEMILTAQNIIKKWNPY
WP_246129921.1	Chitinophaga cymbidii	MNDRLFSTVACNLDANILSACLPLMEASKMEAIEWSFDALFRVKEVPSWFLELLTLYSNEKRLIGHGVFFSLFSAKWRQEQDEWLKQLKQTCSAFRFDHITEHFGFMTGRDFHHGAPLNIPYTPSTLNIGRDRLKRIQDACRCPVGLENLAFAYSLDEVKRHGDFLEQLLEPVNGFIILDLHNLYCQLHNFRLEFEDIIALYPLDKVREMHISGGSWDASQIDPDITVRRDTHDDAVPEEVFHLLDQTIGRCPHLKYVVLEQLGTGLSTPESRQSFYDDFLKMDNIIKTKKTSRSHPSFLPLQNDPPGEVVEDETLYRQQLELSHILETAGSCTDAMEALSRSSLANSDWQIEKWAPYMIETAVKIAQKWKK
WP_149388738.1	Chryseobacterium panacisoli	MKKPLLGISMMADADFVSAVLPLLQSNAIDVLEWSFDTLYHTNEPDWLRDLLNFYAENNRLIGHGVYYSLFDARWTERQDNWLQKLKEEVSLRKYNHITEHFGFMNTENFHQGVPLPVSLHPKTLQIGKDRLYRLQETVGIPVGIENLAFSFSVDDVKEQGFFLDKLTEDTNGFLILDLHNIYCQSCNFGVKMQEIIDLYPLDKVKEIHLSGGSWQETVYGKKQVRRDTHDDVIPEEIFSVLPSVLAKCQNLEYIIIERLGHTLKTEEDKENFLNDFNKVKTIIEASDWKGRKKNSWNKGQIQLPEKPVEDLALFEEQSRLTRLLFDNVEAAVIKDQDFHYFKTENWDPEMILTAQNIIKKWNPY
WP_244620440.1	Chitinophaga japonensis	MPEILPAVACNLDNNILAACLPLMEESRIAAIEWSFDALFKVKQVPPWFQELLTAFSNEQRLIGHGVFFSLFSAKWRQEQEDWLRHLQQTCTAFRFDHITEHFGFMTGKDFHHGAPLNIPYTPATLAIGRDRLKRIYEACQCPVGLENLAFSYSLEEVKRHGEFLEQLLVPVNGFIILDLHNLYCQLHNFDLALEDILALYPLDRVREIHISGGSWDSSRADPGRSVRRDTHDDAVPQEVFRLLEAVITRCPHLKYVVLEQLGNGLATEESRQCFYNDFGVMEIIIREKSPAAAGPSFQSFLPTSFSPPGPVLEDEMLYRQQLELSGILETAPSYAAAIRRLNSSSLAHSGWEIERWAPYMIETAMKIARKWKRK
KAA6432601.1	Rufibacter glacialis	MQEGRVEGLEWSFDTLFQQEQVPSWFLALLETYGREKRLIGHGVFFSLFSGKWSEHQEAWLRHLTQMSAAYGFDHVTEHFGFMTGKNFHHGAPLPIPYTAATLAIGRDRLARIQEAAQCPVGLENLAFSYTLEEVKRHGAFLEELVEPVNGFLILDLHNLYCQLHNFSLPLEALLPWYPLHRVREIHISGGSWQDSAVLPQRKVRRDTHDSAVPEEVFDLLSATMPLCPQLKYVVLEQLGTGLKSPEDKALFYQDFCRMETLVKQHNASTFPSLTAPNLFLPLAPGKLPAPVEDEALYQQQLLLSSILEESPSLTQAHQRLLASSLANTAWAIEHWEPHLLETALHLAQKWKKPDLSA
WP_149072616.1	Hymenobacter lutimineralis	MTKPPTIFAALALNLDADMLAAAFPLLADGAIGALEWSFDALSGANTPVWFTELLHAFSKEQRLIGHGVFFSLLSARWTTEQQQWLQRLNQLARQFSFDHVTEHFGFFTGQNFHSGAPLPVPYTPATLRLGQDRLCRISEACSCPAGLENLAFAYSLEEVTRHGEFLDKLLTPVNGFLILDLHNLYCQLHNFSISYEELIALYPLEKVREIHISGGSWADSSLLPDKKIRRDTHDDAVPDEVFSLLKATMPRCPHLKYVVLEQLGNGLQTEHSQTRFRQDFLRMQKLVAQQNQESKPTDYHFLPRQPVVSGPVEEDVRLYAQQQQLSHILETAHSYEEAHQRLLLSSLAQSEWQIEQWAPYMLETALGIAQKWQLKPS
KAA2242006.1	Chitinophaga agrisoli	MEASKVEAIEWSFDALFKVKEIPSWFLELLTAFSDEQRLIGHGVFFSLFSGKWLQEQEDWLRQLEHTSTAFRFDHVTEHFGFMTGKDFHHGAPLNIPYTNATLTIGRDRLRRIQAACQCPVGLENLAFSYSLEEVKQHGAFLDELLAPVNGFIILDLHNLYCQLHNFEIPFDTLISLYPLDKVREIHISGGSWEDSLAAPPKKVRRDTHDDAVPDAVFELLENTIDLCPHLKYVVLEQLGNGLITAESKTQFQQDFLRMEAIVAEKNATRSTESIQSFLPAHPTIPAHIAEDATLYRQQLELSAILETAATYNDAVHALQTSSLAHSDWRIETWAPYMIETAMKIAQKWKK
WP_150032694.1	Taibaiella lutea	MKTNIHAAIACNFDANILSAALPLFEQEKIEAIEWSFDALFKVKEIPGWFDELLLAFSKEQRLIGHGVFFSLFSGKWLPEQIAWLQHLQELCKQFQFDHITEHFGFMTGTDFHTGAPLGIPYTATTLAIGRDRLARIQEVCKCPVGLENLAFSYSLEEVKQHGHFLQELLNPVNGFIILDLHNLYCQLHNFNIPFEELIYLYPLELVREIHISGGSWESSAIFPEKQIRRDTHDDSVPEEVFELLEKALPLCPQLKYVVLEQLGIGLKPIESRDQFQKDFLKMESVITSFEKESIVKEFNSFLPGNPLPTGIPIEDEILYAQQLLLSDILENASNYNEALQRIQSSSLKQSAWNIESWQPDMLETAIRIAQKWKKGW
WP_149831712.1	Chryseobacterium sediminis	MKKPLLGISMMPEADFVSAVLPLLQSNAVDVLEWSFDTLYNTNEPDWLRDLLNFYAENNRLIGHGVYYSLFDARWTERQEEWLQKLKEEVSLRRYNHITEHFGFMNTENFHQGVPLPVSLHPKTLRIGKDRLHRLQQAVDIPVGIENLAFSFSIDDVKEQGVFLDKLTEDTNGFLILDLHNIYCQSCNFGVGMQEIIDLYPLDKVKEIHLSGGSWQESVYGKKQIRRDTHDDIIPEEIFSVLPSVFAKCQNLEYIIIERLGHTLKTEDDKENFLNDFNNVKTIIEISDWKEREKSSWGKRQIQLPEKPVEDMALFEEQSRLTRLLFDGAGAIAIKDQDFHYFKTENWDPEMILTAQNIIKKWNPY
KAB1228757.1	Chryseobacterium viscerum	MPGLRNGIKMRKPLLGVSMMAEAEFVSAILPLLQNNSIEVLEWSFDTLYHTHEPDWLCDLLNFYAKNDRLIGHGVYYSLLDARWTARQEQWLEKLKEEVQQRKYNHITEHFGFMNTDNFHQGVPLPVSLHPKTLQIGKDRLNRLQEAVDIPVGIENLAFSFSVDDVKEQGMFLDKLTEDSNGFLILDLHNIYCQSCNFGVEMQEIINLYPLDKVKEIHLSGGSWQESVYGKRPVRRDTHDDVIPEDIFSVLPAVLTQCQSLEYVIIERLGHTLKTEEEKSNFLHDFNKVKTIIEASDWKNRRENNWTKKEIQLAEKPVEDLVLYEEQSRLTRLLFDNVGAAAIKDQDFHYFKTENWDPEMILTAQNIIKKWNPY
WP_157814118.1	Olleya sp. Bg11-27	MKINKPKLGLSIMPNPEFIAAALPLFESAQVEVIEWSFDTILDKKYQPEWLPLLLKEYGDNNRLLGHGVYYAPLDANWGANQDNWLQKAKLETLAYNYNHLSEHFGVMSSANAHHGFPLPFDLSNTILNIGIDRLKRLQNTVQLDIGIENLALASNVSDILKQGAFIEKLVAPINGFVILDLHNIYCQSINFDLDLLTIITSYPLHLVKEIHLSGGSWDHDHNLTKPIRRDTHDGRIPEVLLDILPEVLQRCPVLEFVIFERLGDTFQNKNDGLEFRTDFNKIQAIIDHTAFSSNARQWTLKKHDLGPPLIDLPLLNKQQELREHIRLETANNHAEWDTNMWITATKLYKKWNA
MXV51032.1	Hufsiella arboris	MKSDPVTAIASTIACNLDSDILSAAIPLFEESRVEAIEWAFDSLDFSEVPEWFSDLIDAYSKEGRLIGHGVFFSIFSGRWSIQQQEWLDNLKKLSARFKFDHITEHFGFMTGANFHYGAPLAVPFNSKTLTIGRDRLNRIYDACQCPVGLENLAFSYSIDEVKKHGEFLETLVEPVNGFIILDLHNLYCQMHNFSISFDEIIKLYPVDRVREFHISGGS

		WENSSLSGKRIRRDTHDDAVPDEVYQLLDSALDHCKNVRYVTMEQLGSGLKTEESRSNFYNDFLKMQQIVRNKTSVPNDAENTFFPPKIVPPGDIIEDENLYIEQQQLSYILENSTSYDDAVSRIKSSVLAKSDWGIESWAPHMLETAISIAQKWKKK
WP_153391763.1	Chryseobacterium	MKKPLLGLSMMPEADFVSAILPLLQDQSVAVLEWSFDTVEQMHEPEWLSGLLDFYAENNRLLGHGVVYYSLFDARWTERQEVWLEKLKQEIQRRKYNHITEHFGFMNTENYHQGVPLPVPLHPKILQIGKDRLARLQAVVEIPVGVENLAFSFSADDVREQGIFLEKLIEEVDGFLILDLHNIYCQSCNFEIDILDIVTMYPLEKVKEIHLSGGSWQESVYGKKPIRRDTHDDAIPEEVLAVLPEVLSRCPNLEYVIIERLGHTISTNIEKESFFNDFVKTKKIIEGCLFPARKKTVWNRKEYISPEPLEDQLLYHEQTKLTRLLHENGDIAFFKNEDFSYFNVQKWDPEMIETARSIIKKWNPY
WP_149639431.1	Dyadobacter sp. UC 10	MSRIFSAIACNLDVHILQAAFPLFEMEMVEAIEWSFDSLFKVGTIPDWFIELVKEFSDHNRLIGHGVYFSLFSGKWSQDQQNWLDQLRKLSGEFRFDHVSEHFGFMTGADFHKGAPLGIPFNQSTLAIGQDRLARIQDACNCPVGLENLAFSYSYEEVRRHGDFLNQLVESVNGFIILDLHNLYCQCHNFNIHFEQMLSCYPLERVREIHISGGSWDNGTANRPVRRDTHDEAVPEVVFKYLEKALLQCPNLKFVVMEQLGTALDSGEKQYLFQQDFLQMSHIVKSFEENFPPVPVNSFLPPATFPLHSKPVEDLLLAKQQMQLSAILENASNLDHARGMLNASDLKNSDWHIDQWQPHMLKTAMDIARKWKDGF
WP_158978994.1	Cellulophaga sp. L1A9	MRKRIMGIKKPKLGIAIAPSLKFLEAALPLFAGEKIEIIEWSFDTLKDAADEPAWLSLLLKEYGEKNRLLGHGVFYSLLDANWSSRQENWLKKVRQETLSHKYCQISEHFGLMSSANAHSGFPLPIQLSNPVLQIGIDRLKRLQATAQVDVGIENLALTANVADILEQGEFLFKLVNPVNGFVILDLHNIYCQSENFNIDMMTIIKSYPLSLVKELHISGGSWDTDPTLSRKIRRDTHDGRIPEVILEILPEVLKICPFLTFIIFEKIEDSFLTEKDGIDFRADFQKIREIIDATVFSVTPKEKKQVAAILGPPVIDVELLQAQVALRESIALGTYQTNSEWDKDMWKVATKLYEKWNNY
WP_159477007.1	Dyadobacter sp. 3J3	MQKIFTSVACNLDTNILLAALPLFEQDKIQALEWSFDTLFKYEEIPDWFTDLVLEFSKNNRLIGHGVYFSLFSGKWLPSQQEWLEKLKIFTLKFRFDHITEHFGFMTGSDFHKGAPISIPFTPVTLALGKDRLNRIQDACQCPVGLENLAFSYSIEEVRKHGNFLDQLTDEINGFIILDLHNLYCQIHNFDITFKDIIHLYPVEKVREIHISGGSWDERLIGNGKQVRRDTHDQAVPQEVFELLINTIPLCPNLKYVVLEQLGTALGSAESRIMYQEDFLRMDAIVKGAEFNASGHNTFMPLYKTNLQATPLEDLNLYKQQQQLSQILETAVDLHQAQKLLSLSDLRNSAWNVENWEPYMLETAMSIAQKWKNGFD
QHT72239.1	Rhodocytophaga rosea	MEESQVDAIEWSFDTLFNIWHTPGWFARLLTAFSKEERLIGHGVFFSLLSGKWSKEQYQWLSQLKQLSAEFHFDHITEHFGFMTGQSFHQGAPMCVPYSPATLAIGRDRLARIQEACSCPVGLENLAFAASLEQVKQQGEFLERLIEPVNGFIILDLHNLFCQMHNFFMDFEQLLSSYPLERVREIHISGGSWEKSGKQSERIIRRDTHDQAVPPEVFHLLEKTIPVCPNLKYVVLEQLGIALKTQESKAAFYQNFVQMKQIVEDNNQTIANSSENPFIPPSITLPETFVENQELYAQQRELSEILETSSSYEETNRLLKASSLAHTAWNIESWDPAMIETAIHIARKWKK
WP_202842763.1	Chryseobacterium cucumeris	MRKPMLGVSMMAEADFVSAVLPLLQNNSIEVMEWSFDTLYHTNEPEWLRDLLNFYAENNRLIGHGVVYYSLFDARWTARQEEWLKKLKEEVSLRKYNHITEHFGFMNTENFHQGVPLPVSLGPETLQIGKDRLYRLQEAVNIPVGVENLAFSFSIDDVKEQGVFLDKLIEDTDGFLILDLHNIYCQSCNFEVGLQEIINLYPLHKIKEIHLSGGSWQESAYGNKQVRRDTHDDVIPEEILSVLPSVLTQCPNLEYIIIERLGHTLKTEEERNDFLDDFNKVRTIIETSDLKFKENKRWCKKEVKLSEKPLEDLLLYEEQSKLTKLLYDHTDVKAIKDLDFHYFKTQNWDAEMILTAQNIIKKWNPY
NEQ25955.1	Microcoleus sp. SIO2G3	MFKFKRRTPTVGLSLMMESDFFQASQPLFEAGEVEVLEWSFDMGWGVDALPPWVEQLLQFYSQRDRLIGHGVSYSLLSAQPGERQIHWLKSLEEECLDYRYRHISEHFGWMAAGDFYQSAPLPLPLTPETLHLGREHLKRLSDTAKAPVGLENLAFAFGLQDVLQQGEFIERLLEPVDGFLVLDLHNLYCQVHNFQLSADQLLERYPLHRVRELHVSGGSWSVTSKGEQTRTIRRDTHNGTVPEAVFELLALALKRCPSVEAVIFERIGHTLHNEVDIEGFRQDFWRIKQVVKESV
WP_161990772.1	Chryseobacterium viscerum	MRKPLLGVSMMAEAEFVSAILPLLQNNSIEVLEWSFDTLYHTHEPDWLCDLLNFYAKNDRLIGHGVVYYSLLDARWTARQEQWLEKLKEEVQQRKYNHITEHFGFMNTDNFHQGVPLPVSLHPKTLQIGKDRLNRLQEAVDIPVGIENLAFSFSVDDVKEQGMFLDKLTEDSNGFLILDLHNIYCQSCNFGVEMQEIINLYPLDKVKEIHLSGGSWQESVYGKRPVRRDTHDDVIPEDIFSVLPAVLTQCQSLEYVIIERLGHTLKTEEEKSNFLHDFNKVKTIIEASDWKNRRENNWTKKEIQLAEKPVEDLVLYEEQSRLTRLLFDNVGAAAIKDQDFHYFKTENWDPEMILTAQNIIKKWNPY
WP_164720324.1	Chryseobacterium gleum	MLGVSMMAEADFVSAILPLLQNNSIDVLEWSFDTLYHPNEPDWLRDLLNFYAENGRLIGHGVVYYSLFDARWTERQDEWLKKLKEEVRLRNYNHITEHFGFMNTENFHQGVPLPVSLHPKTLQIGKDRLCRLQEAVDIPVGIENLAFSFSVDDVKEQGVFLDQLTEDTNGFLILDLHNIYCQSCNFGVEMKEIVDLYPLNKVKEIHLSGGSWQESAYGKKQVRRDTHDDVIPQDILSVLPSVMEQCQNLEYIIIERLGHTVKTEEEKVSFLNDFNTVRKIIEISDLKLAERNWMKKEMILPEKPLEDRVLYEEQSELTKLLFENADAAVIKNHDFHYFKTENWDSEMILTAQNIIKKWNPY
WP_165446360.1	Chryseobacterium indologenes	MRKPLLGLSMMPEAEFVSAALPLPQNNSVEVLEWSFDTLFNTNEPDWLRDLLNFYAENNRLIGHGVVYYSLFDAQWTDRQEIWLSRLKDEVSKRNYNHITEHFGFMNTENFHQGVPLPVPLHPITLQIGQDRLQRLQDAVNIPVGIENLAFSFCLDDVKEQGVFLEKLIESSGGFLILDLHNMYCQSCNFDVDIQEIIKLYPLQKVKEIHLSGGSWQESVYGKKMIRRDTHDHIIPEEIFAILPFVLSHCENLEYIIIERLGPTIKTKEEEDDFFSDFMRVKTMIDSSAGHERQGDLWIKKEFELSKYPVEDPVLYEEQSRLTKLLFDGTHVDSIKNENFHYFNTAGWDPEMIFTAQEIIKKWNPY
WP_162149646.1	Chryseobacterium indologenes	MRKPLLGLSMMPEAEFVSAALPLLQNNSVEVLEWSFDTLFNTNEPDWLRDLLNFYAENNRLIGHGVVYYSLFDAQWTDRQEIWLSRLKDEVSKRNYNHITEHFGFMNTENFHQGVPLPVPLHPITLQIGQDRLQRLQDAVNIPVGIENLAFSFCLDDVKEQGVFLEKLIDNSGGFLILDLHNMYCQSCNFDVDIQEIIKLYPLQKVKEIHLSGGSWQESVYGKKMIRRDTHDHTIPEEIFAILPFVLSHCENLEYIIIERLGHTIKTKEEEDDFFSDFMRVKTIIDSSAGHERQGDLWIKKEFELSKYPVEDPVLYEEQSRLTKLLFDGTHVDSIKNENFHYFNTAGWDPEMIFTAQEIIKKWNPY
WP_160139545.1	Chryseobacterium sp. c4a	MGKPLLGLSMMAEAEFVSAVLPLLQSNSVEVFEWSFDTFYNAEEPGWLSDLLNFYAGNNRLIGHGVVYYSLFDALWNERQEIWLGKLRDELRKRKYNHITEHFGFMNTENFHEGVPLPVSLHSKTLEIGKDRLHRLQDAVQVPVGIENLAFSFSLDDVMEQGLFLDKLTEDSDGFLILDLHNIYCQACNFKVGMEDIIKLYPLNKIKEIHLSGGSWQESIYGKKPIRRDTHDDIIPEEILSILPFVLSQCKNLEYVIIERLGHTIDTEEKRSDFLDDFRKVKMIIDSMDWKIFDRNSWIKKEIKISKKPLEDKGLYDEQTLLTKLLFDHVNVESIKNHEFHYFKTKDWDPEMILTAQNIIKKWNPY
WP_165748299.1	Cellulophaga sp. Z1A5H	MGNKKPKLGIAIAPSLKFLEAALPLFAEEKIEIIEWSFDTLKDAADEPVWLSLLLKEYGEKNRLLGHGVFYALLDANWSSRQENWLKKVRQETLSHKYCQISEHFGLMSSANAHSGFPLPIPLSNPVLQIGIDRLKRLQATAQVDVGIENLALTANVADILEQGEFLFKLVNPVNGFIILDLHNIYCQSENFNIDMMTIIKSYPLSLVKELHISGGSWDTDPTLSRKIRRDTHEGRIPEVILEILPEVLKICPFLAFIIFEKIEDSFLTEKDGIDFRADFQKIREIIDATSFSVTPKEKKQVAIILGPPVIDVELLQAQVALRESIALDTYQTNSEWDKDMWKVATKLYEKWNN
NJK30207.1	Acaryochloris sp. SU_5_25	MPPLVGLSLMPQADFMAAAQPLFRAGEVEILEWSFDMGWGNVSLPPELPPLLDQFSQCHCLLGHGVSYSLLSAGIDRSHWLACLQAECQDYSYRHISEHFGWMATRHFSRSVPLPLPLLPATLKLGCDHIQRLAEVAQVPVGLENLAFAFGRQDVDQQGEFLDRLLEPVDGFLLLDLHNLYCQMHNFQLSASEILNLYPLGRVRELHLSGGSWSQSGTAKIRRDTHDHAVPEPVFDLLALALQECSHVEAIILERLGNTFTTDAEKIGFRQDFARIKEIVYSHRSSLSRKG
WP_167358395.1	Chryseobacterium culicis	MNKPLLGISMMAEADFVSALLPLLQSNSVDVLEWSFDTLYHNHEPVWLCDLLNFYADNERLIGHGVVYYSLFDARWTERQEDWLKKLKEEVALRRYNHITEHFGFMNTENFHQGVPLPVSLHAKTLEIGKDRLYRLQDAVNIPVGIENLAFSFGIDDVKEQGAFLDKLIEDTDGFLILDLHNIYCQACNFGIEIQEIMDWYPLDKVKEIHLSGGSWQESVYGKRPVRRDTHDDSIPKELFSVLPSVVSQCQHLEYVIIERLGHTLKTDEEKNDFLTDFNRVKKIIEASDWKGEQKKNWKKKEIKLTEKPLEDRVLFEEQSRLTQLLFDHVDAAVIKDVDFHYFKTENWDLEMILTAQNMIRKWNPY
WP_166692314.1	Fibrivirga algicola	MAAIRSTLACNLETNLILAALPLFEAERVAALEWSFDALYAHEQLPDWFTELLTTYSEADRLVGHGVFFSLLSGRWTSEQQAWLIRLRALSREFRFDHITEHFGFMTGADFHKGAPINVPCTAVTLALGQDRLSRIQDACRCPVGLENLAFAYSLDDVKRQGDFLRQLIEPVNGFIILDLHNLYCQSKNFNLDLTALLDLYPLDRVREIHISGGSWAEVEIEPGRHIRRDTHDDAVPDDIFNWLSTAIDRCPNLGYVVLEQLGAGLTTGSERLAFQADFDRMDVIVQQKNKSIAHPDMQSFLPELPLLINPVPLENHRLYTHQLELSAILETAVDVQQAKRRLAESSLANSEWEVERWEPAMLETAVAIARKWKNGFVGKE
WP_165835292.1	Chryseobacterium viscerum	MRKPLLGVSMMAEADFVSAILPLLQNNSIEVLEWSFDTLYHTHEPDWLCDLLNFYAENNRLIGHGVVYYSLLDARWTARQEQWLEKLKEEVQQRKYNHITEHFGFMNTDNFHQGVPLPVSLHPKTLQIGKDRLNRLQEAVDIPVGIENLAFSLSVDDVKEQGMFLDKLTEDSNGFLILDLHNIYCQSCNFGVEMQEIINLYPLDKVKEIHLSGGSWQESVYGKMPVRRDTHDDVIPEDIFSVLPAVLTQCQSLEYVIIERLGHTLKTEEEKSNFLHDFNKVKTIIEASDWKNRRENNWTKKEIQLAEKPVEDLVLYEEQSRLTRLLFDNVGAAAIKDQDFHYFKTENWDPEMILTAQNIIKKWNPY
WP_165851835.1	Chryseobacterium pennipullorum	MAIIAAPPVVWDKMKKPLLGLSMMAEAEFVSAILPLLQSNLVDVLEWSFDTFFHAEEPDWLNDLLDFYSDSRRLTGHGVVYYSLFDAKWTERQEVWLDTLKKEVQTRTYNHITEHFGFMNTENFHQGVPLPVSLHPVTLQIGKDRLFRLQDAVNIPVGIENLAFSFGIDDVKEQGVFLDKLTEDTNGFLILDLHNIYCQSCNFEIPMEEIINSYPLDKVREIHLSGGSWQESAYGKKQIRRDTHDDAIPESLLDVLPSVLLKCKNLQYVIIERLGHTISTEKEKKRFTEDFQKVKKMLEAPGLLTENTTSWIKKGVVIPDVPLEDLALAEEQSRLTRLLFEGADAAAFKKENFHFFNTRNWDSEMIRTAQNIIKKWNPY
WP_167288130.1	Paraflavitalea devenefica	MPKILSSVACNLEADILGACIPLWEEARIEAIEWSFDALFNVEEIPAWFRELLTAFSKEKRLIGHGIFFSLFSGKWLPEQQQWLNKLQQTCTEFQFDHITEHFGFMTGKDFHQGAPLNIPYTTSTLAIGRDRLQRIQEACQCPVGLENLAFSYSLDEVKRHGTFLDQLLEPVNGFIILDLHNLWCQLHNFSIPFEEVIDLYPLHRVREIHISGGSWDDSEVEAGRTIRRDTHDNAVPAAVFQLLEMTISRCPHLQYVVLEQLGTGLLTPESKQQFYQDFGRMESIVSEKNLSRTPHSTCSFLPQAPLQKGGVTEDLFLYQQQRELSAILESAPSYEDARRLLAQSSLAHSDWKIENWDPAMVETAIKIAQKWKQGWLT
WP_168679930.1	Hymenobacter sp. BT18	MTKPPTIFAALALNLDADMLAAAFPLLADGSVGALEWSFDALSGANTPVWFTELLHAFSKEQRLIGHGVFFSLLSARWTTEQQQWLQRLNQLARQFSFNHVTEHFGFFTGQNFHSGAPLPVPYTAATLRIGQDRLCRISEACSCPVGLENLAFAYSLEEVKRHGEFLDKLLTPVNGFLILDLHNLYCQLHNFSISYEELIALYPLEEVREIHISGGSWADSSLLPDKKIRRDTHDDAVPDEVFSLLKATMPRCPHLKYVVLEQLGNGLQTEHSQTRFRQDFLRMQKLVAQQNQESRPTDYHFLPRQPVVSGPVEEDVRLYAQQQQLSHILETAHSYEEAHQRLLLSSLAQSEWQIEQWAPYMLETALGIAQKWQLKPS
NJN38771.1	Acaryochloridaceae cyanobacterium CSU_3_4	MAWAKLPMPPLVGLSLMPQADFMAAAQPLFRAGEVEILEWSFDMGWGNVSLPPELPPLLDQFSQCHCLLGHGVSYSLLSAGIDRSHWLACLQAECQDYSYRHISEHFGWMATRHFSRSVPLPLPLLPATLKLGCDHIQRLAEVAQVPVGLENLAFAFGRQDVDQQGEFLDRLLEPVDGFLLLDLHNLYCQMHNFQLSASEILNLYPLGRVRELHLSGGSWSQSGTAKIRRDTHDHAVPEPVFDLLALALQECSHVEAIILERLGNTFTTDAEKIGFRQDFARIKEIVYSHRSSLSRKG
WP_167028800.1	Chryseobacterium sp. Tr-659	MGKPLLGLSMMAEADFVSAILPLMQNNAVDVLEWSFDTLYNVKEPDWLNDLLNFYAENNRLIGHGVVYYSLFDARWTERQNVWLKKLKEEFLRRNYNHITEHFGFMNTENFHQGVPLPVSLHPKTLQIGKDRLYRLQEAVNVPVGIENLAFSFSSDDVKEQGEFLDQLIEDTDGFLILDLHNLYCQACNFEVEMQELINSYPLDKVREIHLSGGSWQKSVYGKRMVRRDTHDDLIPEEILSILPSVLAQCKNLEYVIIERLGNTIKTEAEKEGFFTDFNKVRAIIDASDLQVGKMGNWTKKEIKLPDEPLENMELFEEQSRLTKLLFDSVGPAILKGQQFHYFKTENWEPEMILTAQNIIKKWNPY
WP_172523132.1	Chryseobacterium indologenes	MRKPLLGLSMMPEAEFVSAALPLLQNNSVEVLEWSFDTLFNTNEPDWLRDLLNFYAENNRLIGHGVVYYSLFDAQWTERQEIWLSRLKDEVSKRNYNHITEHFGFMNTENFHQGVPLPVPLHPITLQIGQDRLQRLQDAVNIPVGIENLAFSFCLDDVKEQGVFLEKLIDNSGGFLILDLHNMYCQSCNFDVDIQEIIKLYPLQKVKEIHLSGGSWQESVYGKKMIRRDTHDHTIPEEIFAILPFVLSHCENLEYIIIERLGHTIKTKEEEGDFFSDFMRVKTIIDSSAGHERQGDLWIKKEFELSKYPVEDPVLYEEQSRLTKLLFDGTHVDSIKNENFHYFNTAGWDPEMIFTAQEIIKKWNPY
WP_168800700.1	Psychroserpens sp. NJDZ02	MKNNKPKLGLSLMPNPEFIAAVLPLFEAAQVEVIEWSFDTILDKTYQPEWLPLVLKEYGDNNRLLGHGVVYYSLLDANWSANQDNWLKKAKQETLTYNYTHISEHFGLMSATNAHNGFPLPFDLSDTILNIGIDRLKKLQNTVQLDVGIENLALASNASDILKQGEFLNKLVTPVNGFIILDLHNIYCQSENFDIDMLTIITSYPLDLVKEIHISGGSWDQDTSLTKPIRRDTHDGCIPEAILNILPEVLQRCPVLEFIIFERIGDAFKDQKDGIDFRADFNKIKAIIDHTNFSTTPRHWSLNKHNLDQPLIDLELVNQQNTLRQNIQLDQPNAHPEWDTDLWKTATKLYKKWNK
QJB36205.1	Chitinophaga oryzae	MEAAKVEAIEWSFDALYAAENVPAWFEELLAAFSKENRLIGHGVFFSLFSGRWLPEQQQWLDHLKTVSRHYRFDHVTEHFGFMTGKDFHHGAPLNIPYTPVTLGIGRDRLKRMQDACGCPVGLENLAFSYSLEEVKRHGDFLNELLEPVNGFVILDLHNLYCQAHNFDLSFEALKDLYPLHRVREIHISGGSWDESEATPGKTIRRDTHDDAVPATVFNYLDKAIDLCPSLKYVVLEQLGSGLASEESRSAFRNDFLRMDQLLNSKNRTTAPLNTFLPEAFQVPAQVPEDETLYRQQLELSQILETAASFDAAMKALQASSLAHSAWQIENWDPAMIETAAKIAQKWKRKDIVFKT
QJB42701.1	Chitinophaga oryzae	MEAAKVEAIEWSFDALYAVENVPAWFEELLNVFSEENRLIGHGVFFSLFSGRWLPEQQQWLDHLKTVSRHYRFDHVTEHFGFMTGKDFHHGAPLNIPYTPVTLGIGRDRLKRMQDACGCPVGLENLAFSYSLEEVKRHGDFLNELLEPVNGFVILDLHNLYCQAHNFDLSFEALKDLYPLHRVREIHISGGSWDESEATPGKTIRRDTHDDAVPATVFNYLDKAIDLCPSLKYVVLEQLGSGLASEESRSAFRNDFLRMDQLLNNKNKSTVPLNSFLPEALQIPAQAQEDETLYRQQLELSQILETAASFGATMKALQASSLAHSDWQIESWDPAMIETAAKIAQKWKRKTTNP
WP_168237931.1	Chryseobacterium gallinarum	MEKPFLGISMMAEADFLTAILPLLRDGSIDVLEWSFDTFYHAEEPDWLTRLLNFYSQNNRLLGHGVVYYSLFDAKWTERQSMWLQKLKEEVQQRKYNHITEHFGFMNTENFHQGVPLPVSLHPKTLEIGKDRLCRLQEVLEIPVGIENLAFSFSIDDVKQQGIFLEKLIEDTDGFLILDLHNIYCQACNFGLGIEEIVRLYPLEKVREIHLSGGSWQTSPYGKRRIRRDTHDDTVPAEILAVLPSVLSQCENLEYVIIERLGHTIQTETEAKDFLDDFLKVKAIIGASEWNSRHKEIKIKKKPECAGRPVEDEGLYREQTILTKLLFDNVKADHFKDFDFQYFKTENWDPEMIRTAQNIIKKWNPY
NNE28949.1	Saprospiraceae bacterium	MIKAAISCNLDTHILQTALPLFRTAEVDAIEWSFDTLYKTKNIPDWFVELLRTFSDGKGLIGHGVFFSIFSGEWTEGQQRWLQHLEKVSKHFSFQHITEHFGFMTGADFHKGAPISVPMDNTSLSIGGDRLKRIANACSCPVGLENLAFTYSLEEVKKHGEFLSRLVSTVNGFIILDLHNLYCQMHNFQISFEEIIKLYPLELVREIHISGGSWQESD

		LAPEKKVRRDTHDQAVPIEVFNMLSKAIPLCPNLKFVVLEAIGTGLKTEKEKEDFQKDYQTMKALLTKSEPNPGIKNFIPDIPYELGEPMSDDEFALQQIELSNILETADNLTTAQASLSNSSLANTSWDIEHWSPHMLEAVIAIAQKWKMGFD
WP_171032588.1	Chryseobacterium indologenes	MRKPLLGLSMMPEAEFVSAALPLLQNNSVEVLEWSFDTLFNTNEPDWLRDLLNFYAENNRLIGHGVYYSLFDAQWTERQEIWLSRLKDEVSKRNYNHITEHFGFMNTENFHQGVPLPVPLHPITLQIGQDRLQRLQDAVNIPVGIENLAFSFCLDDVKEQGVFLEKLIDNSGGFLILDLHNMYCQSCNFDVDIQEIIKLYPLQKVKEIHLSGGSWQESVYGKKMIRRDTHDHTIPEEIFAILPFVLSHCENLEYIIIERLGHTIKTKEEEDDFFSDFMRVKTIIDSSAGHERQGDLWIKKEFELSKYPVEDPVLYEEQSRLTKLLFDGTHVDSIKNENFHYFNTAGWDPEMIFTAQEIIKKWNPY
WP_168801929.1	Chryseobacterium candidae	MLGVSMMAEADFVSAVLPLLQNNSIDVMEWSFDTLYHANEPDWLRDLLNFYAENNRLIGHGVYYSLFDARWTERQEEWLKKLKEEVNLRKYNHITEHFGFMNTENFHQGVPLPVSLHPKTLQIGKDRLYRLQEAVNIPVGIENLAFSFSIDDVKEQGVFLDKLTEDTNGFLILDLHNIYCQSCNFELEMQEIIDLYPLDKVKEIHLSGGSWQDSVYGKKQVRRDTHDDVIPRDILSVLPSVLTQCENLEYVIIERLGHTLKTDEERDHFLDDFSKVKTIIETSELNGKEKSNWSKMKVELSEKPLEDPVLYEEQSRLTQLLYDNADVETIKDLDFYYFKTESWDAEMILTAQNIIKKWNPY
NOT36767.1	Saprospiraceae bacterium	MIYCGTALNLDHKLLSANLPLLANSKLEAIEWSFDALYKLEQVPDWFDQLVKLYSNQKRLIGHGVFFSIFSGKFSSEQKKWLEDLKILSNKYRFDHITEHFGFMTGEDFHKGAPLSIPMNLTALRIGQDRLKRIQDVSNCPIGIENLAFAFNVEDVKRQGEFLNDLLSPINGFMILDLHNLYCQAHNFKIDIDNLLQYYPLELVREIHISGGSWEDDIVHYNEKIRRDTHDDSVPSEVYICLESCLLKLDNLKYVILEQLSNALESESQQKKYQSDFYKILAIVKNNKVDEIRRNDFHPSFLDISTQEAFEDLHLYQQQAELSHILVDSKNLEEAKQKLSHSSLRNSDWNVELWQDNMLYTAMSIAQKWK
HIA55772.1	Candidatus Melainabacteria bacterium	MPCKPKLGLSLMPTEDFEQATVSLFKNKQVQAIEWSFDFSWNGAVVSEWCDGILHEFSNRGALTGHGVNLSPLSARFSKRQEEWLANAKEEFKNRKYVHATEHFGFSEAGPIAQGAPLGVPMNAESLRKGKDMMKRYADATQCPVGLENLAFAFGLEDVKRQGDFIDQLISSVDGFLLLDVHNIYCQLANFGVSELELLNSYPLSKVREIHLSGGTWSGSISGKRAAVRRDTHDDAVPQEVFNLATLALKLCPNIEFVIMERLGYTMMELAEQQEFREDFETMEEILEFCYA
WP_169236210.1	Chryseobacterium antibioticum	MSRPLLGLSMMPEADFVSAILPILQNNSVDVLEWSFDTFYDADEPEWLSGLLDFYAENNRLLGHGVYYSLFDARWTDRQDVWLKKLKEELRHRKYNHITEHFGFMNTENFHQGVPLPVPMHSRILQIGKDRLRRLQDVVDIPVGVENLAFSFSINDVKEQGEFLDKLVEDIDGFLILDLHNIYCQSCNFEVDMQEIIKLYPLEKVKEIHLSGGSWQESAYGKKPVRRDTHDDRIPDKIVEILPKVLSQCPNLEYVIIERLGHTLNVESEKQVFFADFNRVKEMIEMSDDLPGDRKVWNKKKGEYSKPVEDLMLYHEQTKLTKLLFDGEGVGSVKNQKFHYFKPENWDEEMIMTAQQIIKKWNPY
NQZ69047.1	Lentisphaeria bacterium	MKPLIGLSLMHEPEFLQASLELFENDCVDCVEWSFDSLWNNFSSCPWLDELLIEYSSQGRLFGHGVHFSFLSAELSTEQMDYLDKLNNEFDSRNYVHSSEHFGFMARTALGKGAPLPVPLIPDVFQLAEQRLELLGKHTGTAVGLENLALAFNKEDALKHYSFINDLAANGFILLDLHNIYCQIENFGISLADVLERIDSSRVHEIHISGGSWEEAMGKRIRRDTHDDAVPDAVFDLLQAALPQLPNVKCVIFERLGNSFKNENDAIEFRSDFMKIRASVT
WP_171593135.1	Hymenobacter	MSTPTQLAKADAPGIFSSIACNLDANILTAAFPLLEDERVEALEWSFDTLFWAEQVPEWFTALLQAYAAQNRLVGHGVYFSLLSGRWTAEQQQWLQHLKALAGQFAFDHITEHFGFFTGQNFHSGAPLPIPYTSTTLRLGQDRLHRMQEACRCPVGLENLAFAYSLDEVKRHGEFLEKLVEPVNGFLILDLHNLFCQLHNFSVSYEALLALYPLERVREIHISGGSWEASGQVPGRQIRRDTHDEAVPEEVFQLLEQTLPRCPNLKFVVLEQLGTGLKTESSQAQFRADFRRMQQLINHYRSTTHRQASQLFLPQQPYSAGPVAEDAQLHTQQQQLSHILETAPSYAEARQLLQTSALAHTDWKIEEWEPHMLETAISIAQKWKQ
NUO01438.1	Saprospiraceae bacterium	MPRPFAALACNLDRNTLLTCLPLFEAEKVEAVEWSFDALFQFKEIPPWFTGLLQTFSRENRLVGHGVFFSLFSGKWLEGQQTWLDELKKLSKTYCFDHITEHFGFMTGADFHNGAPISLPFTTTTLGIGRDRLARIYDACECPVGLENLAFAYSIEEVKRHGEFLEQLLSPINGFIILDLHNLYCQLHNFDLEFADLIQLYPLDRVREIHISGGSWEDSPLRPGKPVRRDTHDDSVPQEVFQLLEQTIPLCPNLKFAVLEQLGTGLGTEASRVAFQQDFDAMSGILQQSRDDSGHPANAFFPQTRPDTSQPPIESNELEQQQQELSQILETATDYEAAKNRLATSRLAHSAWNIESWEPAMLETAMRISQKWKHGFLQTQR
NUQ53559.1	Phycisphaerales bacterium	MESPLPVGISLMLEEDFLGATLPLFEEGAVDVLEWSFDVGWGRELPAWAQGLLQHFAVSGRLLGHGVSFSPLSARWEEPQRNWLHRAALEVAKLKYRHVSEHFGFMAAGSFTRGSPMPMPADAAVIRTGRDALSRLAEVTGLPVGLENLALALSSRDVDGQGDHLDALLEPVGGFVVLDLHNLWCQAVNFGRDPVGLLGRYPLSRGITLVLLKIDARVLLVSQSVSRSGASMQTLCEIDEPDQVASILLKASRNERSSIADRFQSLLSSSDRAMEKELESAGRQVFRTSSGDTVELASISTPVPLARINAGDAAARRERNEGAPNRLSQPIQPPVQGRPDRREGNPAPVRGYSVAGASRPLTGAEQLRRRLDAWRDQEERAS
MBA4075682.1	Cyanobacteria bacterium PR.023	MLQEDFLAAALPLFSNDEVEILEWSPDIGWATDIPEWADELLNFFSSTHSLYGHGVNYSPLSADWTAVDSGWMKNLERELEQRQFVHFSEHFGFSRLGELKQGAPLPVPHTAQALAAGQFKLAKLAHTTGGRIGLENLALAFSLSDVKEQGRFLDELLEPFDGFILLDLHNLYCQLENFGLSPEELLKTYPMHRVREIHISGGSWSQAINNKIRRDTHDNEVPDEVMALLPLVLAKCPNIEAVIFERLGGTMNTAEQQHRFRDDFRAIAKLIEQHWLEKNLADEALTAMEAIALVEPTKPAELKAGDFPDILRYQKKLMELLQSGQKPEEIIAKLSKDFDGEIADYASSIQPQMIAVGQELVAKWTR
MBA4064164.1	Isosphaera sp.	MDRADPAARPRVGLSLMPEPDFLRAAAPLFAAGAVEVVEWSFDTGWPPAAVPGWIDELLRHYGDAGRLLGHGVSFSPLSAGTGDAQRRWLGRLTEEVARRNYRHVSEHFGFSAAGDFHRSAPLPVPLTGPALRLGRDRLRRLADAAGVPVGLENLAFAFGRRDVDEQGHFLDALLDPVDGFLLLDLHNVHCQAVNFAVPFDDLLDRYPLHRVRELHLSGGSWSDGVRRDTHDGPVPEELFAALPGVLRRCPRVEAVVLERLGGTLAEADDEPFRRDFLRLAGCVRGAG
WP_182412767.1	Adhaeribacter radiodurans	MPQILSSIACNLDADILTAALPLFEESRVGAIEWSFDTLYGNSQIPPWFLELLKAYSNENKLIGHGVFFSLFSGKWSQNQHEWLNQLRQLSSEFHFDHITEHFGFMTGQNFHYGAPLPIPYSKTTLAIGQDRLARIYEASQCPVGLENLAFSYSLDEVKQHGEFLEQLVAPVNGFLILDLHNLYCQIHNFTISYEDIINLYPLDRVREIHISGGTWQDSTVEPERKIRRDTHDEAVPEEVFQLLKRTIDKCPNLKYVVLEQLGNALKTEASKKLFYSDFLKLEEIVRNKNHSLASHFTNSFLPSKSVTTGSILEDEKLYQQQIQLSSILEKASSYQAAIHLLNSSPLAHSDWKIEQWEPHMLETAISIAQKWKKNLD
WP_184174413.1	Rhabdobacter roseus	MPKVLSSIACNLDPNLLLAALPLFEAERVEAVEWSFDALYKYPTLPGWFEELIGTFAENDRLIGHGVYFSLFAGKWSKQQDQWLRQLRSLAARYPFDHITEHFGYMTGADFHKGAPLGVPYTSTTLKLGIDRLRRIQEAGQCPVGLENLAFAYSLDEVKKHGEFLEKLVGAVNGFIILDLHNLYCQLHNFRLDFEEVIRLYPLERVREIHISGGSWEPATTDDKYPIRRDTHDDAVPEAVFALLETTLPMCPNLKYVVMEQLGTALDTDARRLAFQRDFIRMDTLVKRLNDKLPQPATNDFGPLPTGLPTTLPLEDLSLHQQQRELSHILENAKSYPEARDLLAASSLRHSAWNVEAWVPSMLETARQIAQKWKNGFE
MBA3855477.1	Cyanobacteria bacterium PR.3.49	MRIKKPKLGLSLMPTEDFQMATERLFAEERVKSLEWSFDFTWNGVVVDQWAQELLDKYSSAKALTGHGVNLSPLSARFSKRQQEWLKLAKEEFKTRNYVHASEHFGFSEAGPIKHGAPLSVPMDDASLTTGKEMLKRYADATGCPVGLENLAFAFSVNDVKRQGQFIDELISSVDGFLLLDLHNTYCQIANFGVSELELLNSYPLEKVREIHISGGSWSPSVSGKRAAVRRDTHDDGVPQEVFNLAALALKLCPNIEFVILERLGNTMFSLEAQSEFRDDFESMEQILELCYA
MBA2612290.1	Bacteroidota bacterium	MNPWVGISLMPEADFIEAALPLFQSGEIDVIEWSFDTIRSGSKEPQWLSLLLSEYSKRGRLIGHGVRYSLLKGLWDGNQGKWLKQLAIEVKKYNYRHVTEHFGFMSSDDFHKGTPLPVPFNKTTLAIGQDRLKRLQNISQLPIGIENLAFAFSNKQIKEQGEFLEKLLEPVNGFLILDLHNIWCQSHNFKVDALKLIKSYPLHKVKEIHISGGSWATVKTSKNKVRRDTHDEAVPKEVFELLKEALPFCENVEYVIFERLGNTIKTEKDKLQFVKDFKQIKKIVSSQRKKAESPTEKIKRKKIDHKVPLKPLQDNILRKEQQFIIDTLFANKDVDKTIQLLQTKQITGWQPALWTDYMVETAMVLIKKWA
WP_172957645.1	Chryseobacterium indologenes	MRKPLLGLSMMPEAEFVSAALPLLQNNSVEVLEWSFDTLFNANEPDWLRDLLNFYAENNRLIGHGVYYSLFDAQWTERQEIWLSRLKDEVSKRNYNHITEHFGFMNTENFHQGVPLPVPLHPITLQIGQDRLQRLQDAVNIPVGIENLAFSFCLDDVKEQGVFLEKLIDNSGGFLILDLHNMYCQSCNFDVDIQEIIKLYPLQKVKEIHLSGGSWQESVYGKKMIRRDTHDHTIPEEIFAILPFVLSHCENLEYIIIERLGHTIKTKEEEGDFFSDFMRVKTIIDSSAGHERQGDLWIKKEFELSKYPVEDPVLYEEQSRLTKLLFDGTHVDSIKNENFHYFNTAGWDPEMIFTAQEIIKKWNPY
WP_317171826.1	Hymenobacter	MQATPTLCSALACNLDVDILSAALPLLAEGRVEALEWSFDALFWAGQIPDWFSELLRTYSASGRLLGHGVFFSLLSGRWSREQQQWLTQLRQLANQLPFAHITEHFGFFTGQNFHSGAPLPVPYTPVTLRLGQDRLSRLYEACQCPVGLENLAFAYALEEVKRHGEFLDKLLEPVNGFLILDLHNLYCQLHNFSISYEDLIDLYPLHRVREIHISGGSWEASALAPGQRVRRDTHDQGVPEEVFQLLQRTMPRCPHLRFVVLEQLGNSLRTEDSRARFRLDFGRMEALVQEHRAGLTASADQAFLPLRPPQPGPALEEPALHEQQQHLSRILETAPSVAEARQLLLASPLLASTDWNIEQWAPHMLETAVRIAQKWK
WP_184553106.1	Chryseobacterium sediminis	MKKPLLGISMMPEADFVSAVLPLLQSNAVDVLEWSFDTLYHTNEPDWLRDLLNFYAENNRLIGHGVYYSLFDARWTERQEEWLQKLKEEVSLRKYNHITEHFGFMNTENFHQGVPLPVSLHPKTLRIGKDRLYRLQQAVNIPVGIENLAFSFSIDDIKEQGVFLDQLTEDTNGFLILDLHNIYCQSCNFGVEMQEIIDLYPLDKVKEIHLSGGSWQESVYGKQIRRDTHDDIIPEEIFSVLPSVFAKCQNLEYIIIERLGHTLKTEDDKENFLNDFNNVKTIIEISDWKERGKSGWNKQPIQLPEKPVEDMALFEEQSRLTRLLFDGAGANAIKDQDFHYFKTENWDSEMILTAQNIIKKWNPY
WP_185274038.1	Adhaeribacter swui	MPEKILAAVACNLDVDILAAALPLFAAGKVEAIEWSFDTLFRTPQIPAWFSELLQTYGQAGRLIGHGVYFSLFSGQWSVNQQNWLQHLRELSGQYTFDHITEHFGFMTGQNFHHGAPLPIPYSASTLAIGQDRLARIQDACKCPVGLENLAFSYSLDEVKQHGQFLAQLITPVNGFIILDLHNLYCQLHNFRASYDEIVNLYPLDRVREIHISGGSWESSAIEPNRQIRRDTHDEAVPEEVFALLAQTIDKCPNLKFVVLEQLGNALKSEESKQLFYADFLKMEAILQRKNQKLRTNPAKDFLPLSLSIAEHPIEDKTLAAQQVHLSRILENASSAEEAIQRLQTSSLAHSDWQIETWEPYMIETAHNIARKWKM
WP_184160252.1	Chryseobacterium shigense	MKKPLLGLAMMPEAEFVSAILPLLQNNDVDVLEWSFDTLYDVKEPEWLSGLLDFYAENNRLLGHGVYYSLFDARWTERQESWLKKLKKEFKHRKYNHITEHFGFMNTENFHQGVPLPVPLHSKTLQIGKDRLCRLQDAVEIPVGIENLAFSFSVDDVKEQGEFLAELIEDIDGFLILDLHNIYCQSCNFEIDMQDIIRMYPLEKVKEIHLSGGSWQESAYGKKPVRRDTHDDNIPEVILNVLPEVLAQCNNLEYIIIERLGHTLDTEAKKHVFFDDFYRVKAIIDISDFSAGSNKVWNKKNKGYSGPVEDLLLYDEQTKLTKMLFEGNSVQAVKNEKFHYFKPENWDEEMIATAQQIIKKWNPY
WP_187319759.1	Hymenobacter citatus	MPAVPTAPAILSAIACNLDDQILTAALPLLAAGRVEAVEWSFDTLFNGQSLPNWFAELLTAYGEQSRLVGHGVYFSLLSGRWTPEQAQWLEQLRLLAGRFRFDHITEHFGSFTGQNFHTGAPLPVPYTATTLRLGQDRLLRVADACQCPVGLENLAFAYSLAEVQRHGEFLEELVAPVNGFLILDLHNLYCQLHNFNLSSDSLLALYPLHRVREIHLSGGSWEDSQVEQGRQIRRDTHDESVPDEVFELLERTLPQCPQLKYVVLEQLGTGLRTEASQHQFRQDFQRMAAIVEAGRGRVSGHQPNAFQPPASLPAGSVAYDEALHAQQRQLAQILETAASYEEAQQRLRASSLAHTSWQLEQWAPYMLETAISIAQKWKQ
WP_185133763.1	Chryseobacterium indologenes	MRKPLLGLSMMPEAEFVSAALPLLQNNSVEVLEWSFDTLFNTNEPDWLRDLLNFYAENNRLIGHGVYYSLFDAQWTDRQEIWLSRLKDEVSKRNYNHITEHFGFMNTENFHQGVPLPVPLHPITLQIGQDRLQRLQDAVNIPVGIENLAFSFCLDDVKEQGVFLEKLIESSGGFLILDLHNMYCQSCNFDVDIQEIIKLYPLQKVKEIHLSGGSWQESVYGKKMIRRDTHDHIIPEEIFAILPFVLSHCENLEYIIIERLGPTIKTKEEEDDFFSDFMRVKTMIDSSAGHERQGDLWIKKEFELSKYPVEDPVLYEEQSRLTKLLFDGTHVDSIKNENFHYFNTAGWDPEMIFTAQEIIKKWNPY
WP_185246801.1	Chryseobacterium	MKKPLLGLSMMAEPEFISAVLPLLQSNSIEVLEWSFDTFYNAQEPDWLSDLLHFYSENDRLIGHGVYYSLFDAIWNERQEVWLKKLKEEFHKRKYNHITEHFGFMNTENFHQGVPLPVSLHAKTLEIGKDRLYRLQDAVQVPVGIENLAFSFSLDDVTEQGIFLDKLTEDTDGFLILDLHNIYCQSCNFEVEIQDIIKLYPLDKVREIHLSGGSWQESVYSKSPVRRDTHDDAIPKEILSILPSVLSQCKYLEYIIVERLGHTINTEEKKIAFLDDFAQIRTLIDSSDRDQQYKAPWTKKQVEFQKNPLEDKVLHEEQTLLTKLLFNKEKPESIKNHEFQYFKTENWDPEMILTAQNIIKKWNPY
WP_185288143.1	Chryseobacterium lactis	MQKPLLGLSMMAEAEFVSAILPLLQNNSVEVLEWSFDTFYNVEETDWLNELLQFYSENNRLIGHGVYYSLFDARWTERQEIWLKKLKEEVRKRNYNHITEHFGFMNTENFHQGVPLPVSLHPKTLQIGRDRLYRLHEAVDVPVGIENLAFSFSIDDVKEQGMFLDQLVEDTNGFLILDLHNIYCQSYNFEVNIQDIISLYPLDRVREIHLSGGSWQESVYGKKPIRRDTHDDRIPKELLAILPSVLSQCQNLEYVIIERLGHTIKTEEQKKYFFDDFNKVKAIIEVSDTRSRKVNKWTGKEAVHTDHPEEDLALYEEQSRLTRLLFDQAGTEGIKEEKFHYFNTESWDPEMILTAQNIIKKWNPY
MBC8152492.1	Fibrella sp.	MSTIHSAIACNVDTNILLASLPLFDAEKIQAIEWAFDAVFRHDALPDWFDALLRTYADANRLVGHGVFFSLFSGRWTPDQQAWLTQLKTLAATYRFDHITEHFGFMTGADFHKGAPLSIPLTQSTLAIGQDRLRRIQDAGGCPVGLENLAFAYSLDEVKRQGDFLHRLLEPVNGFIILDLHNLYCQSHNFGLAGDELLALYPLEQVREMHISGGSWSASDSEPGRQIRRDTHDEAVPDAVFDWLEGAIDRCPNLRYVVLEQLGSGLTTEADQEQFRNDFLRMDRIVEGKNQVRAASQPNLFRNISGVLPSSMPLEDDDLHWQQRELATILETAADYRQARALLATSGLAHSAWEIERWEPAMLETAMAIAQKWQHGFD
WP_188686807.1	Rufibacter glacialis	MPKVFASLACNLDVDILSASLPLLQEGRVEGLEWSFDTLFQQEQVPSWFLALLETYGREKRLIGHGVFFSLFSGKWSEHQEAWLRHLTQMSAAYGFDHVTEHFGFMTGKNFHHGAPLPIPYTAATLAIGRDRLARIQEAAQCPVGLENLAFSYTLEEVKRHGAFLEELVEPVNGFLILDLHNLYCQLHNFSLPLEALLPWYPLHRVREIHISGGSWQDSAVLPQRKVRRDTHDSAVPEEVFDLLSATMPLCPQLKYVVLEQLGTGLKSPEDKALFYQDFCRMETLVKQHNASTFPSLTAPNLFLPLAPGKLPAPVEDEALYQQQLLLSSILEESPSLTQAHQRLLASSLANTAWAIEHWEPHLLETALHLAQKWKKPDLSA
WP_192218895.1	Microcoleus sp. FACHB-SPT15	MFKFKRRTPMVGLSLMIESDFFQAAQPLFETGAVEVLEWSFDMSWGVKAKPPWVKELLQFYSQRDRLTGHGISYSLLSAQPGERQIHWLKSLQEECLDYRYRHISEHFGWMAAGDFYQSAPLPLPLTPETLQLGREHLKRLSDKANAPVGLENLAFAFGLQDVLQQGEFLARLLEPVDGFLVLDLHNLYCQVHNFQQSAEQLLERYPLQRVRELHVSGGSWSVTSKGQQTRTIRRDTHNGTVPEAVFELLALALKRCPSVEAVIFERIGHTLHTEADIEGFRQDFWRIKQVVKESV
MBC7862316.1	Bacteroidia bacterium	LLQEYSANKRLIGHGVRYSMFDAKWTKRQSDWLSRLKKEVKKYQYNHITEHFGFMSNTDFHKGAPMPVPLNKSSLAIGIDRLKRMSAVAGVPVGIENLAFSFSAEDVKKQGLFLQKLVQPVNGFLILDLHNIYCQSKNFRIGMLKIIRAYPLKKVKEIHISGGSWQQSIYSTDLQKVRRDTHDGQVPEEIFKILPKVLKLCTNLEYIIFERMGNTMQDENEQLQFRKDFLRIKSVAGNVSSSASKRNGRNNWLKPRLSLDKKPITDSKLLSEQQHILEVMRTTENPYDALNKLNKEKLKDWQVLKWKPSMVETAIQLIRKWD
MBC8083770.1	Hymenobacter sp.	MEDEPILFSSIACNLDADILSAAFPLLAEGRVEAIEWSFDTLFWAEQIPDWFAELLSSYSSQNRLVGHGVYFSLLSGKWTKEQQQWLEHLQQLSAQFSFDHITEHFGFFTGQNFHSGAPLPIPYTSTTLRVGQDRLRRIYEACQCPVGLENLAFAYSLDDVKQQGEFLEKLITPVNGFLILDLHNLYCQLHNFSISYEELIDLYPLDRVREIHISGG

		SWEDSGLVPGKKIRRDTHDGAVPEQVFQLLEQTMPKCPHLKYVVLEQLGNGLQTQVSRIRFRNDFRTMTGLVGKWRNGLPRRSDQLFLPLQSFPAGPVAADAILH<br>EQQLQLSQILETAPSFEEARRRLYASSLAGTNWKIEDWEPHMLETAISIAQKWK
GGF13189.1	Hymenobacter cavernae	MEEAPIPFAAIACNLDFDILAAAFPLLEAGQVEALEWSFDTLFRAGQVPDWFSELLSAYSQQGRLIGHGVFFSLLSGKWTPQQQQWLQHLQQLSAQFRFDHITEHF<br>GFFTGENFHYGAPLPIPYTQASLRIGQDRLSRIYEACQCPVGLENLAFAYTPDEVKRHGEFLNKLLEPVNGFVILDLHNLYCQLHNFAVSYEELIELYPLERVREIHIS<br>GGSWEESEQAPQGKVRRDTHDEAVPEAVFELLARTLPRCPNLKYVVLEQLGNGLKTESSRTQFRHDFLRMEALVQKHRASLLGHPEHLFRPPQPLQLGEVIEDQS<br>LHEQQRQLSRILETAASVEEAQRRLHASVLVGTDWQMEQWEPHMLETAIHLAQKWKQ
MBD1867410.1	Cyanobacteria bacterium FACHB-471	MFKRKQNLPWVGLSLMTDPHFFTATRPLFEQGEVEVLEWSFDAGWEDTIPSWASELIDFYSQRDRLLGHGVTYSLLSAEWSDRQMRWLKQFQAECSRHRYVHIS<br>EHFGWMSAGNFARSAPLPMPYTPDAVSLGRDRLQRLADAAKVPVGLENLAFAFGQKDVADQGKFITELLEPVDGFLLLDLHNLFCQIHNFQLSIPELLASYPLARVR<br>ELHLSGGSWSRTTPDSNLVRRDTHDGAVPEAVFDLLAIVLKQCTNVKAVILERLGHTLNSEPEIEQFRQDFNRIKQIVKRCG
WP_190774526.1	unclassified Leptolyngbya	MPTPDFFQASQPLFEAGAVQVLEWSFDMGWEADLPDWVTHLLEFYSQRDRLLGHGVTYSLLSAQWEEAQDQWLKNLQEECKRYRYRHISEHFGWMAAGNFVQ<br>SAPLPLPLTADTLRLGCDRLNCLAAAAKVPIGLENLAFAFGQRDVEEQGTFLDQLLAHVQGFLVLDLHNLYCQIHNFQQSPEALLKRYPLHRVRELHISGGSWSKSA<br>SSDSMIRRDTHDGPVPEMVFELLAIALQHCSHVEAVIFERLGHTLSTETEQQQWRQDFQRIQQIVHQRRR
WP_190680896.1	Leptolyngbya sp. FACHB-671	MFKRKQNLPWVGLSLMADSHFLTATRPLFEQGEVEVLEWSFDASWEDTIPSWASELIDFYSQRDRLLGHGVTYSLLSAEWSDRQMHWLKQFQAECSRHRYVHIS<br>EHFGWMSAGNFARSAPLPMPYTPDAVSLGRDRLQRLADAAKVPVGLENLAFAFGQKDVADQGKFITELLEPVDGFLLLDLHNLFCQIHNFQLSIPELLASYPLARVR<br>ELHLSGGSWSRTTPDSNLVRRDTHDGAVPEAVFDLLAIVLKQCTNVKAVILERLGHTLNSEPEIEQFRQDFNRIKQIVKRCG
WP_190645960.1	Oculatella sp. FACHB-28	MFKRKQNLPWVGLSLMADSHFLTATRPLFEQGEVEVLEWSFDAGWEDTIPSWASELIDFYSQRDRLLGHGVTYSLLSAEWSNRQMHWLKQFQAECSRHRYVHIS<br>EHFGWMSAGNFARSAPLPMPYTPDAVSLGRDRLQRLADAAKVPVGLENLAFAFGQKDVADQGKFITELLEPVDGFLLLDLHNLFCQIHNFQLSIPELLASYPLARVR<br>ELHLSGGSWSRTTPDSNLVRRDTHDGAVPEAVFDLLAIVLKQCTNVKAVILERLGHTLNSEPEIEQFRQDFNRIKQIVKRCG
WP_191041552.1	Spirosoma validum	MPKIYSSIACNVDTNILQATLPLFEAEKVQAIEWSFDTLYQHNQIPDWFAELLQAYGQENRLIGHGVFFSLFAGKWTTAQQEWLDRLKKLASEFTFNHITEHFGFMTG<br>EDFHKGAPISVPFISSTLALGQDRLKRIQNACNCPVGLENLAFAYSLDEVKQHGDFLNQLIEPINGFIILDLHNLYCQSQNFSVAVSDLLSLYPLDRVREIHISGGSWTS<br>SSTEPEKRIRRDTHDEAVPMEVFELLEQVIAQCPNLNFVVLEQLGSGLITEESRRLFQEDFLRMDDIITTYTNTIPPENLFLPLVPFDISSSPLQNHLLHTQQTELSTILE<br>TATNYQQAQHLLRSSSLANSAWQVENWNSTMLETALAIAQKWQHGFN
WP_197901599.1	Rhodocytophaga rosea	MAKILSSVACNLDVHILSASIPLLEESQVDAIEWSFDTLFNIWHTPGWFARLLTAFSKEERLIGHGVFFSLLSGKWSKEQYQWLSQLKQLSAEFHFDHITEHFGFMTG<br>QSFHQGAPMCVPYSPATLAIGRDRLARIQEACSCPVGLENLAFAASLEQVKQQGEFLERLIEPVNGFIILDLHNLFCQMHNFFMDFEQLLSSYPLERVREIHISGGSW<br>EKSGKQSERIIRRDTHDQAVPPEVFHLLEKTIPVCPNLKYVVLEQLGIALKTQESKAAFYQNFVQMKQIVEDNNQTIANSSENPFIPPSITLPETFVENQELYAQQREL<br>SEILETSSSYEETNRLLKASSLAHTAWNIESWDPAMIETAIHIARKWKK
MBD2766342.1	Hymenobacter montanus	MEAGEVEALEWSFDALMGEMPEWFSTLLSVYGEQGRLVGHGVYFSLLSGQWTPEQQQWLHDLGQLAARFSFDHVTEHFGFFTGESFHAGAPLPVPCSAATLRL<br>GRDRLRRIQDAGRCPVGLENLALAYSLADVQQQGAFLDQLLEPVNGFLILDLHNLYCQLHNFAIAPETLIDLYPLDRVREIHISGGSWEASARAPRGRVRRDTHDGA<br>VPEEVFQLLERTMPRCPHLKYVVLEQLGTALKTERQRAGFRRDFRRMARLVQQHDRATTRGSDQLFLPLHPSPGGPVVVDEKLHGQQRQLSRILEAAPTLDEAQR<br>LLHGSSLAHTEWHLENWPPYMLETARRIAQKWKR
WP_193545348.1	Chryseobacteriu m culicis	MNKPLLGISMMAEADFVSALLPLLQSNSVDVLEWSFDTLYHNHEPVWLCDLLNFYAENERLIGHGVYYSLFDARWTERQEDWLKKLKEEVALRRYNHITEHFGFM<br>NTENFHQGVPLPVSLHAKTLEIGKDRLYRLQDAVNIPVGIENLAFSFGIDDVKEQGAFLDKLIEDTDGFLILDLHNIYCQACNFGIEIQEIMDWYPLDKVKEIHLSGGSW<br>QESVYGKRPVRRDTHDDSIPKELFSVLPSVVSQCQHLEYVIIERLGHTLKTDEEKENFLTDFNRVKKIIEASDWNGEQKKNWKKKEIKLTEKPLEDRVLFEEQSRLTQ<br>LLFDHVDAAVIKDVDFHYFKTENWDLEMILTAQNMIRKWNPY
WP_190796908.1	Leptolyngbya sp. FACHB-541	MFKRKQNLPWVGLSLMADSHFLTATRPLFEQGEVEVLEWSFDAGWEDTIPFWASELIDFYSQRDRLLGHGVTYSLLSAEWSDRQMHWLKQFQAECSRHRYVHIS<br>EHFGWMSAGNFARSAPLPMPYTPDAVSLGRDRLQRLADAAKVPVGLENLAFAFGQKDVADQGKFITELLEPVDGFLLLDLHNLFCQIHNFQLSIPELLASYPLARVR<br>ELHLSGGSWSRTTPDSNLVRRDTHDGAVPEAVFDLLAIALKQCTNVKAVILERLGHTLNSEPEIEQFRQDFNRIKQIVKRCG
MBE2206138.1	Saprospiraceae bacterium	MSPSIHTGIACNLDSNIITAALPLLEQEHVEAIEWSFDALFNWNEVPAWFRDLLQVFADAGRLTGHGIFFSIFSARWSAKQDLWLEQLRQLNRELPFDHVSEHFGFM<br>SGRDFHHGAPLSVPLTASTLAIGQDRLLRIQDACQCPVGLENLAFAFSLEEVRQHEAFLEQLLEPVNGFIILDLHNLYCQMKNFDLGFDALVHSYPLHRVREIHISGG<br>SWEPSTIIPTRNIRRDTHDDAVPEEVFEMLAQTIALCPALKFIFLEQLGAGLHTVSQQSQFRSDYLRICEIVKKHAPAVAQGDAESFSPRPNLQPPSAHIQDAVLYEEQ<br>RRLSRILETASDCQDARQKLESSILKNSAWQTEAWPDYMLETALRIAQKWKDGF
MBI1268694.1	Cryomorphaceae bacterium	MTVKPKLGLSLMSTPDFRQASAELFESGKVEVVEWSFDNAFNGAFIAPWCQNIVDRYSDANALLGHGVTLSPLSVRFSRRQQDWLLGAAEEFRRHNYIHASEHFG<br>FSEAGPVAHGAPLAVPMNSATLKSGKEMMKRFAEATRRPVGLENLAFAFCMEDVKRQGEFIDHLLSDVDGFLLLDLHNLYCQQLNFNIPAIELLESYPLARVREIHIS<br>GGSFSPSISGRRIFVRRDTHDEQVPQEVLELVVHALKRCPDIEYVIYERLGFTMEDEESQAEYREDFETLAQLLEMIYV
MBI1224055.1	Bacteroidota bacterium	MPFASIACNLDRNTLLAALPLLESGNVEGLEWSFDALFKVREIPEWFQELVFTYGKASRLIGHGVYFSIFSGKWRPEQENWLSQLRQFSKTYQFDHVSEHFGFMTG<br>EDFHKGAPIGIPYNASTLSIGVDRLFRIQDACQCPVGLENLAFSYSLDEVKKHGDFLSKLVEPVNGFIILDLHNLFCQMHNFEVDFEELMALYPLERVREIHISGGSWE<br>ASNIVSNKKIRRDTHDSQVPETVFKMLESSLSRCPNLKFVVMEQLGNALGGDAEKAQFQADFLKMSSIIQHETPGNFTQQNDFSPNAARILGEPLENEFLAYQQNEL<br>SQILETSPNFETTRQLLANSSLANSDWKVENWEPTMLETAMVIAQKWKHGFV
MBJ6143402.1	Hymenobacter sp. BT559	MLAALACNLDADILSAALPLLEAGQVDALEWSFDALYWASEVPAWFTELLHAYSSEQRLLGHGVYFSLLSGRWTSEQQQWLCQLRELAQRYSFAHVTEHFGAFTG<br>QNFHAGAPLSVPYSPVALRIGQDRLARLAEAARCPVGLENLAFAYSLDEVKRQGDFLSALLAPVNGFLILDLHNLYCQLHNFSIDYEELLGCYELDRVREIHISGGSW<br>ENSQLLPTRRIRRDTHDEAVPTEVFELLARTLPRCPNLKYVVLEQLGTSLKTEASQVQFRRDFAQMAALVQQHNHAPRSVPSAFLPRWPALPALAVQDDLLHEQQ<br>RQLSHILETATSLPEAQQRLRASVLAHSAWQIEHWEPYMLETALSIAQKWK
MBK6512547.1	Polyangiaceae bacterium	MGLAARGVRQGVSHPSGAPPRVGLSLMPADDWWAANEPLFFRGLIDAVEWSVDFGWGPDGVPEWLASLLERYGSQDRLYAHGVELSAMSAIWTPEQDAWLAE<br>LEATCARSRFRHLTEHYGFITAADFVRGTPLPLPPSAAALGLAERRLQTLRERAGVPVGIENLAFALSRRDVEAQASFVSQLLERTDAFLLLDVHNLYCQAHNFGIDP<br>LVLARSYPLHRAREIHVSGGSWSTPTSDPERRRFRRDSHDDRTPNEVFSLLSSVLELCPALEVVILERTDRSLFGRDEAERHREDFVRLRSLVHRRGQRGQAEPP<br>SGSRLPRVELARDDEVALEAFQGALVTTLASQADPHEVKAALQAAAPLAPYREHLDSYEPRALEIGAALVKQWGARAEPEDSMRAVVFRGPGSPLELRSLPRVAP<br>GPGQVLVRVVAVGLCGTDAHAYRGSFPVPTPIVLGHEIAGVVEALGSGVTDLEVGDRVGVSWVQAGCGACAACDRGAFQRCASPRTWIENGGGLSELTVAEASG<br>CTRLPDGLDPELAAPLFCAGHVAFSGLRKAAVRGAERVAVVGLGGLGHLALQIASALGHETLAVSSSLDKLEDAREGFGADHVVRVDDGDVGGALERAGGADIVL<br>ATTSNMSDVARSVRGLRLGGRVVLMGLGEGLLSIDPIELVQREASVIGSVQGSRAELEELLTLAAEGKVRPRVEVFPLLMAQRALARLLEGRVRYRAVVRVG
MBI3238826.1	Flavobacteriia bacterium	MKNQNGILASVACNLDQQLLQATLPLFIQEEVEAIEWSFDTLYKVRNIPDWFVELLTAFGNEGRLIGHGVFFSLFSGKWSAEQEQWLHHLKKVSGNFTFDHITEHFG<br>FMTGEDFHKGAPIGIPYNANTLALGIDRLKRIQGTCQCPVGLENLAFSYSLEEVKRHGEFLEALIEPINGFIILDLHNLYCQLHNFSLDAAELLPLYPLHRVREIHISGGS<br>WEQTRLEPQRTIRRDTHDDAVPEAVFELLQLTIPRCPNLKFVVLEQLGTALDTNQNQLQFQSDFMRMKSIVSTFNTADSINNFLPSSPLILNPEVAEDTLLALQQQEL<br>SAILENATDYENAVALLRQSGLAHSDWNVEEWSPYMLETAMRIAQKWKHGW
MBK7839075.1	Candidatus Obscuribacter sp.	MATEQLFAQDKVDALEFNFDQPLNGIVLAPWCQELLDKFSSASALIGHGVNLSLLSAQFTKRQQDWLTYTREEFKTRRYVHASEHFGFSEAGPIAQAAPLAVPMDA<br>QSLSVGKEMLKRYAEATQCPVGLENLAFAFSLDDIKKQGDFIDQLISSVDGFLLLDLHNIYCQVTNFDLDALELLHSYPLSKVRELHLSGGSWSPSLSGKRIAVRRDT<br>HDEAVPQEVFNLTALALKLCPNTQFVILERLGNTMLEPELQAEHRDDFDTIREILDYSYD
MBK8443732.1	Sphingobacterial es bacterium	MPSIHVGLSLNLDGNLLSAALPLLQKGEVEAFEWSFDALYHRAATPLPGWFEALLDTFSKEHRLIGHGIVFSLFSGKWLPEQNLWLQQLSRIARRYNFAHVTEHFGF<br>MTGEDFHKGAPLGVPLTAATLRLGQDRLCRLQDACSCAVGLENLAFAYHIDEVKKHGDFLQQLVQPVNGFIILDLHNVYCQSVNFNIPFAELLSLYPLHLVREIHLSG<br>GSWAQVAHKTVRRDTHDAAVPAAVWDLLQTALPLCENVQWVILEQIGISLNTSEAQQLFQNDFVRMSQCVAQYCTLQNPAASPVKNFAPPLLSQIPSEIPEDDDLY<br>QQQMELSRILETAPDVEQARALLGNSVLAHSAWAVEKWDNAMLATAMQIAQKWKDGFERFFK
MBK9205492.1	Candidatus Obscuribacter sp.	MATEQLFAQDKVDALEFNFDQPLNGIVLAPWCQELLDKFSSASALIGHGVNLSLLSAQFTKRQQEWLEYTREEFKSRRYVHASEHFGFSEAGPIAQAAPLAVPMDA<br>QSLYLGKEMLKRYADATQCPVGLENLAFAFSLEDVKRQGEFIDQLISSVDGFLLLDLHNIYCQVANFDLDALQLLHSYPLSKVRELHLSGGSWSPSLSGKRIAVRRD<br>THDDAVPQESLILLLLR
MBK9142105.1	Candidatus Melainabacteria bacterium	MVGKIESRHVSRTSPLVGLSLMPTEDFRDFAWNLVDREMVDVIEWSFDMIWEDGEMTPSWCESLLDDFSARGRLLGHGVTFSPFSGGFSEHQKWWLEQLRVEC<br>DRRRYIQISEHFGFMEAGAFVESAPMPVPMTEGAISLGRRCLEALAQSSGRPVGLENLALAFCLEDVRWQGKFLEEVLSRVDGFLVLDLHNLYCQSVNFDVPMSEL<br>LGSYPLERVRELHISGGSWSARRSGTGPSIRRDTHDGPVPERVFEILPEALDRCPALEYVIFERLGGTIDETGGTEELIRDYERILETVDG
MBP7863127.1	bacterium	MPEIKVGLHAMPTDDFRQAATELILSDQVDVLECTFDAHWQLAENKISDYQYPDWYQSLIQVYSDENSLLGHAILLSILSAKTSARQLCWLEKLERECIERNYLHVSE<br>HFGFFEADGFIDGAPLPVPYTKDAVMIGRAMLKKMAQSSNKDVGLENLALAFSKTEAQEQGAFLNDLLDKPNNFLLLDLHNIFCQIANFNLNAKDHLSLYPLHKVREI<br>HLSGGSYSKSWSGKRDAIRRDTHDGPVPAEVFDLLDLALTLCPNLEFVILERLGNTMDGEDDFAQFRADYKRMYDLARGK
MBK7746242.1	Candidatus Obscuribacter sp.	MATEQLFAQDKVDALEFNFDQPLNGVVVAPWCQDLLNKFSSASALIGHGVNLSLLSAQFTKRQQDWLTYTREEFKTRRYVHASEHFGFSEAGPIAQAAPLAVPMD<br>AQSLHLGKEMLKRYADATQCPVGLENLAFAFSLEDVKRQGEFIDQLIASVDGFLLLDLHNIYCQVTNFDLDALELLHSYPLSKVRELHLSGGSWSPSLSGKRIAVRR<br>DTHDDAVPQEVFNLTALALKLCPNTQFVILERLGNTMLEPELQEEYRDDFDTIREILDYSYD
MBK9070561.1	Myxococcales bacterium	MIDAAAARPNSRQIKVGLTLMPSKDWLVAAKPLLIGNYVEALEWSWDVETTQPPWALGLIEYFAGQGALYGHGVMYSAFSAEFEPRQQAALEKTATLRQLASAVHI<br>TEHVGFSTIKGMLRGAPLPSPPCRASVQTARDQLGRIADAVGRPVGVENLALAISMDDVALQGDMIEEILVASDAPFVLDLHNLYCQAINFSIPAEQLLRALPLHRVR<br>EIHVSGGRMHAVAGGATKRRDTHDQLVPEDVWTMLRQCLPQCPRVEVVFLERLAGTLQGQADELFLDFQRMKREVDTFLTASSDLHAGREFGDEARVTRQPLPH<br>PSEAHELAAYQQAFVGLLRKLPSVVTAHHASELRTALAAATPSFAGYAISINDDMLIIAHAAFSRWHKVF
MBK8564694.1	Saprospiraceae bacterium	MLLPSIACNLDKNLLQTALPLFESGKVEGLEWSFDALFKRPNIPDWFQELLLAYANEGRLIGHGVYFSLFSGKWMPEQAEWLTQLRQFAKAYRFDHVTEHFGFMTG<br>EDFHKGAPLSIPFTNSTVAIGIDRLKRIQDACNCPVGLENLAFAYGLEEVKKHGEFLEKLIEPVNGFIILDLHNLFCQLHNFEVGFDEILALYPLQRVREIHISGGSWEDS<br>MVAPNRKVRRDTHDEPVPETVFDLLEKTIGRCPNLKFVVLEQLSNALNTEEEQLAFRQDFWKMNHIIVQHNAPIIAPTNDFMPMERLMLASQPAEDLLLAEQQAELS<br>NILETAANYEDGHQRLLNSTLENSAWQVENWNPAMLETAIAIARKWKQGF
MBK9447954.1	Bacteroidota bacterium	MIHASLSFNVDSNLIQAALPLFAAEAVEGMEWSFDTLHNRPDIPEWFEELLKSYGNAGRLVGHGVFFSMFSGKWLPEQAAWLAELKSLTTRFRFDHISEHFGFFTG<br>ADFHKGAPLSVPYSKRTLALGQDRLLRIADACGCPVGLENLAFATDAHSARIHGEFLEALLAPANGFLILDLHNLYCQLHNFGLDSAELLAGIPLARVREIHISGGSWE<br>PSPFRPQPGVRRDTHDAGVPERVFELLATTMGQCPNLKFVVLEQMSHALADAESQSQYQADFLRMRDLVQLESAKRTSFTLLQNFLPPKIEFQSHPLEDETLHFE<br>QLLLSDILENSANFEEVKAKIQSSALASGEWSAVNWDPAMLETARQIARKWKDGF
MBK9619416.1	Candidatus Obscuribacter sp.	MQTNKPKLGLSLMPTEDFRMATEQLFAQDRVDALEFNFDQPLNGVVIAPWCQDLLDKFSSASALIGHGVNLSLLSAQFTKRQQEWLEYTREEFKSRRYVHASEHF<br>GFSEAGPIAQAAPLAVPMDAQSLYLGKEMLKRYADATQCPVGLENLAFAFSLEDVKRQGEFIDQLISSVDGFLLLDLHNIYCQVANFDLDALELLHSYPLSKVRELHL<br>SGGSWSPSLSGKRIAVRRDTHDDAVPQEVFNLTALALKLCPNTQFVILERLGNTMLEPELQEEYRDDFDTIREILDYSYD
MBL9023440.1	Myxococcales bacterium	MTPKVGLSMLLEDDLRLAQAPLFAEGIVEAIEWTIDCELGRATPPWVEPLLDHYAAAGALYGHGVHFSPLSAIFEPRQAAWLEHAKGALAKRRYQHVSEHFGFMTT<br>PGMTRGVPLPVPYTKGALELGRDRLARLRAATSGAPVGLENLALAMSRDDALVHGEFLDALLEPHDGFLLLDLHNLYCQAHNFDVPWGTLLGAMPLDRVREIHVS<br>GGSWYEVATPAGPQPFRRDTHDDAVPDEVFGLLELALPRCPGVEVVFLERLGGTLADASAGEAYREDYRRLVSVVSRFAKGAAAHG
MBL0015158.1	Bacteroidota bacterium	MIHASLSFNVDSNLIQAALPLFAAEVVEGMEWSFDTLHNRPDIPEWFEELLKSYGDAGRLVGHGVFFSMFSGKWLPEQAAWLAELKSLTTRFRFDHISEHFGFFTG<br>ADFHKGAPLSVPYCKRTLALGQDRLLRIADACGCPVGLENLAFATDAHSARIHGEFLEALLAPANGFLILDLHNLYCQLHNFGLDSAELLAGIPLARVREIHISGGSWE<br>PSPFRPQPGVRRDTHDAGVPERVFELLATTMGQCPNLKFVVLEQMSHALADAESHSQYQADFLRMRDLVHLETERRSPNRILRDFLPSTIRLPKKPLEDTMLYSEQ<br>MLLSQILENSASYDAANAAIQSSALASGEWSAVNWDPAMLETARQIARKWKDGF
MBK9771754.1	Candidatus Obscuribacter sp.	MQTNKPKLGLSLMPTEDFRMATEQLFAQDKVDALEFNFDQPLNGVVIAPWCQELLDKFSSASALIGHGVNLSLLSAQFTKRQQDWLTYTREEFKTRRYVHASEHF<br>GFSEAGPIAQAAPLAVPMDAQSLSVGKEMLKRYAEATQCPVGLENLAFAFSLDDIKKQGDFIDQLISSVDGFLLLDLHNIYCQVTNFDLDALELLHSYPLSKVRELHL<br>SGGSWSPSLSGKRIAVRRDTHDEAVPQEVFNLTALALKLCPNTQFVILERLGNTMLEPELQAEHRDDFDTIREILDYSYD
WP_202092815.1	Chryseobacteriu m endalhagicum	MKKPLLGLSMMPEPEFVSAILPVLQNNSVDVLEWSFDTLFDSEEPEWLSGLLDFYAENGRLLGHGVYYSLFDARWTERQEIWLEKLKKELQLRKYNHITEHFGFMN<br>TENFHQGVPLPVPLHPKILQIGKDRLRRLQDAVEIPVGVENLAFSFSVDDVKEQGAFLSQLVEDIDGFLILDLHNIYCQSCNFEIDMREIINMYPLEKVKEIHLSGGSW

		QESAYGKKPVRRDTHDDRIPEAVFEILPEVLSQCENLEYAIIERLGHTLNTETDQQIFFDDFKRVKEIADAAGNSSGQENIWNKKEEGAHVKPVEDLLLFDEQTKLTQ<br>LLFEERSSQSVKDQDFHYFKPENWDEEMITTAQQIIKKWNPY
MBL7912957.1	Bacteroidia bacterium	MLPWVGISLMPEADFIEAALPLFQNGEVDVIEWSFDTLRSNSKEPQWLSLLLNEYSKNGRLIGHGVRYSLLKAQWDSNQEKWLKQLSTAVKKYNYRHVTEHFGFM<br>SSDDFHKGTPLPVPLNKTTLAIGQDRLKRLQNISQLPVGIENLAFALSTKQVKEQGEFLKKLLEPVNGFLILDLHNIYCQASNFKIDPIELVKSYPLDKVREIHVSGGSW<br>ADVSTSKNKVRRDTHDEAVPKEVFELLKSALPLCKNVEYIIFERLGNTIKTEKDKLQFAKDFKKIKKIVATQKKNENSPSERLTKKIIKHTVAKEPLESKVLRKQQQLIIE<br>TLLKHKDVAKATKVLTKNKALKDWDPALWKNYMVETAMVLIKKWN
WP_202271905.1	Chryseobacterium sp. KMC2	MGRPLLGLSMMAEADFVSAILPLLQDNAVDVVEWSFDTFFAAEEPAWLKDLLNFYAGNGRLLGHGVYYSLFDARWTERQEMWLQKLKHEVQKRNYNHITEHFGF<br>MNTENYHQGVPLPLSLHPKTLEIGKDRLYRLQDAVSIPIGVENLAFSFSVDDVREQGVFLDKLVDDIDGFLILDLHNIYCQSCNFDVSIQEIVSLYPLEKVKEIHLSGGS<br>WQDSVYGKKMIRRDTHDDVIPEEIFSVLPSVLSQCRNLEYVIIERLGHTIHTEEEKQRFFNDFVRVKSILNDSDGMKRKKEKWVKREMLLPEPVEDSILYEEQIRLTKL<br>LFDHTDPTLIKNQNFHYFKTENWDPEMILTAQNIIKKWNPY
MBL7922055.1	Bacteroidia bacterium	MNPWVGISLMPEADFIEAALPLFQNGEVDVIEWSFDTIPPKVKEPEWLKLLLKEYSQNGRLIGHGVHYSLLKAVWDSNQDKWLKQLATEVKKYNYRHVTEHFAFMT<br>SDDFHKGTPLPVPFNKTTLAIGQDRLKRLQNVSQLPIGIENLAFAFSHAQIKEQGEFLKKLIEPVNGFLILDLHNIYCQSHNFKVDVVKLVNSYPLDKVKEIHISGGSWA<br>NVESSKNKVRRDTHDEAVPEEVFELLKEVLPLCKNVEYVIFERLGNTIKTQKDKLQFAKDFRQVKKIVLSQRKKEENLLEEITKTKIDHKVSLNPLEDENLRKQQQFIIE<br>TLLVNKDVAKTIQLLQNKKINSWQPELWTDYMVETGMVLVKKWN
MBL7782349.1	Saprospiraceae bacterium	MLYPSIACNLDSDILLTALPLLESERVQGLEWSFDTLYQVRDIPAWFDELLLEYGRAGRLVGHGVFFSLFSGRWSPEQAQWLKRLKKQCDIFHFDHITEHFGFMTGA<br>DFHSGAPISLPFTASTLAIGHDRLRRIQEVCACPVGLENLAFAYSVEEVKRHGDFLNKLMEPVNGFIILDLHNLYCQAHNFDIPADELLPLYPLDRVREIHISGGSWEN<br>SSLLPDRPVRRDTHDDAVPQEVFQLLKNTIPLCPNLKFVVLEQMGTALHTEASRRQFRQDFDTMHTILEAYESPDNYSGNAFIPLPFPALGAPLEDADLHRQQRQLS<br>EILEKAGSFEEAQRLLAVSDLAGTAWGTEHWAPYMLETATRIAQKWQKGFRQPE
MBL7682615.1	Flavipsychrobacter sp.	MLQACYPLFEASKIEAIEWSFDALYKRRNIPDWFLQLLHAFANEGRLIGHGVFFSLFSGRWTYEQQAWLKHLSKVSSAFRFDHITEHFGFMTGADFHSGAPLSIPYT<br>NTTLTIGKDRLARIYDACGKPVGLENLAFSYDIDEVKRHGDFLSKLIEPVNGFIILDLHNLYCQLQNFSVGVDEIINLYPLERVREIHISGGSWEDSIIDIGRKIRRDTHND<br>AVPQEVFALLEYAIDKCPNLKYVILEQVGGALHTQQSREDFAADFLKMSAIVKQAKGLPPETNDFLPKELIIPALVAEDEELHQQQTELSEIFETAAGYDDALMRLQQS<br>SLANSDWQIENWAPYMLETAISIAQKWKK
MBL7707133.1	Taibaiella sp.	MLSASLPLLEAGLVGGLEWSFDALYARDYQVPDWFHELLSAYSKEQRLVGHGVFFSLFSGRFSEEQQAWLERLRALRGMYQFDHITEHFGFMTGRDFHKGAPLS<br>VPYTATTLAIGRDRISRIADAAQCPVGLENLAFAYDLEEVKIQGRFLDELLSPVNGFMILDLHNLYCQLHNFNLDSSTLLDLYPLEKVREIHISGGSWQPHPGAHLQQV<br>RRDTHDDAVPAAVFELLKITLNRCPNLKFVLLEQLGIALTTPAQRAQYQDDFHTMRNIIEQYNREQQWPRNIDGFRPATLQLTPTPLVDEQLFGHQQIISHILETAADH<br>KDVIDKMQYTSLAGSDWHIEKWQPAMVDTAIHIAQKWK
WP_204662631.1	Dyadobacter sandarakinus	MSEIYSSVVCNLDMHMLRASLPLFEQEKVEAIEWSFDTLFRVEEVPAWFTNLVSEFSNHHRLIGHGVYFSLFSGKWAPEQHDWLIKLRQLSREFHFDHITEHFGFM<br>TGEDFHKGAPISIPFTSTTLALGKDRLRRIQDACQCPVGLENLAFSYSLEEVRKHGDFLGRLVESVNGFIILDLHNVYCQVHNFNIPFDMLIELYPLDRVREIHISGGS<br>WDETLAGPGKPVRRDTHDDYVPEVVFDFLNKAIPKCPNLKYVVLEQMGTALSTPADQLAFQSDFLRMDNIVKSWAPKMLQSPADTFAPIPGIASATPLEDPELHGQ<br>QMQLSRILETAGNAGEARSMLLSSSLHNTAWEVENWAPYMLETAISIAQKWKNGFA
MBN8684453.1	Chitinophagales bacterium	MESERVEAIEWSFDALYSFEQIPEWFTDLLSEFSQAGRLIGHGVFFSLFSGKWLPEQSQWLESLRQLSQAHHFDHISEHFGFMTGADFHRGAPLSIPFTPGTLAIGQ<br>DRLKRISEACGRPVGLENLAFAYSLDGVRRHGAFLDELLEPINGFLIFDLHNFYCQLHNFEQDVDTLIRNYPLKRVREIHISGGSWEISEARPERPVRRDTHDHAVPE<br>SVFELLEMVMPRCPNLKYVVLEQLGPALNSEKERLDFQADFFKMEQAVQNFNRQQQGREIHDFLPMKYTLNAVPLEDEALYQQQIQLTDILENAGDLKSAQAQLIA<br>CGLADSDWQARYWAPEMLDTAINIARKWKNGF
MBN9380196.1	Chitinophagaceae bacterium	MEGSRIDALEWSFDALYREEEIPDWFRDLLTAFANEHRLIGHGIFFSLFSGRWLAGQEEWLHQLRRVCQDYRFDHITEHFGFMTGKDFHHGAPLGVPYTPTTLRIG<br>RDRLKRIYDACRCPVGLENLAFSTSAEEVKRHGVFLEQLLEPVNGFIILDLHNLYCQLHNFGIAFEDLIDLYPLHRVREIHISGGSWDNSKANPDRTVRRDTHDNAVP<br>QEVFRLLENAIERCPHLKYVVLEQLGVGLQTAAARKTFYDDFLKMQSIVQEKSKTLDPGTVDTFLPGSVLLTEIKAEDETLHRQQLELSRILETAVSYEEVIHRLNNSS<br>LVSSDWKIEAWEPCMVETAMKIAQKWMRGV
MBN9166157.1	Myxococcales bacterium	MHPWIGLSLMLEDDFRLAAGPLFAEGVVDVLEWSFDTGWGRAIPEWADALLDHYGEAGRLLGHGVHYSAFSARWEERQARWLERLAGEVARRTYVHVSEHYGF<br>MTAAPFMRGAPLPVPRGEASRSVGRDRLERMRAVLAAGRNDACPIGLENLALAWNRDEALAHGAFLGEVLDHAEDFIVLDVHNLHCQIENFDIDPDELLDSFPAAR<br>IRELHVSGGSFLPAWPHHPEETVRCDTHDGDVPAPVFDLLERALDRFPDVRAVILERLGGTLRTQADIDTFRRDYLRVKQIVDHSRDGEGARDGDG
WP_207426252.1	Pedobacter sp. SYSU D00535	MKTVYSSVACNLDRDILAATIPLLAESRVEAIEWSFDALYKIAEVPDWFQELLHTYSKENRLIGHGVFFSIFSGRFLPEQKEWLKQLRQTASHLKFDHITEHFGFMTGP<br>NFHHGAPLNIPCNEKTLAIGRDRLARIQEACHCPIGLENLAFAFSLDDVQRQGAFLEKLVEPINGFIILDLHNLFCQLHNFQLDFDQLIQLYPLHRVREIHISGGSWESS<br>EHLTNRQIRRDTHDDLVPEEVFQLLELAIPKCPELKYVVMEQVGAGLKTAESKTGFYADFLKMEAIVKRAVQLEGAAPVNRFLPEDILLEAQPVEDEALYQQQLELSE<br>ILETSSSYAETYSRVQHSSLSKSDWNIESWKPWMLETAVALANKWK
WP_208173464.1	Hymenobacter negativus	MDETPPILASLACNLDSDILTAALPLLEAGQVEALEWSFDTLFRANELPEWFTELLHAYGNQQRLVGHGVFFSLLSSRWTPEQQHWLTQVRQVSATFAFDHITEHF<br>GFFTGQNFHSGAPLPVPYTATALRIGQDRLRRIYDACQCPVGLENLAFAYSLEEVEQHGEFLEKLIAPVNGFIILDLHNLYCQLHNFSVSYEALIDLYPLDCVREIHISG<br>GSWDNSAVLPHKKVRRDTHDDAVPEEVFRLLALTMPKCANLKFVVLEQLGTGLQSESSKAQFQRDFQRMANLVQQHRTRFLNHELANTFRPLQLPVLGSAIEDSQ<br>LHTQQVQLSDILENAATFEEAHQRLHASSLANSAWKIEAWEPHMLETAVRIAQKWKK
MBO3269797.1	Hymenobacter defluvii	MPSILSAIACNLDDHLLAAALPLLEAGQVEAVEWSFDTLFDGRQLPNWFAELLTAYGEQGRLIGHGVYYSLLSGRWTPEQAQWLEQLRLLAARFHFDHITEHFGSFT<br>GQNFHTGAPLPVPYTAATLRLGQDRLLRVADACQCPVGLENLAFAYSLEEVQRHGEFLEKLLAPVNGFLILDLHNLYCQLHNFNLSTDSLLALYSLHRVREIHISGGS<br>WEYSQVEQGRQIRRDTHDEAVPDEVFALLERTIPQCPQLKYVVLEQLGTGLRTEASQHQFRQDFQRMAAIVEASRHRAPSHQPNAFQPPAALPTGPAAHDEALHA<br>QQRQLSQILETAASYEEAQQRLRASSLAHTDWQLEQWAPHMLETAISIAQKWK
WP_207177520.1	Cellulophaga sp. E16_2	MRKRIMGNKKPKLGIAIAPSQKFLEAALPLFAEEKIEIIEWSFDTLKDAADEPAWLSLLLKEYGEKNRLLGHGVFYALLDANWSSRQENWLKKVRQETLSHKYCQISE<br>HFGLMSSANAHSGFPLPIPLSNPVLQIGIDRLKRLQATAQVDVGIENLALTANVADILEQGEFLFKLVHPVNGFVILDLHNIYCQSENFNIDMMTIIKSYPLSLVKELHIS<br>GGSWDTDPTLSRKIRRDTHDGRIPEVILEVLPEVLKICPFLAFIIFEKIEDSFLTEKDGIDFRADFQKIREIIDATSFSVTPKEKKQVAIILGPPVIDVELLQAQVALRESIAL<br>DTYQTNSEWDKDMWKVATKLYEKWNN
WP_207331299.1	Fibrella forsythiae	MIRSTIACNLETNLILASLPLFEAERVEALEWSFDTLYAHQQLPDWFVDLLTTYSEAGRLIGHGVFFSLLSGRWTHDQQNWLARLHALSRDYQFDHVTEHFGFMTG<br>EDFHKGAPINIPCTPVTLALGQDRLKRIQDACGCPVGLENLAFAYTLDDVKRQGDFLRQLIQPVNGFIILDLHNLYCQSHNFSIMHDDLMALYPLDRVREIHISGGSW<br>SGSETQPGLLIRRDTHDDAVPDDVFNWLSTTIDQCPNLKYVVLEQLGNGLTTEGQQQAFRADFHRMDTIVQEKNKPATTTTMNSFLPQLPLIINAMPLENHRLHNQ<br>QVELSAILESSTDVSQAQTRLAQSSLANSEWEVERWEPSMLETAVSIARKWKDGFVSKK
WP_207434928.1	Sabulibacter ruber	MPNLLPSLACNLDLDLLAAALPLLEEARVDGLEWSFDALFQTRQVPAWFLDLLTAFGQEKRLVAHGVFFSLFSGKWSEGQRQWLKTLSEASQRYRFDHVTEHFGF<br>MTGQNFHHGAPLAIPYTATTLAIGQDRLARIQEAAQCPVGLENLAFSYSVEDVKRHGEFLEKLIEPVNGFIILDLHNLYCQLHNFSVSFEELIPFYPLERVREIHISGGS<br>WEDSLTTPAKKIRRDTHDNGVPEEVFNLLTQTLPLCPNLKYVVLEQLGNSLKTEQSKTQFYQDYLRMEAIVRDFNATHLNPIPEPERFLPPQATFSAELVEDEALYA<br>QQVELSSILETAGSYEEAQQKLRTSTLAHSAWQVEQWEPAMLETAISLAQKWKKK
WP_291898394.1	Chryseobacterium sp.	MKRPMLGLSIMAEADFVSAILPLMQNNSIDVLEWSFDTLYHTHEPDWLRDLLNFYAENGRLIGHGVYYSLFDAKWTERQEEWLQKLKEEVRLRKYNHITEHFGFMN<br>TENFHQGVPLPVPLHSTILQIGKDRLCRLQDAVNIPVGIENLAFSFSMDDVKEQGIFLDRLTEDTNGFLILDLHNIYCQSCNFEVGMQEIINLYPLDKVKEIHLSGGSW<br>QESVYGKKQVRRDTHDDVIPQDILSILPSVLKQCQNLEYIIIERLGHTLKTEQEKVDFLNDFNTVKMIIETSELKLREGSTWSKKEMKLSEKPLEDLVLYEEQSRLTKLL<br>FDKAGSEVIQGQDFHYFKTEKWDPEMILTAQNIIEKWNPY
WP_291193020.1	Dyadobacter sp.	MSEIYSAVACNLDTHILRAALPLFEQEKVEAIEWSFDTLYKFDEIPPWFTDLVSEFSKQNRLMGHGVYFSLFSGKWSSGQQEWLRKLKKLSAEFNFDHITEHFGFMT<br>GEDFHKGAPIGIPLTPRTLALGQDRLQRIQDACQCPVGLENLAFSYSIDEVRKHGVFLDQLVESVNGFIILDLHNIYCQVHNFNIDFVDILKLYPLNKVREIHISGGSWE<br>DTATPQGKPVRRDTHDESVPEIVFEFLGNAIPQCPNLKFVVLEQMGTALSTYASQAAFQSDFLRMDSIVKSLARKFDPIPERMFAPLAASLNAIPLEDADLYQQQIQL<br>SYILETAGNVSEARAMLLASDLRNTAWEVENWAPYMLETAISIARKWKNGF
MBO9594034.1	Niabella sp.	MAEIHSAIACNLDADILSASLPLFESGSVDAIEWSFDALHQRQELPEWFRALLTAFSRSNRLIGHGIFFSIFSGKWLPEQEQWLKSLKKYAADFHFDHITEHFGFMTG<br>KDFHHGAPLSIPFSPVTLAIGIDRLQRIQEACRCPVGLENLAFSYSLDDVKRHGAFLEKLVEPVNGFIILDLHNLFCQTHNFNVDFEPLLACYPLDRVREIHISGGSWE<br>PSVAVPSTIIRRDTHDEKVPDTVFDFLRTTIPRCPNLKFVVLEQLGTGLRSEASRVAFRDDFLQIQRIVKAASLRATSINTFQPPSFQLRTTPVEDSLLYRQQTALSRIL<br>EEAGSEAEARSQLEASVLAHSDWQIEHWNAAMLETAVHIAQKWKNGFTPDA
WP_209917743.1	Chryseobacterium jejuense	MGRPLLGLSMMAETEFVSAILPLLQSNSVEVLEWSFDTFYDAEEPDWLSDLLDFYSENNRLIGHGVYYSLFDARWTKRQEIWLKKLKEEFRKRNYNHITEHFGFMN<br>TENFHQGVPLPVSLHSKTLEIGKDRLYRLQDAVQVPVGIENLAFSFSLDDVMEQGIFLDKLTEDTNGFLILDLHNIYCQSCNFEVEMQEVIKLYPLDKVKEIHLSGGSW<br>QESIYRKKPIRRDTHDDAVPEDILSVLPSVLSQCKNLEYIIIERLGNTIDAEEKKESFLDDFTKVKKLIDLSDVGTPNKDLWAKKEIRFSENPLEDMILHEEQTLLTKLLFD<br>NAGPASIKSQEFHYFKTKNWDPEMILAAQNIIKKWNPY
WP_209784810.1	Chryseobacterium sp. PvR013	MLGVSMMAEADFVSAILPLLQNNSIEVMEWSFDTLYHAHEPDWLHDLLNFYAENDRLIGHGVYYSLFDARWTERQEEWLKKLTEEVSLRKYNHITEHFGFMNTENF<br>HQGVPLPVSLHPKTLEIGQDRLYRLQEAVNIPVGIENLAFSFSIDDVKEQGVFLDKLTEGTKGFLILDLHNIYCQSCNFGVPIQEIINLYPLDKVKEIHLSGGSWQDSVY<br>GDKQVRRDTHDDVIPQDILSVLPSVLTQCQNLEYIIIERLGDTVKTEEEKDNFLNDFNTVSKIIETSDWKVKGKNGWSKREMKLSEKPLENLVLYEEQSRLTKLLFDN<br>VEVKAIKDQDFHYFKTENWDAEMILTAQNIIKKWNPY
MBP6746809.1	bacterium	MLTEDFRQATGALFRSGKVEALEWSFDFGFNGAVEEPWGNDLLDKFSVAGSLTGHGVELSPLSATFSERQRQWLAQAREEFQKRKYLHASEHFGFSEAGPIEKG<br>APLSVPMNEQSLRLGKEMMKRFADATQCPVGLENLAFAFSLDDVKRQGDFIDQLISEVDGFLLLDLHNIFCQVANFNMPELELLNLYPLSKVRELHLSGGSWSRSV<br>SGGRLAVRRDTHDDAVPQEVFNLTALALKLCPNVEFVILERLGYTMMEPEQQQEFRDDFDTIREILDHCYV
MBP1164381.1	Chryseobacterium sp. PvR013	MRKPMLGVSMMAEADFVSAILPLLQNNSIEVMEWSFDTLYHAHEPDWLHDLLNFYAENDRLIGHGVYYSLFDARWTERQEEWLKKLTEEVSLRKYNHITEHFGFM<br>NTENFHQGVPLPVSLHPKTLEIGQDRLYRLQEAVNIPVGIENLAFSFSIDDVKEQGVFLDKLTEGTKGFLILDLHNIYCQSCNFGVPIQEIINLYPLDKVKEIHLSGGSW<br>QDSVYGDKQVRRDTHDDVIPQDILSVLPSVLTQCQNLEYIIIERLGDTVKTEEEKDNFLNDFNTVSKIIETSDWKVKGKNGWSKREMKLSEKPLENLVLYEEQSRLTK<br>LLFDNVEVKAIKDQDFHYFKTENWDAEMILTAQNIIKKWNPY
MBR9919930.1	Bacteroidota bacterium	MPKVLASIACNLDSDMLLASIPLFASEKVQAIEWSFDTLYKHRNIPDWFVELLRTYSDSGRLIGHGVYFSLFSGKWSADQQAWLDRLSRLSANFNFDHITEHFGFMT<br>GADFHKGAPLSLPFTSNVLNIGRDRLQRIAQVCQCPVGLENLAFAYTLEEVKRHGEFLNKLLEPVNGFIILDLHNLYCQLYNFEQDYEDIIQLYPLDRVREIHISGGSFE<br>QSGIQDGKKIRRDTHDSAVPEAVFELLEKTLDRCPNLKYVVLEQLGIGLQTEDSKKQFQEDYFRMKKMLDTFNRRTTEFPNQDFNPPQKEPKTVPLEDLKLHKQQL<br>ELSAILESSESLADVNTKLRQSSLAKSPWQIENWAPEMLETAWCIARKWKAGFQ
MBS1663462.1	Bacteroidota bacterium	MPRLLPAVACNLDADILAACLPLLEESRVESIEWSFDALFNIKQVPDWFLELLSAFSKEGRLIGHGVFFSLFSARWHSQQEAWLRQLQETSSLFRFDHITEHFGFMT<br>GKDFHRGAPLNIPYTASTLAIGRDRLQRIQEACHCPVGLENLAFSYSLEEVKKHGEFLDRLLEPVNGFIILDLHNLYCQLQNFDLDFEELISLYPLDKVREIHISGGSWE<br>DSDIAPGKRVRRDTHDDSVPGDVFQLLENTIDRCPHLKYVVLEQMGSALRTDDSRAGFRSDFSRMEAVVHRFNDDRIPELVDMSFLPPLHDISHPPVEDEQLYRQ<br>QLELSEILETAASYTEAAQRLSRSSLANSEWRVERWQPHMLETAVNIARKWARGWARQGS
MBS2001764.1	Cyanobacteria bacterium SZAS LIN-5	MPSDDFREASRSLFENNKVEVLEWSFDFSWLGNEIAPWCADIIDRFSKSNKLIGHGVTLSPLSARFSKRQEDWLARVRDEFKTRNYIHASEHFGFSEAGPISHGAPI<br>AVPMTAESLHAGKEMLKRYAEATQCPVGLENLAFAFSMDDVKRQGEFIDRLISAVDGFLVLDVHNLYCQVANFDVPITELLNSYPLTKVREMHLSGGSWTESESRK<br>RFAVRRDTHDDNVPQEVFNFAALGLTMCPNLEFVILERLGWTMLDDESQVGFREDFETVAEIVDATYSKEIQGADSFAQTGKSPSVEAQTESFTVDTSKESPSPSA<br>STKSPSLTASTQAPALVSKSEPSLADYQDQLVELLSSNRTNSEILEILKSASQFRHYQDYISSFDPDMVAVACELMGKWARRADS
MBP9092271.1	bacterium	MNYRVPKLGLSLMPTEDFRQATDELFQSGKVEALEWSFDFSFNGVIAEQSCNVLLDNFSSAGALTGHGVELSPLSAKFSKRQSEWLAHAREEFQARKYTHASEHF<br>GFAEAGVIERGAPLAVPMNEQSLRVGKEMMKRFANATQCPVGLENLAFAFCLDDVKRQGDFIDQLISEVDGFLLLDLHNIFCQVANFNMSELELLNLYPLSKVRELH<br>LSGGSWSRSISGARLAVRRDTHDDAVPQEVFNLTALALKLCPNVEFVILERLGYTMMEPEQQQEFRDDFDTIRELLDYCYD
WP_215237124.1	Dyadobacter helix	MSGILPAIACNLDINILRAVLPLLEEEKVEAIEWSFDSLYKYDEIPAWFESLMQEFGDQGRLVGHGVFFSLFAGKWLPQQQHWLDRLRILSKTYRFDHITEHFGFMTG<br>EDFHKGAPISIPFTAQTLALGRDRLARIQDAASCPVGLENLAFAYSVEEVKKHGQFLDQLVQPVNGFIILDLHNLYCQTHNFNITHHELMALYPLEKVREIHISGGSWD

		ESPFDHKEKIRRDTHDHAVPAEVFELLKIAVSRCPHLKFVVLEQLGTGLDTEESQIKFQEDFMTMRKIIEESNDGSRIPYSFIPLSVNSPDAVPAESFMLFDQQRVLSEILETSSDYEEVRMRLNTSILAHSDWGIESWKPEMLYTAMSIAQKWKNGFA
WP_215234353.1	Dyadobacter linearis	MSRIFSAIACNLDTHILQASVPLFEMEKVEAIEWSFDSLFKVETIPEWFTDLVKEFSDHNRLIGHGVYFSLFSGKWSPEQQNWLSQLRKLSGEFHFDHISEHFGFMTGSDFHKGAPLGIPFNKSTLAIGRDRLARIQEACNCPVGLENLAFSYSYEEVKKHGDFLNQLVESINGFIILDLHNLYCQCHNFDIDFQEMLSCYPLDRVREIHISGGSWDNGSEERTVRRDTHDDSVPETVFEYLEKALPRCQNLKFVVMEQLGTALDTVEKQIAFQHDFVKMDGIVKSFDGDPGSNQTNSFLPFGKHPLDMQPVEDIALASQQMQLSAILETAVNLDHAREMLNASDLKHSDWHIEQWQPHMLKTAIDIAQKWKGGF
MBP9092304.1	bacterium	MLQEDFLAATLPLFSNDEVEILEWSPDIGWAAPTMPEWANELLDFYSSANSLYGHGVNYSPMSAEWTAVDSEWIENLKRELEARKFVHFSEHFGFSRIGHLQQGAPLPVPFTTEAIRAGQFKLEKLAHTTGGRIGLENLALAFSKRDAMQQGRFLEDLLRPFDGFVLLDLHNLYCQMENFSLSAEELLGTYPLERVREIHISGGSWSESQAAQTKRKSKPTLAPASVTRIRRDTHDNEVPDEVLALLPLVLANCPNVEAVILERIGGTMPTAEQQARFRDDYRAIAKLIELHTLRQTVGDDAEAMAKTHITQVELSQGRGTLSRECSQSSDQTEILRYQKKLMELLQSEQSPERIIAELSSAFEGELAEYANLSQPQMLAVGQELVKKWTQ
MBT9556781.1	Myxococcales bacterium	MALSWMREPAFLRAATPLLADGLIDALEWSFDTAWRDEPAPVRDALLGHYASAGRLLGHGVSYSLLSADPADDVRKARWLERWRGEQARHPYRHLSEHIGFMTAPGYQRGPPLPMPLTPEVVTLGRDRLNRLVDASNGPVGLENLAFAFSRREVLEQGELLERLLAPTGGFVVLDLHNLWCQAVNFGLDPAALLDSYPLARVHELHVSGGSWAETSHGPVRRDTHDGTVPFEVLELLQLALRRCPEVRFVVVERLGDSFESPDAAAELRADVMAVRGTLSTFDGVTSSLAKPLPLALWPDVHRDDALAVAQSALYATLDSGPTEDDAWSTSEAVSAIRSMIEHAEPRMLATGEALMRRWGRRNTG
WP_213279122.1	Chryseobacterium indologenes	MKKPLLGISMMAEVDFVSAVLPLLQNNTIDVLEWSFDTLYHNNEPDWLRDLLNFYAENNRLIGHGVYYSLFDARWTERQEEWLQKLKEEVSLRRYNHITEHFGFMNTENFHQGVPLPVSLHPKTLQIGKDRLYRLQEAVDIPVGIENLAFSFSVDDVKEQGVFLDKLTEDTNGFLILDLHNIYCQSCNFGVEMQEIIDLYPLDKVKEIHLSGGSWQESVYGKKQVRRDTHDDVIPEEIFSVLPPVLAKCQNLEYSIIERLGHTLKTEGNKENFLNDFNKVKAIIEASDGKEKEKRSWNKRQIQLPEKPLEDLALFEEQSRLTRLLFDGAGAGIIKDQDFHYFKTENWDHEMILTAQNIIKKWNPY
WP_212662956.1	Acaryochloris marina	MTPQLGLSLMPQPNFWQAAQPLFDADEIDVVEWSFDMGWGGVVLPDLLCSVLRQFSQGNGLLGHGVSYSLLSAQLDRRHWLACLQAECLDYSYRHISEHFGWMATRNFARSAPLPMPLLPETLKLGCDRIQKFADIAQVPIGLENLAFAFGLQDVRQQGEFLDQLLDPVDGFLLLDLHNLYCQMHNFQLSATEIFSLYPLERVRELHLSGGSWSHHGTATIRRDTHDQAVPEPVFELLKLALQQCPQVETVILERMGNTLNSKQEQLQFRRDFAHIRQIINHVSHAAEAGGE
WP_212651256.1	Chryseobacterium arthrosphaerae	MKKPLLGLSMMPEADFVSAILPLLQSNSVEVLEWSFDTFYEAKEPEWLDELLNFYSENQRLIGHGVYYSLFDALWTERQENWLKKLKEEVRKRKYNHITEHFGFMNTENFHQGVPLPVPLHPKTLLIGKDRLYRLQDAVQLPVGIENLAFSFSIEDVKKQGVFLDNLIADINGFLILDLHNIYCQACNFEVEMEDIIRLYPLDKVREIHLSGGSWQESVYARKMIRRDTHDDLIPEEIFSVLPSVITQCNNLEYVIIERLGHTIKTDQEKRAFLDDFTRVRSIIDDSEIKTGKKIRWGQKETGFSNSPVEDDALYEEQSRLTKLLFNSSGAEMIKEQEFHYFKTENWDPDMILTAQNIIKKWNPY
MBU6341249.1	Bacteroidota bacterium	MVFPAIACNLDPDLIQTALPLFETEKVAALEWSFDAVFSSEPLPNWFHELIQVYSKGERLIGHGVFFSLFSGKWLPEQSEWLQNLKRYCQIYQFDHITEHFGFMTGADFHKGAPLSIPYNQSTLAIGRDRLLRISQACNCPVGLENLAFSYSLDEVKRHGAFLNELLEPVNGFIILDLHNVFCQLHNFDLDAKDLRALYPLERVREIHISGGSWEFTRIQPEKPVRRDTHDDSVPKAVFDLLKDVLPCCPNLKYVVMEQLGTALHTQAQRDAYRADFYTMERILTQAQPKIPAKNNFVPPVLPPLGNPIEDLALFEQQRILSNILENALDYQGALNQSLLANSDWDIEHWNPAMLETAVAIARKWKNGW
MBT7958730.1	Akkermansiaceae bacterium	MPHVGLSLMPETQFLQASMPLIEAGDVEALEWSFDTGWKNGVRDWLSNLINVYSEAGRLTGHGVHFSLLSAEWTDRQQGWLDQFREEVTRHQYLHHTEHFGFMTAGDFHRSAPLPVPLTNSTLKLGRERFSQLAEVAPCPLGIENLALALSIDDALQQHEFLEKIVTPANGFLLLDLHNIYCQLCNFNLSPAELLDNYPFHLVREIHVSGGSWAPTASSNSPIRRDTHDDDVPAEVFSLLEHVLPRCPDVEVVFLERLGNTLQEHQFEQFRKDFLTLKSIVANG
WP_216444824.1	Chryseobacterium sp. PCH239	MRKPILGVSMMAEPDFVSAILPLLHNNSIEVMEWSFDTLYHTNEPDWLRDLLNFYAENNRLIGHGVYYSLFDARWTARQEEWLKKLKEEVSLRKYNHITEHFGFMNTENFHQGVPLPVSLHSKILQIGKDRLYRLQDAVNIPVGIENLAFSFSIEDVKEQGVFLDKLTEDTNGFLILDLHNIYCQSCNFELEIQQIIDLYPLDKVKEIHLSGGSWQDSVYRKKQVRRDTHDDVIPKDILSVLPSVMKKCPNLEYIIIERLGHTLKTEEERNYFLADFNKVRTIIETSDLKLKQQNGWCKEEIKLPAKPLEDLLLYEEQSRLTRLLYENANVENIKHLDFHYFKTENWDAEMILTAQNIIKKWNPY
WP_218701771.1	Olleya sp. HaHaR_3_96	MKKNKPKLGLSIMPNPEFIAAALPLFESAQVEVIEWSFDTILDKKYQPEWLPLVLKEYGDNNRLLGHGVYYAPLDANWGPNQDNWLQKAKLETLAYNYNHLSEHFGVMSSANAHHGFPLPFDLSDTILKIGIDRLKRLQNTVQLDIGIENLALASNVSDILKQGAFIEKLVAPINGFVILDLHNIYCQSINFDLDLLTIITSYPLHLVKEIHLSGGSWDHAPNLTKPIRRDTHDGRIPEVLLDLLPEVLQRCPVLEFVIFERLGDTFQNENDGLEFRADFNKIQAIIDHTAFSSNARQWTLKKHDLGPPLIDLPLLNKQQELREHIRLETANNHPEWNTNMWITATKLYKKWNT
WP_218736096.1	Cellulophaga sp. HaHa_2_1	MGNKKPKLGIAIAPSLKFLEAALPLFAGEKIEIIEWSFDTLKDAADEPAWLSLLLKEYGEKNRLLGHGVFYSLLDANWSSRQENWLKKVRQETLSHKYCQISEHFGLMSSANAHSGFPLPIQLSNPVLQIGIDRLKRLQATAQVDVGIENLALTANVADILEQGEFLFKLVNPVNGFVILDLHNIYCQSENFNIDMMTIIKSYPLSLVKELHISGGSWDTDSTLSRKIRRDTHDGRIPEVILEILPEVLKICPFLTFIIFEKIEDSFLTEKDGIDFRADFQKIREIIDATAFSVTPKEKKQVAAILGPPVIDVELLQAQVTLRESIALGTYHTNSEWDKDMWKVARKLYEKWNNY
WP_220614797.1	Hymenobacter sp. HSC-4F20	MSSGLPTTPAEPAILATLACNLDVDILSAALPLLAEGRVEALEWSFDTLFWAEQLPDWFSELLGTYSAENRLLGHGVYFSLLSGRWTPEQQQWLEQVRQVVARYPLVHLTEHFGFFTGQNFHAGAPLPVPYTPATLRLGRDRLSRLYEACRCPVGLENLAFAYSLEEVKRHGEFLGRLLEPVSGFLILDLHNLYCQLHNFSVPYEELIGLYPLEQVREIHISGGSWEDSALTPGRKIRRDTHDEAVPEDVFQLLELTIPRCPHLKYVVLEQLGPSLRTEVSRAQFRHDFGRMEAIVQRLRTGRAGYADQLFLPLHPPPTGPAFEDGQLYAHQQHLSHILETAPTLAEAQRLLHASALASSAWKLEQWAPHMLETAVSIAQKWKR
MBW4543370.1	Symplocastrum torsivum CPER-KK1	MFKFKRRTPMVGLSLMMESDFFQATQPLFETGAVEVLEWSFDMGWGVKAKPPWVEELLQFYSQRDRLTGHGVSYSLLSAQPGERQIHWLKSLQQECLDYRYRHISEHFGWMAAGDFYQSAPLPLPLTPETLQLGREHLKRLSDKANAPVGLENLAFAFGLQDVLQQGEFLARLLEPVDGFLVLDLHNLYCQVHNFQQSAEQLLERYPLQRVRELHVSGGSWSVTSKGQQTRTIRRDTHNGTVPEAVFELLALALKRCPSVEVVIFERIGHTLHTEIDIEGFRQDFWRIKQVVKESV
MBX2922030.1	Chitinophagaceae bacterium	MLQESRVEAIEWSFDALYQVQEIPGWFEELLHAYSNENRLLGHGVFFSLFSGRWSDHQAEWLNHLKQTCTKFHFDHITEHFGFMTGADFHHGAPLNIPYTPVTLSIGKDRLSRIYDACRCPVGLENLAFSYSLEEVKQHGEFLDKLIQDVNGFIILDLHNLFCQLHNFSVGYEEMIALYPLHKVREIHISGGSWDITDTGTQKTIRRDTHDDAVPSEVFGLLEKTIPKCTHLKYVVLEQLGNGLRTEESRQNFYNDFLRMEDIVQKNNKKIRDHENFSFLPVHPPVPSIIVEDEKLHFQQMELSHILETALSYEDAVKKLNASSLACSDWKIETWNSAMIETARKIARKWMKK
WP_218779874.1	Hymenobacter crusticola	MLASIACNLDADILSAAFPLLEEGQVEALEWSFDTLFWAEQVPSWFTELLTTYSNHNRLVGHGMFFSLLSGKWTLEQQQWLRQLHQLAAQFRFDHITEHFGFFTGQNFHYGAPLHVPYCEATLRIGQDRLRRIYEACQCPVGLENLAFSYSLEEVKQHGAFLEKLIKPVNGFLILDLHNVYCQLHNFSVAFEELVMLYPTDRVREIHISGGSWEGSVSAPGGRVRRDTHDEAVPEEVFQLLDATLPRCPNVKYVVLEQLGNGLKTEPSKIQFRQDFLRMQALVAKHRDHTSRPLTNTFLPLHPLPPSSIIEDEELYKQQLQLSHILETASSSDEAQRLLHASTLAKTAWKIEQWEPHMLQTAVDIAQKWKK
MBW4573846.1	Aphanothece sp. CMT-3BRIN-NPC111	MFKSKRRTPMVGLSLMMESDFFRAAQPLFESGEVEVLEWSFDMSWGIAAKPPWVEELLEFYSQRDRLIGHGVSYSLLSAQQDERQTHWLKLLQQECLDYRYRHISEHFGWMAAGDFYQSAPLPMPLTPETLQLGRNNLKRFSDTANVPVGLENLAFAFGWQDVLQQGEFIDRLLEPVDGFLVLDLHNLYCQLHNFQQSAEQLLKRYPLHRVRELHVSGGSWSVTSKGQQTKKIRRDTHDDQVPEAVFELLAIALHSCPNVEAVIFERIGHTLHTEAEIVGFRQDFLRMKQVVKDSASCLMS
MBX2874589.1	Saprospiraceae bacterium	MPEILSSIACNLDRNILTAALPLFQGAEVQAMEWAFDSLYQQPEIPLWFESLLHTFADAGRLVGHGVYFSIFAGKWSAKQEQWLFELEELARKYPFDHVTEHFGFMTGADFHTGAPLSVPYTPTTLAIGQDRLRRIQQAAQCPVGLENLAFAYTPEEVWRQGDFLDRLLTPVNGFLILDLHNLYCQLYNFQLTFENLIQAYPLQRVREIHISGGSWEATKNAPDQPIRRDTHDDRVPEAVFDLLSKALPILPNLKYVVLEQLGLGLDTAAQQEQFRSDFNRMRQIVASFNQTNTELVQHHFKAPKSLPASRPAQSLDLQKDQEVLSYILEESQSLAEAQSALLESRLAKSAWQVEHWSPKMLETAVQIAQKWKKGFR
MBW3130172.1	Hymenobacter profundi	MPSILSAIACNLDDHILAAALPLLEAGQVEAVEWSFDTLFDGRQLPNWFAELLTAYGEQGRLIGHGVYYSLLSGRWTPEQAQWLEQLRLLATRFHFDHITEHFGSFTGQNFHTGAPLPVPYTAAILRLGQDRLLRIADACQCPVGLENLAFAYSLEEVQRHGEFLEKLLTPVNGFLILDLHNLYCQLHNFDLSTDSLLALYPLHRVREIHISGGSWEDSRVEQGRQIRRDTHDEAVPDEVFALLERIIPQCPQLKYVVLEQLGTGLRTEASQHQFRQDFQRMAAIVEASRHRAPSHQPNAFQPPAALPTGPAAHDEALHAQQRQLSQILETATSYEEAQQRLRASSLAHTDWQLEQWAPHMLETAISIAQKWK
MBX3213937.1	Labilithrix sp.	MMSPWVGLSLMTEDDFRVAAAPLFAGGEVDMLEWSFDAGRRPAPAWAEALLDHYAGEGRLFGHGVHYSPFSARWEARQQSWLDALGRELSRRPYARVSEHYGFMTAGPYVRGAPLPVPRDRASLQIGRERLDRLRAVLASAGSCPLGLENLALAWSRDEALAHGGFLDDVLTGHDDFIVLDVHNLYCQAENFGLSPDELLATFPAARVGELHVSGGSVLRAWPGRRDEVRCDTHDDAVPARVFDLLERALARFPNVRAVVFERLGGTIRTVEDAEALRADFRQTKALAALARRPADARAGGPDAGASAGGGGAAPRVLAEEGDVSALAAFQSALLALLERENDESVIRAHLEIAPEIAAYRGVVRAFDGRALGIAARVVKRWARRRPAGEAAE
MBX3076151.1	Candidatus Obscuribacterales bacterium	MPSEDFQQATLELFEQDRVGVVEWSFDFALNGAEIDVWSGQILERFSDKNALLGHGVNLSPLSARFSERQEKWLAHARSEFELRKYVHASEHFGFSEAGPIAHGAPLAVPMTPESLRRGQEMLKRYADAVQCPVGLENLAFAFCMEDVKRQGEFIDQLISSVDGFLLLDAHNIYCQVENFGVSERELLVSYPLSKVREIHLSGGSWSFSETGARLAVRRDTHDDVVPQEVFNIATLALKLCPNIQYVILERLAYTMMEPEQQQGFREDFETIEEILELCYV
MBX2929408.1	Saprospiraceae bacterium	MRKIHPGIACNLDAHILSAAVPLLEQAQVGAIEWSFDALFRLRDVPEWFQGLLQAFAEAGLLTGHGIYFSIFSAQWSKEQEAWLSHLRALSAEIKFDHISEHFGFMTGADFHNGAPMSVPLTNATLAIGADRLMRIQDACNCAVGIENLAFAFCEEEVKQHGDFLSRLLIPVNGFLILDLHNLYCQMKNFDVDYEALIGHYPLDRVKEIHISGGSWAPASGAPGGQVRRDTHDDAVPAEVFELLAHTIPRCPNLKFVLLEQIGSGLQTDLQKVRFRQDYLRMCAICESVEIERDAAAGGNNFLPAGEFHLPAPVEDAMLYEQQAALSQILENASGGADAARRLATSILAGTPWQTEHWPEYMLETAVQIAQKWKKGFVV
MBX3219529.1	Labilithrix sp.	MTPWVGLSLMFEDDFRVAAAPLFAAGDVDVLEWSFDTGPRLVGAAGDRGPSWAEALLDHYAAEGRLFGHGVHYSPFSARWEERQQRWLEALGRELARRRYVHVSEHYGFMTAAPFVRGAPLPVPRDRASLDVGRERLDRMRAVLASAGGCRLGLENLALAWSRDEALAHGAFLDDVLTCDDDFIVLDVHNLYCQAENFGLSPDELFATFPAARVGELHVSGGSVLSAWPGGGGEVRCDTHDHAVPAPVFDLLDRALGRFPNVRAVVFERLGGTIRSAEQAEELRRDFRQVKAIATRPRRFGEEHASTDALRDSTSRPAAPAISAPPRLPSSSRGDVEALAVLQSALLDLLDRESDESIIRAHLEVAPAFAEYRDEVRAFDGRALGIAARIVKKWGRKATTHAS
MBX3262053.1	Labilithrix sp.	MSKLLAVMTPWVGLSLMTEDDFRVAASPLFAGGEVDVLEWSFDAGRRPVPAWAEALLDHYAGEGRLFGHGVHYSPFSARWEPRQQSWLEALGRELSRRRYARVSEHYGFMTAAPYVRGAPLPVPRDRASLQVGRERLDRMRAVLSTGGTCPLGLENLALAWSRDEALAHGAFLDDVLTHDDDFIVLDVHNLYCQTENFGLSPDELLATFPAARVGELHVSGGSVLPAWPGGRDEVRCDTHDHAVPACVFDLLERALARFPNVRAVVFERLGGTIRTVEDAEALRSDFRRTRELAARARGVADAGAAPSLPDARRASAPERGGVVALAALPSGAASSARGDDDALAEFQSALLALFERESDEFVIRAHLEVAPEFAPYRAAVRAFDGRALGVAARVVKKWTRRAPATHDP
MBX7082900.1	Nannocystaceae bacterium	MTVPIGLSLMLEDAWREAALPLFERGDVDALEWSFDQAWRRPLPSWAAALLDHYAAADRLWGHGVTMSPGSVDAGDRHEAWLQRLQHELPQRRLRGVSEHFGFMVAGAIDLGAPLPLPDGPGARAVLRRNLARLAAVAGVPVGIENLALALCRDDAWQQGPMLADVLAHVDGYLVLDLHNLWCQLVNFELDPLATLQRYPLQRARCIHVSGGSWWGEARFRRDTHDDEIPEPVLALLQQAVPLCPALEVVVIERLGETLVGHPDALQRDLERVRARLGAA
MBX9669130.1	Candidatus Obscuribacterales bacterium	MKQQVLAGLSLMPDDDFADSTYPLFEDGLVDWVEWTFDMGWSDRGVPGWLRELLDYFGERGRLSGHGVQYSALSARWTDFHDRWVARLDQEVRERKYAHISEHFGLARAGQYLFHAPLPVPYCDAAISVGRDNIAKIAAIARCPIGLENLALAFCPLDVAHQGEFLDKLLAPVVDGFLVLDLHNLYCQSVNFQVPFFELLKSYPLERVTEIHISGGSFSHSSAGNTTQPIRRDTHDGPVPQELFDFLPRVIESCPRVQVVILERLGNTLSNKSERSGFASEFRKLRKTLYRLQTKSTNIGSQSGFSHTTLQTLSRDRDLNHEEKTLLVYQDNLLNKLSEDRDATWVKMQLVDLHNNHSWLLGYIDELEVRMLEVGQELTKKWGVRV
MBX9696430.1	Cyanobacteriota bacterium	MQQNKPRLGLSLMPTDDFHQASAELFERNLVKVVEWSFDLAWSGAIIEPWCQNIIDRFSVSNALIGHGVNLSVLSARFSKRQQEWLARTKDEFQRRTYIHATEHFGFSEAGPIAHGAPLAVPMNSESLKIGKEMMRRYADATQCPVGLENLAFAFGIEDVKRQGDFIDQLISEVDGFLLLDLHNIFCQVENFGFSAQELLDSYPLSKVREIHLSGGSWSESVSGKRAAVRRDSHDDGVLQDVFNFAALAMKLCPNIEFVILERLGNTMMDGPESQREFRDDFEAMEEILHYCYA
MBX9772085.1	Candidatus Obscuribacterales bacterium	MAPSLKTGFSLMPTDDFYSVLLNLLDSGKVDVVEWSFDMSWDTTTPDWCESILAEFGKRESLLGHGVRFSPLSGKRSARQERWLQLLEKDCRHHKYLHVSEHFGFFEAGSYTDGAPLPLPMNDGSLGAGRSSLQALRDVCKTQVGLENLAMAFSVNDVRQQGDFLEALLEPLDAFLILDLHNLFCQSVNFDIPAAELMNSYPLHRVKEIHISGGSWSSSNTGQRKHVRRDTHDGPVPDEVFGLVSVARAKCPQLEFAILERLGNTMDDPADQSQYMDDFHRLWSLMNE
MBX9567968.1	Candidatus Obscuribacterales bacterium	MVTPSKKEIFCGLALMDSPDFLAASLPLFDAGEVEVLEWSFDIGWENGQDEWASELVAFYSSENRLLGHGVNFSVLSGKWTAGQQAWIERFREECAQHSFMHVSEHFGFSRAGNVIQTSPLPVPYSKSALALGVERLKMMSSIAKVPIGLENLGFAFSIDCVKEQADFINQLLDKSDGFLLLDLHNAFCQIANFGVDAEEYFSLYPLQRVREIHISGGSWSADRDGNSIRRDTHDHEVPEEVFDLLMQVLWRCPNLKTVILERLGNTIRSEEEAARFRSDFLHLKKLIQVFCSESYTEVIPPVSTLRTNGPKKAAKG
MBX9947652.1	Candidatus Obscuribacterales bacterium	MPTKPKLGLSLMPTEDFEQATVSLFQNNQVHAIEWSFDFAWNGAVISEWCEGILDEFSDKGALTGHGVNLSPLSARFSKRQEAWLEFAKEEFRSRKYVHASEHFGFSEAGPIAQGAPLAVPMNSESLRIGKEMLKRYADATQCPVGLENLAFAFGLEDVKRQGDFIDQLISSVDGFLLLDVHNIYCQLANFGVSELELLNSYPLSKVREIHLSGGSWSSSISGKRAAVRRDTHDDAVPQEVFNLATLALKLCPDVEFVILERLGYTMMDSKSQEEFREDFETMEEILEYCYA
WP_223401109.1	Dyadobacter fermentans	MAEIYSSIACNLDTHILQAALPLFEQEKVEAIEWSFDTLYKFGEIPAWFTDLVSEFSSHRRLIGHGVYFSLFSGKWSPGQQEWLARLKKLSGEFNFDHITEHFGFMTGEDFHKGAPIGIPLTPRTLALGKDRLQRIQDACQCPVGLENLAFSYSIEEVRKHGTFLDQLVESVNGFIILDLHNIYCQAHNFDIDFADILGLYPLNKVREIHISGGSWDD

		TLTPTRKPVRRDTHDESVPEAVFDFLAEAIPHCPNLKFVVLEQMGTALETQASQAAFQSDFLRMDSIVKSLARKSLPIPEHLFAPLAGTAHPTPLEDPDLYKQQMQLSHILETAANVSDAHAMLRSSDLGNSAWEVENWAPYMLETAMAIAQKWKNGFV
WP_222165112.1	Edaphocola aurantiacus	MSRTILPTIACNLDADILSASLPLLEEGVVGALEWSFDALFRQDHVIPEWFYDLLTAYSNEGRLIGHGVFFSLFSGRLLPEQAAWLQRLKQLTRQYRFDHITEHFGFMTGSDFHKGAPMSVPYTSGTVAIGRDRLQRIQDAAQCPVGLENLAFAYSLDEVKRQGAFLEQLIEPVNGFIILDLHNVYCQLHNFNIPFEVLIQAYPLDRVREIHISGGSWETAVTDQSRSIRRDTHDGRVPESVFELLERTLPLCPQVKYVVMEQLGAGLQTEAAKIGFRNDFQQMQKITEQFLEHHPRMAAVPNNFLPEQSLPPGQIIEDEYLYGEQQILSYILEHAAGHAEAMEQLQHTALRNSVWDIEKWQPDMLETAIRIARKWK
WP_223559707.1	Chryseobacteriu m lathyri	MKKPLLGLSMMPEADFVSAILPILQNNSVDVLEWSFDTFYDAQEPEWLSGLLDFYSANNRLLGHGVYYSLFDAKWTERQEGWLEKLKKEFRHRKYNHITEHFGFMNTENFHQGVPLPVPLHPKTLQIGKDRLKRLQDAVEIPVGVENLAFSFSIDDVKEQGDFLDKLIEDIDGFLILDLHNIYCQSWNFEISMQEIIRLYPLEKVKEIHLSGGSWQESAYGKKPVRRDTHDESIPEEILNILPEVLSQCHNLEYIIIERLGHTLNTDSEKDAFFDDFNRVKKIIQASDSLPGNKRVWHKKNEEPSVPLEDLLLYDEQTELTKMLFDGTQIHSVKKHDFHYFKPENWDEEMITTAQQIIKKWNPY
WP_223598940.1	Chryseobacteriu m sp. GVT01B	MKKTVLGISMMAEADFVSAVLPLLQNNAVDVLEWSFDTLYYINEPDWLRDLLNFYAENNRLIGHGVYYSLFDARWTERQEEWLQKLKEEVSLRKYNHITEHFGFMNTENFHQGVPLPVSLHPKTLQIGKDRLYRLQQAVDIPVGIENLAFSFSIDDVKEQGVFLDKLTEDTNGFLILDLHNIYCQSCNFEVEIEEIIDLYPLDKVKEIHLSGGSWQESVYGKKQVRRDTHDDVIPEEIFFILPSVLAKCQNLEYIIIERLGHTLKTEEDKENFLNDFNKVKTIIEASDWKEKEKRSWNKRQIQLTEKPLEDLALFEEQSRLTRLLFDGAGAGIIKDQDFHYFKTKNWDPEMILTAQNIIKKWNPY
MCA0235173.1	Bacteroidota bacterium	MLYPSIACNLDTDILLAALPLLESERVQALEWSFDTLYQVRDIPAWFDELLLEYGRAGRLIGHGVFFSLFSGRWSPEQAQWLEHLKKQCSVFQFDHITEHFGFMTGADFHSGAPISLPFTASTLAIGHDRLRRIQEVCACPVGLENLAFAYSLEEVKRHGDFLNRLVEPVNGFIILDLHNLYCQAHNFDMPAEELLDLYPLERVREIHISGGSWENSPLLPERPVRRDTHDDAVPAEVFQLLRNTIPLCPNLKFVVLEQMGTALHTEANRRQFRQDFDAMHTILEAFDAPAQSTGNTFAPFSLPALGTPLEDADLYRQQRQLSEILETADSYEAARQMLENSDLAGTAWGTEHWAPYMLETAMRIAQKWQKGFQQPA
WP_223608183.1	Chryseobacteriu m sp. OSA05B	MKKPLLGLSMMPEADFVSAILPVLQSHSVEVLEWSFDAFYDVAEPEWLSGLLDFYAGNNRLLGHGVYYSLFDAKWTDRQEIWLKKLKEEVSRRKYRHITEHFGFMNTENFHQGVPLPVPLLPKTLEIGKDRLSRLQDAIEIPVGVENLAFSFSIDDVKEQGEFLDKLVEDIDGFLILDLHNIYCQSCNFEVDMMEMIRLYPLGKVKEIHLSGGSWQESAYGKKPVRRDTHDDRIPEEILKILPEVLIQCHPEYVIIERLGHTLNTEVDKEFFFDDFNRVKEIIEFSDEPVGDEKIWNRKNRTYSKPVEDLLLHDEQTELTRLLFEGNGTEAIKNSDFHYFKPEDWDEEMIVTAQQIIKKWNPY
MCA9105848.1	Planctomycetales bacterium	MSRSSAPWVGLSLMLGDAFHDAVRPLFDGDEVDVVEWTFDMGWGRPLPAWLEATLDEFSSRGRLLGHGVSYSVLDASLTDRQPRWLHHLQVELQRRRYHHLSEHFGLMGGGNFHLSSPLPIPLSDTAVEVGNSRLRDLARVARLPVGLENLALAFSLSDVQQQGEFLERLLAPVDGFLLLDLHNLYCQAMNFNVSLEELVDSYPLDRVRELHLSGGSWSQPLSHARPVRRDTHDDRVPEELFQILPTVLRRCPRVEAVILERMDGTIDSANLSDAANFRADYRRLRAITREAG
MCA9805219.1	Cyanobacteria bacterium HKST-UBA02	MPTEDFRQASAELFEQQKVQAVEWSFDFGFDGAQIEPWCLDILDRFSEGGSLIGHGVTLSMLSASFSERQEAWLASAREEFVNRSYVHASEHFGFSEAGPFADGAPLAVPMTSQSLRQGKEMIKRYADAVQCPVGLENLAFAFGMEDVKCQGDFIDQLISEVDGFLLLDLHNIYCQIENFGVEADTLLDSYPLSKVREIHISGGSWSHSLSGRRPAVRRDTHDDSVPQEVFNLAALALKLCPSVEFVILERLAFTMTESKSQRDFRDDFYTMQEILVVRA
WP_225718062.1	Chryseobacteriu m rhizoplanae	MGKPLLGLSMMAEEEFLSAVLPLLQGNSVEVLEWSFDTFYNSQEPNWLSDLLNFYAENDRLVGHGVYYSLFDARWTERQEIWLKKLKEEFRKRKYNHITEHFGFMNTENFHQGVPLPVSLHPEIVEIGKDRLYRLQDAVQVPVGIENLAFSFSLNDVMEQGIFLDRLTEDIEGFLILDLHNIYCQSCNFEVEMSEIIKLYPLDKVKEIHLSGGSWQESIYGKKLIRRDTHDDEIPEAILSVLPSVLLQCKNLEYVIIERLGNTINTREKKKRFLDDFLKVKALIDSSGLEIQNKNSWTKKELHFPESPLENIILHEEQTLLTKLLFDNVEAECIKNHKFHYFKTENWSPEMILTAQNIIKKWNPY
MCB0707287.1	Saprospiraceae bacterium	MSKIHSSIACNLDANMLLASLPLFASEKVQAIEWSFDTLYKHRHIPDWFVELLQAYSAENRLIGHGVYFSLFSGKWSPDQQAWLDHLSRLSTNFNFDHITEHFGFMTGEDFHRGAPLSLPFTTKTLLLGQDRLKRMQEACGCPVGLENLAFAYSLEEVEQHGAFLEALVEPVNGFIILDLHNLYCQLHNFDVSFDDIIKRYPLDRVREIHISGGSWEPSRQKPEKSIRRDTHDDAVPEAVFELLEQSLMLCPNLKYVVLEQLGIGLASEDSKQQFQADFYRMERLVEAYNLSLAPQSAISFLPVQAFESSSRPAVEDMQLYQEQQVLSRLLEDSTDLESFQAGLNNSILAHSDWRIETWAPEMLETAYQIAQKWKKGFA
MCB0644088.1	Phaeodactylibact er sp.	HASIACNLDTHLLLTALPLFEAEQVQAIEWSFDTLFKIPEIPDWFLELLDVFGEADRLVGHGVFFSLFSGQWSPEQADWLAKLEALSQRFRFNHITEHFGFMTGADFHKGAPIPIPYTPTTLNIGRDRLQRIQQACQCPVGLENLAFAYCLDEVKQHGEFLAALVEPINGFIILDLHNAYCQAHNFELGLEEILAQYPLHLVREIHILGGSWQDSSILPERQIRRDTHDDAVPEAVFELLELALPRCPNLRFVVLEQIGTALYDPRQQAQFRADFLRMKTLVEHHNQERNTGPENDFQNPHPIQLAPTPAQNADLARQQQSLSDILETAENLAAAQSALAASPLAQSSWQVERWHPAMLETVLQIAQKWKEGWK
MCB1136801.1	Chlamydiia bacterium	MRSQLEKTRPLAGLSLMTEADFLRAAFPLLASGEVEVLEWSFDTGWGANAPAPALREVIAAFSAKSRLIGHGVSFSLLSGEWTERHQTWVDALREEVEDLSYRHVSEHFGFLTAGNMHQGSPFPVPLTPSILELGQTRMCLLAETAKVPVGLENLSFAFTASDVARQGEFLDLLLEPVNGFLVLDLHNLYCQAVNFECELESLMLRYPLERVREMHISGGSWSETPSSTRPVRRDTHDEAVPEAIFEFLPTALEHCPGCEAVIFERIGNTLQHPTDVLQFQQDFRRLKHILESLYANQPSYARTVPSHTVGSSC
MCA9816168.1	Cyanobacteria bacterium HKST-UBA01	MTVKPKLGLSLMSTPDFHQASAELFESGKVEVVEWSFDNAFNGAFIEPWCQDIVDRYSDANALLGHGVTLSPLSVRFSQRQRDWLAGAAEEFRSHNYIHASEHFGFSEAGPVAHGAPLAVPMNSATLKSGKEMMKRFAEATRRPVGLENLAFAFCIEDVKRQGEFIDHLLSDVDGFLLLDLHNLYCQQLNFNIPAIELLESYPLERVREIHISGGSFSPSISGRRIFVRRDTHDEPVPQEVLELAVHALKRCAGAEFVIYERLGFTMEDEESQAEYREDFETLAQLLEMIYV
MCB9279281.1	Lewinellaceae bacterium	MIHSAISCNLDADILEAALPLLEASEVEALEWSFDTLYKSGGMPPWFEALLDAFGREGRLVGHGVYFSLFSGRWSEDQAKWLAELGRLSDQFQFNHITEHFGFMTGENFHAGAPVSIPFTKATLAIGQDRLKRIQQVCRRPVGLENLAFAYSIEAVKVHGEFLEQLVAPVNGFIILDLHNLYCQLHNFGVDFAEMIRLYPLDRVREIHISGGSWEDATAQPGRRIRRDTHDDSVPDEVFRLLENTIPMCPNLKFAVLEQLGAGLKTEASKVAFRGDFRKMAAIIRRHNPTDNLSGINEFASKIEMPEGPPLEDEVLYGQQRELSYILENAADATTAENLLCKSSLAKTDWRIEDWDPAMLETAVRIARKWRRGFV
MCB9468714.1	Candidatus Obscuribacterale s bacterium	MTVKPKLGLSLMSTPDFHQASAELFESGKVEVVEWSFDNAFNGAFIEPWCQDIVDRYSDANALLGHGVTLSPLSVRFSQRQRDWLMGAAEEFRSHNYIHASEHFGFSEAGPVAHGAPLAVPMNSATLKSGKEMMKRFAEATRRPVGLENLAFAFCIEDVKRQGEFIDHLLSDVDGFLLLDLHNLYCQQLNFNIPAIELLESYPLERVREIHISGGSFSPSISGRRIFVRRDTHDEPVPQEVLELAVHALKRCPGAEYIIYERLGFTMEDEESQAEYRGDFETLAQLLEMIYV
WP_227769778.1	Hymenobacter sp. 15J16-1T3B	MAESPSILATVACNLDADILSAAFPLLEEGRVEALEWAFDALYWTEQPPAWFADLLDSYSQAGRLIGHGVYFSLLSGRWTAEQQQWLRQLAQVAARFRFDHVTEHFGFFTGQNFHHGAPLPVPYSPAALRLGQDRLQRLQAACQCPVGLENLAFAYSLDEVQRHGEFLDQLLAPVNGFVILDLHNLYCQLHNFAVPYEALIELYPLERVREIHISGGSWEASSLVPGRTVRRDTHDEAVPDEVFALLARTLPRCPNLKYVVLEQLGTGLKTAASREQFRRDFRRMAALLEPQRTGRPAAPDSPFAPLPLAPAGPAAEDETLHEQQRQLSQILETAPSYDAARQQLLQSGLAGTDWRIEHWEPHMLETAVNIARKWKK
WP_227941441.1	Chryseobacteriu m sp. X308	MRKPMLGVSMMAEADFVSAVLPLLQNNSIEVMEWSFDTLYHTNEPDWLRDLLNFYAENNRLIGHGVYYSLFDARWTARQEEWLKKLKEEVSLRKYNHITEHFGFMNTENFHQGVPLPVSLHSKILQIGKDRLYRLQDAVNIPVGIENLAFSFSIEDVKEQGVFLDKLTEDTNGFLILDLHNIYCQSCNFEVGLQEIINLYPLHKIKEIHLSGGSWQESAYGKKQVRRDTHDDVIPEEILSVLPSVLTQCPNLEYIIIERLGHTLKTEEERNDFLADFNKVRTIIETSDLKFKEKNRWCKKEVKLSEKPLEDPLLYEEQSKLTRLLYDNSDVKAIQDLDFHYFKTQHWDAEMILTAQNIIKKWNPY
WP_226176240.1	Hymenobacter lucidus	MSTQVPAAAVSEPGILSAIACNLDADILSASFPLLEQGRVEALEWSFDALFWAEQVPEWFTDLLRAFADQKRLVGHGVYFSLLSGRWTREQQQWLEQLRELAGRFAFDHITEHFGFFTGQNFHSGAPLPIPYTSAALRLGQDRLRRIQQAAGCPVGLENLAFAYSLDEVQRHGEFLDQLVEPVNGFLILDLHNLFCQLHNFAVPYEELIKLYPLERVREIHISGGSWEKSGQEPGRQVRRDTHDDSVPEEVFELLALTLPRCPNLKYVVLEQLGNALKTEASRTQFRADFGRMETLVGEFRRRAAPRASQLFLPPQPWLAGPVAEDAHLHEQQQQLSRILETAPSFEAARQQLHASSLAHSAWKIEEWAPHMLETALRIAQKWKQ
MCB9230954.1	Bacteroidia bacterium	MIYSSLALNLDVPLLQAALPLLEAEKVEAIEWSFDTLFKVRDIPPWFVELLREFGQAGRLVGHGVFFSLFSGRWVPEQDHWLKRLREMSAEFQFNHVTEHFGYMTGVNFHQGAPMSVPLTPEIMALGQDRLQRIADAAQCPVGLENLAFANSLDEVRQQGEFLSRLLEPVNGFIILDLHNLWCQLHNFELDFADLIRLYPLDRVREIHISGGSWEDSGARPGKKIRRDTHDHAVPEEVFALLENTLPLVPNLKFVVLEQMGHSLADAAQQEIFRSDFLRMESLVQSSGQKEKAEIPGLFQPPRVSFPEIPLQNENLFLQQRELAHILETSTNLDQALAQLQASALAHSKWKIETWAPEMLETALHIAQKWKDGW
WP_228421676.1	Chryseobacteriu m culicis	MLGVSMMAEADFVSAILPLLQNNSIEVMEWSFDTLYHAHEPDWLHDLLNFYAENDRLIGHGVYYSLFDARWTERQEEWLKKLTEEVSLRKYNHITEHFGFMNTENFHQGVPLPVSLHPQTLEIGQDRLYRLQEAVNIPVGIENLAFSFSIDDVKEQGVFLDKLTEGTNGFLILDLHNIYCQSSNFEVPIQEIINLYPLDKVKEIHLSGGSWQDSVYGDKQVRRDTHDDVIPQDILSVLPSVLTQCQNLEYIIIERLGDTVKTEEERDNFLNDFNTVSTIIETSDWKVKGKSGWSKKEMKLSEKPLENLVLYEEQSRLTKLLFDNVEVKAIKDQDFYYFKTENWDAEMILTAQNIIKKWNPY
MCC6723041.1	Saprospiraceae bacterium	MLLPSIACNLDKNLLTTALPLFESGSVEGLEWSFDALFKQPNMPDWFQELLLTYANEGRLIGHGVYFSIFSGKWKPEQTDWLAQLRQFSKTYRFDHVTEHFGFMTGEDFHKGAPISIPYTSTTLGIGIDRLMRIQDACNCPVGLENLAFSYSLDEVKKHGEFLQKLIEPVNGFIILDLHNLFCQLHNFEVGFEEMLKLYPLQRVREIHISGGSWEDSMVVPNRKIRRDTHDEPVPASVFELLEKTIPHCPNLKFVVLEQLSSALNTGEERTAFQADFLKMNDIVAQHSGIEQLTTNAFLPLTTLYFNSQPVEDEALAEQQAILSQILETANSYEDGRNMLRNSSLAATAWNVENWDPAMLETAIAIARKWKHGF
MCC6283020.1	Saprospiraceae bacterium	MKTLSSIACNLDADILLAALPLLEAEKVQAIEWSFDTLYKFREVPPWFTELLQEFGRAGRLIGHGVFFSLFSGKWSAAQQDWLNQLTQLSKTFRFDHITEHFGFMTGADFHAGAPISLPFTPVTLAIGTDRLSRIFDACQCPVGLENLAFSYSLDEVKKHGDFLFRLIEPINGFIILDLHNLYCQSHNFGLEPDALLQLYPLDRVREIHISGGSWEDSPLLPSRPIRRDTHDDSVPAAVFQMLEYALGQCPNVKFVVMEQLGAGLKTETSRRQFQADFETMDAILRQQESSTQPTNAFLPAGRIELGQPLEDELLHRQQRQLSDILEQASGCTDAQRLLSASELAHTAWDVEKWEPYMLETALRIAQKWKKGFS
CAH0184169.1	Chryseobacteriu m sp. Bi04	MMAEADFVSAILPLLQNNAVDVIEWSFDTFFEAEEPSWLKDLLDFYAGNDRLLGHGVYYSLFDARWTERQEMWLQKLKQEVQKRNYNHITEHFGFMNTENYHQGVPLPLSLHPKTLEIGKDRLYRLQDAVDIPIGVENLAFSFSVNDVREQGVFLDKLIDDIDGFLILDLHNIYCQSCNFEVSMQDIVGLYPLEKVKEIHLSGGSWQDSVYGKKMIRRDTHDDVIPEEIFAVLPSVLSQCHNLEYIIIERLGHTIHTEEEKQCFFNDFMRVKKILNDSGGMKRKNEKWVKRERLLSEPVENSILYEEQIRLTKLLFDHADPTLIKNQNFHYFKTKNWDLEMILTTQNIIKKWNPY
MCC6672286.1	Planctomycetota bacterium	MDVRVGLAWMPEPEFTAAVLPLLAAGEVGALEWSFDTLPDGHDEDPALTGLLDAFAAQERLYLHGVTFNTFGGRLDARSRRWLERLRSACARRRVRHVSEHFGFLRAGGWRDGPPLPVPTDACALDLGRERLRLLRDAACCPVGIENLAFAFARDEARRQGAFLDDLLAPVDGFLLLDLHNLHCQAANFGMDPEELLASYPLHRVREIHVSGGSWREGSHGPVRRDTHDGTVPSAVLDLLAIALPHCPRLEVVILERLGASLPDRAAQLAFASEYRGLAARIAEARVH
MCC6978279.1	Candidatus Melainabacteria bacterium	MFEEERVESLEWSFDFTWNGVVVDPWALELIDKYSEANALTGHGVNLSPLSARFSKRQEEWLARAREEFKTRKYVHASEHFGFSEAGPIKHGAPLSVPMDAASLKVGKEMLKRYADATGCPVGLENLAFAFSINDVKKQGHFIDELISSVNGFLLLDLHNIFCQIANFGISELELLNSYPLDKVREIHISGGSWSPSISGKRAAVRRDTHDDGVPQEVFNLAALALKLCPNIEFVIFERLGNTMFSLEAQSEFRDDFESMEQILEFCYA
MCC7453008.1	Crocinitomix sp.	MKTSQSTIYSTVACNLDQHLLQATLPLFASEKIEAIEWSFDTLFKVKNIPDWFVELLKTYSQANRLIGHGVYFSIFSGKWSEDQTNWLRHLKKTANHFKFDHVTEHFGFMTGSDFHKGAPISVPYNQSTLALGRDRLVRIYDACQCPVGLENIAFAYTLDEVKQQGEFLTKLIEPINGFIILDLHNLYCQLQNFSLSFDTLIKAYPLDRVREIHISGGSWEPSDVHPTRNIRRDTHDNAVPDEVFELLKLSLNRCPNLKFVVLEQLGTALYTSEQQIQFQHDFDRMQNIIHQYLKKNTPQISNTFLPEIKSSLSLEIPQNEELAQQQAELSFILETAKNHSDAHEKLRQSSLASTDWKIENWSAYMLETALKIAQKWKQGW
WP_229047851.1	Chryseobacteriu m arthrosphaerae	MKKPLLGLSMMPEADFVSAILPLLQSNSVEVLEWSFDIFYEAKEPEWLDELLNFYSENQRLIGHGVYYSLFDALWTERQENWLKKLKEEVRKRKYSHITEHFGFMNTENFHQGVPLPVPLHPKTLLIGKDRLYRLQDAVELPVGIENLAFSFSIEDVKEQGVFLDNLIADINGFLILDLHNIYCQACNFEVEMEDIIRLYPLDKVREIHLSGGSWQESVYARKMIRRDTHDNLIPEEIFSVLPSVITQCNNLEYVIIERLGHTIKTDQEKRAFLDDFTRVRRIIDDSEIKTGKKIRWGQKETGLSNSPVEDDALHEEQSRLTKLLFNSSGAEMIKEQRFHYFKTENWDPDMILTAQNIIKKWNPY
WP_229755264.1	Hymenobacter cavernae	MMEEAPIPFAAIACNLDFDILAAAFPLLEAGQVEALEWSFDTLFRAGQVPDWFSELLSAYSQQGRLIGHGVFFSLLSGKWTPQQQQWLQHLQQLSAQFRFDHITEHFGFFTGENFHYGAPLPIPYTQASLRIGQDRLSRIYEACQCPVGLENLAFAYTPDEVKRHGEFLNKLLEPVNGFVILDLHNLYCQLHNFAVSYEELIELYPLERVREIHISGGSWEESEQAPQGKVRRDTHDEAVPEAVFELLARTLPRCPNLKYVVLEQLGNGLKTESSRTQFRHDFLRMEALVQKHRASLLGHPEHLFRPPQPLQLGEVIEDQSLHEQQRQLSRILETAASVEEAQRRLHASVLVGTDWQMEQWEPHMLETAIHLAQKWKQ
MCC6953627.1	Deltaproteobacte ria bacterium	MVRCAIAAMPDERWLGAVMPLLEQGEVEALEWSFDCDWNRGTVPEWTHQLIRHYSEGARLFGHGISLSLLSTELAHHEQWFEKFERVCRDLSFVHVSEHFGFTRTGNAHHEAPLPCPYDPIIATLGASRLQRMKSICGSPVGVENLALALSPDDVRMHSRFIEEIVTPADGFVLLDLHNLYCQSVNFGLGMMELVQSYPLERVCELHLSGGSWNEVNVYGVSSSIRRDTHDAAVPDSLWEVLPEVIRKTPNLRVVTLEQLPDGLAHAPAAEAYREDYRKLRRIVEGGRDAGA
MCC7537066.1	Deltaproteobacte ria bacterium	MDAALPVGVSLMLEEDFLGATLPLFEEGAVDVLEWSFDVGWGRELPAWAQGLLQHFAVSGRLLGHGVSFSPLSARWEEPQRNWLHRAALEVAKLKYRHVSEHFGFMAAGSFTRGSPMPMPVDAAVIRTGRDALSRLAEVTGLPVGLENLALALSSRDVDGQGDHLDALLEPVGGFVVLDLHNLWCQAVNFGRDPVGLLGRYPLGRVRELHVSGGSWSQPSSAEEPFRRDTHDAAIPDEVHALLPEALASCPHVEAVI

WP_230035574.1	Chryseobacterium sp. Bi04	MEKPLLGLSMMAEADFVSAILPLLQNNAVDVIEWSFDTFFEAEEPSWLKDLLDFYAGNDRLLGHGVYYSLFDARWTERQEMWLQKLKQEVQKRNYNHITEHFGFMNTENYHQGVPLPLSLHPKTLEIGKDRLYRLQDAVDIPIGVENLAFSFSVNDVREQGVFLDKLIDDIDGFLILDLHNIYCQSCNFEVSMQDIVGLYPLEKVKEIHLSGGSWQDSVYGKKMIRRDTHDDVIPEEIFAVLPSVLSQCHNLEYIIIERLGHTIHTEEEKQCFFNDFMRVKKILNDSGGMKRKNEKWVKRERLLSEPVENSILYEEQIRLTKLLFDHADPTLIKNQNFHYFKTKNWDLEMILTTQNIIKKWNPY
GJM31625.1	Saprospiraceae bacterium	MSKLHASIACNLDANILLAALPLFEAEKVQAIEWSFDALFKTREVPPWFTALLSTYSKNNRLVGHGVFFSLFSGKWSPAQQNWLDQLKRLSAVYQFDHITEHFGFMTGANFHQGAPISVPFNPVLLAIGQDRLKRIQEACQAPVGLENLAFAYSIDEVKRHGEFLDQLIAPINGFIILDLHNLYCQIHNFEIDFEDIIQLYPLEQVREIHISGGSWEDSPLIPGKKIRRDTHDDAVPQAVFDLLERTIPRCPNLKYIVLEQIGTGLKTEASKRQFQQDFFKMEQIVQNKQRSTTKDQHTFLPSQPFHLGKPLEDVILHAQQLELSDILETATDYQHAQQCLQASSLANSDWKIETWAPAMVETVLRIAQKWK
WP_232831792.1	Taibaiella helva	MDKIRSTIACNLDAGILSASLPLLEEARVEAIEWSFDTLFKLETVPQWFEALLEAFSKEGHLIGHGVFFSLFSGRWLPEQQAWLDHLRRLCRQYHFDHITEHFGFMTGADFHHGAPLSIPFTAETLAIGRDRLARIYEACGCPVGLENLAFSYSLDEVKRHGAFLEALLEPLNGFIILDLHNLYCQLHNFSLDFDTLIRLYPLHRVREIHISGGSWEPSDLFGGQPVRRDTHDDAVPETVFTLLEKTIPMCPLLKYVVMEQLGAGLETEASRQAFRNDFLRMDGIVSAAASDPQAFRPEAFLPQVPVIPGPVTESMALHTQQLELSAILEASAGYEEAYHRLQHSSLAATGWQVETWQPHMLETAIHIAQKWRKKST
MCE3228656.1	Bacteroidota bacterium	MKPLVGLSLMHEQEFLNAAIPLFLKNEVDVLEWSFDTILVEKYKPEWVHQLLKEYSINNRLIGHGVRYSLFDAKWTKRQESWIRKLKAEVKRYNYNHITEHFGFMSHSDFHKGAPLPVPFNKKSLAIGTDRLKRLQHAAELPVGVENLAFAFSKHEINKQGEFLDSLVESVNGFLILDLHNLYCQSQNFKTAIIELVKSYPLKKVKEIHISGGSWQNSVYTKDIKKVRRDTHDEKVPAEIFKVLPEVMKLCPNVEYVIFERLGDTLHNEKEAEDFRKDFRRLRKIIAGINSSKKTQTQNQKPSAAKGLPTARPITNNQKLNPPLFDQPLYNEQQFILDVLRKSQRPSEAFSLLKQKQLPDWNIASWNTSMLETAIGLIRKWDL
MCD6068446.1	Bacteroidota bacterium	MKPLVGLALMHEQEFLRAALSLFAADEVELLEWSFDTILYEKYKPEWLHQLLREYSSKGRLIGHGVRYSLLDAKWTRRQSGWLNKLRKEVNRYPYQHITEHFGFMTSSDFHKGAPLPVPFNKHSLRIGIDRIKRLKDAIGSLPLGIENLAFSFSAADVKEQGAFLAELIRPVDGFLILDLHNVYCQAENFKIDILEIIRSYPLDKVREIHISGGSWKNSIYSKEIKKVRRDTHDERVPEQLFKILPDVLSLCPNIAYVIFERLGNSLQEEEEQEQFRKDFRRIKSIVHRSSPDKKASTVSERSIPYSKAPLNDLSFYKEQRFLSKTILDAGDPVEALLRLKKEELKYWDVDQWNSSMLETAMSIAKKWDL
WP_232720674.1	Chryseobacterium gleum	MRKPMLGVSMMAEADFVSAILPLLQNNSIDVLEWSFDTLYHPNEPDWLCDLLNFYAENGRLIGHGVYYSLFDARWTERQDEWLKKLKEEVRLRNYNHITEHFGFMNTENFHQGVPLPVSLHPKTLQIGKDRLYRLQEAVDIPVGIENLAFSFSVDDVKEQGVFLDQLTEDTNGFLILDLHNIYCQSCNFGLEMKEIVDLYPLDKVKEIHLSGGSWQESVYGKKQVRRDTHDDAIPQNIVSVLPSVMEQCQNLEYIIIERLGHTVKTEEEKVSFLNDFNTVRKIIEISDLKKAERSWMKKEMIFPEKPLEDWMLYEEQSELTKLLFENADAAVIKNHDFHYFKTENWDSEMILTAQNIIKKWNPY
WP_233332259.1	Chryseobacterium gleum	MRKPMLGVSMMAEADFVSAILPLLQNKSIDVLEWSFDTLYHPNEPDWLRDLLNFYGENDRLIGHGVYYSLFDARWTERQDEWLKKLKEEVRVRNYNHITEHFGFMNTENFHQGVPLPVSLHPKTLQIGKDRLYRLQEAMDIPVGIENLAFSFSVDDVKEQGVFLDQLTEDTNGFLILDLHNIYCQSWNFGVEMKEIVDLYPLDKVKEIHLSGGSWQESAYGKKQVRRDTHDDVIPQDILSVLPSVMEQCQNLEYIIIERLGHTVKTEIEKVSFLNDFNTVRKIIEISDLKLAERSWMKKEMILPEKPLEDWMLYEEQSELTKLLFENADAAVIKNHDFHYFKTENWDSEMILTAQNIIKKWNPY
WP_233877771.1	Dyadobacter sp. CY323	MPRMRNGMIYSSIACNLDAHILMASLPLFEQEKVDAIEWSFDTLFKFNEIPSWFSDLIGEFSKNDRLVGHGVYFSLFSGKWLPEQQQWLDKLKKLSDQFHFDHISEHFGFLTGADFHKGAPIGIPLTTSTLAIGSDRLKRIQDACRCPVGLENLAFSYSLEEVKKHGDFLNQLVESVNGFIILDLHNLYCQSHNFSIPFNEIIRLYPLDRVREIHISGGSWDDLSNTHGKIVRRDTHDESVPEQVFDYLKRTVPKCANLKYVVLEQLGTALDSESRRVAFQNDFLRMDGIVKKESSAHINSITNNFLPAIGVIPDKVPPEDLPLYNQQTELSYILENARDLTDARMRLESSALSNSEWNVEQWEPAMLDTAIAIAQKWKNGF
WP_233635913.1	Hymenobacter setariae	MGTPVAPAQPTVLAALACNLDADILGAALPLLEEGQVEALEWSFDALYWASEVPAWFVELLHAYSDQQRLLGHGVYFSLLSGRWTSEQQQWLNQLRELTQRYSFAHITEHFGAFTGQNFHAGAPLSVPYSPVSLRIGQDRLARIAEAARCPVGLENLAFAYSLEEVKRQGEFLGELLAPVNGFLILDLHNLYCQLHNFSIGYEELLECYALDRVREIHISGGSWENSQLLPTRKIRRDTHDEAVPVEVFNLLARTMPRCPNLKYVVLEQLGTSLKTEASHVQFRRDFVQMAALVQQHNHSPRSVPNAFLPRWPALPALAVQDDLLHEQQRQLSHILETAASLPEAQQRLRASALARSAWQIEHWEPYMLETALSIAQKWK
WP_233789821.1	Dyadobacter sp. CY343	MSRIFSAIVCNLDTHILQASVPLFEMEKVEAIEWSFDSLFKVDTIPEWFTDLVKEFSDHNRLIGHGVYFSLFSGKWSPEQQNWLSQLRKLSGEFHFDHISEHFGFMTGSDFHKGAPLGIPFNKSTLAIGRDRLARIQEACNCPVGLENPAFSYSYEEVKKHGDFLNQLVESINGFIILDLHNLYCQCHNFDIDFEQMLACYPLERVREIHISGGSWEMAIEERLIRRDTHDDAVPETVFAYLKKALPRCPNLKFVVMEQLGTALDSVEKQQAFQQDFFQMDKMVKSFEENSNSIQTKHFLPLTALTLQSQPVEDLLLAAQQMQLSSILETAVNLDHARKMLISSNLNNSDWHIEQWQPHMLKTAIDIAQKWKDGF
MCE9579119.1	Deltaproteobacteria bacterium	MTRTPPRVGLNFLPEGDFRAAALPLFEAGLVDALERDIDYAWGSHPRGERRDEPAWALEILDAYAADDALYGHGVWMSVMSGRWTSRQERWIAQLTDECRARRYRHVSEHFGWLSAGPFWRNTMLPAPYTPATVALGVDRLRRLADAVGCPVGLENLGMGLGPVDAQEQGAFLGEILDAVDGFLLLDVHNVWTQAHNARLDPLALLRTYPLARVRELHVSGGDWYEPAPGARPIRLDSHDGDVPGDVWPLVAEALACCPALEVVFLERRGQTLGREPDRAAYRADFLALRAAVEAAFDVGEERCA
WP_233525516.1	Chitinophaga silvisoli	MPEILSAVACNLDANILAACLPLMEESRVEAIEWSFDALYKVKQVPDWFRELLTAFSDENRLIGHGVFFSLFSGKWLPEQQAWLDHLKHTSTEFSFDHITEHFGFMTGKDFHHGAPLNIPYSAATLSIGRDRLKRIYNACGRPVGLENLAFSYSLDEVKRHGAFLDQLLAPVNGFIILDLHNLYCQLRNFDLAFDDLISLYPLDKVREIHISGGSWDDSAASPDRSIRRDTHDEAVPPEVFQLLEWTLPKCPHLKYVVLEQLGNGLVSQESKAAFYNDFLHMQEIVSQSNNKYSDNSFLPLLPTTTGPAVEDLALYQQQLELSAILETAASYDSAMHLLAQSSLANSDWRIEQWEPYMIETAVKIAGKWKK
WP_233823285.1	Dyadobacter sp. CY312	MTKIYTSVACNLDANILLASLPLFEQEKIEAIEWSFDALFKLEQIPPWFADLVSEFGKNNRLIGHGVYFSLFSGKWLPEQQQWLDSLRAFSREFHFDHISEHFGFMTGADFHKGAPISIPYTSSTLALGTDRLMRIQDAANCPVGLENLAFAYTLDEVRRQGAFLSELTNKVNGFIILDLHNLYCQIHNFNVPFEKLLHSYPLEKVREIHISGGSWEQNHAAPEDKIRRDTHDDEVPETVFELLSAAIPKCPNLKYVVLEQLGTALSDEQSRSRFQQDFLRMEQIVKNSKTASDGRIVNNFLPHTSLQSGHVPYEDLQLYREQNELSQILETATDLAHAQKLLASSVLNGTAWQTETWQPHMLETAIAIAQKWKNGFELK
WP_234615733.1	Dyadobacter fanqingshengii	MSEIYSSIACNLDAHILQASLPLFEQEKVEAIEWSFDTLFKVSEIPAWFTDLVKEFSNHNRLIGHGVYFSLFSGKWSPGQQEWLDKLQKLASQFHFDHITEHFGFMTGEDFHKGAPISIPFTAQTLALGKDRLQRIQDACKCPVGLENLAFSYSLDEVKKHGDFLAQLVESVNGFVILDLHNIYCQVHNFNIDFDDIIKLYPLERVREMHISGGSWDETQVGPGRQVRRDTHDESVPEAVFEFLHNAIPKCPNLKYVVMEQMGTALDTLEKQAIFQSDFLRMDSIIKRLGFDYDPKIENTFTPLNTSASNIPLEDHELYKQQLQLSHILETAGSISEAQAMLRSSSLNNTAWEVDKWAPYMLQTAISIAQKWKNGFA
WP_235109853.1	Acaryochloris sp. 'Moss Beach'	MTPQIGLSLMPQPDFWQAAQPLFDADEVDVVEWSFDMGWGGVVLPDLLCSVLRQFSQRNCLLGHGVSYSLLSAQLDRRHWLACLQAECRDYSYRHISEHFGWMATQDFARSAPLPMPLLPETLKLGCDRIQQFADIAQVPVGLENLAFAFGLQDVRQQGEFLDQLLEPVDGFLLLDLHNLYCQMHNFQLSATEIFSLYPLERVRELHLSGGSWSHHGTATIRRDTHDQAVPEPVFELLKLALQQCPQAEAIILERMGNTLNSEQEQLQFRQDFAHIRQITDHVSHVTEAGGK
WP_233850042.1	Dyadobacter sp. CY326	MPEIYASIACNLDAHILQASLPLFEEEKVEAIEWSFDALFNFNEIPSWFTELVREFSDHNRLIGHGVYFSLFSGKWSPEQQEWLHRLRKVSNEFHFDHITEHFGFMTGADFHKGAPIGIPFTTQTLALGKDRLRRIQDACRCPVGLENLAFSYSIEEVKKHGDFLNQLVESVNGFIILDLHNLYCQVHNFDIAFDEIVKLYPLDKVREIHISGGSWDKNPLRGNKKVRRDTHDASVPEAVFQYLRKAMRFCPNLKFVVMEQMGTALHTVERQQMFRNDFLRMSSIIESSNLDQQHPTNTFAPVSNFNINEPPLEDLFLHSQQVQLSQILENASNVDEARVMLQSSDLANTAWKVENWAPYMLETAISIAHKWKNGFS
WP_235149479.1	Dyadobacter sp. CY345	MPQVYSSIACNLDTNILSATLPLFEQEKVEAIEWSFDTLFKYEEIPEWFSDLISEFGNEGRLVGHGVYFSLFSGKWLPGQDAWLKKLNTFSRKFRFDHITEHFGFMTGSDFHKGAPIGIPYTKTFLDIGIDRLKRIQEACQCPVGLENLAFSYSLEEVKNHGEFLSKLVESVNGFIILDLHNLYCQIHNFDIDFQDILALYPLEKVREIHISGGSWDERKTSRGEKIRRDTHDDTVPKEVFDLLKATIPKCPNLKFVVLEQMGTALYSTESQLAFQQDFLKMNAIVKHKEYAFPEKLFNNFLPIDTVSLAPSPLENLTLFEQQLELSSILETAKNFDHARQLLSRSALHHSEWKVENWEPYMLETALAIAQKWQKGFL
WP_234656798.1	Dyadobacter chenwenxiniae	MSGIYSSIACNLDAHILQAALPLFEQEKVEAIEWSFDTLFKFSEIPAWFIDLVSEFSRHDRLIGHGVYFSLFSGKWSSEQQEWLHKLQKLSREFQFDHITEHFGFMTGEDFHKGAPISIPFTSRTLALGKDRLQRIQDACKCPVGLENLAFSYSLDEVKKHGEFLDQLVESVNGFIILDLHNIYCQVHNFNIDFDYIIKLYPLDRVREMHISGGSWDETLADSGRQVRRDTHDERVPEAVFEFLGKAIPRCENLKYVVMEQMGTALDTLESQAAFQSDFFRMDSIIKEARTCFDPKIKNSFAPTNRSADIIPLEDHDLYKQQLQLSHILETAGSVSEAQALLRASSLNDTAWGVDSWAPYMLQTAISIARKWKNGFA
WP_234608367.1	Dyadobacter chenwenxiniae	MSEIYSSIACNLDAHILQAALPLFEQEKVEAIEWSFDTLFKFSEIPAWFIDLVSEFSRHDRLIGHGVYFSLFSGKWSSEQQEWLHKLQKLSREFQFDHITEHFGFMTGEDFHKGAPISIPFTSRTLALGKDRLQRIQDACKCPVGLENLAFSYSLDEVKKHGEFLDQLVESVNGFIILDLHNIYCQVHNFNIDFDDIIKLYPLDRVREMHISGGSWDETLADSGRQVRRDTHDERVPEAVFEFLGKAIPKCVNLKYVVMEQMGTALDTLESQAAFQSDFFRMDSIIKETGTGFDPKIKNSFAPTNRSADIIPLEDHDLYKQQLQLSHILETAVSVSEAQALLRASSLNDTAWGVESWAPYMLQTAISIARKWKNGFA
WP_235139901.1	Dyadobacter fanqingshengii	MPEIYSSIACNLDAHILQASLPLFEQEKVEAIEWSFDTLFKVSEIPAWFTDLVSEFSDHKRLIGHGVYFSLFSGKWSPGQQEWLDKLQKLASQFHFDHITEHFGFMTGEDFHKGAPISIPFTAQTLALGKDRLQRIQDACKCPVGLENLAFSYSLDEVKKHGDFLAQLVESVNGFIILDLHNIYCQVHNFNIDFDDIIKLYPLERVREMHISGGSWDETQVGPGRQVRRDTHDESVPEAVFEFFHNSIPKCPNLKYVVMEQMGTALDTLEKQAVFQSDFLRMDSIIKGLRFDFDPKIENTFTPLNTSASNIPLEDHELYKQQLELSHILETAGSISEAQAMLRSSSLNNTAWEVDKWAPYMLQTAISIAQKWKNGFA
WP_235938302.1	Chitinophaga solisilvae	MPEIRSAVACNLDANILAACLPLMEASKIEAIEWSFDALYKVKDIPDWFVELLTAFSNENRLIGHGIFFSLFSGRWRQEQEQWLQHLRKTSTHFRFDHITEHFGFMTGKDFHHGAPLNIPYTKSTLNIGVDRLKRIHDACRCPVGLENLAFSYSMDEVKRHGAFLDELLEPVNGFIILDLHNLYCQLHNFDITFGEMIILYPLHKVREIHISGGSWEASPAAPERMVRRDTHDDAVPEEVFQLLQKTIPLCPNLQYVVLEQLGTGLDTDTSRTLFRQDFGRMQRIVHEQQPQTGSCFINSFMPDNLLISAVPAADETLFQQQLELSAILESASSCEAAVHALQHSSLAHSDWLIEQWQPYMIETAVSIAQKWKKI
WP_236858879.1	Chryseobacterium sp. MEBOG06	MTKPLLGLSMMPETDFVSAVLPLLESSSVDVLEWSFDTFYDAKEPAWLNELLNFYSENNRLIGHGVYYSLFDAIWTERQEIWLKKLKEEVHKRNYNHITEHFGFMNTENFHQGVPLPISLHPKTLQIGKDRLYRLQDTVNIPVGIENLAFSFCIDDVKEQGVFLDQLVEDTNGFLILDLHNIYCQACNFDVGMQEIISLYPLHKVKEIHLSGGSWQESAYGKKQIRRDTHDDLIPEEIVSILPSVLAQCPDLEYIIIERLGSTIKTDSQKKDFFNDFKKVEKITEASDHQICKKDNWLKKTVEFPEKPIEDLILYEEQTRLTKLLFDSKGSGIIKEESFHYFKTENWDPEMIRTASNIIKNWNPY
WP_235296475.1	Portibacter marinus	MRNGVGKELYSGISCNLDSNILSACFPLFKGGEVEVIEWSFDTLFSHNNLPDWFDHLLTVFGEEGRLIGHGVYFSLFNGTFTKDQQEWLNGLRVTSDRYEFDHISEHFGFMTGQDFHKGAPLNVPLSDNSLAIARDRISRMSYACGKPVGLENLAFCYDKEGLIRQGSFINQILEEVNGFLILDLHNLYCQCMNFDIEFSELILKYPLDRVREIHISGGSWEIAQSEDRKIRRDTHDDAVPREVFEYLQEVIPKLPNLKFVILEQLGTGLDTIVKQARFRDDFETMKSIVDHKQLEKPLATNDFKSKNLKLSKHPISDETLAKQQQELTFILENSVDFRSVKKRLQNSSLASSPWNIESWEDSMLETAFAIANKWKSGF
WP_237144733.1	Pontibacter pamirensis	MDRKELHPVGKEPEILSTIACNLDADILSAAFPLLEEGRVEAIEWSFDTLYWAAQVPYWFTELLLAYSQQNRLVGHGVFFSLLSGKWTKEQQHWLEQLKQLASQYSFDQITEHFGFFTGQNFHSGAPLHVPYTSATLRIGQDRLSRIYEACQCPVGLENLAFSYSLDEVKQHGEFLDKLVAPVNGFLILDLHNLYCQLHNFVISYEDLITLYPLERVREIHISGGSWEASVLEPEKKIRRDTHDDAVPEEVFQLLALTIPKCPNLRYVVLEQLGNGLKTESSRIRFRQDFLKMEALVQKSRSSLSRDVDNLFLPVQPLQKGPAVEDDMLHNQQLQLSHILETAPSYEEARRLLLTSSLAGSDWKIESWAPHMLETAVSIAQKWKKQS
WP_236850266.1	Chryseobacterium sp. MEBOG07	MRKPLLGVSMMAEADFVSAILPLLQNNSIEVLEWSFDTLYHTHEPDWLCDLLNFYAENNRLIGHGVYYSLLDARWTERQEEWLGKLKEEVLQRKYNHITEHFGFMNTDNFHQGVPLPVSLHPKTLQIGKDRLYRLQEAVDIPVGIENLAFSFSVDDVKEQGMFLDKLTEDSNGFLILDLHNIYCQSCNFGVEMQEIINLYPLDKVKEIHLSGGSWQESVYGKMPVRRDTHDDVIPEDIFSVLPTVLTQCQNLEYVIIERLGHTLKTEEEKSNFLHDFNKVRTIIEASDWKNRRENSWTKKEIQLTEKPAEDLVLYEEQSRLTRLLFDNVGAAVIKDQGFHYFNTENWDPEMILTAQNIIKKWNPY
WP_236387059.1	Chitinophaga filiformis	MLAACLPLMEESRIEAIEWSFDALYKVKEVPAWFRELLTAFSNEKRLIGHGVFFSLFSGKWLPEQESWLKHLEQTTDEFHFDHITEHFGFMTGKDFHHGAPLNIPYTTATLNIGRDRLKRIHEACGRPVGLENLAFSYSLDEVKRHGAFLEQLLEPVNGFIILDLHNLYCQLRNFDISFDEMIALYPLDRVREIHISGGSWDDSAAVPNRRIRRDTHDDAVPPEVFQLLEMTMARCPHLKYVVLEQLGNGLETATSKQCFYNDFLQMEEIVRKNSDDNDADTPDPFLPLSPLSTDTAIEDMILYQQQLELSAILESSDSYAQAMDALRQSSLANSDWKIEQWEPYMIETAVKIARKWKK
WP_234645261.1	Dyadobacter sp. CY356	MSTIFSSVACNLDTNILLASLPLFEREQIEAIEWSFDTLFKNDEVPDWFSDLILEFSSQNRLIGHGVYFSLFSGKWMSEQQDWLKKLKILGERFQFDHITEHFGFMTGANFHKGAPISIPLTASTMSLGVDRLHRIQDACNCPVGLENLAFSFSLDEVKRQGNFLDKLVESINGFIILDLHNLYCQIHNFDVDYDYIIDLYPLERVREIHISGGSWDIQLTRTGKKVRRDTHDDAVPEEVFQILSAVIPRCTNLKFVVLEQLGTALDSEVKRTRFRSDFERMDAIVKQSGLALSNSPVNNFKPHHKITPHLIPLEDLSLYNQQLQLSTILETAVDQQHARHLLLNSDLRDSVWEVEKWDPCMLQTAIAIAQKWKDGF
MCH2081132.1	Saprospiraceae bacterium	MTKIHSTLACNLDKNILMAALPLLAAEKVEAIEWSFDALFKTRNIPNWFVDLLRTYSQGQALIGHGIFFSLFSGRWSPEQQQWLNHLKRITKEFNFEHITEHFGFMTGKNFHQGAPISVPYNERTLALGQDRLKRMYEACQRPVGLENLAIAYSTAEVQQHGEFLEALLSPINGFIILDLHNFYCHLHNFEENFDRLIQSYPVHMVREIHISGGSWQNSEWFDDKKVRRDTHDDGVPEIVFELLEKVIPLCPNLKYVVLEQLGTGLKTDGQKTRFQKDFLRMDEIVQPSIPHKKTQNDFLPKQKIKFDEIVEDEVFHQQQMQLSAILENAVDAASAEKLLMNSALAKTDWKVEEWKTHMLDAVIQIAQQWKKGFQSPAN

WP_238387972.1	Hymenobacter sediminis	MQPEPAILATLACNLDADILSAALPLLAEGRVEALEWSFDALFWAEQVPDWFSELLTTYSAANRLLGHGVYFSLLSGRWTPEQQQWLEQVRQVVAQYPLAHLTEHFGFFTGQNFHSGAPLPVPYTPATLRLGQDRLSRIYEACRCPVGLENLAFAYSLDEVKRHGEFLGRLLEPVNGFLILDLHNLYCQIHNFSVSYEELIGLYPLERVREIHISGGSWENSELAPGRKIRRDTHDEGVPEEVFQLLELTIPRCPRLKYVVLEQLGPSLKTEESRIRFRHDFGRMEEVVHRHRAGRLGYADAFFLPPQPLQTGPALEDAQLHAHQQQLSHILETAPSFAEAQRQLHASALASSDWKLEQWAPYMLETAVSIAQKWK
MCG8329440.1	Chitinophagales bacterium	MYASIACNLDTNILLAALPLFETEKVEAIEWSFDALFNKAQVPAWFYELLQAYAKEKRLIGHGIFFSLFSGRWREEQEAWLAQLQEVARDFQFQHITEHFGFMTGENFHAGAPLSIPITEKTLRIGQDRLKRIYQACECPVGLENLAFSYSLEDVKAQGIFLDQLLEPINGFIILDLHNLYCQLYNFEISFEELIALYPLQRVREIHISGGSWEDSVLTPQKRVRRDTHDEAVPDEVYKLLEKTIPLCPNLKFVTLEQIGTGLENEPKRERYRQDFLKMEKIVASYTKSTPVTNDFLPISPLQVSTSPEEDQILHKQQVELSYILENAPDYSSAQQQLEASSLAQSDWKVENWAPHMLETALKIAQKWA
WP_239808446.1	Flavihumibacter fluvii	MQKIYSSLASNLDIQLLSAAYPLLAGGKVDAIEWSFDTLYKVDHIPMWFEDLLAAYSAQNRLIGHGVFFSLFSGRWSDAQQNWLAALRQTSQRYRFDHITEHFGFITGKDFHQGAPMSIPYTPATLAIGQDRLKRIQDACSCPVGLENLAFSYSLDEVKEHGEFLQRLIEPVNGFIILDLHNLYCQINNFDIGFEEIISLYPLDKVREIHISGGSWDEVSEPFPKNVRRDTHDSAVPEPVFDLLKMTIEKCPALKYVVLEQLGSALSTPESQAVFQQDFLILEEIVQEKNKGRWDFFNDAFLPSPLVIPDDIIEDEALYQQQKQLAEILEHCTTAAEAQQQMRQTTLANSAWDIEHWQPYMLETAIRIAQKWKDGFE
WP_240737107.1	Hymenobacter metallicola	MAEQLQPVGASAGTPDILSAIACNLDADILSASFPLLEVGRVEALEWSFDTLFWAEQVPEWFTELLLTYSQQQRLVGHGVYFSLLSGRWTAEQQQWLQQLQELTKRFAFDHITEHFGFFTGQNFHSGAPLPIPYTRSALHLGQDRLQRIQQACGCPVGLENLAFAYSLDEVQRHGEFLDELVEPVNGFLILDVHNLFCQLHNFAVPYEELIRRYPLDRVREIHISGGSWDDSGPTPGRPVRRDTHDGAVPEEVFQLLEQTMPRCPNLRYVVLEQLGNALKTDAQHTQFRADFCRMEALVTRHRSRAAGRAGQLFLPLQPGAAGPVAEDESLYAQQQQLSRILETAPTFEEARRQLQASPLAHSAWKIEEWEPHMLETARRIAQKWQ
WP_241286337.1	Chryseobacterium arthrosphaerae	MKKPLLGLSMMPEADFVSAILPLLQSNSVEVLEWSFDTFYDAKEPEWLDELLNFYSENQRLIGHGVYYSLFDALWTERQENWLKKLKEEVRKRKYNHITEHFGFMNTENFHQGVPLPVPLHPKTLLIGKDRLYRLQDAVELPVGIENLAFSFSIEDVKEQGVFLDNLIADINGFLILDLHNIYCQARNFEVEMEDIIRLYPLDKVREIHLSGGSWQESVYAGKMIRRDTHDDLIPEEIFSVLPSVIAQCNNLEYVIIERLGHTIKTDQEKRAFVDDFTRVRRIIDDSEIKTGKKIRWSQKETGFSNSPVEDDALYEEQSRLTKLLFDSSGAEMIKEQEFHYFKTENWDPDMILTAQNIIKKWNPY
WP_241315041.1	Chryseobacterium arthrosphaerae	MKKPLLGISMMPEADFVSAILPLLQSNSVEVLEWSFDTFYEAKEPEWLDELLNFYSENQRLIGHGVYYSLFDALWTERQENWLKKLKEEVRKRKYSHITEHFGFMNTENFHQGVPLPVPLHPKTLLIGKDRLYRLQDAVELPVGIENLAFSFSIEDVKEQGVFLDNLIADINGFLILDLHNIYCQACNFEVEMEDIIRLYPLDKVREIHLSGGSWQESVYARKMIRRDTHDDLIPEEIFSVLPSVITQCNNLEYVIIERLGHTIKTDQEKRAFFDDFTRVRRIIDDSEIKTGKKIRWGQKETGFSNSPVEDDALHEEQSRLTKLLFDSSGAEMIKEQEFHYFKTENWDPDMILTAQNIIKKWNPY
WP_242696435.1	Longitalea luteola	MTKLLATAACNLDIDMLNACYPLFEEERVQAIEWSFDALFDRGVLPPWFEGLLQVYSNAKRLIGHGVFFSLFSGKWLPEQAQWLETLRTTSRRFPLEQVTEHFGFMTGKDFHEGAPLAIPYSATTLRIGRDRLKRIQEAALCPVGLENLAFAYSMDEVKRHGDFLYELIQPVNGFIILDLHNVYCQLENFQISAEDLLSLYPLDKVREIHISGGSWEPVESEPDRRIRRDTHDDAVPAEVFSLLDMAIDRCPECKYVVLEQLGAGLQTAASRQQFYQDFLQMENMLDAKNKHRNGEAQNDFLPFHIVSNEPPVEDAQLYQQQRELSQILESDMSYNEAITALNQSSLAHSEWNMEQWHPAMLETAMQIARKWKKKV
WP_243900110.1	Hymenobacter defluvii	MAAVPAVPSILSAIACNLDDHLLAAALPLLEAGQVEAVEWSFDTLFDGRQLPNWFAELLTAYGEQGRLIGHGVYYSLLSGRWTPEQAQWLEQLRLLAARFHFDHITEHFGSFTGQNFHTGAPLPVPYTAATLRLGQDRLLRVADACQCPVGLENLAFAYSLEEVQRHGEFLEKLLAPVNGFLILDLHNLYCQLHNFNLSTDSLLALYSLHRVREIHISGGSWEYSQVEQGRQIRRDTHDEAVPDEVFALLERTIPQCPQLKYVVLEQLGTGLRTEASQHQFRQDFQRMAAIVEASRHRAPSHQPNAFQPPAALPTGPAAHDEALHAQQRQLSQILETAASYEEAQQRLRASSLAHTDWQLEQWAPHMLETAISIAQKWK
WP_240773366.1	Pontibacter sp. SGAir0037	MAWDKVVQSYLAIMKAPEVFASIACNLDTDILSAAFPLLEEGRVEALEWSFDTLYWAEQVPDWFSELLHTYSGEERLVGHGVFFSLVSGKWTKEQQQWLEHLKQVCRQFHFDHITEHFGFFTGQNFHYGAPLNIPYTSSTLRIGQDRLMRISDACQRPVGLENLAFSYSLDEVKQHGHFLEKLVEPVNGFIILDLHNLYCQLHNFSVSFEELMQLYPLHRVREIHISGGSWESSVLDPAIRIRRDTHDDAVPAEVFQLLEYALPLCPELKYVVLEQLGNGLKLESSRIQFRQDFLKMEELVKRHTTTRKPAAKNLFLPSHPLQPGAAAEDKTLQAQQYTLSHILETATSYEEALQLLRSSSLANSEWQIESWAPHMLETAISIAQKWRK
MCI5081785.1	Saprospiraceae bacterium	MKEIHATLACNLDQHVLLSALPLLAAEKVDAIEWSFDALFRTPNIPDWFVELLRTFSHARRLVGHGIFFSLFSGRWTPEQQQWLTHLRKVSHEFKFDHITEHFGFMTGENFHQGAPISVPMNERTLAIGHDRLKRMYQACERPVGLENLAIAYSLGDVERQGGFLDRLLEPVNGFMILDVHNIYCQAHNFRLDPMKIIEAYPLDRVREIHISGGSWEESEYFEGDKIRRDTHNDAVPETVFEILAKAIPLCPNLKYVVLEQIGEGLQSPHQKKRFQQDFLRLREIIQSSAQSPTAQNDFLPKGEILVGQAKEDEHLHQQQMQLSAILENAGDLASAKHLLHNSSLADTDWEIERWSDHMLDTVIQIAQKWKAGFRPPAEAGRPKQ
WP_293564951.1	Phaeodactylibacter sp.	MEHSGLQRPRATLACNLDTNLLRAAHPLLAAGQVEALEWSFDALFQFPEIPDWFTALIQAYSEEGRLVGHGVFFSLFSGRWSAEQQQWLDRLKALSEVYRFDHVTEHFGFMTGADFHKGAPLGIPFTPQTLQLGRDRLKRISAAAGCPVGLENLAFAYTLDEVKRHGDFLEQLLEPINGFIILDLHNLYCQLHNFRQSAEELLRLMPLHRVREIHLSGGSWEPAATAGNRQIRRDTHDESVPQAVFDLLEKALPKCPNLKYVVLEQLGGALETPEQQQAFQADFRTMHRILKGHNHMTGKPAPLNDFQAPAFTLSATPRSDETLHQQQQALSEILENAENLRDARTQLSASPLADTAWKVEHWEDDMLETARRIAQKWKGGWG
WP_244674893.1	Hymenobacter cellulosilyticus	MSTQGQPAGAAEPGILSAIACNLDADILSASFPLLEEGRVEALEWSFDTLFWAEQIPDWFTELLQAFAAQHRLVGHGVYFSLLSGRWTPEQQQWLQQLRALAGQFTFDHITEHFGFFTGQNFHSGAPLPIPYTSSALHLGQDRLRRIQDACQCPVGLENLAFAYSLDEVQRHGEFLDKLVEPVNGFLILDLHNLFCQLHNFAVPYEELIRLYPLDRVREIHISGGSWEESGQVPGRQVRRDTHDDAVPDEVFELLKLTMPRCPNLKFVVLEQLGNALQTEPSRAQFRHDFGRMEALVAEYRSRAVGRISQLFLPLQPWVAGPVAEDARLYEQQLQLSRILETAPSFEQAHRQLQASDLAHSAWKIEEWEPHMLETALRIAQKWQ
WP_245125988.1	Hymenobacter volaticus	MEKQPVILSSIACNLDGDILSAAFPLLEEGKVEALEWSFDTLYWAEEVPAWFTELLSAYSEQKRLIGHGVFFSLLSSKWTKEQQQWLQKLQQLSSEFSFDHITEHFGFFTGQNFHYGAPLPIPYTTTTLRIGQDRLSRIYEACQCPVGLENLAFAYSLDEVKQHGEFLDKLIEPVNGFIILDLHNLYCQLHNFSVPYDQLLPLYPLNRVREIHISGGSWEASDRGPNQKVRRDTHDDSVPEEVFQLLELTLAKCPNLKYVVLEQLGNGLKTESSRVQFRHNFLRMESIVKRNANHLCRHSNELFLPAQRIPLGPAVENEQLHEQQVQLSQILETASSLEMARQLLASSSLAYTDWKIESWEPYMLETAIHIAQKWKR
WP_245957479.1	Chitinophaga flava	MPEILSTVACNLDNDILAACLPLMEASRVEAIEWAFDTLFNVTEVPSWFEELLTAYSQENRLIGHGVFFSLFSGKWLPEQESWLKHLRKVSNDFHFDHVTEHFGFMTGKDFHHGAPLNIPYTTATLDIGRDRLKRICEACSCPVGLENLAFSYSPEEVKKHGAFLDQLLEPVNGFIILDLHNLYCQVHNFNLSFDDISMLYPLHRVREIHISGGSWENSIATPGRNIRRDTHDDAVPEEVFAYLEKAIDLCPHLKYVVMEQLGTGLYTDASKIAFRNDFLRMDDIIKNKNKTIAVANSFLPETLIVPPEVKEDLSLYQQQLELSNILETALSYETAMTALSSSSLAHSAWQIEQWDPAMIETAVNIAQKWKKKT
WP_241614085.1	Chryseobacterium sp. SSA4.19	MKPYLGLSMMAETDFVSAILPLLQNSEIEVLEWSFDTFFNAEEPNWLSNLIDFYSDNNRLIGHGVYYSLFDAKWTSRQQQWLDSLKLETQKRKYSHITEHFGFMNTENFHQGIPLPVPLCSKTLQIGKDRLSRLQNVLEIPVGLENLAFSFSADDIKQQGDFLDQLIRDIDGFLILDLHNIYCQAHNFRMDIKEIINSYPLNKVKEVHLSGGSWQESVYGKISMVRRDTHDDCIPKELFSILPFVLEKCHHLNYIIIERLGNSIKTKKEIQNFLHDFRRVRKIMDDSSAESVIEKWVKPEFLQEEPLEDLLLYQEQALLTKMLFEKENQETIRNYPFTYFNTASWDLEMIKTAENIIKKWNPY
WP_245096096.1	Hymenobacter aerilatus	MSAPAILSAIACNLDDQTLTAALPLLEAGQVEAVEWSFDTLFDGRQLPDWFVELLTAYGEQGRLVGHGVYFSLLSGRWTPEQAQWLEQLRLLAGRFRFDHITEHFGAFTGQNFHTGAPLPVPYTAATLRLGQDRLLRVADACQCPVGLENLAFAYSLEEVQRHGEFLEQLVAPVNGFLLLDLHNLYCQLHNFNLLSDSLLALYPLHRVREIHISGGSWEDSRVEQGRQIRRDTHDEAVPDEVFSLLKRTIPKCPQLKYVVLEQLGTGLRTDASQDRFRQDFQRMAAIVEASRCHAHSHQPNAFQPPASLPAGPAAHDETIHTQQRQLAQILETAASYEEAQQRLRASSLAHTDWQLEQWAPHMLETAVRIAQKWKQ
WP_245999635.1	Paraflavitalea soli	MSKILSTVACNLDNDILGACLPLMEESRIEALEWSFDTLYKVQEIPTWFQELLTAFSKEKRLIGHGVFFSLFSGKWLPEQEAWLKALQQTCSEFQFDHITEHFGFMTGKNFHQGAPLNIPYTPTTLAIGQDRLKRIYAACQCPVGLENLAFSYSLDEVKRHGRFLDKLLEPVNGFIILDLHNLWCQLHNFSIALEELIGLYPLDRVREIHISGGSWDHSQIEKGRTIRRDTHDDAVPAAVFQLLETVIPACPHLKYVVLEQLGNGLESIESKQQFYADFLEMESIIRRQSQPAYLTNLASFLPLSPIVMGPPVEDEGLYEQQAALSTILETSHSYEEARTLLGQSSLACSEWQVENWDPAMIETAMKIAQKWKAGFVGFIS
WP_246000679.1	Pontibacter diazotrophicus	MNGKELHPVGKEPEILSTIACNLDADILSAAFPLLEEGRVEAIEWSFDTLYWAAQVPYWFTDLLLAYSQQSRLIGHGVFFSLLSGKWTKEQQHWLEQLKQLSSQYSFDHITEHFGFFTGQNFHSGAPLYVPYTSTTLRIGQDRLSRIYDACQCPVGLENLAFSYSLDEVKQHGEFLEKLVAPVNGFLILDLHNLYCQLHNFSITYEDLITLYPLNRVREIHISGGSWEASVLEPEKKVRRDTHDDAVPEEVFHLLELTVPKCSNLKYVVLEQLGNGLKTESSRNRFRQDFLKMEALVQKSRSSLSRQDDNLFLPVQPLQTGPVVEDDMLHDQQLQLSHILETAPSYEEARRLLFTSSLAGSDWKIENWAPHMLETAVSIAQKWKR
WP_246197742.1	Chitinophaga agrisoli	MPKILSAVACNLDVDILGACLPLMEASKVEAIEWSFDALFKVKEIPSWFLELLTAFSDEQRLIGHGVFFSLFSGKWLQEQEDWLRQLEHTSTAFRFDHVTEHFGFMTGKDFHHGAPLNIPYTNATLTIGRDRLRRIQAACQCPVGLENLAFSYSLEEVKQHGAFLDELLAPVNGFIILDLHNLYCQLHNFEIPFDTLISLYPLDKVREIHISGGSWEDSLAAPPKKVRRDTHDDAVPDAVFELLENTIDLCPHLKYVVLEQLGNGLITAESKTQFQQDFLRMEAIVAEKNATRSTESIQSFLPAHPTIPAHIAEDATLYRQQLELSAILETAATYNDAVHALQTSSLAHSDWRIETWAPYMIETAMKIAQKWKK
WP_247814364.1	Chitinophaga filiformis	MPEVLSAIACNLDANILAACLPLMEESRVEAIEWSFDALYKVKEVPSWFRELLTAFGNEQRLIGHGVFFSLFSGKWLPEQERWLRHLEQTATEFRFDHITEHFGFMTGKDFHHGAPLNIPYTAATLNIGRDRLKRIYHACRRPVGLENLAFSYSLDEVKRHGAFLEQLLEPVNGFIILDLHNLYCQLRNFDISFDEMIALYPLERVREIHISGGSWDDSAVDPSRRVRRDTHDDAVPDEVFHLLEMTMPRCPHLKYVVLEQLGNGLSTAISRQCFYNDFLQMEEIVRKHNHDRDTGSSDPFLPISPLSIDAVVEDMTLYQQQLELSAILESSGSYAEAMYALRQSSLAGSDWRIEQWEPYMIETAVKIAQKWKK
WP_246269322.1	Chitinophaga oryzae	MSKLVSTVACNLDNNILAACLPLMEAAKVEAIEWSFDALYAAENVPAWFEELLAAFSKENRLIGHGVFFSLFSGRWLPEQQQWLDHLKTVSRHYRFDHVTEHFGFMTGKDFHHGAPLNIPYTPVTLGIGRDRLKRMQDACGCPVGLENLAFSYSLEEVKRHGDFLNELLEPVNGFVILDLHNLYCQAHNFDLSFEALKDLYPLHRVREIHISGGSWDESEATPGKTIRRDTHDDAVPATVFNYLDKAIDLCPSLKYVVLEQLGSGLASEESRSAFRNDFLRMDQLLNSKNRTTAPLNTFLPEAFQVPAQVPEDETLYRQQLELSQILETAASFDAAMKALQASSLAHSAWQIENWDPAMIETAAKIAQKWKRKDIVFKT
WP_250225925.1	Chryseobacterium gallinarum	MEKPFLGISMMAEADFLTAILPLLRDGSIDVLEWSFDTFYHAEEPDWLTRLLNFYSQNNRLLGHGVYYSLFDAKWTERQSMWLQKLKEEVQQRKYNHITEHFGFMNTENFHQGVPLPVSLHPKTLEIGKDRLCRLQEVLEIPVGIENLAFSFSIDDVKQQGIFLEKLIEDTDGFLILDLHNIYCQACNFGLGIEEIVRLYPLEKVREIHLSGGSWQTSPYGKRRIRRDTHDDTVPAEILTVLPSVLSQCENLEYVIIERLGHTIQTETEAKDFLDDFLKVKAIIGASEWNSRHKEIKIKKKPECAGRPVEDEGLYREQTILTKLLFDNVKADHFKDFDFRYFKTENWDPEMIRTAQNIIKKWNPY
MCP5090420.1	Gammaproteobacteria bacterium	MIEEDFLEAARPLFDTDTVEVLEWSFDIGWQQPSVSRRVEEIIDEYDRRGRLLGHGVTYSPMSAEFSEHQRQWLRHLERECRRRQYIHVSEHFGFMAAGGFAEAAPLPVPRTRATLGVGQDNFKRLKDAAQVPIGLEDLAFAFCERDVIEQGRFLDELLEPVDGFLVLDLHNVYCQSRNYGIPFDELLQTYPLDRVREIHVSGGSWSTLWHSAESRRVRRDTHDNSVPEEIFRGVAAMLQRCDGVVAVILERIGGSLGSELALREFRRDYERLRKIVDDFRNR
WP_255814981.1	Chryseobacterium sp. MA9	MKKPLLGISMMAEADFVSAVLPLLQNNAIDVLEWSFDTLYHTNEPDWLRDLLNFYAENNRLIGHGVYYSLFDARWTERQEEWLQKLNEEVSLRKYNHITEHFGFMNTENFHQGVPLPVSLHSKTLQIGKDRLYRLQEAVDIPIGIENLAFSFSIDDVKEQGVFLDRLTEDTNGFLILDLHNIYCQSCNFEVEMREIIDLYPLDKVKEIHLSGGSWQESVYGKKQVRRDTHDDVIPETIFSVLPSVLEQCQNLEYIIIERLGHTLKTEEDKENFLNDFNKVKTVIEASDWKRREKGSWSKQKIQIPKKPVEDLALFEEQSRLTRLLFDNAGAVVIKDQDFHYFKTDNWDPEMILTAQNIIKKWNPY
MCP4807725.1	Pseudomonadota bacterium	MSERHIGVGLSVMPEPAWRDAAVPLLSDGQVDLLEWSFDTGWGRDLPAWLDPLLDFASENGVLWGHGVSYSLLTAGFPERQEAWLDNLRRELDRRSYVGISEHFGFCHAEGFRRAPPLPVPWCEATVRIGQQRLARLAEVAGVPVGLENLAFAFGRRDMLDQGPFLDELLSAVDGYLVLDVHNLWCQVQNFGVEAGSLLSRYPLDRVRVAHVSGGSWSIAGSSPWRRDTHDGPVPDEVFALLPTVINICPSLEAVVLERLGTSLEGQGQAFRDEFLRLRQVVG
WP_246601894.1	Hymenobacter profundi	MAAVPAVPSILSAIACNLDDHILAAALPLLEAGQVEAVEWSFDTLFDGRQLPNWFAELLTAYGEQGRLIGHGVYYSLLSGRWTPEQAQWLEQLRLLATRFHFDHITEHFGSFTGQNFHTGAPLPVPYTAAILRLGQDRLLRIADACQCPVGLENLAFAYSLEEVQRHGEFLEKLLTPVNGFLILDLHNLYCQLHNFDLSTDSLLALYPLHRVREIHISGGSWEDSRVEQGRQIRRDTHDEAVPDEVFALLERIIPQCPQLKYVVLEQLGTGLRTEASQHQFRQDFQRMAAIVEASRHRAPSHQPNAFQPPAALPTGPAAHDEALHAQQRQLSQILETATSYEEAQQRLRASSLAHTDWQLEQWAPHMLETAISIAQKWK
WP_250254317.1	Chryseobacterium sp. Marseille-Q3244	MGRPLLGLSMMAESEFISAVLPLLQSNSIEVLEWSFDTFYNTEEPVWLSELLNFYSENNRLIGHGVYYSLFDALWTERQELWLEKLKEEFHKRKYKHITEHFGFMNTENFHQGVPLPVCLHSKTLEIGKDRLYRLQDAVEVPVGIENLAFAFSLDDVTEQGVFLDKLTEDIDGFLILDLHNIYCQSCNFNVDMRDIIKLYPLNKVREIHLSGGSWQESVYSKTPVRRDTHDDAIPKEILSILSSVVFQCKNLEYIIIERLGHTINTEEKKKSFLDDFAQVRTLIDSSDWEGVNKDLWNKKKVQFQKTPLEDMILNEEQTLLTKLLFDNAGVASIKNHEFHYFKTKDWDPEMILTAQNIIKKWNPY
WP_257200057.1	Chryseobacterium sp. WG14	MEKPLLGLSMMAEADFVSAILPLLQNNAVDVIEWSFDTFFEAEEPSWLKDLLDFYAGNDRLLGHGVYYSLFDARWTERQEMWLQKLKQEVQKRNYNHITEHFGFMNTENYHQGVPLPISLHPKTLEVGKDRLYRLQDAVDIPIGVENLAFSFSVNDVREQGVFLDKLIDDINGFLILDLHNIYCQSCNFEVSMQDIVGLYPLEKVKEIHLSGGSWQDSVYGKKMIRRDTHDDVIPEEIFAVLPSVLSQCHNLEYIIIERLGHTIHTEEEKQCFFNDFMRIKRIISYSDTMNGKAEKWVKRERLLSEPLEDLIIYEEQIRLTKLLFDHADPTLIKNQNFHYFKTKNWDPEMILTAQNIIKKWNPY

WP_255373044.1	Chitinophaga sp. YR573	MPKILSAVACNLDANILSACLPLMEESRVEAIEWSFDALYKEKEVPSWFRELLTAFSNEKRLIGHGVFFSLFSGKWLPEQEAWLKQLEQTAAEFRFDHITEHFGFMTGKDFHHGAPLNIPYTTTTLNIGRDRLKRIYEACRRPVGLENLAFSYSLDEVKRHGTFLEQLLEPVNGFIILDLHNLYCQLRNFDIAFDEMIALYPLDRVQEIHISGGSWDDSAADPNRSIRRDTHDDSVPPEVFQLLEMTIAQCPHLKYVVLEQLGNGLVTEMSKRCFYNDFLQMEEIVRKNNNENDAGASDPFLPLFPLSTGTAVEDMMLYQQQLELSSILESSASYAEAMHVLSQSSLANSDWRIEQWEPYMIETAVKIAQKWKK
WP_257883251.1	Hymenobacter sp. DG01	MQANPAICSALACNLDVDILSAALPLLAEGRVEAVEWSFDTLFWTEQMPDWFSELLRTYSAGNRLLGHGVFFSLLSGRLSPEQQQWLSQLRKVAAELPFAHITEHFGFFTGQNFHSGAPLPVPYTPATLRLGQDRLSRLYEACRCPVGLENLAFAYSLDEVKRHGEFLDKLLEPVNGFLILDLHNLYCQLHNFSVSYEELIRLYPLHRVREIHISGGSWEPSALAPEQKVRRDTHDQGVPEEVFQLLQRTMPQCPRLRFVVLEQLGNSLRTEENRARFRLDFGRMETLVQEHRASLTVSAAEQAFLPLQPPRRGPVLEDPALHEQQQHLSRILETAPSVAEARQLLHTCPQLSATAWKIEQWEPYMLETAVGIAQKWKK
WP_258539328.1	Chitinophaga oryzae	MSKLVSTVACNLDNNILAACLPLMEAAKVEAIEWSFDALYAVENVPAWFEELLNVFSEENRLIGHGVFFSLFSGRWLPEQQQWLDHLKTVSRHYRFDHVTEHFGFMTGKDFHHGAPLNIPYTPVTLGIGRDRLKRMQDACGCPVGLENLAFSYSLEEVKRHGDFLNELLEPVNGFVILDLHNLYCQAHNFDLSFEALKDLYPLHRVREIHISGGSWDESEATPGKTIRRDTHDDAVPATVFNYLDKAIDLCPSLKYVVLEQLGSGLASEESRSAFRNDFLRMDQLLNNKNKSTVPLNSFLPEALQIPAQAQEDETLYRQQLELSQILETAASFGATMKALQASSLAHSDWQIESWDPAMIETAAKIAQKWKRKTTNP
WP_259830879.1	Chryseobacterium pyrolae	MSRPLLGLSMMPEADFVSAILPILQNNSVDVLEWSFDTFYDADEPDWLSGLLDFYAENNRLLGHGVYYSLFDARWTDRQEVWLKKLKEELRHRKYNHITEHFGFMNTENFHQGVPLPVPMHSRILQIGKDRLRRLQDVVDIPIGVENLAFSFSIDDVKEQGEFLDKLVEDIDGFLILDLHNIYCQSCNFEVDMQEIIKLYPLEKVKEIHLSGGSWQESAYGKKPVRRDTHDDRIPGKIVEILPEVFSQCPNLEYVIIERLGHTLNVESEKQTFFADFNRVKEMIEMSDDLPGNRQVWNKKKGEYSKPVEDLLLYDEQTKLTKLLFDEESTGSVKNQKFHYFKPESWDEEMIMTAQQIIKKWNPY
WP_257188871.1	Chryseobacterium sp. WG23	MEKPLLGLSMMAEADFVSAILPLLQNNAVDVIEWSFDTFFEAEEPSWLKDLLDFYAGNDRLLGHGVYYSLFDARWTERQKMWLQKLKQEVQKRNYNHITEHFGFMNTENYHQGVPLPLSLHPKTLEIGKDRLYRLQDAVDIPIGVENLAFSFSVNDVREQGVFLDKLIDDIDGFLILDLHNIYCQSCNFEVSMQDIVGLYPLEKVKEIHLSGGSWQDSVYGKKMIRRDTHDDVIPEEIFALLPSVLSQCHNLEYVIIERLGHTIHTEEEKQCFFNDFMRVKKILNDSGGMKRKNEKWVKKERLLSEPVEDLIIYEDQIRLTKLLFDHADPTLIKNQNFHYFKTKNWDPEMILTAQNIIKKWNPY
MCR9102186.1	bacterium	MEHADLQRPRATLACNLDTNLLRAAHPLLAAGEVEALEWSFDALFQFPEIPDWFTALIQAYSEEGRLVGHGVFFSLFSGRWSAEQQQWLDRLKALAEVYRFDHVTEHFGFMTGADFHKGAPLGIPFTPQTLQLGQDRLQRISAAAGCPVGLENLAFAYTLDEVKRHGDFLEQLLEPINGFIILDLHNLYCQLHNFRQSAEELLRLMPLHRVREIHLSGGSWEPAAGESKRLIRRDTHDEAVPQAVFDLLEKALPKCPNLKYVVLEQLGGALETPRQQRAFQADFRTMHGILKRQNHSGKTSAPANDFQPPAFTLMATPRSDEALHQQQQALSEILENAANLRTARAQLSSSPLAETAWNVEDWEDDMLETARRIAQKWKGGWG
MCR9285899.1	Bacteroidota bacterium	MSKILSAIACNLDQHILQTALPLFAEEKVQAIEWAFDTLFNHKNIPDWFVELLESFGKEGRLIGHGVFFSLFSGKWKPEQDEWLLRLKRMSNHFHFNHVTEHFGFMTGENFHKGAPISIPYTPTTLSIGHDRLKRIAEAAQCPVGLENLAFAYSMEEVKKHGDFLNALIEPINGFIILDLHNLYCQMHNFEVSFEEIIELYPLDRVREIHISGGSWEDSDLQPSKKIRRDTHDDSVPKEVFQILEKSIPKCPNLKFVVLEQLGLGLKTEESKINFRNDFLKMDKIISGVNSKNENIRQKSFLPSTNFQLKAPLESIQLHHQQLELSQILENALHYENAQHLLQDSSLAQSDWQIENWSPHMVETVIKIAQKWKAGFN
WP_258049066.1	Hymenobacter sp. NBH84	MKLSKALLGAVLVGITAQTTACTKGDEPAPKGEKVAKGSEKKNARLLPRVWPGLSNAPETMAAVPALPSILSAIACNLDDHLLAAALPLLEAGQVEAVEWSFDTLFDGRQLPNWFAELLTAYGEQGRLIGHGVYFSLLSGRWTPEQAQWLEQLRLLAARFHFDHITEHFGSFTGQNFHTGAPLPVPYTAAILRLGQDRLLRIADACQCPVGLENLAFAYSLEEVQRHGEFLEKLLAPVNGFLILDLHNLYCQLHNFDLSTDSLLALYPLHRVRGNPHFGRQLGRFAGGAG
WP_259835626.1	Chryseobacterium herbae	MKKPLLGLSMMPEADFVSAILPILQTHSVEVLEWSFDTFYDVEEPEWLSGLLDFYAGNNRLLGHGVYYSLFDARWTDRQETWLKKLKEEVSRRKYNHITEHFGFMNTENFHQGVPLPVPLLPKTLEIGKDRLIRLQDAVEIPVGVENLAFSFSLDDVKEQGEFLDKLVEDIDGFLILDLHNIYCQSCNFEVDMKEIIKLYPLEKVKEIHLSGGSWQESAYGKKPVRRDTHDDRIPEEILQMLPEVLIQCHPEYVIIERLGHTLNTEAEKEFFFDDFNRVKEIIEFSDHPVDHEKIWNRKNSTYAQPIEDLLFHDEQTELTRLLFDGNSTEAIKNRNFHYFKPENWDEEMIITAQQIIKKWNPY
WP_261511996.1	Chryseobacterium paludis	MKKPLLGLSMMAEADFVSAILPLFENNQIEILEWSFDTFYDVEEPEWLKGLLDFYSQNNRLIGHGVYYSLFDAKWTERQEEWLNNLKTETEKRHYNHITEHFGFMNTENFHQGIPLPVSLNSKTLQIGKDRLLRLQDVVNVPIGIENLAFSFSIEDVKQQGQFLDQLITDIDGFLILDLHNIYCQAYNFDVQLEEIISAYPLEKVKEIHLSGGSWNQSIYTKNKLIRRDTHDDAIPEEILNILPFVLPKCENLQYIIIERLGCTIKTKDEKEAFFDDFRRVREIIDSIEYHENNYPAIWEKKNVNLVSPLEDLQLYEDQTRLTQLLFDDKPELIKSQTFHYFKTDDWDIEMINTAKSIIKKWNPY
WP_260545184.1	Chryseobacterium oranimense	MKKPLLGLAMMPEADFVSAILPVLQNNSVDVLEWSFDTFYDVEEPEWLSGLLDFYAENDRLLGHGVYYSLFDARWTSRQKDWLQKLKEEFKHRKYNHITEHFGFMNTENFHQGVPLPVPLHSKTLQIGKDRLKRLQDTVEVPMGVENLAFSFSIDDVKEQGEFLGKLIEDIDGFLILDLHNIYCQSCNFEIDMQEIIRMYPLEKVKEIHLSGGSWQESAYGNKPVRRDTHDDYIPEEIINVLPEVFSQCLNIEYVIIERLGHTLDTEVKKQLFFDDFKKVKAIETSDFPAGITKEWNRKKREYSEPVEDMLLYEEQTKLTKMLFEGNTVQSVKNEPFHYFKPENWDKEMITTAQQIIKKWNPY
MCU0533392.1	Hydrococcus sp. Prado102	MFRQKQTLPWVGLSLMADPSFFQATRPLFEAGDVDVLEWSFDVGWGLESLPSWVEQLLEFYSQRDRLLGHGVSYSLLSAQWSDRQAQWLKLLKSECQRYRYRQISKHFGWMAAGNFERSAPLPMPLTRDTLRIGCMNLRRLTEVTSSPIGLENLAFAFGLPDVLNQGNFLEQLLQSVNGFLVLDLHNVFCQIHNFDRAADEIINSYPLHRVREIHISGGSWSQSTRGDRGLMRRDTHDGDVPEEVFQLLALVLKKCPTTESIILERLGKTLDDEAKMARFRQDFYRIKEIVKNSLCKQKN
MCU0328123.1	Chitinophagales bacterium	MYSTLAINPYPHLLTTALPLLEAGKVEALEWSFDTLPFPQIPEWFQELLQTYSQKDRLIGHGVFFGLMKGNFSQNQQEWLNQLKGISKKIKFHHISEHFGYFSGRDFHSGAPLPVPMNKITLDIGIDRLQRIQEASECPVGLENLAFAGNIEEVKYHGEFLDKLLSPVQGFIVLDLHNIYCQMVNFDLRLEEILASYPLNKVREIHISGGSWDTVSSLKKIRRDTHDDSVPTEVMTLLKKTIPLCPCIEFITLEQMDYALITDQMQQNFRNDFEEIQKIVFSTSLPKSDLIITNKENSDIHLYATPIESEELNRAQELFSTLLEDSDNLNQLKINLFKVQNTLSILEVNQWQDDMLETAMRIAKKWR
WP_264518140.1	Chryseobacterium viscerum	MKKPLLGISMMAEADFVSAVLPLLQNNAIDVLEWSFDTLYHTNEPDWLRDLLNFYAENNRLIGHGVYYSLFDARWTERQGKWLQKLKEEVSLRKYNHITEHFGFMNTENFHQGVPLPVSLHLKTLQIGKDRLYRLQEAVDIPVGIENLAFSFSIDDVKEQGVFLDKLTEDTDGFLILDLHNIYCQSCNFEVEMQEIIDLYPLDKVKEIHLSGGSWQESVYGKKQVRRDTHDDVIPETIFSVLPSVLEQCQNLEYIIIERLGHTLKTEEDKENFLNDFNKVKTMIEASGGKEREKSSWSKQEIQFSKKPVEDPALFEEQSRLTRLLFDNVGTAVIKDQDFHYFKTDNWDPEMILTAQNIIKKWNPY
WP_265130928.1	Chryseobacterium oranimense	MNKPLLGLAMMPEADFVSAILPVLQNNSVDVLEWSFDTFYDVEEPEWLSGLLDFYAENDRLLGHGVYYSLFDAQWTSRQKDWLKKLKEETKRRKYNHITEHFGFMNTENFHQGVPLPVPLHAKTLQIGKDRLKRLQDTVEVPVGVENLAFSFSMDDVKEQGEFLEKLVEDIDGFLILDLHNIYCQSCNFEIDMQEIIRMYPLERVKEIHLSGGSWQESAYGNKPVRRDTHDDNIPVEITDVLPEVFSQCPNLEYVIIERLGHTLNTEVKKQLFFDDFNKVKEIIEVSDFPAGIKKAWNKRKGESSEPVEDLLLYEEQTKLTKILFEGNTVQSVKNETFHYFNPENWDKEMITTAQQIIKKWNPY
WP_273635928.1	Chryseobacterium rhizosphaerae	MGKPLLGLSMMAEADFVSAILPLLQDNAVDVVEWSFDTFFAAEEPSWLKDLLNFYAGNGRLLGHGVYYSLFDARWTERQEMWLQKLKHEVQKRNYNHITEHFGFMNTENYHQGVPLPLSLHPKTLEIGKDRLYRLQDAVPIPIGVENLAFSFSVDDVREQGVFLDKLVDDIDGFLILDLHNIYCQSCNFDVSIQEIVSLYPLEKVKEIHLSGGSWQDSVYGKKMIRRDTHDDVIPEEIFSVLPSVLSQCHNLEYVIIERLGHTIHTEEEKQRFFNDFMRVKSILNDSDGMKRKKEKWVKREMLLPEPVEDSILYEEQIRLTKLLFDHIDPTLIKNQNFHYFKTENWDPEMILTAQNIIKKWNPY
WP_276161883.1	Chryseobacterium arthrosphaerae	MKKPLLGLSMMPEADFVSAILPLLQNNSVEVLEWSFDTFYEAKEPDWLDELLNFYSENQRLIGHGVYYSLFDALWTERQENWLKKLKEEVRKRKYSHITEHFGFMNTENFHQGVPLPVPLHPKTLLIGKDRLYRLQDAVELPVGIENLAFSFSIEDVKEQGVFLDNLIADINGFLILDLHNIYCQACNFEVEMEDIIRLYPLDKVREIHLSGGSWQESVYARKMIRRDTHDDLIPEEIFSVLPAVITQCNNLEYVIIERLGHTIKTDQEKRAFFDDFTRVRRIIDDSEIKTGKKIRWGQKETGFSNSPVEDDALHEEQSRLTKLLFDSSGAEMIKEQEFHYFKTENWDPDMILTAQNIIKKWNPY
WP_267406217.1	unclassified Chryseobacterium	MKPHLGLAMMAEADFVSAILPLLQSNEIDVLEWSFDTSYNADEPDWLNELLDFYSENNRLIGHGVYYSLFDAKWTDRQEDWLKKLKSETQKRHYNHITEHFGFMNTENFHQGIPLPISLHQKTLQIGKDRLSRLQDVLNVPVGIENLAFSFSIDDVKEQGEFLEKLIEDIDGFLILDLHNIYCQAHNFDVDIQDLINSYPLDKVKEIHLSGGSWQKSVYGKEKLIRRDTHDDAIPEELFNVLLYVFSKCNNVEYVIIERLGNTITTGIEKENFFDDFKKVREIMNVSDFQLNSKKWNKPDLSLTEPLEDLSLYKEQVLLTKMLFENEKPELIKEYPFSYFNTENWDLEMLNTARNIIKKWNPY
WP_269427484.1	Pedobacter punctiformis	MNKPYLGISIMPEHDFLQAVLPLLQDGDIETIEWSFDTIKFEKYKPKWLNRLLQEYADNNRLIGHGVYYSLFQAKWSKEQANWLKKLKKEVEKYNYRHISEHFGFMSDHVDHHKGCPFPVPLNKTTLAIGIDRLQRLQDAAQLPVGVENLAFSFSKYDVKRQGEFITKLIEPINGFVILDLHNIYCQSENFGIEMMDLIDCYPLDSVKEIHISGGSWQESVYQGVIKRIRRDTHDGNVPKKIFDVLPAVLQKCKSVEFVIFEKMGNSFQKPGDFVDYRNDFNKLKNIIENTTLKFEAKEWGNKIIPPKNPIEDGLLFTEQNLLSRTLLESSNYKELLSKISLKNWEIDKWDPRMIDTAIALSKKWS
WP_276349236.1	Daejeonella sp. JGW-45	MPAIIPSIACNLDADILSASLPLFSEHRVGGIEWSFDALYGIEQVPEWFTELLTAYSNERRLVGHGVYFSIFSGRWSEDQQNWLNELKLLSQKFKFDHISEHFGFMTGKSFHHGAPLSIPYTGSTLRLGRDRLSRIYDACRCPVGLENLAFSYSLDEVKRHGEFLEKLVEPLNGFIILDLHNLYCQVHNFSADFEDLIKLYPLDRVREIHISGGSWEQSVLTGKQVRRDTHDESVPDEVFDLLRKTLSRCPNLKYVVLEQLGDALKTSGSRKAFYSDFIEMESIIAAGSKSRPDTQTNLFSPDYNLVSDQVAEDHDLYLQQRELSGILETSGSYEQAVDRLKLSSLHNSEWDTQNWQPYMLETAMNIAQKWKSTDD
WP_276484500.1	Paraflavitalea pollutisoli	MLEACLPLLEEARVEALEWSFDVLYQTAETPAWFHDLLTAFSKEERLIGHGVFFSLFSGRWLPEQDVWLQHLRTCCATFHFDHITEHFGFMTGTDFHQGAPLNIPYTASTLAIGQDRLKRIYEACACPVGLENLAFAYSIDDVQRQGSFLDELLHPINGFIILDLHNLWCQLHNFSIPYEQLIHLYPLDRVREIHISGGSWDDSLLETGRRIRRDTHDDAVPAAVFDLFRQTIPRCPHLKYVVMEQLGNGLTSHKSKQQFYADFVTMASIVDTANQQREAGATHAFLPAEQLYLGQPLEDETLYQQQRELANILESAGSFDAARHLLLTSSLAGTAWHVEQWDAAMIETAVKIAQKWKEGF
WP_277235126.1	Hymenobacter sp. YC55	MHPDSAMEKQPTILSSIACNLDANILSAAFPLLEEGKVEALEWSFDTLYWAEQVPDWFTELLSAYSEQKRLIGHGVFFSLLSSRWTKEQQQWLQKLQQLSLEFNFDHITEHFGFFTGQNFHYGAPLPIPYTDITLRIGQDRLKRIYEACQCPVGLENLAFAYSLDEVKQHGEFLEKLIEPVNGFVILDLHNLYCQLHNFSVSYDELIALYPMDRVREIHISGGSWEDSALGPDQKVRRDTHDDSVPKEVFQLLERTICRCPNLKYVVLEQLGNGLKTESSRTLFRHDFLEMERIVKLNASSLSTRPNELFLPPQLLLTGPAVEDEPLYEQQLQLSQILETAPSLERARQLLAASSLAQSDWKIESWEPYMLETAIHIAQKWKK
WP_276964884.1	Chryseobacterium sp.	MLGVSMMAEADFVSAVLPLLQNNSIDVMEWSFDTLYHANEPDWLRDLLNFYAENNRLIGHGVYYSLFDARWTERQEEWLKKLKEEVNLRKYNHITEHFGFMNTENFHQGVPLPVSLHPKTLQIGKDRLYRLQEAVNIPVGIENLAFSFSIDDVKEQGVFLDKLTEDTNGFLILDLHNIYCQSCNFELEMKEIIDLYPLDKVKEIHLSGGSWQDSVYGKKQVRRDTHDDVIPRDILSVLPSVLTQCENLEYVIIERLGHTLKTDEERDHFLDDFSKVKTIIETSELNGKEKSNWSKMKVELSEKPLEDPVLYEEQSRLTQLLYDNADVKAIKDLDFYYFKTENWDSEMILTAQNIIKKWNPY
WP_264691061.1	Hymenobacter sp. YIM 151858-1	MYATIACNLDADLLATAFPLLQQGRVEALEWSFDTLYGAGQMPEWFTALLRTYAEAGRLVGHGVFFSLLAGKWSPEQQQWLAHLGQLAGQFRFDHITEHFGFFTGQNFHHGAPLPIPYTAAALRIGQDRLRRIHDACRCPVGLENLAFAYTLEEVKRHGAFLEELLLPVNGFLILDLHNLYCQLHNFGVAYDALIALYPLERVREIHISGGSWHDSEQAPGRRIRRDTHDGAVPDEVFRLLALTIPRCPNLKFVVLEQLGHALHTESSRTQFRQDFGRLEHLVAQHRAALRPEPTNDFRPRQTALPAAPAAEDEHLHEQQRQLSDILENAPSYAEAERQLRASALAHSDWRIEQWQPHMLETALHIARKWKP
WP_277694591.1	Chryseobacterium sp. WX	MRKPILGVSMMAEPDFVSAILPLLHNNSIEVMEWSFDTLYHTNEPDWLRDLLNFYAENNRLIGHGVYYSLFDARWTARQEEWLKKLKEEVSLRKYNHITEHFGFMNTENFHQGVPLPVSLHSKILQIGKDRLYRLQDAVNIPVGIENLAFSFSIEDVKEQGVFLDKLTEDTNGFLILDLHNIYCQSCNFELEMQQIIDLYPLDKVKEIHLSGGSWQDSVYRKKQVRRDTHDDVIPKDILSVLPSVMKKCPNLEYIIIERLGHTLKTEEERNYFLADFNKVRTIIETSDLKLKQQNGWCKEEIKLPAKPLEDLLLYEEQSRLTRLLYENANVENIKHLDFHYFKTENWDAEMILTAQNIIKKWNPY
WP_276878162.1	Chryseobacterium joostei	MRKPLLGLSMMAETEFVSAILPLLQSNSVDVLEWSFDTLYHTKELDWLGDLLNFFSENNRLIGHGVYYSLFDARWTERQNIWLNKLKKEVKKRKYNHITEHFGFMNTENFHQGVPLPVSLHSKTLQIGKDRIFRLQEALDIPVGIENLAFSFSIDDVREQGLFLDKVIEDTDGFLILDLHNVYCQSCNFEVEMQEIINSYPLDKVKEIHLSGGSWQNSVYGKKLIRRDTHDDVIPEEILSVLPWIFSQCQNLEYVIIERLGHSILLESEKDAFIEDFAKVEKLISESDWRNQKKINWNKKELKLSESPLEDMLLHEEQTMLTKLLFDSADVSLIKNQEFHYFKTDTWDTEMILTAQNIIKKWNPY
WP_029449562.1	Cellulophaga baltica	MGNKKPKLGIAIAPSLKFLEAALPLFAGEKIEIIEWSFDTLKDAADEPAWLSLLLKEYGEKNRLLGHGVFYSLLDANWSSRQENWLKKVRQETLSHKYCQISEHFGLMSSANAHSGFPLPIQLSNPVLQIGIDRLKRLQATAQVDVGIENLALTANVADILEQGEFLFKLVNPVNGFVILDLHNIYCQSENFNIDMMTIIKSYPLSLVKELHISGGSWDTDPTLSRKIRRDTHDGRIPEVIFEILPEVLKICPFLAFIIFEKIEDSFLTEKDGIDFRADFQKIREIIDATAFSVTPKEKKQVAAILGPPVIDVELLQAQVALRESIALGTYHTNTKWDKDMWKVATKLYEKWNNY
WP_168737805.1	Chitinophaga eiseniae	MEAARVEAIEWSFDAVEQAPSWFEELLTVYSQENRLIGHGIFFSLFSGKWLPEQQQWLSHLHKISRRYHFDHVTEHFGFMTGKDFHHGAPLNIPYTPVTLNIGRDRLKRIQGACECPVGLENLAFSYSLEEVKKHGDFLERLLEPVNGFIILDLHNLYCQAHNFGLSFEALKELYPLHLVREIHISGGSWEDSDAMQGRIVRRDTHDNAVPEAVFIYLEKAIDLCPSLKYVVMEQLGTALTTTDSQATFRSDFLRMDQIIRNKNRMTASLNTFMPLPLSIQPRPLEDIPLYQQQLELSDILETAGSFDTLMEALQASSLAHSDWRIERWDAAMIETAASIAQKWKKK

WP_169229115.1	Chitinophaga fulva	MEEARVEAIEWSFDALYAVEEVPAWFGELLSVFSNENRLIGHGVFFSLFSGRWLPEQQQWLNDLKAVSRRYQFDHVTEHFGFMTGRDFHHGAPLNIPYTPVTLEIGRDRLKRMQDACSCPVGLENLAFSYSLEEVKKHGDFLNELLEPVNGFMILDLHNLYCQAHNFNLTFEALNSLYPLHRVREIHISGGSWEVSGATPGKTVRRDTHDDAVPEEVFAYLDKAIDFCPSLKYVVLEQLGSGLDCDSSRAAFGHDFLRMDELIKYKNKTAAPLNNFLPEALQIPAQAREDESLYQQQLELSQILETATSFDALMKALQASSLAHSAWQIESWDPAMIETAAKIAQKWRRKDPIF
WP_157303704.1	Chitinophaga oryziterrae	MEESRVEAIEWSFDALYKVKEMPSWFRELLTAFSNEKRLIGHGVFFSLFSGKWLPEQESWLRHLERTAAEFRFDHITEHFGFMTGKDFHHGAPLNIPYTTTTLNIGRDRLKRIYEACRRPVGLENLAFSYSLDEVKRHGTFLEQLLEPVNGFIILDLHNLYCQLRNFDISFDEMIALYPLDRVREIHISGGSWEDSAVAPNRSIRRDTHDDAVPPEVFQLLEMTIAKCPHLKYVVLEQLGNGLVTETSKRCFYNDFLQMEDIVRKNSNESNTPDPFLPLFPLSTGPVVEDMTLYQQQLELSSILESSASYAEAMHVLTQSSLANSDWRIEQWEPYMIETAVKIAQKWKK
WP_168764034.1	Chitinophaga polysaccharea	MEAARVEAIEWAFDILYTAEQVPSWFEELLTAYSRENRLIGHGIFFSLFSGKWLPEQQQWLNHLRKTSRYYHFDHVTEHFGFMTGKDFHHGAPLNIPYTPVTLNIGRDRLRRMQDACECPIGLENLAFSYSLEEVKRHGDFLEKLLEPVNGFIILDLHNLYCQAYNFGLSFEALKGLYPMHLVREIHISGGSWEDSETMPGRIVRRDTHDDAVPEAVFIYLEKAIDFCPSLKYVVMEQLGTGLTTAGRQAAFRNDFHRMDQLIRNKNRTAAPVNTFIPAPLSLPTLAIEDNFLYQQQLELSAILETAGSFDTLMEALQGSSLAHSDWQIESWDPAMIETAARIAQKWKKK
WP_188091977.1	Chitinophaga qingshengii	MEAASVEAIEWSFDTLYAVEEVPAWFEELLTAYSQENRLIGHGVFFSIFSGRWLPEQQQWLHHLKKISQQYRFDHVTEHFGFMTGKDFHQGAPLNIPYTAVTLDIGRDRLKRMQDTCRCPVGLENLAFAYSLEEVKRHGDFLNELLEPVNGFIILDLHNFYCQAHNFELSFDELKSLYPLHRVREIHISGGSWDHSEAMPGKTVRRDTHDNAVPDTVFSYLDKAIDLCPSLKYVVLEQLGTGLTTVDSQVAFRNDFLRMNQLIKNNNNTSLPENSFLPQPLQLPAQVREDESLYQQQLELSHILEAAVSFDAVMCALQASSLAHSDWQIERWDPAMIETAAKIAQKWKHGWT
WP_236015555.1	Chitinophaga solisilvae	MPEIRSAVACNLDANILAACLPLMEASKIEAIEWSFDALYKVKDIPDWFVELLTAFSNENRLIGHGIFFSLFSGRWRQEQEQWLQHLRKTSTHFRFDHITEHFGFMTGKDFHHGAPLNIPYTKSTLNIGVDRLKRIHDACRCPVGLENLAFSYSMDEVKRHGAFLDELLEPVNGFIILDLHNLYCQLHNFDITFGEMIILYPLHKVREIHISGGSWEASPAAPERMVRRDTHDDAVPEEVFQLLQKTIPLCPNLQYVVLEQLGTGLETDTSRTLFRQDFGRMQRIVHEQQPQTGSCPVNSFMPDNLLISAVPAADETLFQQQLELSAILESASSCEAAVHALQHSSLAHSDWLIEQWQPYMIETAVSIAQKWKKI
WP_169190781.1	Chitinophaga sp. Ak27	MEAARVEAIEWAFDTLYTAEQVPSWFEELLTAYSRENRLIGHGIFFSLFSGKWLPEQQQWLNHLRKTSRYYHFDHVTEHFGFMTGKDFHHGAPLNIPYTPVTLNIGRDRLRRMQDACECPIGLENLAFSYSLEEVKRHGDFLEKLLEPVNGFIILDLHNLYCQAYNFGLSFEALKGLYPMHLVREIHISGGSWEDSETMPGRIVRRDTHDDAVPEAVFIYLEKAIDFCPSLKYVVMEQLGTGLTTAGRQAAFRNDFHRMDQLIRNKNRTAAPVNTFIPAPLSLPTLAIEDNFLYQQQLELSAILETAGSFDTLMEALQGSSLAHSDWQIESWDPAMIETAARIAQKWKKK
WP_168873219.1	Chitinophaga varians	MDAARVEAIEWSFDALYAVTKVPVWFEELLTVFSNENRLIGHGVFFSLFSGRWLPEQQQWLNDLRAVSRRYQFDHVTEHFGFMTGRDFHHGAPLNIPYTPTILEIGRDRLKRMQEACSCPVGLENLAFSYSLEEVKKQGDFLDKLLGPVNGFMILDLHNLYCQAHNFNLSFEALISLYPLHRVREIHISGGSWEVSGAAPGKTVRRDTHDDAVPEEVFGYLDNAIDLCPSLKYVVLEQLGAGLDSDSSRAAFGHDFLRMDDLIKQKNETIAPLNSFLPEALQIPAQPPEDESLYRQQLELSQILETAVSFDGLMQALKASSLAHSAWQIESWDPAMIETAAQIAQKWKKR
WP_188100661.1	Chitinophaga varians	MEAAQIEAIEWSFDALYAVEKVPSWFEELLTVFSDENRLIGHGVFFSLFSGRWLPEQQQWLDHLKAVSRRYRFDHVTEHFGFMTGRDFHHGAPLNIPYTPVTLEIGRDRLKRMQDACGCPVGLENLAFSYSLEEVKKHGDFLNELLEPVNGFIILDLHNLYCQTHNFNLTFEELNKLYPLHRVREIHISGGSWEISGATPGKTVRRDTHDDAVPEEVFAYLDKAIDLCPSLKYVVLEQLGTGLDTEDSRIAFGSDFLRMDELIKNKNKTAAPLNNFLSEALPVAAQAREDESLYQQQLELSQILETATSFEALMKALHASSLAHSAWQIESWDPAMIETAAKIAQKWKRKDVIL
WP_207335620.1	Fibrella aquatilis	MAAALPLFEAGEVGAIEWAFDSLYRYDELPGWFADLLTTYGEAGRLVGHGVFFSLFSGQWTPEQAAWLTSLRALSQRFRFDHVTEHFGFMTGANFHQGAPLGVPCTPQTLALGRDRLQRIQDACGCPVGLENLAFAYAPDEVKQHGEFLRQLIEPINGFIILDLHNLYCQLMNFDISAPDLLALYPLDRVREIHISGGSWHDSAADPTRAIRRDTHDDAVPDAVFNLLTYVMDQCPNLRFVVLEQLGTALDTDESRQQFRADFRRMDAIVKAMDTDGNGLVNSFLPPKQASVSNPPLTDAALHAQQRTLATILETATDYQQAQTRLASSELAHTAWQPERWQPAMLETAIAIAQKWRRGIEGKL
WP_085413297.1	Fibrella sp. ES10-3-2-2	MAAIRSTLACNLETNLILAALPLFEAERVAALEWSFDALYAHEQLPDWFTELLTTYSEADRLVGHGVFFSLLSGRWTSEQQAWLIRLRALSREFRFDHITEHFGFMTGADFHKGAPVNVPCTAVTLALGQDRLSRIQDACRCPVGLENLAFAYSLDDVKRQGDFLRQLIEPVNGFIILDLHNLYCQSKNFNLDLTALLDLYPLDRVREIHISGGSWAEVEIEPGRHIRRDTHDDAVPDDIFNWLSTAIDRCPNLGYVVLEQLGAGLTTGSERLAFQADFDRMDVIVQQKNKSIAHPDMQSFLPELPLLINPVPLENHRLYTHQLELSAILETAVDVQQAKRRLAESSLANSEWEVERWEPAMLETAVAIARKWKNGFVGKE
WP_214460436.1	Flavihumibacter fluvii	MWFEDLLAAYSAQNRLIGHGVFFSLFSGRWSDAQQNWLAALRQTSQRYRFDHITEHFGFITGKDFHQGAPMSIPYTPATLAIGQDRLKRIQDACSCPVGLENLAFSYSLDEVKEHGEFLQRLIEPVNGFIILDLHNLYCQINNFDIGFEEIISLYPLDKVREIHISGGSWDEVSEPFPKNVRRDTHDSAVPEPVFDLLKMTIEKCPALKYVVLEQLGSALSTPESQAVFQQDFLILEEIVQEKNKGRWDFFNDAFLPSPLVIPDDIIEDEALYQQQKQLAEILEHCTTAAEAQQQMRQTTLANSAWDIEHWQPYMLETAIRIAQKWKDGFE
WP_246398786.1	Hymenobacter	MSAALPLLAEGRVEALEWSFDALFWAGQIPDWFSELLRTYSASGRLLGHGVFFSLLSGRWSREQQQWLTQLRQLANQLPFAHITEHFGFFTGQNFHSGAPLPVPYTPVTLRLGQDRLSRLYEACQCPVGLENLAFAYALEEVKRHGEFLDKLLEPVNGFLILDLHNLYCQLHNFSISYEDLIDLYPLHRVREIHISGGSWEASALAPGQRVRRDTHDQGVPEEVFQLLQRTMPRCPHLRFVVLEQLGNSLRTEDSRARFRLDFGRMEALVQEHRAGLTASADQAFLPLRPPQPGPALEEPALHEQQQHLSRILETAPSVAEARQLLLASPLLASTDWNIEQWAPHMLETAVRIAQKWK
WP_223847018.1	Hymenobacter montanus	MSAAFPLLEAGEVEALEWSFDALMGEMPEWFSTLLSVYGEQGRLVGHGVYFSLLSGQWTPEQQQWLHDLGQLAARFSFDHVTEHFGFFTGESFHAGAPLPVPCSAATLRLGRDRLRRIQDAGRCPVGLENLALAYSLADVQQQGAFLDQLLEPVNGFLILDLHNLYCQLHNFAIAPETLIDLYPLDRVREIHISGGSWEASARAPRGRVRRDTHDGAVPEEVFQLLERTMPRCPHLKYVVLEQLGTALKTERQRAGFRRDFRRMARLVQQHDRATTRGSDQLFLPLHPSPGGPVVVDEKLHGQQRQLSRILEAAPTLDEAQRLLHGSSLAHTEWHLENWPPYMLETARRIAQKWKR
WP_262904639.1	Hymenobacter lucidus	MSAIACNLDADILSASFPLLEQGRVEALEWSFDALFWAEQVPEWFTDLLRAFADQKRLVGHGVYFSLLSGRWTREQQQWLEQLRELAGRFAFDHITEHFGFFTGQNFHSGAPLPIPYTSAALRLGQDRLRRIQQAAGCPVGLENLAFAYSLDEVQRHGEFLDQLVEPVNGFLILDLHNLFCQLHNFAVPYEELIKLYPLERVREIHISGGSWEKSGQEPGRQVRRDTHDDSVPEEVFELLALTLPRCPNLKYVVLEQLGNALKTEASRTQFRADFGRMETLVGEFRRRAAPRASQLFLPPQPWLAGPVAEDAHLHEQQQQLSRILETAPSFEAARQQLHASSLAHSAWKIEEWAPHMLETALRIAQKWKQ
WP_208419998.1	Paraflavitalea devenefica	MEADILGACIPLWEEARIEAIEWSFDALFNVEEIPAWFRELLTAFSKEKRLIGHGIFFSLFSGKWLPEQQQWLNKLQQTCTEFQFDHITEHFGFMTGKDFHQGAPLNIPYTTSTLAIGRDRLQRIQEACQCPVGLENLAFSYSLDEVKRHGTFLDQLLEPVNGFIILDLHNLWCQLHNFSIPFEEVIDLYPLHRVREIHISGGSWDDSEVEAGRTIRRDTHDNAVPAAVFQLLEMTISRCPHLQYVVLEQLGTGLLTPESKQQFYQDFGRMESIVSEKNLSRTPHSTCSFLPQAPLQKGGVTEDLFLYQQQRELSAILESAPSYEDARRLLAQSSLAHSDWKIENWDPAMVETAIKIAQKWKQGWLT
WP_237487918.1	Hufsiella arboris	MIDAYSKEGRLIGHGVFFSIFSGRWSIQQQEWLDNLKKLSARFKFDHITEHFGFMTGANFHYGAPLAVPFNSKTLTIGRDRLNRIYDACQCPVGLENLAFSYSIDEVKKHGEFLETLVEPVNGFIILDLHNLYCQMHNFSISFDEIIKLYPVDRVREFHISGGSWENSSLSGKRIRRDTHDDAVPDEVYQLLDSALDHCKNVRYVTMEQLGSGLKTEESRSNFYNDFLKMQQIVRNKTSVPNDAENTFFPPKIVPPGDIIEDENLYIEQQQLSYILENSTSYDDAVSRIKSSVLAKSDWGIESWAPHMLETAISIAQKWKKK

Supplementary Table 3. Calculated [M+H]$^+$ masses for peptides characterized in this study. [M+H]$^+$ masses shown are monoisotopic unless otherwise noted.

Peptide	Peptide sequence	Modification	Expected [M+H]+
DybA	GSSHHHHHHKISKPVLQAVAVAVALSTITACADMDIKPEKDKKAKTRTLDPCPACGMG	N/A	6137.12
DybA + DybH	GSSHHHHHHKISKPVLQAVAVAVALSTITACADMDIKPEKDKKAKTRTLDPCPACGMG	-4.03	6133.09
DybA + DybH (LysC digested fragment)	TRTLDPCPACGMG	-4.03	1317.54
ChrA	GSSHHHHHHSQMKIPALVMASLLAVSVSGQTTKPVKKGTKSVKKVKKMDSPKTVKAEPTKVVKRDTILKHGGGCPACGMG	N/A	8442.54
ChrA + ChrH	GSSHHHHHHSQMKIPALVMASLLAVSVSGQTTKPVKKGTKSVKKVKKMDSPKTVKAEPTKVVKRDTILKHGGGCPACGMG	9.98	8452.52
ChsA	MHHHHHHKLSKSLLSAIMIGIAVQTTVVSCSKDEQVKPKKADQANKQSESKPVDNPDSCPACGMG	N/A	7013.45
ChsA + ChsH	MHHHHHHKLSKSLLSAIMIGIAVQTTVVSCSKDEQVKPKKADQANKQSESKPVDNPDSCPACGMG	-4.03	7009.42
HymA	MHHHHHHKLSQAILGAVLVGLTAQTTGCIKKSDPTPKEEQGKSGKKSPEIPYNCPGCGLG	N/A	6351.22

HymA + HymH	MHHHHHHKLSQAILGAVLVGLTAQTTGCIKKSDPT PKEEQGKSGKKSPEIPYNCPGCGLG	-4.03	6347.18
ArlA	GSSHHHHHHNQNLREKATQLLDVASNTQGMKSS SPLPCPACGMMMALPESSPQLVTPATR	N/A	6453.07
ArlA + ArlH	GSSHHHHHHNQNLREKATQLLDVASNTQGMKSS SPLPCPACGMMMALPESSPQLVTPATR	-4.03	6449.04
MelA1 (LysC digested fragment)	LFPQPAPCPACGMG	N/A	1388.62
MelA1 + MelH + MelHc (LysC digested fragment)	LFPQPAPCPACGMG	-4.03	1384.58
GFP-CPACGMG 4aa linker (LysC digested fragment)	GSAGCPACGMG	N/A	910.32, [M+Na]$^+$: 932.30
GFP-CPACGMG 4aa linker + DybH (LysC digested fragment)	GSAGCPACGMG	-4.03	906.29, [M+Na]$^+$: 928.27
GFP-CPACGMG 10aa linker (LysC digested fragment)	GSAGSAAGSGCPACGMG	N/A	1340.50, [M+Na]$^+$: 1362.48
GFP-CPACGMG 10aa linker + DybH (LysC digested fragment)	GSAGSAAGSGCPACGMG	-4.03	1336.47, [M+Na]$^+$: 1358.45
DybA A46P	GSSHHHHHHKISKPVLQAVAVAVALSTITACADMD IKPEKDKKAKTRTLDPCPPCGMG	N/A	6163.13, Average: 6167.18 Average (+3 IAA): 6338.34
DybA A46P + DybH	GSSHHHHHHKISKPVLQAVAVAVALSTITACADMD IKPEKDKKAKTRTLDPCPPCGMG	-4.03	6159.11, Average: 6163.15 Average (+2 IAA): 6277.25
DybA A47ins	GSSHHHHHHKISKPVLQAVAVAVALSTITACADMD IKPEKDKKAKTRTLDPCPAACGMG	N/A	6208.15, Average: 6212.22 Average (+3 IAA): 6383.28
DybA A47ins + DybH	GSSHHHHHHKISKPVLQAVAVAVALSTITACADMD IKPEKDKKAKTRTLDPCPAACGMG	-4.03	6204.13, Average: 6208.19 Average (+2 IAA): 6322.29
DybA His-embed + DybH	MKISKPVLQAVAVAVALSTITACADMHHHHHHDIK PEKDKKAKTRTLDPCPACGMG	-4.03	6033.04
lipo-DybAH	CADMHHHHHHDIKPEKDKKAKTRTLDPCPACGM G	-4.03 (+800.73)	4641.47
lipo-DybAH (observed N-terminal LysC fragment)	CADMHHHHHHDIKPEK	800.73	2772.60
NedA	MKTTSTLMALAGALVLAGCQVEPAQSSSPEVSVQ QKAGEGKCGAASQAGKAAEGKCGEGKCGSKHH HHHH	N/A	7004.36
NedA + NedBC	MKTTSTLMALAGALVLAGCQVEPAQSSSPEVSVQ QKAGEGKCGAASQAGKAAEGKCGEGKCGSKHH HHHH	-8.06	6996.30
lipo-NedABC	CQVEPAQSSSPEVSVQQKAGEGKCGAASQAGKA AEGKCGEGKCGSKHHHHHH	-8.06 (+800.73)	6067.08
lipo-NedABC (N-terminal LysC fragment)	CQVEPAQSSSPEVSVQQK	800.73	2731.74

Supplementary Table 4. Assignment of proton and carbon chemical shifts of LysC-digested DybAH in 90% D_2O and 10% H_2O. Chemical shifts were referenced to D_2O at 4.790 ppm. Br: broad.

Residue number	Residue	NH CO	alpha	beta	gamma	delta	other
1	T	- 168.2	3.90 (C:58.8)	4.17 (C:66.7)	1.32 (C:18.8)		
2	R	8.84 173.2	4.47 (C:53.6)	1.79, 1.85 (C:28.3)	1.65 (C: 24.3)	3.22 (C:40.7) ζC=O:157.0	Side chain NH: 7.20
3	T	8.39 171.4	4.29 (C:59.5)	4.15 (C:66.8)	1.20 (C:18.9)		
4	L	8.38 173.9	4.36 (C:52.4)	1.60 (C:39.8)	1.59 (C:24.2)	0.86 (C: 20.7), 0.92 (C: 22.0)	
5	D	8.46 170.5	4.93 (C:48.5)	2.79, 2.88 (C:36.0)	γC=O 174.7		
6	P (trans)	- 173.6	4.33 (C:61.1)	1.92, 2.28 (C:29.6)	2.01 (C:29.2)	3.76, 3.76 (C: 47.9)	
7	C	8.58 N/A	4.92 (C:53.5)	2.98 (C:33.3)			
8	P (trans)	- 174.4	4.43 (C: 62.5)	2.04, 2.37 (C:29.0)	2.04 (C:24.5)	3.47, 3.79 (C: 47.7)	
9	A	8.52 172.4	4.72 (C:48.2)	1.34 (C:14.9)			
10	C	7.90 157.0	5.02 (br) (C:51.7)	5.80 (br)			
11	G	- 172.0	4.42, 4.15 (C:45.5)				
12	M	- 170.6	4.97 (C:53.5)	2.43 (C:26.2)	2.59 (C:30.0)	2.09 (C: 14.3)	
13	G	8.42 174.4	3.93, 3.88 (C:42.4)				

N/A: not observed.
-: No such proton exists.

Supplementary Table 5. Assignment of proton and carbon chemical shifts of LysC-digested, IAA-alkylated DybAH in DMSO-d6. Chemical shifts were referenced to DMSO-d6 at 2.50 ppm (^{1}H) and 39.5 (^{13}C).

Residue number	Residue	NH CO	alpha	beta	gamma	delta	other
1	T	8.09 (-NH_3^+) 166.6	3.57 (C:58.0)	3.79 (C: 65.9)	1.14 (C:19.6)		Side chain OH: 5.55
2	R	8.66 170.6	4.49 (C:52.0)	1.69, 1.53 (C:29.1)	1.53 (C:24.5)	3.11 (C:40.3)	Side chain NH: 7.44 Cζ: 156.7
3	T	8.01 169.0	4.26 (C:57.7)	3.97 (C:66.2)	1.00 (C:19.1)		Side chain OH: 5.03
4	L	7.84 169.2	4.31 (C:50.7)	1.42 (C:40.7)	1.60 (C:23.8)	0.82 (C: 21.3) 0.86 (C:23.0)	
5	D	8.35 171.8	4.75 (C:47.5)	2.37, 2.73 (C:35.5)			
6	P	- N/A	4.12 (C:60.2)	1.86, 1.81 (C: 24.2) 1.80 (C:28.7)	2.04, 1.74 (C:29.1)	3.66, 3.56 (C:46.6)	
7	C	8.33 N/A	4.82 (C: 51.8)	2.61, 2.96 (C:33.7)			

8	P	-<br>170.7	4.24 (C:61.8)	1.87, 1.82<br>(C:24.2)	2.18, 1.80<br>(C:28.5)	3.65, 3.32<br>(C:46.5)	
9	A	7.99<br>170.9	4.62 (C:46.3)	1.22 (C:15.5)			
10	C	7.34<br>N/A	5.20 (C:57.3)	5.92 (C:64.5)		3.39, 3.16<br>(C:32.8)<br>(IAA: αH)	170.2<br>(IAA:O=C-NH_2)
11	G	-<br>171.0	4.27, 4.04<br>(C:44.9)				
12	M	-<br>167.8	4.60 (C:52.9)	2.37, 2.28<br>(C:26.9)	2.38<br>(C:29.9)	2.02 (C:15.2)	
13	G	8.42<br>170.9	3.82, 3.69<br>(C: 40.8)				

N/A: not observed.
-: No such proton exists.

Supplementary Table 6. Protein sequences of predicted MNIO-modified RiPP-lipoproteins. Sequences are grouped by cluster as shown in the SSN in Fig. 6c.

Cluster	Annotation	Sequence
I	Chryseobasin-like	MKVSKSVLQAVAVAVTVTALATACTDSTIGPNGEKTTKTKTVDPCPACGMG
I	Chryseobasin-like	MKISKSVLQAVAVAVTVTALATACTDSSVKPNGEKTSKTKTMDSCPACGMG
I	Chryseobasin-like	MKISKSVLQAVAVAIVVTTTISACANDDVSPEKEKATKSQKLDPCPACGMG
I	Chryseobasin-like	MKLSKPLLQAIAVAVTVTTISSCTKDKVIDPKAPQGEQQREPYNCPACGMG
I	Chryseobasin-like	MKLSKSLLQAIAVAVTVTTISSCGKGTVVDPKAPNGEQQKKVPYNCPGCGLG
I	Chryseobasin-like	MKLPKAVLGAVLVGLAVQTTSCIKKDEVKPNGEKTGQKGGKTIDSCPACGMG
I	Chryseobasin-like	MKISKSVLQSVAVAVALTTLVSTTACVDNIIKPNSEKKLRQKVDPCPGCGMG
I	Chryseobasin-like	MKLPQALLGAILLGVVAQATTGCKKDAPSPKQEEGSGKGKPVPINCPACGMG
I	Chryseobasin-like	MKLSQAILGAVLVGLTAQTTGCIKKSDPTPKEEQGKSGKKSPEIPYNCPGCGLG
I	Chryseobasin-like	MKLSQAVLGAVLVGLTVQTTGCIKKEDPTPKEEQGKPSKKNPKVPDSCPGCGMG
I	Chryseobasin-like	MKISKPLLQAMALAVAVTTVGSSCKDGLVKPKKEEAKKEQQRKLPDNCPACGMG
I	Chryseobasin-like	MKLSQAVLGAVLVGLTVQTTSCIKKDDPTPKEEQGKPSKKSPKVPDSCPGCGLG
I	Chryseobasin-like	MKIPQAVLGALLVGLAAQTTGCIQKNEPVVKQEQAKPKAPAPEPYSDPCPACGMG
I	Chryseobasin-like	MKLSPALLQAITLGVAVTALSSSCHQNEVNSGRTKEIKAKKEKPVRDNCPVCGMG
I	Chryseobasin-like	MKISQALLGAMLVGLAAPTTSCTKKGDPTPKQEEGKQKKGESTKTPDSCPACGMG
I	Chryseobasin-like	MKLPKTLLSAIVVGIAVQTSACQKDDLPKPTAENGGKDGKEVIKNPANCPACGMG
I	Chryseobasin-like	MKLSKTLLSALLLGITVQTTTSCEKKELPQPNSGKEESSKEQGKEAPVNCPGCGMG
I	Chryseobasin-like	MKIPQAVLGAVLVGLAVQTTGCSSKNDPKPKQTSEQEAKKSGEASKEPINCPACGLG
I	Chryseobasin-like	MKISKKLIGAIVVGIAIQVSTTSCNKKEIKPQKAPNAENGQPVNHDPVLDCPACGMG
I	Chryseobasin-like	MNLPKALLSAILLGITVHTTTSCTDKNELPGPKTEKEKTSKTPEKNDPGNCPGCGMG
I	Chryseobasin-like	MKLSKALLAAMLTGITLQGVQSCTKDKDEPSKEQKEKEKEGENKPKPHPYSCPACGMG
I	Chryseobasin-like	MKLPKSLLSALVIGIAVQAVPACTKTKESPSQKAKKDAKEKETEKTSIPFNCPACGMG
I	Chryseobasin-like	MKLSKSLLSAIMIGIAVQTTVVSCSKDEQVKPKKADQANKQSESKPVDNPDSCPACGMG
I	Chryseobasin-like	MKLSKTLLAALLTGITLQGLESCTKDKDGPSKEEKEKEEEGKGKPKPPTVPYNCPACGQG
I	Chryseobasin-like	MKVSKSLLQAIALGVALGSSATSCMLLDNSDEVKPDTEKAAEDDRCPTDDGDKPWYNCPACGMG
I	Chryseobasin-like	MKLSKSLLGAILVGVTVQTAVTSCNKKSNETFKPTSEAQANPESQANPTNQNPNSVPSECPACGMG
I	Chryseobasin-like	MKIPKTVLQAVAAAVVITTVTACSVSDVNPEGEKVTKNKIVDSCPGCGMG
I	Chryseobasin-like	MKLSKALLQTIAVAVAVTTLSSCDKGKVIDPKKDDTKQQIPYDCPGCGLG
I	Chryseobasin-like	MKISKSVLQAVAVAVALSTITACADIDIKPGKEKKAKTRTMDPCPGCGMG
I	Chryseobasin-like	MKVPASVLKAMAAAVIVASVTACTGEDIGPKNGKKKEEKTLASCPACGMG
I	Chryseobasin-like	MKISKPVLQAVAIAVALSTITACADSDIKPEKEKKTNTKTMGSCPACGMG
I	Chryseobasin-like	MKISKSVIQAVAAAVIVSSVVSCTSGLINPEKEKGSKTKNVDPCPGCGMG
I	Chryseobasin-like	MKIQKNTLQAIAVAVAVTAVTAACAVDNVGPKEKQAKTKVIDNCPACGLG
I	Chryseobasin-like	MKISKSVLQAVAVAVTVAALTTACTDNAVGPNGEKTSKTKTLDNCPACGMG
II	Bufferin-like	MKRTSIKIGLTLAATAAALFATGCTTLCGESQEALVKCSNVNACKGSSECATPSNSCKGRNSCKSSGW<br>VYMTKSECLANGGSVRD
II	Bufferin-like	MNYKRSLLVGAAIAGLSLSACTTTNTSNTGSKVEGECHGINACKGQGACGGKGSSCAGTNACKGKG<br>WLKTAENECVSKGGKFVKG
II	Bufferin-like	MKKSGLFFTAAVGGLMFAATCSPQAEKPKAEAVKGECHGINSCKGQGACGGKDHSCAGQNACKGQ<br>GWLKMTEEECNAQKGTFKPLSMSM
II	Bufferin-like	MGLKTKGTGIALAVSASMLIAGCASNQQQADASSHGSADGMVQCHGVNSCKGTSSCKAEGSSCAGK<br>NACKGKGWLPMSQSECESHGGRMGGFNMN
II	Bufferin-like	MKTASKVTGFTLASAAAALLLAGCSTGGDSAKSASMSKDKKMADVKCAGINSCKGTSACATATSACK<br>GLNSCKGQGWVKASATDCADKGGKVLGQA

II	Bufferin-like	MSAFALSAAALFLGACDKTKSGEGSQAPAEQPPAASSQAKDAKVKCFGVNECAGQSVCAVNKPELGIEHACAGENECKGKGWIKLSSAECDDLGGEIL
II	Bufferin-like	MTNTNKQLMSAFALSAAALFLGACDKSSGGTTDPGDAAGGDANADEVVKCFGINECKGESACNVNKPELGIEHSCAGENDCKGKGWIKVPRSECDAKSGEVL
II	Bufferin-like	MTKNSKQFMSAFALSAAALFLGGCKKTTTEVTSEPTATPGPAVTADEQVKCFGINSCTGESACAVNKPDLGIEHACAGENACEGKGWIKVSRSDCEEKSGEVLGTL
II	Bufferin-like	MTRNNKQFVSAFALSAAALFLGGCKKTEPTAEPTAEPTATAPASNADEDVKCFGINSCSGESVCAVNKPDLGIEHACAGENACEGKGWIKVSRSECETQSGEVLGTL
II	Bufferin-like	MSIETSKARRGAMLALGAAALILAGCAATGAGTNTAANSTVGACVGVNSCKGTSDCKTAGNSCKGQNACKTATNACKGQGSCKSAANSCKGQNECKGQGHLALTGADCDAKGGHLV
II	Bufferin-like	MGLKTKKTGIALAVTASMLIAGCASNQEQASAKGHGHGHGHSHGADNMGQCHGANSCKGKGSCGAAIADSCKGTDSCKAKKDNSCKGKAACKTKKDNSCKGKSGCKAKAEAHSCAGMNSCKGKGWLPMSQSDCDAKGGRFKGAK
II	Bufferin-like	MKALTGAAMAMMVAGLVGCNSTENNSTSAQAAAAETDLVHCYDVNVCGGHNDCKTASNACSGQASCKGTGFVAMPSKACSDVGGNQKDAWVGSVAKADLVHCHDVNVCGGHNDCKTASNACAGHASCKGTGFVSMPAKACADIGGKVKS
II	Bufferin-like	MKKFTGAAMAVMVAGLVGCNSTEADTSAASVANMNSTTDLVHCYDVNVCGGHNDCKTADNACSGQASCKGTGFVAMPSKACGDVGGAIKDKWVGTVAQTDLVHCNDVNVCGGHNDCKTADNACAGQASCKGTGFVSMPAKSCGDIGGSVDS
II	Bufferin-like	MNKNTKNVMSGLAVAFAAAAIAGCSATANTNTTAAAANTTDLVHCAGVNVCKGHNDCGGASNACAGQGSCKGSGFVVMPSKACGDVGGKVQDDWRGEVSKAELVQCTGVNVCKGHNDCSGADNSCAGQASCKGTGFVKMGAKACGDIGGKS
II	Bufferin-like	MKKFTGAAMAVMVAGLVGCNATETQTSSSNTVANNTATTDLVHCYDANVCGGHNDCKTAENACAGQASCKGTGFVAMPAKACADVGGKVKDKWVGTVTKTDLVHCNDVNVCGGHNDCKTANNACAGHASCKGTGFVSMPAKACGDIGGSVDS
II	Bufferin-like	MKALTGAAMAVMVAGLVGCNSTDNTSSNTANANTSGTSTDLVHCYDVNVCGGHNDCKTASNACSGQASCKGTGFVAMPAKACADVGGKVKDKWVGSVAKSDLVKCNDVNICGGHNDCKTANNACGGHASCKGTGFVKMPAKACQDIGGKVDS
II	Bufferin-like	MKQTKLNLSGVAIAMTAASLISGCVGPSKSTHSSTAAAGKTDLAHCYGVNICGGHNDCKTANNSCKGQASCKGQGFVAMPTKACNDVGGKVKDDWRGQVSKADLVKCYDVNLCKGHNDCKTANNACAGQASCKGQGFVLTPQKSCEDIGGKVG
II	Bufferin-like	MITNMKTKLSGAAIAMAAAGLAGCMGANAANSSTAAPTDTVELGHCYGINSCKGHNDCKTANNACKGMGSCKGQGFVTMPTKACTDAGGKVRDDWRDQIAKAELTQCFGVNVCKGHNDCKTADNACAGHGSCKGTGFVLTTAKSCADIGGTTK
II	Bufferin-like	MSQHQRLLRGAGLAVAAAGLAACGHTQTTAQTSSAAAEKTDLIHCYGVNVCKGHNDCKTASNACAGHGSCKAQGFVAMPTKSCEDAGGKASREWVGEIARADLIHCYGVNICKGHNDCKTKENACAGHATCKGTGFIEAPAKSCKDIGGTVGK
II	Bufferin-like	MKALTGAAMAMMVAGLVGCNSTSESGATSGASAANAGAAKTDLVHCYDVNVCGGHNDCKTASNACAGQASCKGTGFVGMPSKACADVGGKQKDDWVGSIAKADLVHCHDVNICGGHNDCKTASNACAAQASCKGTGFVNMPAKACKDIGGKVGS
II	Bufferin-like	MKALTGAAMAMMVAGLVGCNSTSDSGATSNASSANAGGAKTDLVHCYDVNVCGGHNDCKTASNACAGHASCKGTGFVGMPSKACADVGGKRKDDWVGSIAKADLVHCHDVNICGGHNDCKTASNACAAQASCKGTGFVNMPAKACKDIGGKVGS
II	Bufferin-like	MKALTGAAMAVMVAGLVGCNATDSSQSSTASATASSGGAKTDLVHCYDVNICGGHNDCKTASNACAGQASCKGSGFVGMPAKACADVGGKQKDSWVGSVAKADLVHCHDVNICGGHNDCKTANNACAAQASCKGTGFVNMPAKACKDIGGKVGS
II	Bufferin-like	MKALTGAAMAVMVAGLVGCNATDSSQSSSAGATASSGGAKTDLVHCYDVNICGGHNDCKTASNACAGQASCKGSGFVGMPAKACADVGGKQKDDWVGSVAKADLVHCHDVNICGGHNDCKTANNACAAQASCKGTGFVNMPAKACKDIGGKVGS
II	Bufferin-like	MKALTGAAMAMMVAGLVGCNATDSSQSSNASTAASSKGATTDLVHCYDVNICGGHNDCKTANNACAGHASCKGSGFVGMPAKACADVGGKQKDSWVGSIAKADLVHCHDVNICGGHNDCKTANNACAAQASCKGTGFVNMPAKACKDIGGKVGS
II	Bufferin-like	MNTKAQATLTGAALALAMAGLTGCNSTDKSETTQAAMAKGSSDLVHCYGVNACKGHNDCKTATNACGGHASCKGTGFVAMPAKSCGDVGGKVQDDWVGSIAKTDLVHCYSVNVCKGHNDCKTANNACGGHASCKGTGFVAAPAKACADIGGKVGA
II	Bufferin-like	MKKVSQKITGAAIAVAAATLMGCTTTHSGNSASTATAQGGTTDLVHCYSVNQCKGHNDCKTAENACAGHAECKGHGFVAMPSKACDDIGGKIKDSYRGTIAESELVHCYGVNQCKGHNDCKTAENACAGHAECKGHGFVAMPAKSCGDVGGKEGA
II	Bufferin-like	MNTKAQATLTGAALALTMAGLTGCASTEKSETTQAAMAKGSTDLVHCYGVNVCKGHNDCKTATNACGGHASCKGTGFVAMPSKSCADVGGKIQDDWVGSIAKTDLVHCYSVNVCKGHNDCKTANNACGGHASCKGTGFVSAPAKSCADIGGKVGA
II	Bufferin-like	MNSNLKSSFSGAAIAVAAAGLAGCMGSTSASTSSAPMASGNTVELGHCYGVNSCQGHNDCKTADNDCKGQSACKGQGFVTMPLKACTDAGGNVRDDWRDQIAKADLTQCFGINVCKGHNDCKTADNACAGQGSCKGTGFVMTTEKSCDDIGGTTK
II	Bufferin-like	MKPNTKSALQGTAVALAVAGMMGCAQTSSQSGSSSYAAKNTAELGHCYGVNKCSGHNDCKTADNACKGQASCKGHGFVAMPTKSCGDIGGTVKDDWRGKVTTADMAHCYNVNVCGGHNDCKTANNACKGQASCKGQGFVKMSPKACNDVGGKVGK

II	Bufferin-like	MKALTGAAMAMMVAGLVGCNSTSTSNSSAGSATASAAASASSTDLVHCYDVNVCGGHNDCKTASNACAGQASCKGTGFVAMPKKACGDVGGKTKDAWVGEIAKAALVHCHDVNICGGHNDCKTASNACAGQASCKGTGFVSMPAKSCGDIGGKVKS
II	Bufferin-like	MKRTSKTALALAAAAAALFATGCSTMCGDSHEALVKCAGSNACKGSSECATPDNSCKARNSCKATGWSYMTKGDCSAAGGSVVD
III	Oxazolin-like	MNVKTLAALVGTLSLSALATGCASTKSAEAQPAAAEKGAEGSGAATTEGQAAEGAQTQEGAAPAATPEKGSEMSCGSGSCGGGKK
III	Oxazolin-like	MNVKHIAATLAILGSASFAVTGCKKNAEGTEVPAEGEKKADGSCGANKDGSCGANKDGSCSAKKEGEGDVPAAGDAPAEATPPPAN
III	Oxazolin-like	MNVKALAAVVGTLSLTALATGCASSKSAEATPASTDTSTQSPGSQAANPEQGAAGDTTAAPSAPKGEEGKCGEGKCGEGKCGEGKCGGKK
III	Oxazolin-like	MNVKALAAVVGTLSLSALATGCASSKSAEAAPASGDTSAPSPGSQAAAPEQGAAGDTTAAPSAPKGQEGTCGEGKCGEGKCGEGKCGGKKE
III	Oxazolin-like	MQKRNLSLALSVGAILVAGSTLSACNHTPVKSDDGASTAAKPADGKCGAKKDGSCGAKKDGSCGANKDGSCGAKKDATCGAKKDGSCGAKQ
III	Oxazolin-like	MNVKTLAAIVGTLSLGSLAAGCATSSGSAQPGTEKGAQGSCSATGGEASCSGKATQKGGEASCGEGTCSGAPKAATPEKGTHASCGESGCGGGR
III	Oxazolin-like	MANLKDIVATLAILGAGSLALTGCKKEAETANPDEATEATEATDAEATDAEASCAGEGEADAEASCAGEGEADAEASCAGEGEGEADAEAATEAV
III	Oxazolin-like	MANLKDIVATLAILSAGTLTLTGCKKEGGETTNPDEVTADDAAGEGEGEGEGEASCSGEAEGEASCSGEDAAAEGDAEGDAEGEAADAEGEASCSGAA
III	Oxazolin-like	MNVKTLAALVGTLSLGSLAAGCATTHRSAEPGTEKGTQGAGSTRGGEASCGEGTCSGKATQKGGEASCGEGTCGGDQKAAAPEKGSHASCGESGCGGGR
III	Oxazolin-like	MANLKDIVATLAILSAGSLALTGCGDKKDTTNPTDAAGEGGDVAAPAEGEGSCKGEDGEGSCKGDADGEGSCKGDAAEGEGSCKGDAAEGEGSCKGDAAEGEEAAAE
III	Oxazolin-like	MNVKALAAVVGTLSLGALATGCASNKAAEGSKVHAASPGAPEASESSAAPAAEGAAPAAAPEKGAEGSCGAGSCGAGSCSGKK
III	Oxazolin-like	MNAKALAAIVGTLSLASLATGCASTKAAEGQPAAAEKGAEGSCGAAKTEENKPAEGTDAAAPAATPEKGAEHSCGAGGCNGQKK
III	Oxazolin-like	MNVKALAAIVGTLSLGALATGCASNKAAEGSTEGAATTESSAKGTEASCKGGTEAKGTEASCKGATEAKGTNASCGEGTCANKK
III	Oxazolin-like	MNVKTLTALVGTLSLGALATGCATAKPAAAPSEQGSATVNSAAATPEKAAEGTCSGKASPTTEKAAEHGCGAGGCGASGCGGKK
III	Oxazolin-like	MNVKTMAAIVGTLSLGSLATGCATAKPAAATSEAKGTEASCKGGAEATQEKGGEAKCSGKAASTSEKGSEHGCGKDGCGAAKPQ
III	Oxazolin-like	MNLKTLTALVGSLSLGTLATGCATAKTAAAPAEQESTSAHAASAVPAESSTAPAAEGTATPSTEKSSEHSCGSGGCGANGCGGKK
IV	Oxazolins	MEKKSKSILWSSVAAGAMLGAASIFASCSTESKDVTTEVQTPAKDTTGEHTCGTEHTCGKDSAEAKCGEGKAADAKCGEHKTTEGKCGEGKCGADKKK
IV	Oxazolins	MKTTSSLIALAGFLALAGCQGQSNGSAESTPAASVSAPKAEEGKCGEGKCGASHAGEKAGEGKCGEGKCGASHAGEKAGEGKCGEGKCGTSHAGEKAGEGKCGEGKCGSH
IV	Oxazolins	MSNLNRKITGLAAATLLATGLSACKEEKMQAQPLAQGYQNADTAQPAVAQPNQAVVSDTKTAEGKCGEGKCGEAGCSATMGDKSASASCGASTANADKSASAACGASTDKNANASCGGSK
IV	Oxazolins	MQNTISQSSQNKKHLAKNLALSSLLAVSVVGCAESQMGAKPMEQGYQNNQNPPQASQENMSQASMSQENMSQASTTVNEQQSNLFKAPEAKCSEGMCGASHKDSSGGCGASHKDASGGCGASH
IV	Oxazolins	MKTTSSLIALAGLLALAGCQDKNQQPAPTAAAASAAASKAEEGKCGEGKCGAASHAESKAEEGKCGEGKCGAASHAHSKAEEGKCGEGKCGAAAKAGSKAEEGKCGEGKCGAGSKADAKSAEGKCGEGKCGSK
IV	Oxazolins	MTKTSSSLIALAGLLALAGCQGQSNSEPVQATSAASSSTSKAEEGKCGEGKCGAASQAAGKSAEGKCGEGKCGAASQAAGKSAEGKCGEGKCGAASQAADKSAEGKCGEGKCGAASQAAGKSAEGKCGEGKCGSK
IV	Oxazolins	MSNLNRKITGLAVATLLATGLTACKEEKVEAQPMEQGYQNAEPAQPATAEPNTATATKTAEGKCGEGKCGEAGCSATMTDKSAEGKCGEGKCGEGKCGAEAKAGDKSANAGCGANTATATADKSATASCGASTDKNANASCGGNK
IV	Oxazolins	MKTTSTLMALAGALVLAGCQVEPAQSSSPEVSVQQKAGEGKCGAASQAGKAAEGKCGEGKCGSK
IV	Oxazolins	MKISSSLIALAGLLALAGCQQEAVSGKPAVEAASHADKSAEGKCGEGKCGASHSKNAKSAEGKCGEGKCGSK
V	BON-domain containing protein	MKKYYLNGLIGFCLAILVGCQSLTHENFFRVGPSDETITTSVQSAFSNNPDLSAQRIHVETHKGTVVLSGYVKTIRQSDTAGDIAGKISGVKSVQNELIVRK
V	BON-domain containing protein	MKNYYLKGIIGFFFVLLVGCQSLTNENFFRIGHSDASITASVNSAFASHQDLSTQRIHVETQKGTVVLSGYVKTIRQSDTAGDIAGKIPGVKSVQNGLIVRK
V	BON-domain containing protein	MRKHVQIVLLALIGLILVGCSFTNGRPVFTPRLSDDAITTSVHQAFVNNRILSDAPVHVETHQGNVVLSGYVKTIRQSDTAADVAAKVPGVKFVQNNIIVRK
V	BON-domain containing protein	MMRRYIQVVLVGALFVLIASCQMISSPSIFNPRLSDQAITNSVYQAFADSKILSDVPVHVETHQGNVLLSGYVKTIRQSDTAQDVATKVPGVRGVQNNIIVRK
V	BON-domain containing protein	MRRYCCIALISISLIGMAGCQMLTSGNLFAPRFSDDAITSSVNQAMMNNINLAGIPIHVETHQGNVMLSGYVKTIRQSDTAGDVASKVPGVKSVQNNLIVRKW

V	BON-domain containing protein	MRRYGRMVLISLGFFAMTGCQVLTGGNVFSPRLSDDTITTSVSQAMMNNQNLINVPIHVETHQGNVILSGYVKTIRQSDTAGEVASKVHGVKTVQNNLIVRKW
V	BON-domain containing protein	MMRKYCRIALMCISFMAMSGCQMLSGGNIFAPRLSDEAITSSVKQAMMNNNNLMNVPIQVETHQGNVMLSGYVKTIRQSDTAGDVASKVPGVKSVQNNLIVRKW
V	BON-domain containing protein	MKRISERIPVVIMLTLVMLLAGGCAGNGETRSTGQYVDDAALTSKVKTALFRDDDVSGFQVDVDSFKGRVQLSGFVDSEEQKLRAEQVARGIEGVQEVTNNLEVK
V	BON-domain containing protein	MLKYLLSALISVFLVLVSGCQTHTTAGSGLFSPTYPSGMTLAQSVQEALIQSNDPVIAQVRVETNQNTVILSGYVKKIRQSDIAEQIARNVQGVQMVQNNIIVRP
V	BON-domain containing protein	MARENTAALLLAAIMASTVVACSSTPTQQSTGQAIDDGVVTAKVKAKLIEDPVTKAHQINVETFKGAVQLSGFVETDQARTRALQLARDTDGVKSVKDALEIRKGG
V	BON-domain containing protein	MRILLTACALMLLSGCTALMVGGAAAGGYQLGKDERSASQVTRDGATTASIKSRLIADKLVSAFNVNVDTYENRVTLRGTVGSYAARARAAAIANEVDAVVAVDNQLKVVNSR
V	BON-domain containing protein	MNCIRRVFLVLITVTTTIAISGCATAVMTGAAQGGYDPDDGRSARQAPADAEISAAVTRALVHHPQIPAMPITVRTERGRVTLSGKVPDRDTARRAARLAAAVPGVESVRNLLRVEQR
V	BON-domain containing protein	MRFPQLSFAASAGVLAAIGATTLLTACAWGDSSTASGARMLDDSLLSYQVKAALDQDTALSTRQIRIRSNPDGKVTLTGWVDTPEMARRAGEDVKRFVDPAKLDNQLRVLSRMQVLGGGPYIPAGLPPEAPASAPAAR
V	BON-domain containing protein	MRFPQLPFAAPAGALAAIGAAVLLTACAWGDSPNSTGARMTDDSLLAYQVKAALDQDTALNPRQIRIHTTPEGKVTLTGWVDTPEMARRAGDDVKRFVDPAKFDNQLRVLSRSQVLGGGPLIPAGLPPEAPASAPAAR
VI	LPS translocon maturation chaperone LptM	MMRLPLKLAMLATVALLSACGQQGPLYFPKDAPAQNEPVNPETSQATQASTQP
VI	LPS translocon maturation chaperone LptM	MMRLTLKLAALATVALISACGQQGPLYFPEDAPAQNEQVNPETPQDTQESTQP
VI	LPS translocon maturation chaperone LptM	MRTILNASLLMAIVTLSGCGQRGPLYFPEDAPATQGSPADNSQQDTAPDSTKTQ
VI	LPS translocon maturation chaperone LptM	MRMTLKLAVLSAAILLSACGQQGPLYFPEDAPAQNTAPAQTETAHDTQAQDPAQQ
VI	LPS translocon maturation chaperone LptM	MRTTLKLAVLSATVLLSACGQQGPLYFPEDAPAQNAAPAQTEIANDTQAQDPAQQ
VI	LPS translocon maturation chaperone LptM	MMRTTLKLFVLAAVTVLSACGQQGPLYFPEDAPAQNSAPATSDTTQDTQPQAASQP
VI	LPS translocon maturation chaperone LptM	MMRTTLKLVVLAAITLLSACGQQGPLYFPEDAPAQNSAPAETGNTQDTQAQGTDQP
VI	LPS translocon maturation chaperone LptM	MLHSKLSSQTVILIVKSTLALQFTALACTAALLGGCGQQGPLYLPKQPAKPASANTQGKPGVAPPSAPVPSTPPASQQSPVN
VII	Glycine zipper two transmembrane domain-containing protein	MRKSVLLAACFTTLSLLLGGCASSLTGDSYSRDEARRVQTVRMGTIESLRPVKIEGTKTPIGGAAGAVIGGVGGSAIGGGRGSIVTAVIGAVAGGLLGSATEEGLTRTQGVEITVREDDGSTRAYVQAVQENEIFRIGDRVRIMTVDGTSRVTR
VII	Glycine zipper two transmembrane domain-containing protein	MRKSVLLIAAFSTMTVLLGGCTSNLTGDSYSRDEARRVQTVRMGTIVALRPVQIEGTKTPIGAATGAIVGGVGGSAIGGGRGSIVTAVIGAVAGGLLGSAAESGLTKTQGVEITVREDDGSTRAYVQQVEPNQVFRTGERVRIMTVDGTSRVTQ
VII	Glycine zipper two transmembrane domain-containing protein	MRKSVLLVACFTTLSLLLGGCASSLTGDSYSRDEARRVQTVRMGTIESLRPVKIEGTKTPIGGAAGAVIGGVGGSAIGGGRGSIVTAVIGAVAGGLLGSATEEGLTRTQGVEITVREDDGSMRAYVQAVQENEIFRIGDRVRIMTVDGTSRVTR
VII	Glycine zipper two transmembrane domain-containing protein	MRKSVLLVACFTTLSLLLGGCASSLTGDSYSRDEARRVQTVRMGTIESLRPVKIEGTKTPIGGAAGAVIGGVGGSAIGGGRGSIVTAVIGAVAGGLLGSATEEGLTRTQGVEITVREDDGSTRAYVQAVQENEIFRIGDRVRIMTVDGTSRVTR
VII	Glycine zipper two transmembrane domain-containing protein	MRKSVLLVACFTTMSLLLGGCASSLTGDSYSRDEARRVQSVRMGTIESLRPVKIEGTKTPIGGAAGAVIGGVGGSAIGGGRGSIVTAVIGAVAGGLLGSATEEGLTRTQGVEITVREDDGSMRAYVQAVQENEIFRIGDRVRIMTVDGTSRVTR
VII	Glycine zipper two transmembrane domain-containing protein	MRKSVLLVASFSTMAMLLTGCQSNLSGDSYSRDEARRVQTVRMGTIESLRPVKIEGTKTPIGGLAGAAVGGVGGSAIGGGKGSIVAAVIGAVAGGLIGSATEEGLTRTQGVEITVREDDGSMRAYVQQVQENEVFRVGERVRIMSVNGTSRVAH

VII	Glycine zipper two transmembrane domain-containing protein	MRKSVLLVASFSTMAMLLTGCQSSLTGDSYSRDEARRVQTIRMGTIEALRPVKIEGTKTPIGGAAGAVVGGVGGSAIGGGRGSIVAAVIGAVAGGLIGSATEEGLTRTQGVEITVREDDGSMRAYVQQVQENEVFRVGERVRIATVDGTSRVSH
VII	Glycine zipper two transmembrane domain-containing protein	MRKSVLLVASFSTMAMLLTGCQSSLTGDSYSRDEARRVQTIRMGTIEALRPVKIEGTKTPIGSIAGAAVGGVGASSIGQGNGSIVAGIIGAVAGGLIGSATEEGLTRTQGVEITVREDDGSMRAYVQQVQENEVFRVGERVRIASVDGTSRVSH
VII	Glycine zipper two transmembrane domain-containing protein	MRKSVLLVASFTAMATLLGGCASNLTGDSYSRDEARRVQTVRMGTIESLRPVKIEGTKTPIGGAAGAVIGGVGGSAIGGGRGSIVTAVIGAVAGGLLGSATEEGLTRTQGVEITVREDDGSMRAYVQQVQENEVFRIGDRVRIMTVNGTSRVTR
VIII	TssQ family T6SS-associated lipoprotein	MLVSALAAAVLAAGCAQTPPPPVGLLDVTSRPAERALQGGIRAYEDGQYPEAEKQLNLALTTGLVSPRDRAAAHKHLAFIYCTSSRTSDCEAAFRAARQADPAFALNKSEQGHPAWGPVYKRVQP
VIII	TssQ family T6SS-associated lipoprotein	MRWPALALVVLSISGCALPPAPSPGLMDVAERPAEKALLGGMRAYDDGQYVQAEAQLKQALATGLAAPRDRAAARKYLAFIYCTSQRVPACEAGFRAARRDDPAFALTRAEAGHPLWGPVWLKSRD
VIII	TssQ family T6SS-associated lipoprotein	MNRLTAISALFALACAGCATPPAAPPAGGLSDLLERPAERALYEGMRAYDDGQYPAAEKSLRRALGEGLASPRDRANAYKLIAFIACTSERLGECEAAFMAARTVYPAFVLSRSEAGHPLWGPVYRRVAP
VIII	TssQ family T6SS-associated lipoprotein	MRTLLGTTLASLVLATLAGCVTQPKQAPIGLLDVVSRPAEKALQAGLRAYDDAQYGEAEKQLNNALKVGLVSPRDQAEAHKVLAFIYCTSRRPVECEAEFRAAKASDPAFALSKSEQGHPLWGPIYKKIP
VIII	TssQ family T6SS-associated lipoprotein	MTKTLLLAMSATALVLIAGCVQPPPAPPVGLLDVTSRPAERALLAGIRAYEDGQYPESEKQLNAALQSGLVSPKDAAAAQKHLAFIYCTSNRTPQCEGAFREARKADPAFALSRSEQGHPSWGPVYKRMSP
VIII	TssQ family T6SS-associated lipoprotein	MRLPPVATLVSTFAGMLLAAGCAQTPPAPVGLLDVTSRPAERALQGGIRAYEDGQYPEAEKQLNLALTTGLVSPRDRAAAHKQLAFIYCTSNRTSDCEAAFRAARQADPAFALNKSEQGHPVWGPVYKRVQP
IX	Hypothetical protein	MKLVTSVLMGLLLLGANGCAELKEVGTVIGHTTRDATKAIGHASRDAVNSIKEDMSDEE
IX	Hypothetical protein	MLFNKILLGAGLAVVMSGCAELKEAGTAIGHSTRDTTTAIGHASRDAVNSVKEDLSSED
IX	Hypothetical protein	MKRVLLVAGLLIVVAGCAELKQAGTEVGHATRDVTTAIGHATRDTTKAIGHASRDAVNSVKDDLSED
IX	Hypothetical protein	MLKLGKMLILSVLLGASGCAELKQAGTDIGHATRDATTAIGHATRDATKAIGHASRDAVNSVNDDLNSDG
IX	Hypothetical protein	MTQCQKLSFSLFTTLLLLGCAELKQAGKEVGHATRDATTAIGHATRDTTKAIGHASRDAVTNISNDLSKE
IX	Hypothetical protein	MLKLGKMFVLSALVLLGASGCAELKQAGTEIGHATRDAATTIGHATRDTTKAIGHASRDAVNSVKDDLSDGN
IX	Hypothetical protein	MQTLTKSMVLTGMMLFTISGCAELKQAGSDIGHATKDATTAIGHAARDTTKAIGHASRDAVNSVKDDLSSED
IX	Hypothetical protein	MSKFTQALWLTTGILLGVTGCAELKEAGRTVGHATRDATTAIGHATRDTTKAIGHASRDAVNSVKDDLSGDSSD
X	Likely uncharacterized RiPP precursor	MLSKKSIYETLTALGAGAMISACGGSQTPVNATEVPAAQDATPAAEDPATPADVTPDETPATAPPSEPGAAAAPVDPKPAETAQAPATPATAKPAPKPAAKKPAAKKPDASAACGAGTCA
X	Likely uncharacterized RiPP precursor	MNAKSIYETLAALGASAMIAGCAGTQEPVKAAEEPQAEAPAEAAPAEKTEEAAGTEAAGATNAAAAPAEAPKEAAAPAATTTPAPAAAPAPAATPAPAKAAAPAPKPAAKKAAAPKKAGAEAACGEGSCA
X	Likely uncharacterized RiPP precursor	MLSKKSIYETLAALSAGAMVVSGCGGAQAPVNAAEVPAEHETAPAAPTEATSGDDAAGATTPATPAEPSSTTEAPTPAPPTDVQSAATAPQVAPTATPAPAAPAPPSPTTPAKPTAKKPAGKKPGHAGCGAGTCG
X	Likely uncharacterized RiPP precursor	MLSKKSIYETLAALSAGALLMGGCGGAQAPVNATEVPAEQEATPAAPAAPAEPAPAETTSGDAAAGANVPAAPAAPDSAAPPSDAQSAATSPQVAPTATPAATAPTPPTPTAAPKPAAKKAAAKKPGHAGCGQGTCA
X	Likely uncharacterized RiPP precursor	MLSKKSIYETLAALSAGAMVMSGCAGAQAPVNASEVPAEQTAPAPAEPAPAPGPTEAASGDAAAGANVPAAPAAPAPPSDAQSAATSPQVAPTATPAATAAPPAPPTAAPATAAPAKPAAKKTGAAAKKPGHAGCGQGTCG
X	Likely uncharacterized RiPP precursor	MLSKKSIYETLAALGTGAMVMSGCGSAQAPVNAAEVPAAQETPPAAPAEATSGDAAAGEGAPAAPAPAAPAAAAPAPAAPAAPAAPASADTPTPPADAPSALTAPQVAPTATPAPAAPGPAPTSAPAKPAAKKPAAKKPAHAGCGQGTCG
Other	-	MKKILFLFLLAGLFYACKKDSIGTKPILSFKSYSIDSVISSTQQMVLTMNVEDGDGDIEDSIWIGPVFKSNGPNADTFYSVKKMGDIGANKGNKVKAEVQILLRSIEFKLVQNTGVDSIHFVVFVRDNAGHFSDTISTPKIPYNY
Other	-	MKKFLFLFLVVALAASCGKDSYGPVPILSFEGYSVPSIDSNTTTFEAIFRVKDGDGDIDSSIFYTIHYYIPSTLEEQANARMPNIGQNTGKSVNAQVKVTLEAIDFVRWVEHTGTRPDSLWMEVFVQDRAGHISDTIQTTKIPIYKRQ

Other	-	MKKILYLLLAVGLLYACKKDNVGTKPLLFFKSYQPDSITPDTRQFVLTMRVEDGDGDIEDSIGVAMLKDSEQAVNKDTIWQFYKMPKIGQNRGNSIKADVIMPFEEIDFAAAYNPAPGDSAHYIVFLRDNSGNISDTVPTPKFPFRRNR
Other	-	MKKILFLILAAGLFYACKKDKIGTKPLLSFKSYSEDSITPTTKTFVLTMRVEDGDGDIEDSIAVAMFKDSQQATSHDTIWTFYKMPNIGQNRGNKVKADVIMPFQEIDFAAAYNPVPNDSAHYIVFLRDNAGNFSDTIPTPKFPFRRNR
Other	-	MKKFLLLFLFAAFVVLSCNKDNYGPIPTISFESWSLPSIDSNTAVVNAFFRVKDGDGDIDSAIFYRIHYFIANPATDTARFGMKHMAGIGENTGKSVNAQVKLPLEKIDFVRWIEHTEDRPDSMWMEVFIQDRAGNISDTITTKKIPIFKKRAP
Other	-	MKKILMFAAVCSVLAACKKESSGTKPSLSFASYDPSVYFASTAAGVPQNGYNFNVTFNIADADGDINDTLGIRAHYKSKDINNTLPDTTAWIYAQMPDIGANKGHSVKGQVTIALQSIDIIGFNPTSTNDSIWFSAYVRDAAGHFSDTVLTAKTAIIKE
Other	-	MKGKLTVLSVLLSAGLAAGCQGMNQQETASDSKLQQELAGAMDKQDFRLYYTTGRRPVVPGFEQFEFKALEARCGVKAMPGSGDTLRSEADKAARAEAYQYAKAYNLKIYDACLNRL
Other	-	MKDKITAVGALLSIGLTMGCQGMHKQEDADVDDKLQQALTEAMDKQDFRLFYTTGRRPVVPGFEQFAFKDLEARCGVKVMPGTGDTLRSEADKAARSEAYQYAKSYNLKIYDACLNKL
Other	-	MKVLMGFCLLCCLTTGCANDYVQRQTDSPVNDNTTEVSEAVALALKDKDYRLFATTGRRPIFPGLEKMSFSTLKAKCGVKYLSGAGDVLKSEQDKQQRFERYEYAKAYNIEMYAKCKAIK
Other	-	MKGFIGIALFCCLAVGCVNDYAQRQTDSSDPVRDNTTKVSQAVASALRDKDYRLFATTGRRPVFPGLEKLAFSMLKAKCGVKYLSGSGDVLKSEQDKQQRLERYEYAKAYNIEMYAKCKALK
Other	-	MKTYIGLMLSAWLLAGCADGYGEHQNESTTDMTLQKDVSDAIARAVENKDFRLYATTGRRPVFPGLEQLDFDELKVSCGLKYLPSTGDVIKSEQDKQQRLKQYEFAKSYNLKIYAKCKKNAKA
Other	-	MKWIQILACLGLMIGCAHEQPQGSKQSNMNKRSDDLNVDVAIEKALENKDYRLLLTQGRRPVSPGLEHIPIEELKDRCGTKFLTGMGDVIKSTAEKKARVAKYNFAKAYNLKMYAICQKATDKYNKV
Other	-	MYFKLSGSKLLTAAALVIAFTVSSCGLIPSQVESSINAVNVDESSLAIKVYDAVAYFTQAKPVLGSSQFTAEHLAATYYFSSAPHQSLFKGDPNKYAAQYGRKLLTNRSQNYKFLVGINSMLWLRYAT
Other	-	MNKSYFLFPFLLLLSCCGSVQVADPVYKADGRTAIKGYDPVSYFTENKPVAGEEKFQTSWNGAQWKFSSRKNLDAFRKNPENYAPQYGGYCAYAMRDGETYEIDPNAWKIVNGKLYLNYNEKVNGFWSRDIPGNIKKADEQWSKLPKKSQAP
Other	-	MNKSYFLPIVFLLLLDCGSRQLVEPVFKPDGKTAISGYDPVSYFTESKPKEGNPKFSFRWKGADWRFSSQKNLESFKKSPENFAPQYGGYCAYAMRDGEAYETDPKAWKIVSGKLYLNYNEKVHGFWERDVPGNITKADNQWKVLPRKESNP
Other	-	MNKLYFLFPSLLLFIAACSARQAVDPIFKADGLVAIRGYDPVAYFSENKAVAGNEKFQTTWNGAKWKFSSNKNMEAFKKKPESFAPQYGGYCAYAMRDGETYEIDPNAWKIVDGKLYLNYNEKVNGFWSRDIPGNIKKANDQWIKLPKKKEIP
Other	-	MAAGMLIVLAAITAACTTTPRNPNPPDFRYQPRYSVNPACSHGFRPTNALSCSY
Other	-	MSKFRAFMAAGMLIVLAAITAACTTTPRNPNPPDFRYQPRYSVNPACSHGFRPTNALSCSY
Other	-	MPREETHMSKFRAFMAAGMLIVLAAITAACTTTPRNPNPPDFRYQPRYSVNPACSHGFRPTNALSCSY
Other	-	MLIVLAAITAACTTTPRNPNPPDFRYQPRYSVNPACSHGFRPTNALSCSY
Other	-	MHRICVLAVTALILVAGCSGGNVVVAGAGPDDMLKLRAGPGLGFTVIMGLPDGTALTQQDCVTELGQRWCRVTLADAPGVTGYVSADYLSPR
Other	-	MRTHFAALLVLLIATGCTAGSGAVVKGAGPNDLLKLREGPGLEHEIIIGLPDGTRLTRQNCVTTDGKVWCRVFLTDKPSVSGYVSADYLAHR
Other	-	MRRSLGALAGAMLMAACSGSPADVFADRYEVHGVESGDMLKMRGGPGTGFNIITGLPNGTVVRVQECTQTGGTRWCEVVMDRPGGLRGYVSFAYLRAV
Other	-	MRGPVLGALAGAMLMTACGSQPADIFQTRHEVYGVEAPDMLKMRAGPGTGFNIITGLPNGTVLWVHDCTQTGGTRWCEVSMDRAQGLRGYVSNAYLRKL
Other	-	MRAAFLLVPLLLLAACKDEPDFDTRYDKAAKEIEARAKAMDADIAEAEKAAASKGLQAETKPSNPPASSGE
Other	-	MRALLIIPLLLLAACQDEPDFDTRYDKAAKEIDARAKAMDADIAEAEKAAAAASDLPDAAPPSTAAASSGE
Other	-	MRAAVLLVPLLLLSACKDEQRFEDRYDKAAKEIEARAKAMDADIAKSDEAAAAASDLSERPKPSNAPTSSGE
Other	-	MRAIVPILPLLLLAACQDEPDFDTRYDKAVEAIDARAKAMDADIAEAERAAATASDLPDESPSSNAAASSGE
Other	-	MTRIKRLVRPDTQALNWKAAIPVLGLAATLLSACASTAPAPEEIKERAVVDFSTCAKPVWPAESLKNENTGAVTLAFLIGTDGKVKDSSVKKSSGFVPLDEAARVGIEKCSFKPATVNGKPVEDWMLMQYVWMLK
Other	-	MSLSRAALAGLALAAGLTGCGKSSQPPAAPTVPPTELAALKTPPPEYAPELACAGVGGTSVLRVVIGIEGKPTDVSVTQSSGQPVLDEAAMKRVREWQFKPATRNGQAVPQTIQVPVAFKPPVPQPDECFAIEERARRGG
Other	-	MSMSRAALAGLTLAASLTSCGKPSQPPAAPRVAPTELAALKTPPPEYAPELACAGVGGTSVLRVVIGTEGKPTDVSVTQSSGQPVLDEAALKRVREWQFKPATRNGQAVPQTIQVPVAFKPPVPRPDECFAIEERARRGG
Other	-	MRPMYAVAILFLLAGCSSVPAEKTRAIPAERLLGYQQPVSGGGRLEVHRDYGYLGGGCYVAFLIDRQVAARIGVGEEASFQVPAGEHVVGIGIDTQDDTLCGKGLLNRELRTRIAADGNARFRIVSEASSGFDIRAE
Other	-	MRLISAVLPLLLLAGCSSFRADPEDVRPVPADRLLAFQEAREGGGQIVVNRDLGMMGGGCYVAIEVDRQVAARIGVGEVASFQVPAGTRVVGITLDKQDDTLCSKGRLLRELAVPVKVGESHAFRIVSQNKGGFDILPEQPRP

Other	-	MRSILAVLPLVLLAGCSSFRPDAEHITQVPDDRLLAYQEAREGGGQLVVNRDMGLMGGGCYVAIEVDRKLAARIGMAEVASFNVPAGTRVLGLTIDPLDDTLCGMGRLHKEVAVKVAPGSVQYFHIVSENRGGFDIRPDAQPPKLQ
Other	-	MNIFRTKLCVSFGLLTTSVLFSGCNAIAPVKASPATAATPAKASDVTASNTPVKSIEEIANEAMQSKGQLSREQIDALIRANAKCKPNDPRYNS
Other	-	MHTSPQLTVIVSILLATSLITGCTETLPSKATEPQSVQDIAKEAMNSKGKLSKEQIDALIRANAACRPDDK
Other	-	MNKAHPHAATAASLFLTASLFAACSEAPPTKEADSKSVQEIAKEAMTSKGKLSKEQINALIKANAACRPGDQH
Other	-	MKLVKGILFALSVVAMSACGTQAEMSAAQEGEAALSQQESRQEVGTLAALDCSVSIQCSNGTTRSCSGSSGACSASASGSGSVTCNGVTSSCALTLCSCRADGCCNNTCAADPDCGLSNCPQGAACSSNTQCGSNGRCVSGQCMCLIEP
Other	-	MNQERVMKLVKGILFALSVVAMSACGTQAEMSAAQEGEAALSQQESRQEVGTLAALDCSVSIQCSNGTTRSCSGSSGACSASASGSGSVTCNGVTSSCALTLCSCRADGCCNNTCAADPDCGLSNCPQGAACSSNTQCGSNGRCVSGQCMCLIEP
Other	-	MKKFTLTIIMGVFGLTACNSFANMPIKDNTTQAQVQTPITPKAKGVWIDVRTPEEYQAGHLTDSINVPVDDITKRIFAIEPNKNSPINLYCRSGRRAEVARTQLLKLGYTNVTNHGGYEDLKQQGYR
Other	-	MKKLTLAIAISVLGLTACNSFANTSSKQNNAKSQTQTQSQIKPKAKGVWIDVRSPEEYQAGHLSNSVNIPVGDIASKISAIEPNKDNPINLYCRSGRRAEAARTELLKLGYTNVTNHGGYEDLKQQGYR
Other	-	MKYIAPLLAALALSACSSRPVTSGISVEQHKNSFSDQLRVDNLNLAKKLAITDVKTRQTNQLTDVVVTLSSNYKKSQYLQYQFNWFDKDGFIIKGNHSPWQALTLFGFAKTQLPGLAPSPDAVTFSLAVREVSTKSQEFKD
Other	-	MKYLVPLLAVSLLTACAGRPTTSGIAVENASAQYQQQLKVNNPQLGKRLVISDVKTRQTNQLTDVVVTLSSQYKKSQYLQYQFTWFDGDGFVIKGNHSPWQALTLFGFANIQLPGLAPTQEAVTFSLAVREVSTQAQEFKE
Other	-	MKTFTFTKNIFFAALTSLVMLLTSMGCSDNEDKTATATSEHNPFDHPHDFPVTDIQKHKFEHEFAKQCVQNELRNSVNKELDKERYTEPCMCIATFMMKDLTAQEAKKFITEHENPRSLQIKFDSAAYHCLQQKAQAIKGPQLFGKR
Other	-	MKTPTFTKNFFFAALTSMVIALAVTGCSDDEQKTAAAKPAAANVVHNPFDHSHDGEVTDIQKHKFEHDFASQCVDRELKNSTNKEFDKVRYATPCMCIAKFLMKDLTAEEAEKFIGEHKNAQSLVIKYENAAYHCLQQNAHPKGPDFSRAPQQAN
Other	-	MNMQWYRLGLSASLALVLAACASSGGSSNGNWQNIGTTSNGNIKVAVDKSSIKSNGQLVTFRDRKVISKLSEEKFANTPAYKTALADWEIHCGNKTYRLTALQLLNERGQTVTNQRYTATNLRPMSVMSGSITEKQYELVCGKKL
Other	-	MIKETLMRPIFLSFVLFPILITACSTPDKSARWENIGTISNGNIHTYINKDSVRKNGNLMIFQDKKVVTNLKQERFANTPAYKTAIAEWEIHCNNKTYRLSSLQLFDTKNTEISTQNYTASSLRPMSILSGTLTEKQYETVCGKKL
Other	-	MGSKSIIILLMAAGIGLGACSQGNTQQEVSTADNVSRATPTAKAATATPKTAEDVVINGNNTVQTQAVSGQNIEVTGDSNTATFTGHSQEFNLTGSDNVVVLENVKTIEVTGDNNTVTWRGSTPTVTNLGQHNVIERAK
Other	-	MIRKTAMMLLLAVGTGLGACSSGETHSELSGSDSIPSATGEASRASHAAQQVESATSASSAEDIVINGSNTVQTLALQRQNIELTGDDNTATFTGQGRECSVTGSNNVVALEDVKAIEVTGDNNTVTWRGGTPTVTNLGQHNVIERAK
Other	-	MLKISLAAAVLLTVSGCATQNDWGAVGGSKSDAVVRLSYELGALDKHTPDESDGLKLAKLRCGYWGFSGAKPFDVETKTCADEQCRSYTITKEYQCVD
Other	-	MKKRTKLLFATCVMLVVSGCATRSDWEAVSGSKSDGVVTLRYELVASKAYLPDEPEAIALAKQRCGYWGFSSAKAFDTETQHCSDEECRTYIITKDYQCIN
Other	-	MFLVGCNGAAQSVRHATAQQERCVDVPRSDVAPGARNEAFHSARAGVEAVPVAATLGAASPTAPLTPPSSPPLEAQSPQQPIVCSCSA
Other	-	MSRAWAVLLCMFLVGCNGAAQSVRHATAQQERCVDVPRSDVAPGARNEAFHSARAGVEAVPVAATLGAASPTAPLTPPSSPPLEAQSPQQPIVCSCSA
Other	-	MKKIRQTLKQFGLVLILGTALSFSACSTGTKDGDTNVEDSGAKDKDPENMNNTETEVSGRPDSLDMDKNKTYQKVDPDSGARDADNDGKVDQ
Other	-	MKKILLSINRFSLVLLLGAALQLTACSTGTKEGDTNVEDGGAKDKNPEKLNTNEPENSAAADSIRNSDDPDANKTYQKVDPDNGTRDADNDGQADQ
Other	-	MRSFYATLAISCSALTACSTTPPCEDILEVKRQEQECKRLSQVINNPKNPQQALTARQRFEAECENLRYYRDDYDTICKGSNTPIGNVETKRKDP
Other	-	MKFKYGIAPALLALIVGCSSKSPCEDVLEVKRQELECKRLQAASNSKNLQQAGVAKSRFKAECENLRYYRDDYDTICKGEQKPIGEPQTAPAVKQD
Other	-	MYYCRWLVLVALLGGCKDSTAPATPVEIPEQRVVATVYVVNASSGSANCYRTPDSNAPSVATLRNGQLVDLVAEQEGIIQRGTQFWLHVYPRLSHRPSCYLNVDSLVPVS
Other	-	MRGLFYFFTTLITILLTACTDNGNLLNIADISSRPIATVYKVNTAAIDYAPIECRRSPAKNAEVMIRLYNGSLVDLVALEEGMLKRDDRYWLHVYPPLSHRPSCYLDVRYLVPHA
Other	-	MIMKKSGLVIAATAAAIFLSGCATGARNIPENTPLCEASCPPPCVTPCNTCKCMSQCKQVCPPKYKKHRHRRCRTAENATNQMNG
Other	-	MKKTGLVIAAAAAAMILSGCATGARNIPENSPICETPCAAPCNTCKCMSSCKSACPTKKHKRHKKHRRCSAENTQTQMSA
Other	-	MHAIRIRTMIATASLVSLGLLSACSKEAEPSANASGAMSATENSGGMADGAAMTSDNSMAADGNSVAAGNAMGDSMQPSDSMQKNSH

Other	-	MPVLRNRLMVVVFAATGLVLLSGCNSTSGGSSNNEAASGPSDNGGAMADGAAMMSSNGSMAADEN AMSAGSENGGSMQSTDGMQKNSQ
Other	-	MKKYNMMMAAAVASLTLAACSKPAEAPKADATAKADAKTAADPAKSEKCYGIAMAGKNDCANKTGTH SCAGQAKTDNDPNEWKYVDKGTCAGLGGKTA
Other	-	MSRHPLPLPGFAAALAGAAMLAACGSGGKVVQEPTTEANGKGTEIATREKCFGVALKGHNDCKAGP GTTCAGTATVDYQGNAWKYVDTGSCEASGGSLIERAGNTPPAAQKG
Other	-	MRTLLALAFVALTAACGGGSDEAADPPQLRQGSVQVMSSERVLVDITVSAPIKLGKNELAVDFPSRPN TELYGVSALMPAHGHGSPAPTIERTDEGFLVHDVVLYMSGRWELHFQIRVDGRDDEAVVVVDVP
Other	-	MRLPARLANVLLSVLLACACSPGSEQGTSPDAGRDTASGLVRLEAGFEGTLQLRGNMLRIHVTDTAGT PVEATRVSVSLWMPGHGHGAPAPAVTREARGDYLATVDFTMPGTWTVTIQVDTEGRSDTLELSVEAP
Other	-	MATKMGKTLAFTAISGLVAGLAACGGSAPPAETPEAPAAADAPAAEGGEKAGCSGAKEGEEKAGCSG AKDGEKGGCSAKNGCSGAK
Other	-	MATKMGKTLAFTAISGLMAGLAGCGGATPPADAAAPAPEAPAAEGAEKASCSGTKEGGEKSGCKAG SCGGKKEGAEPAAEGEKASCSGAK
Other	-	MNRTIQRARFALAGATVAIVAGCGGGGNDDASTPPQQVPASASASVDGFISYLKSLLGSDADRSEPAD VGAFTPPLDETGEPQKVE
Other	-	MNKLQLALIVVSSAVITACGGSDDEVMGTPPVVVVPPVTAEVPPSASATEAGLFAYLDALFKASADGLE PVDLSTFNPALSDTTEPMAIG
Other	-	MRNLAIASVTALALGLAACSAETEQKADAAAEAAGDDIEANANAAGEVIENAADDVEASVDEAAKDLDA EADKAGDKIEKEWDEAKAEAKEEAQ
Other	-	MRTFAIVSLAALALGVSACSAETENKTDAAAEAAGDDIEANVDAAGEAIDDTMDDVEASVDQAAKDLD AEADKAGDKIEKEWDDAKAEVRKETD
Other	-	MNPLKSNFYGIIIGLITALLMSCNESKSTTKENTKEYKRALQKLKNTDSIKPEPLDTSSSEEMNALKKQLK EDQYGEWPIPNTVK
Other	-	MKFKLISVGAAVLVLGGCASLMKETVSKRAPFDLNCDAGSITIQQLGYRTYGVSGCDKRATYVLQGPC SGPGSQCLAVMNSNVDEG
Other	-	MKKTFIAIAAAATALSFSSCQNPYGPGATNTQRDATTGALIGGIAGAVIGNQSGRSLEGAAVGAALGGA GGAAVGSTKDKQQNYYR
Other	-	MNSKLKTSLVVAASALLGLSACGGGSAQSSESTGETASSGSETTTETTTTESTGDATGTQASCGAGS CGGAAGESTGEAPAEGSGT
Other	-	MLKKVLVVSALALALGGCETARQDRMAGGALIGGGGGALIGGLATNSVGGAVAGGLVGAAAGAIIADA TRPGRCYYHDRYGRRHYVRCR
Other	-	MKLFTRSLFATPLIAAATLGLSACNSPAPADNGTTNDLKLVNEEAPLDANLSTDGELSGNAGSLDAVPT GNETALGNAADGNASVGNTTAH
Other	-	MKPVLFVSLILTLATSLTACDNSSNTDKGTEVSAQLKETGAKVSEPAENFSATVATEAEQVSQSAQEAT SDTTEIIEDNLESIQENVENNP
Other	-	MKRIVIVCSLLFILIITGCNNLSTPKISEDEAQSILLEEHTKHIGKVKIISVSHKGEEYVVQWENKENCKNG TDYVHDQTGEITKGEVSIC
Other	-	MHHLKLFCLAFLFTFLVGCSNERIEISNDVKLFASIPDGAREFEVEVLIIIPKGEVLKVNSRDYMKDFMVI GVTYRDVEGFVIFDSRKMQLIQN
Other	-	MKRHSASLAVAGTIVAVLSGCAAPQESLPLPSPGPQACNAAASQSLIGSQVGAVSFAADANVRVVCTT CPTTRDYRPDRLNVRFDQATGRIEKVDCG
Other	-	MKKILIVVVSSYLVLTSCSKATNKNAIVVRDCTGTYLQIDGKDYQVCNLEKVAAFQDRTAVTATYKKLAE CNGSAKDAIVCMMLHSNEGWIEVTDIK
Other	-	MKKTSSLIALAGALALAACQDSASQNSAAPAAQQQPAAAAKAGEGKCGEGKCGGAASAAAGKAGEG KCGEGKCGGASAAASKAGEGKCGEGKCGGEKK
Other	-	MKKVAVGGFAVLLSACASMAVTDQRLEQNTAFALGLDVGDFTIANRMDEGLKTTYSVKTKAGKQYNC YVMGTIGITGKNVSDAMCNEKGKPAVNPLTGK
Other	-	MKTAKFLAVIILSIFILGCGNSDPGPLAGTWQMSGLMPMTVQFRKGETETMGIIEKVSYKISGNDVLVTY EDGIMKGTTMRYTIADKDTVKTELGLLQRVK
Other	-	MNKISIAALAGALSLAACGEAQKPSQPEAPKTTEAPAATSAPAKAAEGKCGEGKCGGASAPASGAKAA EGKCGEGKCGGASAPAKSAEGKCGEGKCGGAASASAK
Other	-	MLTMMTMKTRSMQPSRRTAAAGLLLALTLAAGCGGDPHRGDGDGGSNGGSNGGGDNGSLPDRVST AAEFLAYVQQVIARFSADTEPLDVDRLGAPTADRDEAADV
Other	-	MKPVITGLTAIALFGSLAACGGTDQPAENLTANDVNAMMAPPELPAVNDIMADPVTEAEPAAETQTPA APAPAAKPTPTKPVPAAPKPKPAEPAEDPHAGHDMNNM
Other	-	MRSVGTTTSRGARRALHRGSAAGALALLLLAGCAGDAGSAPGPEQLGVLFSEHAAVVLGGAARFARD AGGHGFVARTPGGFEASLPGHGEHAIALRLPGGARAHGA
Other	-	MKSAHVILFITTTLLIGCNNFLPENYVKTNINNSHAINAIDHKRINTTDCKDADDWYLDGYRVGKSFSTQK KEMLDQRLGFCHLTSKKLPKKFRTNWEKGFSVGSRG
Other	-	MKTSALFAAGLAALFASACTSTPKVDVRQPGDRNMSCAQLEAEMEALDDIQEEAENNQGVNTANVAA VVFFWPAAVGNYMEADRAMDLAEERQDHLMDIYTEKNCDG
Other	-	MKLRYLLLVGLAVPLTACSVHVGGHRPPPYYAPPPHVEWRWDPGIDAYVVIGWPHLYYRDHIYYRWH DNHWYSSGRHDGPWAKGPKGVPPGLNKKYGKPGRGRGGGGY
Other	-	MKIKVLSAVMLSVLLSGCAGQMAVSNATMKFNMDAVDNRYARGGLTILMAPVYAVTTVADYGLFNPIE FWTGENILTDKKSIYDMKGKNYIEINDDLDESLKTAPIKLD
Other	-	MSLNKGKYLATATTVLFCMLLSGCASPPEHPQLVEADLLFAALQSKSESITLVPQETQAAFVPLAQAYIL SNRNRSDPRIEELSILAKKRIALAEQLISEKLALGCNGSC

Other	-	MYLRYLALAGVLVMATGCVEERVVHERRGPPPRAEHVEVIAPQAPPERIIEEEPAPRPGYIWSRGYWR WNGNRYVPVHGHWEAVRPGYRYQHPYWENRGDGWHYHVGVWVN
Other	-	MSKSNSLKGIVFVLFSFLMLGCTSSYESVTQVDDTKAFILLTGNFENASLQINTNSPITLTDSIETFMLDG KKVAKFEVTAGTNTVKVFKNGALVVNRKLYVTNGNSFEVNVQ
Other	-	MRKGLVFIAIAAFVYACGGSSAPAESNSSAGAKAVAAVAKPDGEKIYKQYCVTCHGLYGDMGASGAY NLQESALTLEERINVITNGRKAMTAFEALLDEKEIKAVAKYTMKLGE
Other	-	MKIKGLIIIGAVLLLSACNSTQGGLYWGSYSDTLHNYKQEPGDTTRQRHVKTLNDIIKTSDKRGTRVPP GVLIELAVMEIESGSSENADALLNREMSLYPESRTLVLELKKRNGA
Other	-	MRRILLFLVITGLLTACDNSPKPPSERVTAALPADPALARVYDTSCKLCHANPASGAPLRGDRAAWSP RVARGADTLLDHSINGYNGMPPMGLCMQCSEEQFLALISFMAGVELQ
Other	-	MILNNLKVIFIFVTCLTLSACSLFDIQQLDDKTKIVKIYDAFEHIEQCQFINELVGSEGTWYSYLFVSNKDL TLGSINDLKNQANAMGANAVHIQYSLDFNTSVTFFAQAYDCAQ
Other	-	MTLLQVINKYSGITIALALLLSCNHVSEQKTDADSTARVQTFKTKDGWAYSIFLGKKEFIRQLYIPCIQGQ IPFETDSQALKAGTLVLNKLKDHQIPSLNLKELEENHLLPAKDVNNNNK
Other	-	MRHHIAATAAVALAVGVALTGCTTEQRRDLLGDVAAEGLRVAAEDAFASAGFPIEDQLDCQVEDVGES EIAADCTGTTQQGADVTFTGSYDTGDTDFTDGVQGTFTGSVEGTEVFSSDCLGCGG
Other	-	MAQSIAKMLKALLVSGLLFLTSAVTACGYAPQMAWADGLPVTTMIGVTSSGTSAWIALSGLDVSQDFD PNKQERIQGTTDMMEESMLVPGGIPSVSKNVEQSLERASGLITGETQEQMMKQSSD
Other	-	MGHGVIRTLVAGGALAASAAALLAGCGGGGNSAATCGDAKKAYERYIGGVRAVSAADAAQWRQPTE QLAANLSGLADKASDDRLKSALKDQADKLRAAAGTVATGDVEQLNATLAAAPTALGTACG
Other	-	MHTRSDSPRRGLSLFFTGLLFTGLALVSIAACSSQQPATQGTSMAAGERRENDCSTGPLLECPEGQL DGCANGTTTEHRCVEAGRGRCLDIRAALVKCLDGEILTHDGCPEPAYMQRCAPRSAQLP
Other	-	MKTSYFLKFIPISFFTLAAVICLLSSSCSPAYEEIEVVYQATDGQRITAVYHNPTNDESEFSVTLKFPSGQ SVTLNQGAAASGVRYTDDKTLVWWTKGGGAIMMKPDGKNDWEITGTYTEIPMHHNR
Other	-	MTMLSGKYTLLGSSTLLLTGCVFVPHTEVSFDEYCGVYKESTSIDMAPRAASNLVFHELELVGGLALAL SNGVVKAQQHQAYKEACLANGIDIDAEAKLSADSVRSQVKQSHYCASQASDLCEIERRR
Other	-	MRLRQHVRRSVTAAFVAAPFAVLVLSGCETLDQAAATVDKAGVCVDALQAANFTPDVNNPDISAQNA ADAAQKLSDLAGQTTDVTLQDALEAMSSTISQMNVGDLNPQAIADWTQQKADLYQSLSSACR
Other	-	MIAGTAKRAAAAAALTIVAAPALSACGGSDLDGAYYDKSGRITIDGGSVTYHTLGCESTGKSAVIINDKA KKTGELNDAGDQVIWSGGGGTKPITVSKNGDTIDIDGKRYAVMDEKEAMDGYKHMCGQN
Other	-	MANRLLITVFILLFMQGCKPTQQQTVFYGQPYVYVSMFELLVHTERFNWRDFIIRGYLADGYLYLSPEQ AKHRDTSYAIAIHPDLSSPAINFSDFNEKWVFINGRLLFEYDSKAYAGYSTLVVREISSRD
Other	-	MFRTLLMLCVCIVLTACSGTPSDTLIEESVAQQKTVSNMIRVVSAEKLNGWKDQEFYVADVRYELEFLT DYKTFSESLKDETPDSLVGSFFSGFGLLALSMQYGKFEKGQKVTERAEFRFRDTENGWQLAD
Other	-	MNAYLLKAIALTFVIALTSACTTTEKYVSTQDQLQQQLNTLATLQRKITTANTEDERNNLLMEQQRAIQQ SINTLEQIQQSKRDANIECIEREKSVPLGDETCHEVESLDDTRITMMVALMQQLLLRTQGH
Other	-	MRGFGLRLAGLLTAFVMAGCAGNQNGEGDTPPQVLQVTIQNDGAITQQPRINLVPEGGGAMTILVGR LTTLGVETLTVRRPDLGGTYRLQAQATGGYTVNSPIFSTRGNERLFWDLRRNVVSVRSLTDDSAQ
Other	-	MKRFFLLSSVLALASCASFINMHRMHAAQSKLQATRTLQQSGQWDAALEMAERMHSSVAKTIQSAPT QKSPGGKVDLRPLLTGWESGPFAALKTALQKHDAKASTAAFSSLRQQCMNCHTVIGKADLRLKEIP
Other	-	MLALSVFLAACATSPKATTYDQLGGASGIEGIVDALLEKIVEDERINFQFADADIVRLRSMLIEQFCAESD GPCTYSGLSMQESHAGRNIDDAQFNALVEDLIEVMTVRKVPVGTQNRLLKRLAPMHGDIVEP
Other	-	MRYQRLPALGAALFALGLLSGCFEAKETAPEQRHSSLSNFQQFIQKTGGLKSLNTFQKRRPEINSEESI GVIYDGARTQGEVMRFRDKQLAKEMLPALKAVRQRLGESENCTLRGYFIACGEEKFVDVFKKWE
Other	-	MNLRWLAVSGLLLLPLAGCAIANDDAAASEVSFSSIAGTSTAPSAAPIDVVDLAAPGTICGSVAAAQDD APVVIRSGSANCVRVLRAARAYSSAAAHADGQAVTVETGGWRCTATVSRGPATCRAGDASFSVG
Other	-	MKKRNLSIGGFALALLLAGCGTPYEQCVRQVSKESRVLKSAIAESRLRISRGYAIHRQTVSYRVPSICH RHDHKMHRPAPYPCGRTEYTTIETPVPIDVAAERRKLASYQKQLVQAEAATKRGIRQCEAQYPKEG
Other	-	MASLRPRTLFCRLPLALLLCAALGACMSERPPIGLPSAQSIQFDGVHALGPNCASIALPSTIGDPDMVE HPSIPFGCATYSNLAAQLARPADIVQPTPYGGTNGVVAERALSRYNAMPEPHAAPDAAPATTNVGH
Other	-	MKRFILVAMLLTVAACERKQETAEVPPSTVPPETTQMMPPVTIAPAPTVPPATTPETAPPTTQPSSGSS APAGSSGSEGAMGSSGAAGAGEYTVSAGDTLSGIAREHNVTSGDLANWNNIQNPNRIHKGQTLRLTA P
Other	-	MKTFYAILLTFIVALAGCNGNKVNLSSLKPAATRTVGKLTIVLLNKSGELMQGQNEFVIQFKDDQGQPA DVGDVQLGSNMSMPGMSPMSGDAEITPAGQTGVYKVTSNFAMSGAWHFTLSWSGPYGQGHTTFNN NVR
Other	-	MTGPSRLAALALVTAAAGLAACQREPAGGPLIATVDNSAAIALLQSVNMQAQTCWMRSKDRDFAAYR LIPELDTRVGKPRILLVQARAAQGLPQLVIEAHGNPVQLTTYGPLTGKPLSGRINTDVTRWATGGTGCK A
Other	-	MTISSARLSLHRSLPYIGIVCLFLAGGCATSPSERQGQRSDIEIGTTTKADVLKRYGAPDLVQMLPDGEI ATYRPSASPQAKPTVSVPTVQAGPAGMMTTQNQTVEPGLGKNTRSSNRLQTELQIRYDQQGVVRELI Q
Other	-	MKKTVCLLFVFFLGCAGRPDSTAVRISLVNNNRSIRFKGLDPAIMGEVSRNASPAVWENLIPVYRMPAD TDLKDYQPIQHGVYQVKDSVIVFTPDTPFVKGHTYFLRYFRFEGGTKPWDFIGGKKKLGSVPHQDLVV RD
Other	-	MRAKPAVAAVVLTVSLTLAGCGAGSSSDSAGKARGVERQADGARGEKGGEDGGAAVAATGAPGAE GKAADGRNPAVVGAQVIRTAELTVEVRDARKALGTARTAAEKAGGLVRNETTERVEDDEVTSTLVLRV PQDAYDA

Other	-	MRRMMLLLAASVVAACQAPMPAANPQMAWVDFSTPFPNDRLLLAERLDKQRLSDGRFFEVSPGRHELIVRFDYEVTGGGGMGMMGGPTVRVCYLTIHYEHFEAGQRYVLEGRSMAMTPEARLYNAKREIVAEASDYYCLM
Other	-	MSRVITKSVAAAALCVTLSACGSFFSDAQSRHETPTLPPGTPNPIALAKLDGLWTGTGKADGLTVRIARAGSSTPRALYGFRGEQPIEPQCSAADATLNCTLPAGLTTTYTANADGSVDFTAKGPNPGDELIAAKLQRAQ
Other	-	MMISKSWAAVTGRGGLAFGLLAILALAGCATPQQRAAEKDDNLAAAGFVIRPANTPQRQAMLRSLPPNRIVQRAHGNTVSFVYADPLVCNCLYIGTQQAYDAYRRYMQQKQLADEQQVTAETWSDAGWDWGPWGPWPWGP
Other	-	MTPRWIATLALPLFAGLSACAMPPGPGGAPSLVGTEWRLEDLGGAGVLDRVPATLAFPEAGRVAGNGSCNRFFGSYTLVQDRIAFGQMGMTRMACVGAVGEQENRYMAALQKAQRVQVQGSTLTLSVEGMDKPLRFVRTKP
Other	-	MNNMIKALAASLTVALALAACATPVQRTASGEIPPARIYIKSMTEQAAGLSQVELSRNGGLLNNELLELAINDVVLAQIAGGEHLSIWLKPGSYDFSVKRINTLTEGSAPTQHKLTLQVNQSGAYKIQISSELRGLSIQLMK
Other	-	MTKGNIYLILAAAGLALVGCNKAESPSEVQHDVTNAQAEAQRDVTDAQSDARESMADAQKDVADAQADNDADDVADQARDASETAAQGDFKVAVARAEATHKIAIEKCEALQDNAQEDCKARADADLENAKRAAEARRDGAG
Other	-	MNRLLHTVVFFPLILLSACATGTVVSKSPEAKSPIPSGKSRIVVYRTQIVGMAVQPSVKVDGQKTGLCSPNGLFYVYVTPGVHEVSATTETTDTATVETRAGQTTYIECSVTTGIFVGRPSIIEVTESRAKPKISGLSFTGQY
Other	-	MTRQQRTPMVRGGIALVLTASVLTGLTACGSKNDADKFAEKLRTKGYGKVHVSADIERKGGKKRTVAYDAHVLVNTDADPQTCDVELENDVRSSGRGLVDYFDVDEVRDARGSGHEVEDDRTWPDNPTLTQLRAELVEHSIDC
Other	-	MRSRARRVLLWAVISGFTALGCEGATPDPPTGEPDGGTSLPDAGPGSEEPSGCPVVPAPTACPSPAPHYPDVAPIFDRRCVVCHSGSPGGPWSLADYGHVADWQDTIRTNVRDCTMPPADAGVPMTLEERLAILTWIRCGLPR
Other	-	MNHYLKRFIPALAVLGLTACAGMHGQHAHHGPLDTAPPGGEYAQVSELVPLPAFIPGLGTLYVQPATLPAGPFLAYDRDDRLVSTIYMIPLDDLNARKNFPELATAERKVVRTELAYNAGHPGVAEPHYHIVLWHVPPAEAKLK
Other	-	MPRFMLNRSAALLALAAAAVLPLASCANAGTNSSTTQKDVMTSGVGPMLEEIYASRKPVRFEQIDVSDIVAKYIPPSAEKSTVLETFGKSPTSKIVENTPDKIVVRDNKGQAMLDPDARSIVMTFFLDADGKVTKVDAVHIKNQ
Other	-	MRKITLLSLITITTLFLLGCDAQKTEQASAGSISLANPASTYCVAQGGTLSIESEEDGQVGYCTLPSGERVEEWQLYKRDHKESNEDIEVTEMLHTPNPAAEYCLKLGGTSDLSSGNCILPNGEKIDQWQLFRRDHLFIDPQNR
Other	-	MRKMMTAGLLAISGVLAASMGCIAPPADEPIADEGVAAEVEEAAAEPTGEAADELSVCGGGLVCQWAFASGAPQCEQYPSGPRLYCCPSGYGLVNNTCVPKCGSGLQCSGSSIPGSHSCTQRDTGSSVIHCCESGQRISGGRCVW
Other	-	MNMRKASPRSLWQRLGLATLLAGLLVLGGCMNQPTHAPDSRTAVIAPNSGTLTSQQVFVTLLTTLAERGFIIERADPDLERLDASYAARPPLALEAWVSEVNGQIRLSVSGDSNGADIAPGRLDNLLVEVAVALDARTVPVIGAP
Other	-	MRYLLLFILCISLWSCSTSKTTSKKEPISKDALAQLQDKRWVLQKLPDTTVPKMTKQIYILFDKNGVEVKGFGGCNGFGGSYKTDDKGLHLSNMISTQMWCDYGPVEQKFMHAIQDCDNFIITGDDMQLLKNNKPVAYFTAVYLNN
Other	-	MTNTFHATLLATLAFIISIALSACAQTKALDQGKPGKAFVADTSVIAIFNDRSKINFSSEELSLVDSILQQCINDYDLMNISAYKRQYVPAINDKGEKVVWVNCFCGTSRNWKKDLIVVNDGGSCYFNVTINLTSHKYENLIVNGVA
Other	-	MTNAIRPSNPVARLAIGLLLGTAFGCGEAPEPYRAIQGRVTYQGKPVPHAVVVFESTAQELTIAADADEQGIYKAIRSVNVPGLPIGSYRVAVTPPLFYPGLGPVGKNPGPPTREDIPASYRESATSGLTIDVNKEGSDASFDIEMK
Other	-	MKKALLLASSVLLGSVLVGCQTSEDSTTMTQTTPAAMKTVTGTLAYRERIALPENAVVTVTLQDVSLMDAPAKVIATQTFETKGKQVPFAFELAYDSAQIDARHTYSVSARIELNGKLRFITDTHYGVITDDNNTNQVDLKLVGVSAN
Other	-	MNKTLPNFLLISSLALIFLAGCSQDKSNEPVSGRWYTPNQLALGKTIFADNCAACHGTNAQSIPNWQQSLADGSYPPPPLNGSAHAWHHPLNLLVRTIEEGGAAVGGKMPAFKNVLNKQEQLAAIAFFQSYWDDRIYQIWKENGGLTK
Other	-	MRIPLTLSARPALLLSLLLGACAGGPPQRSSVNPNDPSLRRGEALVRHGAGSDALSPVAPAPAVQDLGAQAGFGRAWTASSVRASVYLFNTYQEARVAEDWLKAHVPEGLRGAGTVNGDLLVWATADATDEAGRAILENLIGSFAGEE
Other	-	MRNWTMAILPLALGMTVTACNGTDHDGKPQGKSEAGAPAAAGPVTTQSYALTGFTGVEVAGPDDVTIRQGDNFSISARGRADVIDRLEIKVDGTRLVIGRKRDGFSFSSRDDDDLDIAITMPRLDALRLTGSGSIDADAAGGMPLKPS
Other	-	MHSPLRSASFVVLLFALEGCSWLPSWLGGAPAVVNTTIASPLLIEDAGLLATRDRRAEIGIRLNNASSRPLWVGVRVQTPGGRTDCVILKELPPQAHQLFTCPQTKVFADVDYAVVIDSYRDPGLNERLAPLQTLLRFNANDLNTIGQP
Other	-	MDIRTISATLALLGSGLTLGGCNKAQTDATEVPGNAGAAGEKSCKGHADSDKSCGGHAEGEKSCGGEKGCGGEKGCSGVKGDTSCAASKPTETAATDAAPAADPATTATATAPADAAATPATTTEKKKKKPAAKKAKGEAACGEGTCG

Other	-	MKHYQITAAMMVLMTSGCASIVSKSSYPVTIRSEPQGASVLIKDKHGVAIHKGTTPFTVTLASGKGYFGPGDYTLDFELSGYQPHHTVMQAGVNPWYLGNIFIGGAIGFLIVDPLTGAMWKLADQKSVTLNAQATDIIPPLASPAETSQP
Other	-	MKKRYRVFLALLVMFATGACLSKMSSHEMLSLQQLKWKHRIVVTTLVDNDAVNALAEQIKKNSDFFDERKLIVVVKVNDMLVEIKQNIMIEGNNKTQFLNRLQNNNTILIGLDGGTKATYPALNWQEIFADIDSMPMRRNEIERNIKDNK
Other	-	MRPNLSIDKIAGALAVLGTSVLMGACGGESKPAEAPVSGTEVAPAGEKAGEHKAGEANCSADHKGSASCGAGKGSASCGAGKADPAAAAPATTPAAAAPAAAAPAAADAKADSKAPAAAPTGSSKKPATPPAAGKKAGGASCGAGTCSAKK
Other	-	MKISTKLLTIMFVPVFIVACGGGGGGGGGSSTPAPTPTVETIDNTQDIVAERNFSFDVGETITISMNYQGSADGALHLYTKAAFTTENGVVVADPMSRITTIYPTRTNEVELEVNGNWSQLYAQWVPMSANESEKNWVVSLNQSNNNYHLDF
Other	-	MHSFAKPGFACVVALLLSSLAGCASTGANSPSARPVLYPNASLNRVGDAQGRAEADACMARAVSAGLTPDEKNNAVARGAGVGAATGAVASAVGALITGRGGEGVVRAGAAGAAVGGSAGAVQGAFRNDRPSSTYRNFVQRCLGDRGFEVIGWN
Other	-	MFKKALVLALPVVLAGCSAPKFTAEPIAQSNQSKEITIVKDDATREVFMDSLQEWCLDTARKCTVVSDGTAPKTDELTLTYVSRWSWDLRTFIADAKINAYKNDQKVGHVEFKASNNGNLDKFGDDTKRIESMIQILFGEQTISDAQQKIKSGEI
Other	-	MKKIIPSLVLIGACLCGCAESAAPAQPGPARPPVVMDEGTPWTVDDVTYTVTWVTLPHGYGGDPAPHVEINVHVDNHGKNYAHFPDPSAVYDGGATAVAGWTQPPSGAEIAPGHTADFRSSFPLRMPGGTLRVRVLAFTGDVEELGYWIGTVHSG
Other	-	MKKLLLVCGLSALLIGCSDNEVGDVSLGIFTLKDIKLNSLVDPLVPGVTCHVASIEADLSLADPSDSSISCRQTSEITPEMIAQINKSKDGEVIFTKSKSIFFKSMKIRRIFDQKHQTLMYLSYSTKETSGSFKHSLSTVPLWGTKAYVEPAQQK
Other	-	MHLMLLGSMLGLALLLAGCASSPRPDRQPAFRTLSENQSSDIAIHALGLVGTPYRYGGNTPESGFDCSGLIGYVYKASAGVAPPRTVAQLVDFGGSIDASEARTGDLVIFGGSRPFHAGIFVGEGRFVHAPSTGGRVRLDRLNNPYWQRQGQAFRRP
Other	-	MRARAAHALVTAGTAVFVVACDGTASSGDAAGPAVEPTITVRVRGLTVEAEVADSDAERRQGLMFXTHLPPGQGMIFDFGGETTSGFYMYRTVLPLSIMFVRDGRVVGVREMTPCPGDDPSACPVYYPDGVYTHAVEAPAHTFAGVVVGDPVAMGSD
Other	-	MAWRLTLLALLLSLLSSCGFHLRGNLPLSQFPAIYVQSEAHSELAALLKQRFSQNQVELLGSYQQDRPALQLVRDTLERRTLSLFANGQVAEYELIYKVEYRIQLPGEQEQFYQFELYRDYQDDPNQALAKAQELELLLSELRQQAANRIIRQLARLG
Other	-	MHRSIPISLLLATMLAACAQTGQHPAAAVQPTPAPAQARPAPAAAPAAATTSTSVAPTPAPVVQADEPNVVAFATGSDKLDDAASAKITDLVEQARQAKKVEVLGSAQVNGKFNKKLALSRAFAVKRALAHAGVRPGKIMVRYSTIESRDVATITFKP
Other	-	MNLKAIGLVLLSALHVSGCGSDAHAGNEIETPPVEGGKNTCTGFGSPHINVFIRESLNSDVLIENASVRVVMESENEESTVEPVFISSDDRNEDTKTGAYHAILEHNNLAFDVSIVAFAEGYYPFVTKNISFEVNTGCGASNDLTYDVYLCPVGTTCL
Other	-	MHKHVFSLFVMLLFIHACATMGASQKNAESPASPPLTSILDSPGLKAFKRTLNQIVLERNPGTGQSQHFFVSKYSENESLAYMFWQEKKLLWIMSLGATDEESWLGVRYPSSGQLIDLKNSVVASVDDVGGSSYLVTKEWAAERLFDTVVHGDLIVISQ
Other	-	MKLFNIILVLLVVSGCKNSNTSENKLSNAIKVIGAVKAQEEQEAIDAMEDTKAMETLDWQGTYFGITDCANCDGIETELVLKDNEYILSLKPTNTDEEKFVQKGTFTWKGSIIILEDIIGSASMYKIEENQVKQVYYIDNKIQGEDWKGYILKKMTNSK
Other	-	MMRVTQRSLLSAGFCLMLAACGGKALAPPPPPTIVQLAVSADAGVNPDAKGRATPVVVRYYLLGNAGPFEAADFFSLFDGDEKALGATMVSREEVTLRPGDSVSSRLAPMQEAKALGVFVAFRDPNKTQWRAVVPVPANKTTAYKVSVLRDRVTITPAP
Other	-	MRYALLAGFAAIALSACSQGADEAPADPVENAWVRLPAVDGRPAAAYFTLHGGDAGDTLIAVDSERVASIELHETTMEGGAMRMRPIMSVDIPAGETIAFEPGGRHAMLFGVDPEVTAGTNLTLHFRFDSGRDVSIEAATIDAGDDAPAQESGQDHGAH
Other	-	MRIVGVLGCLLVLGACGSEPEVGCTMIGTPVGIGLDVAMPEVRTATMQVCWDDKCVDPVVELHPSSSAGPQTCTGTAPTDSCGVSVVPTGGRNGFADVPDLPTAPVRVTLRLSDANGVVLVDEVLTATPKPAYPNGVECGGQGPQTGLVVSAAGVVTERT
Other	-	MKIMRLAAAAALLTLAACSTSPTAPAPAASSPATRSDLTNGQGMIGGGTRAEP
Other	-	MKAVIIILASATLFTGCVTHIPVGHHHGHSVIVKKKHGHHRHHHRGHRKHIRIIR
Other	-	MQKNIYTRYLSSCLFVGCAHSPQSHSSLCSRGFAPLPSRSILKSIGYDTISIQLT
Other	-	MRRVPAGLGVLPALTGCSGLQEAAQDEGQDGRKASVKGGVTSVGLAFDGKDRVGAC
Other	-	MKSIILVLVSVALLSGCQLTRVEGEVDDMEVKVGTKENNDDNGKFCPPGQKKKGKC
Other	-	MNKLSILLALAAALAVAACDNNIPPTKAPGTADQYPQVDPAPNLTSGGDQSGTPPAQ
Other	-	MKLLAAFLALAILSACAARARVPPAPVAAPPAAVQPAVEPLPAGVKPLTVEQDEQISQG
Other	-	MTPVRPIFTAFACAALLALGACAARKPSEAEVLTVACSAGIAEACGYLSRGRSVTGTPL
Other	-	MKRIMRTWGLLAGLLWLSGCVAYVPYGHRPAGYYAPHGGYGYDAGPRHGGGWHHRHRHHR
Other	-	MRGALPLLLILAASGCGDRRTFDDRFNDTQANLQKRARTLDEQLNHQADETANNQQAPTGR
Other	-	MKTRGLWLLAALALASCSDDSPPPTPEQPQGLSSCLESPTQLARPPSGQLPCELLPPGFGT
Other	-	MRILIPWALSALLLAGCSALPTASERQAHATGLAASKGWLPSHIPAGNFDLMAYAPPSLAAW
Other	-	MTQFKRLKRAKAGAIVALLSLLCGCTLGGAAAGGYAGNRLTHGSAVGTVGGAVAGGIVGHEVGEHW
Other	-	MMFRKLLAAAGCACALAGCSAAVAVADATVTVVATGVKVTAKTVGAVADAVIPDGEDDAAQSAPQD

Other	-	MVNAALKKITPLLVLAVMLAGCQSHEGPAEKAGKKIDNAASQVKKDVGNATDKAGKKIEDAGNSIKNN
Other	-	MTKSMTRSLFLAATFAGVVGLAGCAGNGYDSGASTSTDASAQAPMTSDGSTAAPGTTAQPMPTSPAPTAQP
Other	-	MNKKIKIGGLSLLTAAFFIFNTGCKKRETTLDSKPALHKLNGTLSLSTKNLKSYHYAYYGNEKIELNSELPKCL
Other	-	MTKKECNATHDLMNRSPAIAAALVAALTSGCVQVSAPEEPIVIELNINVQQTVDVNLQEDVENLIENNPELFPQ
Other	-	MKKITLMFMVVWGLSGCGGSGDDHASNNTQTQSDPVLRETLTLTNQSQSSFSQSEPKSLKDISEASIDNKAEPLTVKF
Other	-	MRLVSLGTRWTGAVWAPALTAGCTKNDNPVSAELLRGPGHADNASSHGGLGPAPSPNAGTAPPGAVPLDPAGKRIRLRC
Other	-	MAKKMTIVALPLAFALTTSIVSGCSNEESHPKTKQTPASKQQTSTSERKLQQYAKDPDAKKTDEDFDLVGVVKSVKPLKKSH
Other	-	MKTTLIVLLSLCILSACQTADVIDTAGHVLTPTEIRHDGAPNEVVRFGVNACGHLKAQCRVEHYQEWPALFRDVGCRCDRSH
Other	-	MIRTSGIALAALYLIGCAGDSPPTSGECRALLQSRTATRAEMVAFIVRAKRDLDAAPRNRIDSHTEGLGLCIGALMRKAQSIK
Other	-	MRFRMVIAPCILMLGLGLGGCTRSCEDLAAEARAKSADAMHMAADAYADGASTEKIAAKMSNLQKDMIDLRNEMAGKNCPMTF
Other	-	MKLSFKSLVLFSAFAAFATTSCSERQQENTEAATENAVDQAGDATSEAANEVQADMATEPGDTAVVQNKEADKLVEKVPATPQN
Other	-	MTRSSVVGSVLVLGLAIALAGCTTVSGPGGAVARQGAGGGAGGGEGVARRVAKDSASEKARARITAQRPAAAPSRRMMVIGAGW
Other	-	MTTRLSTMLALSLVLTLVGCNQQNFQYDARYQLTEVDGVLYRLNQKTGQVDRLEQDRFVPMKEPLPDTMADAVQGQAEQESDGE
Other	-	MKPTHLIPAALLLALAACSKVTVANYDKLRAGQSYDEVQQLLGKPSECDDVLSARNCVWRSGKATVNVSFVGGQVILFTAQGLR
Other	-	MRLFAALPCLTLLATLAACTGDAPASNELQLENDGGGKFSGKAGPEWTGAELKQEAATSVCGGAEPATFKLSRKKDVWSFKGKC
Other	-	MFRRPGRAFAAVGLLLLLSGCGQTGPLYMPPPEAPAAPPAAESTPPAQPAQ

Supplementary Table 7. Protein sequences of predicted autoinducing peptide-lipoproteins. Sequences are grouped by cluster as shown in the SSN in Supplementary Fig. 27).

Cluster	Annotation	Sequence
I	AIP	MKKALAITLSAIGAVLSTVATTGCAFVLFDEPEMPRSLID
I	AIP	MKKALAIALSAVGAVLGTVATTGCIFVLFDEPEMPRSLID
I	AIP	MKKALAIALSAIGAVLGTVATTGCILALFDEPEMPRSLID
I	AIP	MKKRVAKLLVALGMLATASASVGCLWWSLDEPKALKGMD
I	AIP	MKKALAIALSAIGAVLGTVATTGCFAVLLDEPEMPRSLID
I	AIP	MKKRVAAFVSAIALLATGAASMGCAWILVDEPNNIKAIKD
I	AIP	MKKRLSQLLMAVAMLATGAASLGCWWWSVEEPDSVGVFND
I	AIP	MKKFLSVALAAIGAVIGTVGTSGCFVLLLDEPEMPASLID
I	AIP	MKLFATIAAAIGSLITATATTGCIMVVFDEPEMPESML
I	AIP	MKLFATITATLGAFLATIATAGCVIVYVDEPEMPESML
I	AIP	MKKLLAMILSGVGVLAAGAATTACVIVLIDEPSMPNKMIEK
I	AIP	MKKAFTYVLTAIAILATGAASMGCAIALTDEPKAPKSMMD
I	AIP	MKKLLSIGLAAIALLASTASSVGCMWIIIDEPKALKNMD
I	AIP	MKKLLSIGLAAIALLASTAASVGCTWVLIDEPKALKNMD
I	AIP	MKKALAIALSAIGAILGTVATTGCVVVLLDEPEMPKSLID
I	AIP	MKKKLAGVVAAVAMMLTGAASMGCMIFLVDEPVAPRDLID
I	AIP	MKLFATIAAAIGSLIAATATTGCIMVVFDEPEMPESML
I	AIP	MKKRLASILAAVAIMATGAASMGCIWIYWDEPNSYNLFCD
I	AIP	MKKALAIALSAIGAIIGTVATTGCVVVLFDEPEMPKSLID
I	AIP	MKKKLAGVVAAVAMMLTGAASMGCMFILIDEPVAPNALID
I	AIP	MKKALAITLSAIGAVLGTVATSGCIFAIFDEPEMPRSLID
I	AIP	MKKALAIALSAVGAILGTVATSGCIMVLFDEPEIPRSLID
I	AIP	MKKALAITLSAIGAVLGTVATSGCIMVLLDEPEMPRSLID

I	AIP	MKKALAITLSVIGAVLGTVATTGCLVVLFDEPEMPKSLID
I	AIP	MKKLLSISLAAIALLASTAASVGCMWILIDEPEALKNMD
I	AIP	MKKALAIALSAIGAVLGTVATTGCIFVLFDEPEMPRSLID
I	AIP	MKKALAIALSAIGAVLGTVATSGCIFGLLDEPEMPKSLID
I	AIP	MKLFATITATLGAFLATIATAGCVIIYVDEPEIPESML
I	AIP	MKKLLAMVLSGVGILAAGAASIACPMLIIDEPEMPKEMIER
I	AIP	MKLFATITATIGAFLATVATSGCILAYIDEPEMPESLL
I	AIP	MKKLLSIGLAAIALLASTAASVGCTWILIDEPKALKNMD
I	AIP	MKLFASIAAFVGSLIAATATTGCIFVVFDEPEMPESLL
I	AIP	MKKFLSIALAAIGAVVGTVATSGCIALLLDEPEMPNSLIG
I	AIP	MKKALAIALSAIGAVLGTVATSGCIFGLLDEPEMSRSLID
I	AIP	MKKFLSIALATIGAVIGTVAYSGCAFLLLDEPEMPDSLID
I	AIP	MKKALAITLSAIGAVLGTVATTGCLVVLLDEPEMPKSLID
I	AIP	MKKFLSIALAAIGAVVGTVATSGCFVLLLDEPEMPASLID
I	AIP	MKKFLSIALAAIGAVVGTVATSGCVLALFDEPEMPASLID
I	AIP	MKALFVSLISAVGALVAASATSGCILVWIDEPTMPKSLLER
I	AIP	MKKALAIALSAIGAVLGTVATTGCVMVLFDEPDMPKSLID
I	AIP	MKKALAITLSAIGAVLGTVATTGCFLALLDEPEMPKSLID
I	AIP	MKKLLSIGLAAIALLASTAASVGCMWILIDEPKALKNMD
I	AIP	MKLIAKITAFLGALVATTATTGCITVILDEPEMPETLL
I	AIP	MKKRMAKLLAAVAMLATASASVGCAWFCVEEPKALKNMD
I	AIP	MKKRVAKLLVALGMLATASASVGCIWWTVDEPKALKGMD
I	AIP	MKKLLSIGLAAIALLASTAASVGCMWVIIDEPKALKNMD
I	AIP	MKKRVAAFVSAIALLATGAASMGCVWMLIDEPNNIKAIKD
I	AIP	MKKFLSIALATIGAVVGTVATSGCIIMLLDEPEMPDSLID
I	AIP	MKLFASIAAFVGSLIAATATTGCVFIVFDEPEMPESLL
I	AIP	MKLFATIAAAIGSLIAATATTGCILAVFDEPEMPESML
I	AIP	MKKRVAAFVSAIALLATGAASMGCVWLLVDEPSNIKAIKD
I	AIP	MKKFLSIALAAIGAVVGTVASSGCVLALLDEPEMPASLID
I	AIP	MKKALAIALSAVGAVLGTVATSGCIMVLFDEPEIPRSLID
I	AIP	MKKRLSQLLMAVAMLATGMASTGCIWFILEEPNSIDTFCE
I	AIP	MKLFATIAATVGAFLATISTAGCLIIYVDEPEMPESML
I	AIP	MKKRVAKLLVALGMLATASASVGCVWWTVDEPKALKGMD
I	AIP	MKLIASILASVGALVASIGTVGCPALICDEPKMPKSLLNK
I	AIP	MKKALAITLSAVGAVLGTVATTGCVVIFLDEPEMPKSLID
I	AIP	MKKALAIALSAIGAVLGTVATTGCVVLLFDEPEMPRSLID
I	AIP	MKKRVAAFVSAIALLATGAASMGCAWMFVDEPNNIKAIKD
I	AIP	MKKVLAIALSAVGAILGTVATSGCVMVFLDEPEMPRSLID
I	AIP	MKKLLSIGLAAIALLASTAASVGCTWIIIDEPKALKNMD
I	AIP	MKKFLSIALAAIGAVVGTLATSGCLFLLLDEPEMPVSLID
I	AIP	MKLIAKITAFLGALVAATATTGCITVILDEPEMPETLL
I	AIP	MSKVFAKIAAALGGIMAYTTVGACSWGWLEEPEMPKSLIK
I	AIP	MKKFLSIALAAIGAVVGTVATSGCFLLLLDEPEMPASLID
I	AIP	MKYLSSLLAVLGTLAATLGTQGCSYLICDEPKMPKSLLNK
I	AIP	MKKALAIVLSAIGAVLGTVATTGCIFVLFDEPEMPRSLID
I	AIP	MKKLFAMVLSGLGILAAGAATSACVCLVIDEPEMSKEMIER
I	AIP	MKLFYSILAAIGAVSATIGTQGCVVLLLDEPKMPKSLLNK
I	AIP	MKALFVSAISALGALLAATATSGCAIVFIDEPTMPKSMIER
I	AIP	MKKRVAAFVSAIALLATGAASMGCIWILADEPNNIKAIKD
I	AIP	MKKLFAMVLSGLGILAAGAATSACLCLVLDEPEMSKEMIER
I	AIP	MKKRMAKLLAAVAMLLTTTASVGCVLFIVEEPKALKNMD

I	AIP	MKKALAIALSAVGAFLGTVATSGCLFVLLDEPEMPRSLID
I	AIP	MKLIAKITAFLGALVAATATTGCITIILDEPEMPETLL
I	AIP	MKKRVAKLLVALGMLATASASVGCVWWSLDEPKALKGMD
I	AIP	MKKRLSQLLMAVAMLATGAASVGCVWWFYDEPDAVNIFIND
I	AIP	MKKRVAKLLVALGMLATASASVGCIWWAVDEPKALKGMD
I	AIP	MKLFATIAATIGSLIAATATTGCIMVVFDEPEMPESML
I	AIP	MKLFATIAAAIGSLIAATATTGCIVAVFDEPEMPESML
I	AIP	MKLITSILASVGALVASIGTVGCPALICDEPKMPKSLLNK
I	AIP	MKKFLSIALAAIGAVVGTVAYSGCAFLLLDEPEMPASLID
I	AIP	MKKRVAKLLVALGMLATASASVGCIWWTIDEPKALKGMD
I	AIP	MKKRVAKLLVALGMLATVSASVGCVWWALDEPKALKGMD
I	AIP	MKLIAKITAFLGALVAATATTGCITVILDEPEMPKTLL
I	AIP	MKKLLSIGLAAIALLASTAASVGCTWVIIDEPKALKNMD
I	AIP	MKKRLSQLLMAVAMLATGATSVGCFWVVLEEPDSLDLFKE
I	AIP	MKLFYSILATIGAVSATIGTQGCSYFLVDEPKMPKSLLNK
I	AIP	MKKLFSVALAAVALLASTAASVGCMWLFIDEPEAPKFMD
I	AIP	MKKALAITLSAIGAVLGTVATTGCVMVIFDEPEMPRSLID
I	AIP	MTKRVAKLLVALGMLATASASVGCVWWSLDEPKALKGMD
I	AIP	MKKLFAMVLSGLGILAAGAATSACLLIVLDEPEMSKEMIER
I	AIP	MKKALAIALSAIGAIIGTVATTGCILALFDEPEMPRSLID
II	AIP	MKKLMSIARKATTTLAALALVIATTSVPGACYHWFAQPVEPEELRKFMNDK
II	AIP	MKKLMSIARKATTTLAALALVIATTSVPSACYHWFAQPVEPEELRKLVNDK
II	AIP	MKKLGKLFKLGISLIAALSAIAAMGISVNACHLWFAQPKVPKELDAFRNENK
II	AIP	MKKTSKVLRMAASLMAALSAVAAMGISVNACHFWFAQPKVPQGLDKFRSEK
II	AIP	MKKLMSIAKKASTALAALALVVATTSVPSACFHWFAQPVEPEELRNYVNRK
II	AIP	MKKLMSIAKKASTALAALALVVATTSVPSACFHWFAQPVEPEELRNYVSKK
II	AIP	MSIAKKASTALAALALVVATTSVPSACFHWFAQPVEPEELRNYVNKK
II	AIP	MKKLMSIAKKASTALAALALVVATTSVPIACSHWFAQPVEPEELRNYVNKK
II	AIP	MKKTSKVLRMAASLMAALSAVAAMGISVNACHFWFAQPKVPQGLDKFRSKE
II	AIP	MKKLMSIAKKASTALAALALVVATTSVPSACFHWFAQPVEPEELRNFVNNK
II	AIP	MKKIILNKCIFVMSNLVLFIATTSISQCCNTQIYQPSVDEDLKNKIIQANKK
II	AIP	MKKLMSIAKKASTALAALALVVATTSVPSACFHWFTQPVEPEELRNYVNKK
II	AIP	MKNLMKIARKTSFVLAAMALVVATSSVASCCYHWFAQPVEPEELRKFVGEN
II	AIP	MKKLMSIAKKASTALAALALVVATTSVPSACFHWFAQPVEPEELRKYVNKK
II	AIP	MKKLMSIAKKASTALAALALVVATTSVPSACFHWFAQPVEPEELRNYVNKK
II	AIP	MKKLMSIAKKASTALASLALVVATTSVPSACFHWFAQPVEPEELRNYVNKK
II	AIP	MKKLMSIAKKASTALAALALVVATTSVPSACYHWFAQPVEPEELRNYVNKK
II	AIP	MKKLMSIAKKASTALAALALVVATTAVPSACFHWFAQPVEPEELRNYVNKK
II	AIP	MKKLMSIAKKASTALAALALVVATTSVPSACYHWFAQPVEPEELRKYVNKK
II	AIP	MKKLMKLAVKASTVLAAMAMFVATSSVSACCHCCFAQPVEPEELRNLVNNK
II	AIP	MKKLMSIAKKASTALAALALVVATTSVPSACFHWFAQPVEPEELRNYVNKE
II	AIP	MKKLMSIAKKASTALAALALVVATTSVPSACFHWFAQPVV
II	AIP	MKNLMKIARKTSSVLAAMALVAATSSVASCCYHWFAQPVEPEELRKFVGEN
II	AIP	MKNLMKIARKTSSVLAAMALVVATSSVASCCYHWFAQPVEPEELRKFVGEN
II	AIP	MKKLMSIAKKASTALAALVVATTSVPSACFHWFAQPVEPEELRNYVNKK
II	AIP	MKKLMSIAKKASTALAALALVVATTAVPSACFHWLAQPVEPEELRNYVNKK
III	AIP	MKNKVLRSLVKVLTVAAFVCASTSSLCNCYEPEKPASLR
III	AIP	MKYRILNSMVKVLTLVAFVCAMSPSRCNCYEPEKPASLR
III	AIP	MKNKVLRSLVKVLTVAAFVCASTSSLCNCYEPEKPVSLR
III	AIP	MKNKVLRSLVKVLTVAAFVCASTSSLFNCYEPEKPASLR
III	AIP	MKYRILNSMVKVLTLVAFVCAASPSHSNCYEPEKPASLR

IV	AIP	MKKLNKNLLTLFAALTTVVATTVATSACIWFTHQPEEPKSLRDE
IV	AIP	MKKLNKNLLTLFAALTTVIATTVATSACIWFTHQPEEPKSLRDE
IV	AIP	MKKKLFMTVATVATLVASFVATSACWACFYQPEEPKSLRDE
IV	AIP	MKKKILMSLAAISTFIASIVATSACTWYFYQPEEPECLRDK
IV	AIP	MKRRILMVVATAATLIASIVSTSACIWGHYQPEEPKCLREE
IV	AIP	MKKKILMGIAAVATVFASVISTSACYFSFYQPEEPECLREE
V	AIP	MEKFKQYSLKTFAALLSFVAISSVNSACAVIFGQEKEPDSLAKYKKC
V	AIP	MSKKDFGKKFLNHLASLALIAGVISSSTACRFVLHQPAMPDKMKKLLGEK
V	AIP	MKKEKFIKSFANVLAAVAVTVTSGVATTGCFAFLHQPELPKGAEKLRKFK
V	AIP	MQKFKNFIMKFGSSFAVLALIIGISTSNSACGMIFHQPKEPAAMNKFKF
V	AIP	MKKMMLFLSKFTCIMAMALAVLSVNSTCGFTAYQPDVPESLNHR
V	AIP	MQKIKNFIMKFGSSFAALALIIGITTSNSACGMIFHQPEEPAAMNKFKF
V	AIP	MRKKGFGKKFLNHLASLALIAGVISSSTACRFVLHQPAMPDKMKKLFSEK
V	AIP	MQKFKNFIMKFGSSFAALALIIGISTSNSACLMVFHQPKEPAAMNKFKF
V	AIP	MEKFKQYSLKTVAALLSFVAILSVNSACAVIFGQEKEPDSLAKYKKY
V	AIP	MKKEKFIKGFANVLAAVAVTVTSGVATTGCFAFLHQPELPKGAEKLRKFK
V	AIP	MQKIKNFIMKFGSSFAALALIVGISTANSACDIIWHQPKEPTAMNKFKF
V	AIP	MKKIMLLLSKVTCIMAMALAVLSVNSTCGFTAYQPDVPESLEHD
V	AIP	MKKSMNFIKKLSTVVVTCLTLVLTINANTASCFILNEPEEPKNIEKFKMFK
V	AIP	MEKFKQYSLKTVAALLSFVAISSVNSACAVIFRQEKEPDSLAKYKKY
V	AIP	MQKIRNFIMKFGSSFAALALIIGISTSNSACGMIFHQAKEPEAMNKFKI
V	AIP	MKKEKFIKGFANVLAAVAVTVTSGVAITGCFTFLHQPELPKGAEKLRKFK
V	AIP	MKKFKNIIKKISPAIVGCLTLVLTINANTASCFILNEPKEPKTIEKFKLRK
V	AIP	MKKMMLFLSKFTCIMAMTLAVLSVNSTCGFTAYQPDVPESLNHR
V	AIP	MEKFKQYSLKTVAALLSFVAILSVNSACAVIFGQEKEPDSLAKYKKC
V	AIP	MQKIKNFIMKFGSTFAALALIVGISASNSACNILWHQPEEPAAMNKFKF
V	AIP	MRKKGFGKKILNHLASLALIAGVISSSTACRFVLHQPAMPDKMKKLLGEK
V	AIP	MEKFKQYSLKTFAALLSFVAISSVNSACAVIFGQEKEPDSLSKYKKY
V	AIP	MKKEKFIKGFANVLAAVAVTVTSGVATTGCFTFLHQPELPKGAEKLRKFK
V	AIP	MRKKGFGKKFLNHLASLALIAGVISSSTACRFVLHQPAMPDKMKKLLGEK
V	AIP	MLIFGKFTCVMAMALAILSVNSTCGFTAYQPDVPESLNNE
V	AIP	MKKTKNIIKKISPVIVGCLTLVLTINANTASCFILNEPKEPNLINKFKIFK
V	AIP	MKKIKNLIKRISPVLTACLTLILAINANTASCFILNEPKEPNEIQKFKIFK
V	AIP	MLKKLTIQSAKILAVLALAVGTISGQVACSGVYYQPKMPEKMKK
V	AIP	MKKKGFGKKFLNHLASLALIAGVISSSTACRFVLHQPAMPDKMKKLLGEK
V	AIP	MVYNFKKICLSLFARLLTLLTIIGVNSACNIVYGQPNEPQSLARYKKR
V	AIP	MKKIKSLLKKFSPIMVSCLTMVLAINANTASCFLINEPQEPSSIENFKKFK
V	AIP	MKKIKRFLSIISPAITGCLTLALAINANSASCFIINQPKEPETIKKFKMFK
V	AIP	MKNCKQYFLKTFAFLLSFLAISSVNSACAVLFGQEKEPDSLARYKKC
V	AIP	MKKGKKFFLNSFAFLMSFLAISSVNSACAIVFGQEEEPESLKRYKKIK
V	AIP	MEKFKQYSLKTVAALLSFVAISSVNSTCAVIFGQEKEPDSLAKYKKY
V	AIP	MEKFKQYSLKTVAALLSFVAISSVNSACAVIFGQEKEPDSLAKYKKY
V	AIP	MKKIILKYGGMLAALAMVFTTLTVNSTCTWMTYQEELPETAKKLRKF
V	AIP	MEKFKQYSLKTVAALLSFVAISSVNSACAVIFGQEKEPDSLSKYKKY
V	AIP	MQKFKNFIMKFGSSFAALALIIGISTSNSACMMVFHQPKEPAAMNKFKF
V	AIP	MKNGKKFFLNSFAFLMSFLAISSVNSACAIIFGQEEEPESLKRYKKIK
V	AIP	MTYNLKKLMLNAFAGLLTLLAIVGVNSACNIVFGQPEEPESLSRYKKIKG
V	AIP	MEKFKQYFLKTVATLLSFVAISSVNSACAVIFGQEKEPDSLAKYKKC
V	AIP	MEKFKQYFLKTVAALLSFVAISSVNSACAVIFGQEKEPDSLAKYKKY
V	AIP	MKTHNKKWMLIFGKFTCVMAMALAILSVNSTCGFTAYQPDVPESLNNE
V	AIP	MKKIILKYGGMLAALAMVFTTLTVNSTCTWMTYQEELPDTAKKLRKF

VI	LptM chaperone	MSKREKMKKLLSVFLLSAVCALVSGCGVKGPLYFPEKEPAQQQVK
VI	LptM chaperone	MKKLLSILLLSAFCTLATGCGVKGPLYFPEKEQPQNTQNTQ
VI	LptM chaperone	MKKLLSILLLSTFCTLATGCGVKGPLYFPEKEQPQNTQ
VI	LptM chaperone	MKKLLSILLLSAFCTLSTGCGVKGPLYFPEKEQPQNTQKTQ
VI	LptM chaperone	MKKLLSILLLSAFCTLATGCGVKGPLYFPEKEQPQSTQKTQ
VI	LptM chaperone	MKKLLSILLLSAFCTLATGCGVKGPLYFPEKEQPQNTQKTQ
VI	LptM chaperone	MKKLLSILLLGAFCTLATGCGVKGPLYFPEKEQPKNTQ
VI	LptM chaperone	MKKLISVFLLVTLCALSTGCGVKGPLYFPEQDSAQKTNK
VI	LptM chaperone	MKKLISLFLLVTLCALSSGCGVKGPLYFPEQDTAQQATK
VI	LptM chaperone	MKKLLSILVLSTICIMATGCGVKGPLYFPEKEQAQQTK
VI	LptM chaperone	MKKVLSILLLSAFCTLATGCGVKGPLYFPEKEQPQNTQKTQ
VI	LptM chaperone	MKKLLSILLLSAFCTLATGCGVKGPLYFPEKEQPQNTQ
VI	LptM chaperone	MKKLLSVLLLSAFCTLATGCGVKGPLYFPEKEQPQNTQKTQ
VI	LptM chaperone	MKKLLSILVLSAICILATGCGVKGPLYFPEKEQAQQTK
VI	LptM chaperone	MKKLLSILLLSVFCTLATGCGVKGPLYFPEKEQPQNMQKTQ
VI	LptM chaperone	MKKLLSILLLSTFCTLATGCGVKGPLYFPEKEQPQNTQKTQ
VI	LptM chaperone	MKKLLSILLLGAFCTLVTGCGVKGPLYFPEKEQPQNTQKTQ
VI	LptM chaperone	MKKFISLLLLGAICVLSTGCGVKGPLYFPEQEQASNN
VI	LptM chaperone	MKKLISVFLLITLCALSTGCGVKGPLYFPEQDSAQKTSK
VI	LptM chaperone	MKKLISVFLILTLCALSTGCGVKGPLYFPEQDSAQKTSK
VI	LptM chaperone	MKKLLSILLLGAFCTLATGCGVKGPLYFPEKEQPQKTQKTQ
VI	LptM chaperone	MKKLLSILLLSAFCTLAAGCGVKGPLYFPEKEQPQNTQKTQ
VI	LptM chaperone	MKKLLSILLLSAFCTLATGCGVKGPLYFPEKEQLQNTQKTQ
VI	LptM chaperone	MKKLLSILLFSAFCTLATGCGVKGPLYFPEKEQPQNTQKTQ
VI	LptM chaperone	MKKLLSILVLSAICIVATGCGVKGPLYFPEKEQAQQTK
VI	LptM chaperone	MKKLISVFLLVTLCALSIGCGVKGPLYFPEQDSAQKTSK
VI	LptM chaperone	MKKLLSILLLGAFCTLATGCGVKGPLYFPEKEQPQNAQKTQ
VI	LptM chaperone	MKKVLSILLLGAFCTLATGCGVKGPLYFPEKEQPQNTQKTQ
VI	LptM chaperone	MKKLLSILVLSAVCIVATGCGVKGPLYFPEKEQAQQTK
VI	LptM chaperone	MKKLLSILLLSAFCTLATGCGVKGPLYFPEKEQPQNTQKHNKN
VI	LptM chaperone	MKKLLSILVLSAICIAATGCGVKGPLYFPEKEQAQQTK
VI	LptM chaperone	MKKLLSILVLSAVCIAATGCGVKGPLYFPEKEQAQQTK
VI	LptM chaperone	MKKLLSILVLSAICIMATGCGVKGPLYFPEKEQAQQTK
VI	LptM chaperone	MKKLISVFLLVTLCTLSTGCGVKGPLYFPEQDSAQKTTK

VI	LptM chaperone	MKKLLSILLLGAFCTLATGCGVKGPLYFPEKEQPQNTQKTQ
VI	LptM chaperone	MKKLLSILLLGAFCTLATGCGVKGPLYFPEKEKPQNTQKTQ
VI	LptM chaperone	MKKLISVFLLVTLCALSTGCGVKGPLYFPEQDSAQKTSK
VI	LptM chaperone	MKKLLSILLLSAFCTLATSCGVKGPLYFPEKEQPQNTQKTQ
VI	LptM chaperone	MKKLLSILLLSAFCTLATGCGVKGPLYFPEKEQSLNTQKTQ
VI	LptM chaperone	MKKLISVFLLITLCVLSTGCGVKGPLYFPEQDSAQKTSK
VI	LptM chaperone	MKKLLSILLLSAFCALATGCGVKGPLYFPEKEQPQNTQKHNKN
VI	LptM chaperone	MKRSVSILLLGAFCTLATGCGVKGPLYFPEKEQPQKTQKTQ
VII	TolC-subunit	MNTSKIIACGATLLLSSCGIYTSYEPQTSVPENLYGEEVAEAVSNKL
VII	TolC-subunit	MNTLKIIACGATLLLSSCGIYTSYEPQTSVPENLYGEEVTEAVSK
VII	TolC-subunit	MNNMNTSKIIACGATLLLSSCGIYTSYEPQTSVPENLYGEEVTEAVSK
VII	TolC-subunit	MNNMNTSKIIACGATLLLSSCGIYTSYEPQTSVPENLYDEEVTEAVSK
VII	TolC-subunit	MNNMNTSKIIACGATLLLSSCGIYSSYEPQTSVPENLYGEEVTEAVSK
VII	TolC-subunit	MKKQIITLTVAALTLSSCGIYTQYKPATEVPDNL
VII	TolC-subunit	MKKLIISLAAILALSSCGIYSKYKPVTEIPD
VIII	AIP	MNKWIYRVCTKLAACAFLAAIVSVGTASCNGMYQPEIPSQLK
VIII	AIP	MKKIMYQVCTKLAACAFLAAIVSVGTASCNGLYQPKMPDKLVK
VIII	AIP	MKKIIYQVCTRLAACAFLAAVVSVGTASCGGLYQPEVPSKPVK
VIII	AIP	MKKIIYQVCTKLAACAFLAAIVSVGTASCNGMYQPAVPKQLNKQS
Other	N/A	MTKSINVFSAILLNLFNLVSKSGCSSMWGEPDYPEELL
Other	N/A	MSEKLRKFLVMAAGVLVILETTVISGCRANISGTESDRMVEYTVICGDEIPEELKSR
Other	N/A	MRKYIQIISFLLAAVTLSGCENWLDVKPETEVNG
Other	N/A	MRKNFLWIMATAIIFTSSCAKAPEEVKKGK
Other	N/A	MRKFKSLITVLAITVTSAALFTGCGNTGSNDSNSSSQLQDESK
Other	N/A	MRIKRLLYAIATILPFLFLCSCYEEQEPQQEKQDKEK
Other	N/A	MQKLLNCFSTLLINIFGLVSNTSCAGYYGEPDYPEELLK
Other	N/A	MPMERHEQTNSIKKMDYRNMKGIFLAAALAAVFTTGCWAQQPASPDFVTQKN
Other	N/A	MNMKKIASVVLALALSVSVFAACGSNGNSSASSTACLLYTSDAADEL
Other	N/A	MNLKKIFFSAVTVSVLCALTGCDYIEEGKPESSLLKQQEEH
Other	N/A	MNKKNFSGGITFALVVSLSIVTSGCSSNIDDPTDAQNLC
Other	N/A	MNKFSIVLTLLLCGSCALALDPNLEKN
Other	N/A	MNIKVDLKKLFAAAVSAIMCLSAGGCYLLPDEEEVLDAP
Other	N/A	MNHLSSLIRLVFLPIVLIACNSTVEKPQEDKLQELQKK
Other	N/A	MNDYQKKVLSLALSVSLLFNASCNKDKVIYNLPPEPIKSEEPKELKLKPKKIDNE
Other	N/A	MNDYQKKVLSIALSVSLLFNASCNKGTVVYNLPPEPIKSEEPKELKIKPKKIDEE
Other	N/A	MMKKYLFLLLIGAASLTACGDDDDLVVPELDKLT
Other	N/A	MKTFFKTSCKKIAAILPAIALFFAVSPCVGKIYEPKLPEQLK
Other	N/A	MKTELARKLLSGLSLWVLLFALSSCGSKMDEDAAAAYFDESPAVTLYATCFFDEEQPNLKADFSVKFC
Other	N/A	MKSKIFYACLFAASVLTSCSDFLEEKPKGVL
Other	N/A	MKRNLKLALLMAFCLTATACGKTAEKPAEKRLKSKP
Other	N/A	MKQKLAVLLSLVMTISLTACSSAGTYSKSSTFASDDVK
Other	N/A	MKNLVKNFIVKHSKLLCSLMVVSAYASLTSCRFLYFQPEVPEGLDKIVK
Other	N/A	MKNLVKNFIVKHSKLLCSLMVVSAYASLTSCRFLYFQPEIPEGLDKIVK

Other	N/A	MKNLKNIFTVALIATCMSGCTGFLDIEPETTLTGKNFYTRQYGIDKS
Other	N/A	MKNLKKYIIIDILASVIIYLSGCASLTYNPHSQTEKVKVTLKNK
Other	N/A	MKNLKKYIIIAILASVIIYLSGCASLTYNPHSQPEKVKVTLKNK
Other	N/A	MKNLFHIAKKTFTWVTVTMAVSLFNGCSDFLDKQP
Other	N/A	MKNKFTHFLMAGALVCWSSYTLHAQTEVPMSVKR
Other	N/A	MKNKALVWMCGLLSAIMAFTSCTNFLEEDPKTFLSPSEY
Other	N/A	MKNKAHVWMCGLLSATMAFTSCTNFLEEDPKTF
Other	N/A	MKLSNKLNSLLVAPLAIALFAACSTDENLSDGG
Other	N/A	MKLSLVKYILLFCGVIPLLTSCESFLDKQETEDLTFE
Other	N/A	MKLKNILLCASVLGGLALSSCTGNFLDEDRNPNSLDP
Other	N/A	MKLKKLVPFVISVLMIFQVCVPFTFADEDPP
Other	N/A	MKLKKIIACAAAFAMTATCFAGCGSTSDSAGAK
Other	N/A	MKKYLFLLLIGAASLTACGDDDDPVVPELNKLTKS
Other	N/A	MKKVLVSSMLILGGLFSACSGFLDEDPKSKIPEEEAYKSEKLV
Other	N/A	MKKVILIAVALLGALTVSSCGPGYQPLSEEEWKKQQK
Other	N/A	MKKSKFKLFAALLLAAAMTVLSLSSACLLYTSDAADE
Other	N/A	MKKSDLFKIGVLLVATTLGTTGCSFGEDEKKPEIVVDPAEKTIE
Other	N/A	MKKSDLFKIGVLLMATTLGTTGCSFGEDEKKPEIVVDPAEKQ
Other	N/A	MKKRKIAMLVMTGVLLTGCMGTNYLEEGVSQLEEKQ
Other	N/A	MKKNKLLTALALASACLLYTSDAADE
Other	N/A	MKKLTALLLALVMALSLTACSFVMLPEEQEPPADPKQE
Other	N/A	MKKLLALLMALMMCCTAFAFAEEEAEVPAVEMNVSFEA
Other	N/A	MKKLILFVSKNLCNVAVFAAAFAVTTSCSRWFYQEELDEQVMSLNRYRD
Other	N/A	MKKLILFVSKNLCNVAVFAAAFAVTASCSRWFYQEELDEQVMSLNRYRD
Other	N/A	MKKLFLFPVMLLFALTITACSNSPNGFEEPETCLLYTSDAADE
Other	N/A	MKKKYFACAMGLVLALSLAACGEEEVVVENTNEEVTQEVQTQEVQTQASCCIVYQPKAPKELERYRKTK
Other	N/A	MKKKMGAFILCFALLGAGCVFAEEEKTSP
Other	N/A	MKKKLAFKLATLMCAVTSIGQIATCAAPNSLLLWGEPTPPMKKEDK
Other	N/A	MKKKFIYSFMIAAAAAAFSSCSNFLEERPTDAFDEETAF
Other	N/A	MKKIYRLLLTFMPALVLYTGCYAFFQEKIPMDISKDSSSLTDLKVP
Other	N/A	MKKINLLSALFLCTILASCASENTSSSSSYSSSSTISSS
Other	N/A	MKKILLALLTSCALVSCEGYFDQLPKTELPS
Other	N/A	MKKILFAFMAMYCFIACDPSDSFADNE
Other	N/A	MKKFLSVLLMVAGLMSCGDDDKPFIPELNKR
Other	N/A	MKKFLSLILILMLMTLSTGCAKEEEPECLSGTCPFYPTETLKVN
Other	N/A	MKKFLSLILILMLMALSTGCAKEEEPECLSGTCPFYPTETLKVN
Other	N/A	MKIKSILKLGTLALLTAALPACSFLDTDPQINPDDGYYNS
Other	N/A	MKHLSSILLLAFVCTMFASCYDEPDPAEREHDSNLIGE
Other	N/A	MKFKNIMKSCLSLCVMLVAYCSVNTACTLLVYQDELPEGVQRMREAKFK
Other	N/A	MFNLKYIENMKTELARKLLSGLSLWVLLFALSSCGSKMDEDAAAAYFDESPAVTLYATCFFDEEQPNLKADFF
**Other**	**N/A**	**MFKIAKKLTVIMLSASIIFSCSGIIAHAEGIKELLEKQMKK**

Supplementary Table 8. Sequences and accession IDs of proteins characterized in this study. Bolded sequences indicate added purification tags.

Protein name	NCBI accession no.	Organism	Protein sequence (bold indicates added tag)	Coding sequence	Codon opt.?
DybA	WP_157488328.1	Dyadobacter crusticola strain DSM 16708	M**GSSHHHHHH**KIS<br>KPVLQAVAVAVALS<br>TITACADMDIKPEK<br>DKKAKTRTLDPCPA<br>CGMG	ATGGGCAGCAGCCATCACCATCATCACCACAAAATCTCAA<br>AACCAGTACTTCAGGCCGTTGCCGTTGCAGTTGCACTGAG<br>CACAATTACCGCTTGCGCCGACATGGATATTAAACCTGAA<br>AAGGATAAAAAAGCGAAGACGCGCACCCTGGATCCTTGC<br>CCGGCCTGCGGAATGGGTTGA	No
DybH	WP_031530742.1	Dyadobacter crusticola strain DSM 16708	MSQIFSTVACNLDA<br>HILQASIPLFEMERV<br>EAIEWSFDALFKVD<br>VIPEWFTDFIKEFSD<br>QNRLIGHGVYFSLF<br>SGKWSAEQQKWL<br>DQLSKLCDQFQFD<br>HITEHFGFMTGSDF<br>HKGAPLGIPFNAST<br>LAIGKDRLARIQNA<br>CNCPVGLENLAFSY<br>SLDEVKRHGDFLA<br>QLVESVNGFIILDLH<br>NLYCQCHNFELDFI<br>DFLSFYPLERVREM<br>HISGGSWDTQIPGR<br>VVRRDTHDEAVPS<br>EVFEYLRQAIPRCP<br>NLKFVVMEQLGTAL<br>DTAEKQAGFQKDF<br>LEMDRIVKSVGATL<br>PDSVKNFQPLSQQ<br>VQIGPPIEDLTLHQ<br>QQMQLSEILEKARD<br>LHEAKNLLLASDLS<br>NTDWHIERWEPHM<br>LQTAMNIAQKWKA<br>GF	ATGTCGCAAATATTCTCCACAGTCGCCTGTAACCTCGACG<br>CGCACATTCTGCAAGCGTCTATTCCATTGTTTGAAATGGA<br>GCGAGTGGAGGCTATTGAATGGTCTTTCGATGCGCTCTTC<br>AAAGTGGATGTGATACCGGAATGGTTTACTGATTTTATAAA<br>AGAATTCAGTGACCAGAACAGGCTGATTGGTCACGGCGTT<br>TACTTTTCGCTGTTTTCGGGAAAATGGTCGGCAGAGCAGC<br>AAAAATGGCTGGATCAGCTCAGCAAGCTCTGCGACCAGTT<br>TCAGTTTGATCACATCACGGAGCATTTCGGCTTTATGACC<br>GGTTCTGACTTCCACAAAGGCGCGCCGCTCGGCATTCCC<br>TTCAATGCATCTACATTAGCAATCGGAAAAGACAGGCTGG<br>CGCGCATTCAGAATGCGTGTAATTGTCCGGTTGGATTGGA<br>AAACCTGGCTTTTTCCTATTCACTGGATGAGGTAAAACGG<br>CATGGAGACTTTTTGGCCCAACTGGTGGAAAGTGTCAACG<br>GGTTCATCATCCTCGACCTGCACAACCTGTATTGCCAATG<br>CCATAATTTTGAGCTCGACTTCATCGATTTCCTTTCGTTCT<br>ATCCACTTGAACGCGTGCGGGAAATGCATATCTCCGGCG<br>GGAGCTGGGACACACAGATTCCGGGAAGGGTAGTACGGC<br>GCGACACCCACGACGAGGCAGTACCATCGGAAGTATTCG<br>AATACCTCAGGCAGGCAATCCCGCGCTGCCCTAACTTGAA<br>GTTTGTAGTAATGGAGCAGCTGGGCACAGCACTCGATACA<br>GCTGAAAAGCAGGCTGGGTTTCAAAAGGACTTTCTTGAAA<br>TGGACCGCATTGTCAAATCCGTCGGAGCAACCCTGCCCG<br>ATTCAGTAAAGAACTTTCAGCCTCTTTCACAACAGGTGCAA<br>ATTGGCCCGCCGATCGAAGACCTTACTTTGCACCAGCAG<br>CAAATGCAATTGTCGGAAATCCTGGAAAAAGCACGGGACC<br>TGCATGAGGCAAAAAACCTCTTGCTTGCGTCTGACCTCAG<br>CAATACAGACTGGCACATTGAACGTTGGGAGCCGCACAT<br>GCTGCAAACAGCGATGAACATTGCGCAGAAATGGAAGGC<br>AGGATTTTGA	No
ChrA	WP_034727518.1	Chryseobacterium sp. JM1	M**GSSHHHHHHSQ**<br>MKIPALVMASLLAV<br>SVSGQTTKPVKKG<br>TKSVKKVKKMDSP<br>KTVKAEPTKVVKRD<br>TILKHGGGCPACG<br>MG	ATGGGCAGCAGCCATCACCATCATCACCACAGCCAGATG<br>AAGATTCCGGCTCTGGTGATGGCCAGCCTGCTGGCGGTA<br>TCAGTGAGCGGGCAAACCACGAAACCGGTCAAAAAAGGC<br>ACGAAAAGTGTTAAAAAAGTGAAAAAAATGGATAGCCCAA<br>AAACCGTTAAAGCAGAACCCACCAAAGTCGTTAAACGTGA<br>CACGATCCTGAAACATGGCGGCGGTTGTCCGGCGTGCGG<br>TATGGGGTGA	Yes
ChrH	WP_034727520.1	Chryseobacterium sp. JM1	MKKPLLGLAMMPE<br>ADFVSAILPLLQTQ<br>SVDVLEWSFDTLY<br>DVEEPEWLSGLLD<br>FYSDNSRLLGHGV<br>YYSLFDARWMERQ<br>EIWLKKLKEEVKRR<br>KYNHITEHFGFMNT<br>ENFHQGVPLPVPLL<br>PKTLQIGKDRLSRL<br>QDAVEIPVGVENLA<br>FSFSMDDVKEQGE<br>FLDRLVEDIDGFLIL<br>DLHNIYCQSCNFKV<br>DMMEIINLYPLEKV<br>NEIHLSGGSWQES<br>AYGKKPVRRDTHD<br>DRIPEEILNLLPEVLI<br>QCHPEYVIIERLGH<br>TLNTEVAKQIFFDD<br>FNRVKKILELSDYP<br>VGEEKIWNRQNTV<br>HSKPVEDLLLYAEQ	ATGAAGAAACCTCTCCTTGGATTAGCCATGATGCCGGAAG<br>CTGACTTTGTTTCCGCTATTCTTCCGCTTTTGCAAACGCAA<br>AGTGTTGACGTACTCGAGTGGAGCTTCGATACGCTGTATG<br>ACGTCGAAGAGCCGGAATGGCTGTCCGGACTGCTTGACT<br>TCTATTCAGACAATTCACGCCTGTTGGGACACGGGGTGTA<br>CTATTCATTATTCGACGCCCGCTGGATGGAGCGCCAAGA<br>GATTTGGCTCAAGAAGCTTAAAGAAGAGGTGAAGCGCCG<br>CAAGTACAATCATATCACCGAGCATTTTGGCTTCATGAACA<br>CTGAGAATTTCCACCAAGGCGTACCTCTTCCTGTACCACT<br>TCTGCCGAAGACGCTGCAAATTGGGAAGGATCGCCTGTC<br>TCGTCTCCAGGACGCCGTGGAGATCCCGGTGGGTGTCGA<br>GAATCTGGCATTCTCTTTCAGCATGGATGACGTCAAGGAG<br>CAAGGTGAATTCTTGGACCGTTTGGTGGAAGACATCGACG<br>GGTTCCTCATCCTCGATCTCCACAATATTTACTGCCAGAG<br>CTGCAACTTCAAGGTGGACATGATGGAGATCATCAATTTA<br>TATCCGCTCGAGAAGGTCAACGAGATCCACCTCTCCGGC<br>GGCTCTTGGCAAGAGTCGGCATACGGCAAGAAACCTGTG<br>CGCCGTGATACGCATGACGACCGTATCCCTGAAGAGATT<br>CTGAATTTATTACCCGAGGTTTTAATTCAGTGCCATCCTGA<br>GTACGTAATTATTGAACGCTTAGGCCACACACTTAATACA<br>GAGGTGGCCAAGCAAATTTTCTTCGATGACTTTAACCGCG<br>TAAAGAAAATCTTGGAGTTATCAGATTACCCAGTCGGAGA<br>AGAGAAAATTTGGAATCGCCAGAATACCGTCCACTCGAAG	Yes

			TELTRLLFDGNTTESIKNRDFHYFKPESWDEEMITTAQQIIKKWNPY	CCTGTTGAAGATCTCCTCTTGTATGCTGAGCAAACTGAATTAACCCGTTTACTTTTCGATGGTAATACGACGGAATCTATTAAGAATCGCGATTTTCATTATTTCAAGCCTGAATCCTGGGACGAGGAGATGATCACAACTGCGCAACAGATCATTAAGAAGTGGAACCCGTACTAA	
ChsA	WP_072362158.1	Chitinophaga sancti strain DSM 784	M**HHHHHH**KLSKSLLSAIMIGIAVQTTVVSCSKDEQVKPKKADQANKQSESKPVDNPDSCPACGMG	ATGCATCATCATCATCATCATAAACTGAGCAAAAGCCTGCTGAGCGCGATTATGATTGGTATTGCGGTTCAGACCACCGTGGTGAGCTGTAGCAAAGATGAACAGGTGAAACCGAAAAAAGCGGATCAGGCGAACAAACAGAGCGAAAGCAAACCGGTGGATAATCCGGATAGCTGCCCGGCGTGCGGCATGGGCTAA	Yes
ChsH	WP_072362160.1	Chitinophaga sancti strain DSM 784	MPKILSAVACNLDANILAACLPLFEESRVEAIEWSFDALYKVKEVPGWFRELLSAFSDENRLIGHGVFFSLFSGKWLPEQEAWLSHLQQTATEFSFDHITEHFGFMTGEDFHHGAPLNIPYSSSTLNIGKDRLKRIYHACGRPVGLENLAFSYSLDEVKRHGTFLEQLLEPVNGFIILDLHNLFCQLKNFDLDFNTLISLYPLDKVREIHISGGSWDDSAAAPDRSIRRDTHDESVPPEVFQLLEMTIPQCPNLKYVVLEQLGNGLQSTASKQGFYNDFLQMQEIAQQHNYKEGNPFLPLESLPIITGAAIEDLRLYQQQLELSAILENAGTYAEAMQQLQQSSLAHSDWKIEEWEPYMIETAVKIARKWKK	ATGCCGAAAATTCTGAGCGCGGTGGCCTGCAATCTGGATGCAAACATTCTGGCGGCGTGCCTGCCGCTGTTTGAAGAAAGCCGCGTTGAAGCCATTGAATGGAGCTTTGATGCCCTGTATAAAGTGAAAGAAGTGCCGGGCTGGTTTCGCGAACTGCTGAGCGCCTTTAGCGATGAAAACCGCCTGATTGGCCATGGCGTGTTTTTTTCACTGTTTTCGGGCAAATGGCTGCCGGAACAGGAAGCGTGGCTGAGCCATCTGCAGCAGACCGCGACCGAATTTAGCTTCGATCACATCACCGAACATTTCGGCTTCATGACCGGCGAAGATTTCCACCATGGCGCCCCGCTGAATATCCCGTACAGCAGCAGCACCCTGAACATTGGCAAAGATCGTCTGAAACGCATTTATCATGCGTGCGGTCGCCCGGTTGGCCTGGAAAATCTGGCCTTCAGCTATAGCCTGGATGAAGTGAAACGCCATGGCACCTTTCTGGAACAGCTGCTGGAACCGGTGAACGGCTTCATTATTCTGGATCTGCATAACCTGTTTTGTCAGCTGAAAAATTTTGATCTGGATTTTAATACCCTGATTTCACTGTATCCGCTGGATAAAGTGCGTGAAATTCATATTAGCGGCGGTAGCTGGGATGATAGCGCGGCGGCGCCGGATCGTAGCATTCGCCGCGATACCCACGATGAAAGCGTTCCGCCGGAGGTTTTTCAGCTGCTGGAAATGACCATTCCACAGTGCCCGAACCTGAAATATGTGGTGCTGGAACAGCTGGGCAACGGCCTGCAGTCTACCGCGTCCAAACAGGGCTTCTATAACGATTTTCTGCAGATGCAGGAAATTGCCCAGCAGCACAATTATAAAGAAGGCAACCCGTTCCTGCCGCTGGAAAGCCTGCCGATTATTACCGGCGCGGCGATTGAAGATCTGCGTCTGTATCAGCAGCAGCTGGAGCTGTCAGCGATTCTGGAAAATGCGGGTACCTATGCGGAAGCCATGCAGCAGCTGCAGCAGTCGAGCCTGGCCCATAGCGATTGGAAAATTGAAGAATGGGAACCGTACATGATTGAAACCGCGGTGAAAATTGCGCGCAAATGGAAAAAATAA	Yes
HymA	WP_170934670.1	Hymenobacter gelipurpurascens strain DSM 11116	M**HHHHHH**KLSQAILGAVLVGLTAQTTGCIKKSDPTPKEEQGKSGKKSPEIPYNCPGCGLG	ATGCATCATCATCATCATCATAAGCTCAGCCAAGCCATATTAGGCGCAGTATTAGTGGGTCTGACAGCTCAAACAACCGGTTGTATAAAGAAGTCTGATCCTACTCCAAAGGAAGAACAAGGAAAGAGCGGTAAGAAGAGCCCGGAGATTCCGTACAATTGTCCAGGATGCGGACTGGGGTAA	Yes
HymH	WP_088841642.1	Hymenobacter gelipurpurascens strain DSM 11116	MRTVAPPDNANERAIYSSIACNLDANILTAAFPLLEQGKVEALEWSFDALFWAEQVPDWFTELLQAYSAQHRLVGHGVYFSLLSGRWTAEQQQWLQHLKELTRRFSFDHVTEHFGFFTGQNFHAGAPLPIPYGSNTLRIGQDRLRRMQEACGCPVGLENLAFAYSLEEVKRHGEFLEQLVEPVNGFLILDLHNVFCQLHNFSVPYEELMALYPLDRVREIHISGGSWEASGQVPGLQIRRDTHDEAVPEEVFQLLYNTMPRCPNLKYVVLEQLGTGLQTEDSRAHFQSDFHRMQALVNQHRSTTSIRAVQPFLPIHSAVAGPVAEDAQLHEQQQQLSHILETAPSYEDAQRQ	ATGAGAACTGTGGCTCCACCGGATAACGCTAACGAACGTGCGATTTATTCATCAATCGCGTGTAATCTTGATGCGAATATCCTCACCGCTGCGTTCCCTCTGCTTGAACAAGGCAAAGTTGAAGCGTTAGAGTGGTCATTCGACGCACTTTTCTGGGCAGAACAGGTTCCAGACTGGTTCACAGAGCTCTTACAAGCATATTCGGCCCAGCACCGCTTAGTAGGTCACGGTGTTTATTTTAGCTTATTAAGCGGCAGATGGACCGCGGAACAACAACAATGGCTCCAGCATCTTAAAGAATTGACACGACGTTTCAGTTTTGATCATGTAACCGAACATTTCGGTTTCTTCACAGGGCAAAACTTCCACGCAGGTGCCCCATTGCCGATTCCCTACGGATCTAATACGCTCCGTATCGGCCAGGATCGATTACGACGCATGCAAGAAGCATGTGGTTGCCCAGTCGGGCTGGAGAATTTAGCATTCGCATACAGCCTGGAAGAGGTTAAGCGCCATGGTGAATTCTTGGAGCAATTGGTTGAACCTGTCAATGGGTTCTTGATCTTGGATTTACATAATGTCTTTTGTCAACTGCATAATTTTAGCGTACCTTATGAGGAATTGATGGCTTTGTACCCATTAGACCGTGTCCGTGAGATACACATCAGCGGCGGATCATGGGAAGCTAGTGGCCAAGTTCCCGGGCTGCAAATACGACGTGACACGCACGACGAAGCAGTTCCTGAGGAAGTTTTCCAACTTTTGTATAATACGATGCCTCGTTGCCCGAATCTTAAGTACGTCGTATTAGAACAACTCGGCACCGGTCTGCAAACAGAAGATTCAAGAGCGCATTTTCAATCAGATTTCCACCGAATGCAAGCTTTAGTAAATCAGCATCGTAGTACAACATCCATACGAGCGGTGCAACCCTTCTTGCCGATTCACAGCGCCGTAGCTGGGCCTGTGGCAGAGGACGCACAACTTCATGAACAACAACAACAACTGTCTCATATTCTTGAGACCGCACCTTCTTATGA	

			LQASALAYTAWKV<br>EEWEPHMLETAISI<br>AQKWKPQPR	GGATGCACAACGCCAACTGCAAGCCTCGGCTCTCGCGTA<br>TACAGCCTGGAAGGTAGAAGAATGGGAGCCGCACATGCT<br>TGAGACTGCTATTTCCATCGCACAAAAGTGGAAACCACAA<br>CCACGTTGA	
ArlA	WP_25744<br>8965.1	Archangium<br>lipolyticum strain<br>CY-1	M**GSSHHHHHH**NQ<br>NLREKATQLLDVAS<br>NTQGMKSSSPLPC<br>PACGMMMALPESS<br>PQLVTPATR	ATGGGCAGCAGCCATCACCATCATCACCACAACCAGAATC<br>TCCGTGAGAAGGCCACCCAGCTCCTCGATGTGGCGAGCA<br>ACACCCAGGGAATGAAGTCGAGCAGTCCTCTTCCCTGTC<br>CGGCCTGCGGCATGATGATGGCGCTGCCCGAGTCCAGCC<br>CCCAACTCGTGACCCCGGCCACCCGCTGA	No
ArlH	WP_25744<br>8967.1	Archangium<br>lipolyticum strain<br>CY-1	MPPRVGLNLLTDD<br>AFREAVRPLFAEGL<br>VAALEWDIDDSWG<br>FSTRALPGWTERM<br>LDLYAEEGALYGH<br>GVWLSVLTAAWQP<br>RQEAWIERLAQEC<br>RRRRYRHVSEHFG<br>FTAAGPFTRSTMLP<br>LPYCTAAVDIGRDR<br>LERLRAATGGPVGL<br>EVLANTLAPVDALH<br>QGPFLDAVLTPTEG<br>FLVLDVHNVWTQA<br>VNTGLPPELLLETY<br>PLECVREIHLSGGS<br>WGRALGAGDSRPV<br>RLDSHDGPLLSPVL<br>SLLRRALALCPHCE<br>VVIVERRSETLESE<br>EVRAGWRAQYRAV<br>VQLVAEASATPVSP<br>VRVSASAPPPELLD<br>TEELARYQRELVTA<br>LVEESESRVILQRL<br>RQGIAGARLAPYLE<br>TFDPRMVELLAILA<br>SRWSAFDPARATP	ATGCCGCCTCGCGTGGGTCTGAATCTTCTGACCGACGAC<br>GCCTTCCGCGAGGCGGTACGGCCGCTCTTCGCCGAGGG<br>GCTGGTCGCCGCGCTCGAGTGGGACATCGACGATTCCTG<br>GGGATTCTCCACCCGTGCACTCCCCGGCTGGACCGAGCG<br>TATGCTCGACCTCTATGCGGAGGAGGGAGCCTTGTACGG<br>CCACGGCGTGTGGCTGTCGGTGCTGACCGCCGCCTGGC<br>AGCCTCGGCAGGAGGCGTGGATCGAGCGTCTCGCCCAG<br>GAGTGCCGCCGCCGCCGCTACCGCCACGTCTCCGAGCA<br>CTTCGGCTTCACCGCCGCGGGGCCGTTCACCCGCAGCAC<br>CATGCTGCCCCTGCCGTATTGCACCGCCGCCGTGGACAT<br>CGGCCGCGACCGGCTCGAACGGCTGCGCGCCGCCACCG<br>GCGGCCCCGTCGGCCTCGAGGTGCTGGCCAACACCCTC<br>GCCCCCGTCGATGCGCTCCACCAAGGTCCGTTCCTCGAT<br>GCCGTGCTGACCCCCACCGAGGGCTTCCTGGTGCTCGAC<br>GTCCACAACGTGTGGACCCAGGCGGTCAACACCGGCCTG<br>CCTCCAGAGCTCCTGCTCGAGACCTATCCGCTGGAGTGC<br>GTCCGGGAAATCCACCTCTCGGGTGGGAGCTGGGGCCG<br>CGCGCTCGGCGCCGGGGACTCCCGTCCCGTCCGGCTCG<br>ACAGTCACGACGGACCCCTCCTGTCACCCGTGCTGTCCC<br>TGCTCCGCCGGGCGCTCGCCCTCTGCCCTCATTGCGAGG<br>TCGTCATCGTCGAGCGGCGAAGCGAGACCCTCGAGTCAG<br>AGGAAGTGCGCGCCGGGTGGCGCGCCCAGTACCGCGCC<br>GTGGTCCAGCTGGTGGCCGAGGCCAGCGCAACCCCCGT<br>CAGCCCGGTCCGTGTCTCCGCCTCCGCGCCGCCCCCCG<br>AGCTGCTCGACACGGAGGAGCTCGCCCGGTACCAGCGC<br>GAGCTGGTCACGGCCCTCGTCGAGGAGTCCGAGAGCCG<br>GGTTATCCTGCAGCGTCTGCGCCAGGGTATCGCCGGCGC<br>ACGGCTCGCGCCCTACCTCGAGACCTTCGATCCGCGCAT<br>GGTCGAGCTGCTGGCCATCCTCGCGTCACGCTGGTCCGC<br>CTTCGACCCCGCTCGAGCCACTCCCTGA	No
MelA1	MCC6978<br>278.1	Candidatus<br>Melainabacteria<br>bacterium isolate<br>SJ512	M**GSSHHHHHHSQ**<br>**M**KNKSAKVLTSLFA<br>LGLTTGAVPARADK<br>AAVETAEASVKMFE<br>QRSGTNNSSYTSLL<br>MYLAGVYLANDRE<br>KDAEIIYNKAIFNLK<br>SKPDRKAEVPALML<br>NWAMLLASNKGAS<br>KEKAEKALSDGLKL<br>ANDLPAASKERINY<br>LIGTINFYNVIGKPA<br>EKQARIKAADEHLA<br>TLEKNEKLNNEEIT<br>NVAANLVKLAEIQT<br>FPMPVMRLRYQPP<br>VFQVVPDNSPDKP<br>NTVRAKEFKSAEAF<br>QLRAIKQYDRLPET<br>VPWRIEAHRKLILW<br>YRSLGQTKQEEFQI<br>QQLGKIMHTTDRD<br>KLFPQPAPCPACG<br>MG	ATGGGCAGCAGCCATCACCATCATCACCACAGCCAGATG<br>AAAAACAAATCAGCCAAAGTTCTAACTTCGCTATTCGCGCT<br>CGGTCTGACCACTGGCGCCGTGCCCGCGCGCAGATA<br>AAGCAGCGGTGGAAACAGCCGAAGCAAGCGTAAAAATGT<br>TCGAGCAACGCTCAGGCACGAACAATTCGTCTTATACCAG<br>CCTACTGATGTACCTGGCTGGGGTATATCTGGCGAACGAC<br>AGAGAGAAAGACGCTGAGATCATATACAACAAAGCAATCT<br>TCAATCTCAAATCGAAACCGGATAGGAAAGCTGAAGTGCC<br>CGCCTTGATGCTGAACTGGGCAATGCTGCTGGCATCGAA<br>CAAAGGTGCATCTAAAGAAAAAGCGGAAAAAGCATTGTCG<br>GATGGACTGAAACTCGCAAACGACTTGCCGGCCGCCTCC<br>AAAGAGCGGATCAATTATCTGATAGGAACAATCAATTTCTA<br>CAACGTAATTGGCAAACCTGCCGAGAAGCAAGCCCGCAT<br>CAAAGCAGCCGATGAGCATCTTGCCACGCTGGAGAAAAA<br>CGAAAAACTAAACAATGAAGAAATCACCAATGTCGCAGCG<br>AATCTAGTGAAACTGGCAGAGATTCAAACGTTTCCCATGC<br>CGGTCATGCGTTTGCGCTACCAGCCTCCGGTGTTTCAAGT<br>AGTGCCGGACAACTCGCCGGACAAACCGAACACAGTGCG<br>AGCAAAGGAGTTCAAGTCGGCGGAAGCTTTCCAATTGCG<br>CGCGATAAAACAATACGATCGTTTGCCGGAAACGGTGCC<br>CTGGCGCATCGAAGCGCACAGAAAGTTGATTCTCTGGTAT<br>CGCAGCCTGGGTCAGACGAAACAGGAAGAGTTTCAAATT<br>CAGCAGTTAGGCAAAATCATGCACACTACAGATCGCGACA<br>AACTGTTTCCCCAGCCGGCACCCTGCCCGGCTTGCGGCA<br>TGGGTTAG	No

MelH	MCC6978279.1	Candidatus Melainabacteria bacterium isolate SJ512	MQKSKPKLGLSLMPTEDFQMATEQLFEEERVESLEWSFDFTWNGVVVDPWALELIDKYSEANALTGHGVNLSPLSARFSKRQEEWLARAREEFKTRKYVHASEHFGFSEAGPIKHGAPLSVPMDAASLKVGKEMLKRYADATGCPVGLENLAFAFSINDVKKQGHFIDELISSVNGFLLLDLHNIFCQIANFGISELELLNSYPLDKVREIHISGGSWSPSISGKRAAVRRDTHDDGVPQEVFNLAALALKLCPNIEFVIFERLGNTMFSLEAQSEFRDDFESMEQILEFCYA	ATGCCCACCGAGGACTTCCAAATGGCAACGGAACAGTTATTCGAGGAAGAGCGCGTGGAGAGCTTGGAGTGGTCGTTCGACTTTACGTGGAATGGAGTCGTTGTGGACCCCTGGGCATTAGAGCTGATCGATAAATACAGCGAGGCTAACGCTCTTACGGGACACGGTGTTAATCTGTCCCCGCTGTCTGCCCGTTTCTCTAAGAGACAAGAGGAGTGGCTCGCTCGTGCTCGCGAGGAATTCAAGACCCGCAAATACGTGCACGCTTCTGAACACTTTGGGTTCTCCGAGGCTGGTCCTATCAAGCACGGAGCTCCCCTGTCTGTTCCGATGGACGCGGCCAGCCTTAAGGTTGGTAAGGAAATGTTAAAGAGATATGCCGACGCTACCGGGTGTCCCGTTGGACTTGAAAATCTGGCGTTCGCTTTCTCTATAAACGACGTTAAGAAACAAGGTCACTTCATTGATGAGCTGATCAGTTCTGTAAACGGATTCCTGTTATTGGACTTACATAACATTTTCTGCCAAATTGCCAATTTCGGAATAAGTGAGCTGGAGCTTCTTAATAGCTATCCGTTAGATAAGGTCCGGGAGATACATATATCTGGTGGTTCATGGTCACCCAGTATTTCTGGTAAACGTGCGGCCGTGAGAAGAGATACCCACGATGACGGGGTGCCTCAGGAAGTGTTTAACCTGGCAGCGTTGGCGTTGAAACTGTGTCCGAACATCGAGTTTGTGATATTCGAGCGACTTGGAAACACTATGTTTTCTTTAGAGGCACAGTCCGAATTCCGGGATGACTTTGAATCAATGGAACAAATTCTTGAATTCTGTTACGCGTAA	Yes
MelH$_c$	MCC6978280.1	Candidatus Melainabacteria bacterium isolate SJ512	MPDTLKLQEFQDALNELLASGKSHAEIMEELNSNPQFESYREYISEFDPDMVEVARELMGKWAQRVGE	ATGCCCGACACTCTGAAGCTCCAGGAATTTCAGGATGCATTGAACGAACTCCTCGCTTCGGGTAAGAGCCACGCGGAGATTATGGAAGAGTTAAATAGTAACCCGCAGTTCGAGTCATACCGGGAGTATATATCGGAGTTTGACCCCGACATGGTGGAAGTTGCCCGAGAACTCATGGGTAAATGGGCCCAACGGGTAGGTGAATGA	Yes
eGFP-CPACGMG-10 aa linker	-	N/A	M**GSSHHHHHHSSGLVPRGSHMASM**VSKGEELFTGVVPILVELDGDVNGHKFSVSGEGEGDATYGKLTLKFICTTGKLPVPWPTLVTTLTYGVQCFSRYPDHMKQHDFFKSAMPEGYVQERTIFFKDDGNYKTRAEVKFEGDTLVNRIELKGIDFKEDGNILGHKLEYNYNSHNVYIMADKQKNGIKVNFKIRHNIEDGSVQLADHYQQNTPIGDGPVLLPDNHYLSTQSALSKDPNEKRDHMVLLEFVTAAGITLGMDELYKGSAGSAAGSGCPACGMG	ATGGGCAGCAGCCATCATCATCATCATCACAGCAGCGGCCTGGTGCCGCGCGGCAGCCATATGGCTAGCATGGTGAGCAAGGGCGAGGAGCTGTTCACCGGGGTGGTGCCCATCCTGGTCGAGCTGGACGGCGACGTAAACGGCCACAAGTTCAGCGTGTCCGGCGAGGGCGAGGGCGATGCCACCTACGGCAAGCTGACCCTGAAGTTCATCTGCACCACCGGCAAGCTGCCCGTGCCCTGGCCCACCCTCGTGACCACCCTGACCTACGGCGTGCAGTGCTTCAGCCGCTACCCCGACCACATGAAGCAGCACGACTTCTTCAAGTCCGCCATGCCCGAAGGCTACGTCCAGGAGCGCACCATCTTCTTCAAGGACGACGGCAACTACAAGACCCGCGCCGAGGTGAAGTTCGAGGGCGACACCCTGGTGAACCGCATCGAGCTGAAGGGCATCGACTTCAAGGAGGACGGCAACATCCTGGGGCACAAGCTGGAGTACAACTACAACAGCCACAACGTCTATATCATGGCCGACAAGCAGAAGAACGGCATCAAGGTGAACTTCAAGATCCGCCACAACATCGAGGACGGCAGCGTGCAGCTCGCCGACCACTACCAGCAGAACACCCCCATCGGCGACGGCCCCGTGCTGCTGCCCGACAACCACTACCTGAGCACCCAGTCCGCCCTGAGCAAAGACCCCAACGAGAAGCGCGATCACATGGTCCTGCTGGAGTTCGTGACCGCCGCCGGGATCACTCTCGGCATGGACGAGCTGTACAAGGGATCCGCTGGCTCCGCTGCTGGTTCTGGCTGCCCGGCCTGCGGAATGGGTTAA	Yes
eGFP-CPACGMG-4 aa linker	-	N/A	M**GSSHHHHHHSSGLVPRGSHMASM**VSKGEELFTGVVPILVELDGDVNGHKFSVSGEGEGDATYGKLTLKFICTTGKLPVPWPTLVTTLTYGVQCFSRYPDHMKQHDFFKSAMPEGYVQERTIFFKDDGNYKTRAEVKFEGDTLVNRIELKGIDFKEDGNILGHKLEYNYNSHNVYIMADKQKNGIKVNFKIRHNIEDGSVQLADHYQQNTPIGDGPVLLPDNHYLSTQSALSKDPNEKRDHMVLLEFVTAAGITLG	ATGGGCAGCAGCCATCATCATCATCATCACAGCAGCGGCCTGGTGCCGCGCGGCAGCCATATGGCTAGCATGGTGAGCAAGGGCGAGGAGCTGTTCACCGGGGTGGTGCCCATCCTGGTCGAGCTGGACGGCGACGTAAACGGCCACAAGTTCAGCGTGTCCGGCGAGGGCGAGGGCGATGCCACCTACGGCAAGCTGACCCTGAAGTTCATCTGCACCACCGGCAAGCTGCCCGTGCCCTGGCCCACCCTCGTGACCACCCTGACCTACGGCGTGCAGTGCTTCAGCCGCTACCCCGACCACATGAAGCAGCACGACTTCTTCAAGTCCGCCATGCCCGAAGGCTACGTCCAGGAGCGCACCATCTTCTTCAAGGACGACGGCAACTACAAGACCCGCGCCGAGGTGAAGTTCGAGGGCGACACCCTGGTGAACCGCATCGAGCTGAAGGGCATCGACTTCAAGGAGGACGGCAACATCCTGGGGCACAAGCTGGAGTACAACTACAACAGCCACAACGTCTATATCATGGCCGACAAGCAGAAGAACGGCATCAAGGTGAACTTCAAGATCCGCCACAACATCGAGGACGGCAGCGTGCAGCTCGCCGACCACTACCAGCAGAACACCCCCATCGGCGACGGCCCCGTGCTGCTGCCCGACAACCACTACCTGAGCACCCAGTCCGCCCTGAGCAAAGACCCCAACGAGAAGCGCGATCACATGGTCCTGCTGGAGTTCGTGACCGCCGCCGGGATCACTCTCGGCATGGACGAGCTGT	Yes

			MDELYKGSAGCPA<br>CGMG	ACAAGGGATCCGCTGGCTGCCCGGCCTGCGGAATGGGTT<br>AA	
NedA	WP_08536<br>5104.1	Neisseria dentiae<br>strain CCUG<br>53898 310003	MKTTSTLMALAGAL<br>VLAGCQVEPAQSS<br>SPEVSVQQKAGEG<br>KCGAASQAGKAAE<br>GKCGEGKCGSK**HH**<br>**HHHH**	ATGAAAACCACGTCGACCCTGATGGCACTGGCAGGGGCG<br>CTGGTGCTGGCGGGCTGCCAAGTCGAACCAGCGCAGTC<br>GTCCAGTCCTGAGGTTAGCGTGCAGCAGAAAGCAGGCGA<br>AGGCAAATGCGGCGCAGCCTCGCAGGCGGGTAAGGCGG<br>CGGAGGGAAAATGTGGTGAAGGTAAATGCGGCTCGAAGC<br>ATCACCATCATCACCACTGA	Yes
NedB	WP_08536<br>5102.1	Neisseria dentiae<br>strain CCUG<br>53898 310003	MNTLHGAGLGYKR<br>SMAADFLQLDRNN<br>SPIRFIEIAPENWLR<br>MGGAARKQFDEVA<br>ERFPVACHGLSLSL<br>GGQDPLQLDFLKQI<br>KAFLRQYRIGFFSE<br>HLSYCSHHGHIYDL<br>LPLPFTEESVRHTA<br>ARIRAVQDILEMRIA<br>VENTSYYAHNPIAE<br>MDEAEFLNAVVRE<br>ADCDIHLDINNIYVN<br>AVNHGIVAPRDYID<br>RTDLARVSYMHMA<br>GHDEKTENLLIDTH<br>GQPVCDDVWDLFA<br>YACRRLPHSVPTLL<br>ERDSNFPPFAELEA<br>EVARIAAIQQQAEK<br>ERHAAA	ATGAACACTCTGCAcGGCGCTGGGCTGGGTTATAAACGTT<br>CTATGGCAGCAGATTTCCTGCAGCTGGATCGCAACAATTC<br>TCCGATTCGCTTTATCGAGATTGCCCCGGAAAATTGGTTG<br>CGTATGGGCGGCGCGGCCCGCAAGCAATTCGACGAAGT<br>GGCCGAGCGTTTTCCGGTGGCTTGCCAcGGTCTGAGCCT<br>GTCCCTGGGAGGCCAGGACCCGTTACAACTGGATTTCCT<br>GAAACAGATCAAAGCGTTTTTGCGCCAGTACCGtATCGGT<br>TTCTTTTCAGAACATCTGTCCTACTGTTCCCACCAcGGACA<br>CATCTACGATCTGCTGCCGCTTCCGTTCACGGAAGAGTCG<br>GTACGTCACACAGCTGCCCGCATTCGCGCGGTGCAGGAC<br>ATTTTGGAGATGCGTATCGCCGTGGAAAATACATCTTACT<br>ATGCACACAATCCCATCGCGGAGATGGATGAAGCGGAGT<br>TCCTGAACGCCGTGGTGCGTGAGGCGGATTGCGACATCC<br>ACTTAGATATTAATAATATTTACGTGAATGCCGTCAACCAC<br>GGCATCGTGGCCCCACGCGACTACATTGACCGCACGGAT<br>CTGGCCCGTGTGAGTTATATGCACATGGCAGGCCATGAC<br>GAGAAGACTGAGAATTTGTTAATCGACACCCACGGCCAAC<br>CGGTTTGTGATGACGTGTGGGACTTATTTGCCTACGCGTG<br>TCGCCGCCTGCCTCACTCTGTGCCAACCTTACTGGAACGC<br>GATAGCAATTTTCCGCCTTTTGCCGAATTAGAAGCCGAGG<br>TAGCTCGCATCGCCGCTATCCAACAGCAGGCCGAGAAAG<br>AACGTCATGCGGCGGCGTAA	Yes
NedC	WP_08536<br>5101.1	Neisseria dentiae<br>strain CCUG<br>53898 310003	MQPHNPSPSARAQ<br>AELADHVRNPALPA<br>PAGIAPERLAVYTR<br>LVRNNLKSFLDLCF<br>SDSSLMLDPAQWQ<br>GWQNRFLIEARPE<br>SPFFNDIPAQFLAY<br>LNRLPEHDRPSENI<br>LAMMDFETALLHAE<br>TARQPDSDGRWSE<br>HSVLSWAPAARLQ<br>QYPCDFVSSGLAQI<br>NAGACHVLSWRNR<br>RNEVYYRIVEDTDL<br>FLLQHFQSQNDTFA<br>RLLESLQSLLPGQD<br>IEGRLKTAVGGWV<br>EAGVLLTAE	ATGCAACCGCATAACCCAAGTCCGTCGGCGCGTGCGCAA<br>GCCGAGCTGGCAGATCATGTTCGCAACCCTGCACTGCCC<br>GCCCCGGCGGGGATTGCACCGGAACGCCTGGCGGTATA<br>TACCCGCCTGGTGCGTAACAATCTTAAATCGTTCCTGGAT<br>CTGTGCTTCTCTGATTCCAGCCTGATGCTGGATCCCGCGC<br>AATGGCAGGGCTGGCAGAATCGCTTTCTGATCGAGGCAC<br>GCCCCGAAAGCCCGTTTTTTAATGATATTCCGGCGCAGTT<br>TCTTGCcTATTTAAACCGTTTGCCTGAACATGATCGTCCGT<br>CAGAGAACATTCTTGCGATGATGGATTTCGAAACCGCTCT<br>GTTGCATGCGGAGACGGCGCGCCAGCCTGATTCTGATGG<br>CCGTTGGAGCGAGCATAGCGTCCTGAGCTGGGCCCCGG<br>CCGCACGTCTGCAGCAATATCCGTGTGATTTCGTGAGCTC<br>CGGCCTGGCACAGATCAAcGCGGGCGCGTGTCACGTTCT<br>GTCCTGGCGTAACCGTCGCAACGAAGTCTATTATCGTATC<br>GTGGAAGATACCGATCTGTTTCTGCTGCAGCAcTTTCAGT<br>CACAGAACGACACTTTTGCGCGCCTGCTGGAAAGCCTGC<br>AGTCCTTATTACCGGGTCAAGATATTGAAGGTCGTCTTAA<br>AACGGCCGTCGGTGGGTGGGTTGAAGCCGGCGTGCTGC<br>TGACCGCGGAGTAA	Yes

Supplementary Table 9. Primers and gblocks used in this study.

Primer/gblock name	Sequence (5'-3')	Purpose
pACYC:DybA insert F	ATCACCACAAAATCTCAAAACCAGTACTTCAGGCC	For cloning DybA into pACYCDuet
pACYC:DybA insert R	GCAAGCTTTCAACCCATTCCGCAGGCC	For cloning DybA into pACYCDuet
pACYC:DybA plasmid F	AATGGGTTGAAAGCTTGCGGCCGCATAATG	For cloning DybA into pACYCDuet
pACYC:DybA plasmid R	GTTTTGAGATTTTGTGGTGATGATGGTGATGGCTGC	For cloning DybA into pACYCDuet
pRSF:DybAH insert F	ATCACCACAAAATCTCAAAACCAGTACTTCAGGCC	For cloning DybA and DybH into pRSFDuet
pRSF:DybAH insert R	AGCCTAGGTTAATTATCAAAATCCTGCCTTCCATTTCTGCG	For cloning DybA and DybH into pRSFDuet
pRSF:DybAH plasmid F	GATTTTGATAATTAACCTAGGCTGCTGCCACCGC	For cloning DybA and DybH into pRSFDuet
pRSF:DybAH plasmid R	GTTTTGAGATTTTGTGGTGATGATGGTGATGGCTGC	For cloning DybA and DybH into pRSFDuet
pACYC:ChsA/HymA plasmid F	AACAGAAAGTAATCGTATTGTACACG	For cloning precursor peptides into pACYCDuet
pACYC:ChsA/HymA plasmid R	ATTTCCTAATGCAGGAGTCGCATAAG	For cloning precursor peptides into pACYCDuet
pACYC:MelA1 plasmid F	AGCTTGCGGCCGCATAATGCTTA	For cloning precursor protein into pACYCDuet
pACYC:MelA1 plasmid R	CTGCTGCCCATGGTATATCTCCTTATTAAAG	For cloning precursor protein into pACYCDuet
pRSF:ArlAH plasmid F	CAATAACTAGCATAACCCCTTGGGG	For cloning in ArlA and ArlH gblock into pRSFDuet
pRSF:ArlAH plasmid R	TATCTCCTTATTAAAGTTAAACAAAATTATTTCTACAGG	For cloning in ArlA and ArlH gblock into pRSFDuet
pCDF:DybH insert F	TTTAATAAGGAGATATACATGTCGCAAATATTCTCCACAGTCG	For cloning modifying enzyme into pCDFDuet
pCDF:DybH insert R	CGCAAGCTTTCAAAATCCTGCCTTCCATTTCTGC	For cloning modifying enzyme into pCDFDuet
pCDF:DybH plasmid F	CAGGATTTTGAAAGCTTGCGGCCGCATAATGC	For cloning modifying enzyme into pCDFDuet
pCDF:DybH plasmid R	GCGACATGTATATCTCCTTATTAAAGTTAAACAAAATTATTTCT	For cloning modifying enzyme into pCDFDuet
pCDF:ChsH/HymH plasmid F	GCATAATGCTTAAGTCGAACAGAAAGTA	For cloning modifying enzyme into pCDFDuet
pCDF:ChsH plasmid R	CGGCATGTATATCTCCTTATTAAAGTTAAACAAAATTATTTC	For cloning modifying enzyme into pCDFDuet
pCDF:HymH plasmid R	GTTCTCATGTATATCTCCTTATTAAAGTTAAACAAAATTATTTC	For cloning modifying enzyme into pCDFDuet
pCDF:MelH+Hc plasmid F	AATGACTAGGCTGCTGCCACCG	For cloning modifying enzyme into pCDFDuet
pCDF:MelH+Hc plasmid R	GGGCATGTATATCTCCTTATTAAAGTTAAACAAAATTATTTC	For cloning modifying enzyme into pCDFDuet
pCDF:MelH+Hc N-term fix F	AGAAGTCGAAACCCAAACTAGGACTTTCTCTCATGCCCACCG AGGAC	For cloning in missed N-terminal residues
pCDF:MelH+Hc N-term fix R	AGTTTGGGTTTCGACTTCTGCATGGTATATCTCCTTATTAAAG TTAAACAAAATT	For cloning in missed N-terminal residues
pCDF:MelH plasmid F	ACATATGCCCGAATGACTAGGCTGCTG	For deleting MelHc to only express MelH
pCDF:MelH plasmid R	CTAGTCATTCGGGCATATGTATATCTCCTTCTTATAC	For deleting MelHc to only express MelH
pCDF:His-MelH+Hc F	GGCAGCCATCATCATCATCATCACTCTCAGAAGTCGAAACCC AAACTAG	For adding His-tag to MelH
pCDF:His-MelH+Hc R	ATGATGATGATGGCTGCCCATGGTATATCTCCTTATTAAAGTT AAACAAAATTA	For adding His-tag to MelH
pRSF:ArlA F	TACATATGCCGACTCCCTGATAATTTACCTAGGC	For removing ArlH from plasmid
pRSF:ArlA R	AGGGAGTCGGCATATGTATATCTCCTTCTTATAC	For removing ArlH from plasmid
pACYC:DybA A46P F	CTTGCCCGCCATGCGGAATGGGTTGAAAG	Site-directed mutation to probe DybH substrate scope
pACYC:DybA A46P R	CATTCCGCATGGCGGGCAAGGATCCAGG	Site-directed mutation to probe DybH substrate scope

pACYC:DybA 47Ains F	CCCGGCCGCTTGCGGAATGGGTTGAAAG	Site-directed mutation to probe DybH substrate scope
pACYC:DybA 47Ains R	CCATTCCGCAAGCGGCCGGGCAAGGATCC	Site-directed mutation to probe DybH substrate scope
pET28:GFP-CPACGMG F	CCGGCCTGCGGAATGGGTTAAGATCCGGCTGCTAACAAAGCCC	Adding CPACGMG with a 4-aa linker to the end of GFP
pET28:GFP-CPACGMG R	ACCCATTCCGCAGGCCGGGCAGCCAGCGGATCCCTTGTAC	Adding CPACGMG with a 4-aa linker to the end of GFP
pET28:GFP-CPACGMG_long_linker F	TGCCCGGCCTGCGGAATGGGTTAAGATCCGGCTGCTAACAAAGC	Adding CPACGMG with a 10-aa linker to the end of GFP
pET28:GFP-CPACGMG_long_linker R	CCATTCCGCAGGCCGGGCAGCCAGAACCAGCAGCGGA	Adding CPACGMG with a 10-aa linker to the end of GFP
pRSF:DybAH no His F	ATAAGGAGATATACCATGAAAATCTCAAAACCAGTACTTCAG	To remove N-terminal His tag from DybA
pRSF:DybAH no His R	GAGATTTTCATGGTATATCTCCTTATTAAAGTTAAACAAAATTATTTCTAC	To remove N-terminal His tag from DybA
pRSF:DybAH His embed F	ATCACCATCATCACCACGATATTAAACCTGAAAAGGATAAAAAAGCGAAGACGC	Inserting His tag to position 27 (following signal peptide)
pRSF:DybAH His embed R	GTGGTGATGATGGTGATGCATGTCGGCGCAAGCGGTAATT	Inserting His tag to position 27 (following signal peptide)
pRSF:NedA C-His plasmid F	AACAGAAAGTAATCGTATTGTACACG	For cloning in NedA gblock into pRSFDuet
pRSF:NedA C-His plasmid R	ATTTCCTAATGCAGGAGTCGCATAAG	For cloning in NedA gblock into pRSFDuet
pCDF:NedBC plasmid F	CAATAACTAGCATAACCCCTTGGGG	For cloning NedBC gblock into pCDFDuet
pCDF:NedBC plasmid R	TCCTAATGCAGGAGTCGCATAAGGGAG	For cloning NedBC gblock into pCDFDuet
ChsA gblock (codon optimized)	TGCGACTCCTGCATTAGGAAATTAATACGACTCACTATAGGGGAATTGTGAGCGGATAACAATTCCCCTGTAGAAATAATTTTGTTTAACTTTAATAAGGAGATATACCATGCATCATCATCATCATCATAAACTGAGCAAAAGCCTGCTGAGCGCGATTATGATTGGTATTGCGGTTCAGACCACCGTGGTGAGCTGTAGCAAAGATGAACAGGTGAAACCGAAAAAAGCGGATCAGGCGAACAAACAGAGCGAAAGCAAACCGGTGGATAATCCGGATAGCTGCCCGGCGTGCGGCATGGGCTAAAAGCTTGCGGCCGCATAATGCTTAAGTCGAACAGAAAGTAATCGTATTGTACACG	Precursor peptide
ChsH gblock (codon optimized)	TTGTTTAACTTTAATAAGGAGATATACATGCCGAAAATTCTGAGCGCGGTGGCCTGCAATCTGGATGCAAACATTCTGGCGGCGTGCCTGCCGCTGTTTGAAGAAAGCCGCGTTGAAGCCATTGAATGGAGCTTTGATGCCCTGTATAAAGTGAAAGAAGTGCCGGGCTGGTTTCGCGAACTGCTGAGCGCCTTTAGCGATGAAAACCGCCTGATTGGCCATGGCGTGTTTTTTTCACTGTTTTCGGGCAAATGGCTGCCGGAACAGGAAGCGTGGCTGAGCCATCTGCAGCAGACCGCGACCGAATTTAGCTTCGATCACATCACCGAACATTTCGGCTTCATGACCGGCGAAGATTTCCACCATGGCGCCCCGCTGAATATCCCGTACAGCAGCAGCACCCTGAACATTGGCAAAGATCGTCTGAAACGCATTTATCATGCGTGCGGTCGCCCGGTTGGCCTGGAAAATCTGGCCTTCAGCTATAGCCTGGATGAAGTGAAACGCCATGGCACCTTTCTGGAACAGCTGCTGGAACCGGTGAACGGCTTCATTATTCTGGATCTGCATAACCTGTTTTGTCAGCTGAAAAATTTTGATCTGGATTTTAATACCCTGATTTCACTGTATCCGCTGGATAAAGTGCGTGAAATTCATATTAGCGGCGGTAGCTGGGATGATAGCGCGGCGGCGCCGGATCGTAGCATTCGCCGCGATACCCACGATGAAAGCGTTCCGCCGGAGGTTTTTCAGCTGCTGGAAATGACCATTCCACAGTGCCCGAACCTGAAATATGTGGTGCTGGAACAGCTGGGCAACGGCCTGCAGTCTACCGCGTCCAAACAGGGCTTCTATAACGATTTTCTGCAGATGCAGGAAATTGCCCAGCAGCACAATTATAAAGAAGGCAACCCGTTCCTGCCGCTGGAAAGCCTGCCGATTATTACCGGCGCGGCGATTGAAGATCTGCGTCTGTATCAGCAGCAGCTGGAGCTGTCAGCGATTCTGGAAAATGCGGGTACCTATGCGGAAGCCATGCAGCAGCTGCAGCAGTCGAGCCTGGCCCATAGCGATTGGAAAATTGAAGAATGGGAACCGTACATGATTGAAACCGCGGTGAAAATTGCGCGCAAATGGAAAAAAATAAAAGCTTGCGGCCGCATAATGCTTAAGTCGAACAGAAAGTA	Modifying enzyme
HymA gblock (codon optimized)	TGCGACTCCTGCATTAGGAAATTAATACGACTCACTATAGGGGAATTGTGAGCGGATAACAATTCCCCTGTAGAAATAATTTTGTTTAACTTTAATAAGGAGATATACCATGCATCATCATCATCATCA	Precursor peptide

	TAAGCTCAGCCAAGCCATATTAGGCGCAGTATTAGTGGGTCT<br>GACAGCTCAAACAACCGGTTGTATAAAGAAGTCTGATCCTACT<br>CCAAAGGAAGAACAAGGAAAGAGCGGTAAGAAGAGCCCGGA<br>GATTCCGTACAATTGTCCAGGATGCGGACTGGGGTAAAAGCT<br>TGCGGCCGCATAATGCTTAAGTCGAACAGAAAGTAATCGTATT<br>GTACACG	
HymH gblock (codon optimized)	TTGTTTAACTTTAATAAGGAGATATACATGAGAACTGTGGCTC<br>CACCGGATAACGCTAACGAACGTGCGATTTATTCATCAATCG<br>CGTGTAATCTTGATGCGAATATCCTCACCGCTGCGTTCCCTCT<br>GCTTGAACAAGGCAAAGTTGAAGCGTTAGAGTGGTCATTCGA<br>CGCACTTTTCTGGGCAGAACAGGTTCCAGACTGGTTCACAGA<br>GCTCTTACAAGCATATTCGGCCCAGCACCGCTTAGTAGGTCA<br>CGGTGTTTATTTTAGCTTATTAAGCGGCAGATGGACCGCGGA<br>ACAACAACAATGGCTCCAGCATCTTAAAGAATTGACACGACGT<br>TTCAGTTTTGATCATGTAACCGAACATTTCGGTTTCTTCACAG<br>GGCAAAACTTCCACGCAGGTGCCCCATTGCCGATTCCCTACG<br>GATCTAATACGCTCCGTATCGGCCAGGATCGATTACGACGCA<br>TGCAAGAAGCATGTGGTTGCCCAGTCGGGCTGGAGAATTTAG<br>CATTCGCATACAGCCTGGAAGAGGTTAAGCGCCATGGTGAAT<br>TCTTGGAGCAATTGGTTGAACCTGTCAATGGGTTCTTGATCTT<br>GGATTTACATAATGTCTTTTGTCAACTGCATAATTTTAGCGTAC<br>CTTATGAGGAATTGATGGCTTTGTACCCATTAGACCGTGTCCG<br>TGAGATACACATCAGCGGCGGATCATGGGAAGCTAGTGGCC<br>AAGTTCCCGGGCTGCAAATACGACGTGACACGCACGACGAA<br>GCAGTTCCTGAGGAAGTTTTCCAACTTTTGTATAATACGATGC<br>CTCGTTGCCCGAATCTTAAGTACGTCGTATTAGAACAACTCGG<br>CACCGGTCTGCAAACAGAAGATTCAAGAGCGCATTTTCAATC<br>AGATTTCCACCGAATGCAAGCTTTAGTAAATCAGCATCGTAGT<br>ACAACATCCATACGAGCGGTGCAACCCTTCTTGCCGATTCAC<br>AGCGCCGTAGCTGGGCCTGTGGCAGAGGACGCACAACTTCA<br>TGAACAACAACAACAACTGTCTCATATTCTTGAGACCGCACCT<br>TCTTATGAGGATGCACAACGCCAACTGCAAGCCTCGGCTCTC<br>GCGTATACAGCCTGGAAGGTAGAAGAATGGGAGCCGCACAT<br>GCTTGAGACTGCTATTTCCATCGCACAAAAGTGGAAACCACA<br>ACCACGTTGAAAGCTTGCGGCCGCATAATGCTTAAGTCGAAC<br>AGAAAGTA	Modifying enzyme
MelA1 gblock (not codon optimized)	ATAAGGAGATATACCATGGGCAGCAGCCATCACCATCATCAC<br>CACAGCCAGATGAAAAACAAATCAGCCAAAGTTCTAACTTCGC<br>TATTCGCGCTCGGTCTGACCACTGGCGCCGTGCCCGCGCGC<br>GCAGATAAAGCAGCGGTGGAAACAGCCGAAGCAAGCGTAAA<br>AATGTTCGAGCAACGCTCAGGCACGAACAATTCGTCTTATAC<br>CAGCCTACTGATGTACCTGGCTGGGGTATATCTGGCGAACGA<br>CAGAGAGAAAGACGCTGAGATCATATACAACAAAGCAATCTT<br>CAATCTCAAATCGAAACCGGATAGGAAAGCTGAAGTGCCCGC<br>CTTGATGCTGAACTGGGCAATGCTGCTGGCATCGAACAAAGG<br>TGCATCTAAAGAAAAAGCGGAAAAAGCATTGTCGGATGGACT<br>GAAACTCGCAAACGACTTGCCGGCCGCCTCCAAAGAGCGGA<br>TCAATTATCTGATAGGAACAATCAATTTCTACAACGTAATTGG<br>CAAACCTGCCGAGAAGCAAGCCCGCATCAAAGCAGCCGATG<br>AGCATCTTGCCACGCTGGAGAAAAACGAAAAACTAAACAATG<br>AAGAAATCACCAATGTCGCAGCGAATCTAGTGAAACTGGCAG<br>AGATTCAAACGTTTCCCATGCCGGTCATGCGTTTGCGCTACC<br>AGCCTCCGGTGTTTCAAGTAGTGCCGGACAACTCGCCGGACA<br>AACCGAACACAGTGCGAGCAAAGGAGTTCAAGTCGGCGGAA<br>GCTTTCCAATTGCGCGCGATAAAACAATACGATCGTTTGCCG<br>GAAACGGTGCCCTGGCGCATCGAAGCGCACAGAAAGTTGATT<br>CTCTGGTATCGCAGCCTGGGTCAGACGAAACAGGAAGAGTTT<br>CAAATTCAGCAGTTAGGCAAAATCATGCACACTACAGATCGC<br>GACAAACTGTTTCCCCAGCCGGCACCCTGCCCGGCTTGCGG<br>CATGGGTTAGAGCTTGCGGCCGCATAATGCTTA	Precursor protein
MelH + MelHc gblock (codon optimized)	TTGTTTAACTTTAATAAGGAGATATACATGCCCACCGAGGACT<br>TCCAAATGGCAACGGAACAGTTATTCGAGGAAGAGCGCGTGG<br>AGAGCTTGGAGTGGTCGTTCGACTTTACGTGGAATGGAGTCG<br>TTGTGGACCCCTGGGCATTAGAGCTGATCGATAAATACAGCG<br>AGGCTAACGCTCTTACGGGACACGGTGTTAATCTGTCCCCGC<br>TGTCTGCCCGTTTCTCTAAGAGACAAGAGGAGTGGCTCGCTC<br>GTGCTCGCGAGGAATTCAAGACCCGCAAATACGTGCACGCTT<br>CTGAACACTTTGGGTTCTCCGAGGCTGGTCCTATCAAGCACG<br>GAGCTCCCCTGTCTGTTCCGATGGACGCGGCCAGCCTTAAG<br>GTTGGTAAGGAAATGTTAAAGAGATATGCCGACGCTACCGGG	Modifying enzymes

	TGTCCCGTTGGACTTGAAAATCTGGCGTTCGCTTTCTCTATAA<br>ACGACGTTAAGAAACAAGGTCACTTCATTGATGAGCTGATCA<br>GTTCTGTAAACGGATTCCTGTTATTGGACTTACATAACATTTTC<br>TGCCAAATTGCCAATTTCGGAATAAGTGAGCTGGAGCTTCTTA<br>ATAGCTATCCGTTAGATAAGGTCCGGGAGATACATATATCTGG<br>TGGTTCATGGTCACCCAGTATTTCTGGTAAACGTGCGGCCGT<br>GAGAAGAGATACCCACGATGACGGGGTGCCTCAGGAAGTGT<br>TTAACCTGGCAGCGTTGGCGTTGAAACTGTGTCCGAACATCG<br>AGTTTGTGATATTCGAGCGACTTGGAAACACTATGTTTTCTTT<br>AGAGGCACAGTCCGAATTCCGGGATGACTTTGAATCAATGGA<br>ACAAATTCTTGAATTCTGTTACGCGTAAAAGCTTGCGGCCGCA<br>TAATGCTTAAGTCGAACAGAAAGTAATCGTATTGTACACGGCC<br>GCATAATCGAAATTAATACGACTCACTATAGGGGAATTGTGAG<br>CGGATAACAATTCCCCATCTTAGTATATTAGTTAAGTATAAGAA<br>GGAGATATACATATGCCCGACACTCTGAAGCTCCAGGAATTT<br>CAGGATGCATTGAACGAACTCCTCGCTTCGGGTAAGAGCCAC<br>GCGGAGATTATGGAAGAGTTAAATAGTAACCCGCAGTTCGAG<br>TCATACCGGGAGTATATATCGGAGTTTGACCCCGACATGGTG<br>GAAGTTGCCCGAGAACTCATGGGTAAATGGGCCCAACGGGT<br>AGGTGAATGACTAGGCTGCTGCCACCG	
ArlAH gblock (not codon optimized)	AATTTTGTTTAACTTTAATAAGGAGATATACCATGGGCAGCAG<br>CCATCACCATCATCACCACAACCAGAATCTCCGTGAGAAGGC<br>CACCCAGCTCCTCGATGTGGCGAGCAACACCCAGGGAATGA<br>AGTCGAGCAGTCCTCTTCCCTGTCCGGCCTGCGGCATGATGA<br>TGGCGCTGCCCGAGTCCAGCCCCCAACTCGTGACCCCGGCC<br>ACCCGCTGAAAGCTTGCGGCCGCATAATGCTTAAGTCGAACA<br>GAAAGTAATCGTATTGTTCACGGCCGCATAATCGAAATTAATA<br>CGACTCACTATAGGGGAATTGTGAGCGGATAACAATTCCCCA<br>TCTTAGTATATTAGTTAAGTATAAGAAGGAGATATACATATGCC<br>GCCTCGCGTGGGTCTGAATCTTCTGACCGACGACGCCTTCCG<br>CGAGGCGGTACGGCCGCTCTTCGCCGAGGGGCTGGTCGCC<br>GCGCTCGAGTGGGACATCGACGATTCCTGGGGATTCTCCAC<br>CCGTGCACTCCCCGGCTGGACCGAGCGTATGCTCGACCTCT<br>ATGCGGAGGAGGGAGCCTTGTACGGCCACGGCGTGTGGCTG<br>TCGGTGCTGACCGCCGCCTGGCAGCCTCGGCAGGAGGCGT<br>GGATCGAGCGTCTCGCCCAGGAGTGCCGCCGCCGCCGCTAC<br>CGCCACGTCTCCGAGCACTTCGGCTTCACCGCCGCGGGGCC<br>GTTCACCCGCAGCACCATGCTGCCCCTGCCGTATTGCACCGC<br>CGCCGTGGACATCGGCCGCGACCGGCTCGAACGGCTGCGC<br>GCCGCCACCGGCGGCCCCGTCGGCCTCGAGGTGCTGGCCA<br>ACACCCTCGCCCCCGTCGATGCGCTCCACCAAGGTCCGTTC<br>CTCGATGCCGTGCTGACCCCCACCGAGGGCTTCCTGGTGCT<br>CGACGTCCACAACGTGTGGACCCAGGCGGTCAACACCGGCC<br>TGCCTCCAGAGCTCCTGCTCGAGACCTATCCGCTGGAGTGC<br>GTCCGGGAAATCCACCTCTCGGGTGGGAGCTGGGGCCGCGC<br>GCTCGGCGCCGGGGACTCCCGTCCCGTCCGGCTCGACAGTC<br>ACGACGGACCCCTCCTGTCACCCGTGCTGTCCCTGCTCCGC<br>CGGGCGCTCGCCCTCTGCCCTCATTGCGAGGTCGTCATCGT<br>CGAGCGGCGAAGCGAGACCCTCGAGTCAGAGGAAGTGCGC<br>GCCGGGTGGCGCGCCCAGTACCGCGCCGTGGTCCAGCTGG<br>TGGCCGAGGCCAGCGCAACCCCCGTCAGCCCGGTCCGTGTC<br>TCCGCCTCCGCGCCGCCCCCCGAGCTGCTCGACACGGAGGA<br>GCTCGCCCGGTACCAGCGCGAGCTGGTCACGGCCCTCGTCG<br>AGGAGTCCGAGAGCCGGGTTATCCTGCAGCGTCTGCGCCAG<br>GGTATCGCCGGCGCACGGCTCGCGCCCTACCTCGAGACCTT<br>CGATCCGCGCATGGTCGAGCTGCTGGCCATCCTCGCGTCAC<br>GCTGGTCCGCCTTCGACCCCGCTCGAGCCACTCCCTGATAAT<br>TTACCTAGGCTGCTGCCACCGCTGAGCAATAACTAGCATAAC<br>CCCTTGGGG	Precursor peptide and modifying enzyme
NedA gblock (codon optimized)	TGCGACTCCTGCATTAGGAAATTAATACGACTCACTATAGGG<br>GAATTGTGAGCGGATAACAATTCCCCTGTAGAAATAATTTTGT<br>TTAACTTTAATAAGGAGATATACCATGAAAACCACGTCGACCC<br>TGATGGCACTGGCAGGGGCGCTGGTGCTGGCGGGCTGCCAA<br>GTCGAACCAGCGCAGTCGTCCAGTCCTGAGGTTAGCGTGCA<br>GCAGAAAGCAGGCGAAGGCAAATGCGGCGCAGCCTCGCAGG<br>CGGGTAAGGCGGCGGAGGGAAAATGTGGTGAAGGTAAATGC<br>GGCTCGAAGCATCACCATCATCACCACTGAAAGCTTGCGGCC<br>GCATAATGCTTAAGTCGAACAGAAAGTAATCGTATTGTACACG	Precursor peptide
NedBC gblock (codon optimized)	CTCCCTTATGCGACTCCTGCATTAGGAAATTAATACGACTCAC<br>TATAGGGGAATTGTGAGCGGATAACAATTCCCCTGTAGAAATA	Modifying enzymes

	ATTTTGTTTAACTTTAATAAGGAGATATACCATGAACACTCTGC<br>ACGGCGCTGGGCTGGGTTATAAACGTTCTATGGCAGCAGATT<br>TCCTGCAGCTGGATCGCAACAATTCTCCGATTCGCTTTATCGA<br>GATTGCCCCGGAAAATTGGTTGCGTATGGGCGGCGCGGCCC<br>GCAAGCAATTCGACGAAGTGGCCGAGCGTTTTCCGGTGGCTT<br>GCCACGGTCTGAGCCTGTCCCTGGGAGGCCAGGACCCGTTA<br>CAACTGGATTTCCTGAAACAGATCAAAGCGTTTTTGCGCCAGT<br>ACCGTATCGGTTTCTTTTCAGAACATCTGTCCTACTGTTCCCA<br>CCACGGACACATCTACGATCTGCTGCCGCTTCCGTTCACGGA<br>AGAGTCGGTACGTCACACAGCTGCCCGCATTCGCGCGGTGC<br>AGGACATTTTGGAGATGCGTATCGCCGTGGAAAATACATCTTA<br>CTATGCACACAATCCCATCGCGGAGATGGATGAAGCGGAGTT<br>CCTGAACGCCGTGGTGCGTGAGGCGGATTGCGACATCCACT<br>TAGATATTAATAATATTTACGTGAATGCCGTCAACCACGGCAT<br>CGTGGCCCCACGCGACTACATTGACCGCACGGATCTGGCCC<br>GTGTGAGTTATATGCACATGGCAGGCCATGACGAGAAGACTG<br>AGAATTTGTTAATCGACACCCACGGCCAACCGGTTTGTGATG<br>ACGTGTGGGACTTATTTGCCTACGCGTGTCGCCGCCTGCCTC<br>ACTCTGTGCCAACCTTACTGGAACGCGATAGCAATTTTCCGC<br>CTTTTGCCGAATTAGAAGCCGAGGTAGCTCGCATCGCCGCTA<br>TCCAACAGCAGGCCGAGAAAGAACGTCATGCGGCGGCGTAA<br>AAGCTTGCGGCCGCATAATGCTTAAGTCGAACAGAAAGTAAT<br>CGTATTGTACACGGCCGCATAATCGAAATTAATACGACTCACT<br>ATAGGGGAATTGTGAGCGGATAACAATTCCCCATCTTAGTATA<br>TTAGTTAAGTATAAGAAGGAGATATACATATGCAACCGCATAA<br>CCCAAGTCCGTCGGCGCGTGCGCAAGCCGAGCTGGCAGATC<br>ATGTTCGCAACCCTGCACTGCCCGCCCCGGCGGGGATTGCA<br>CCGGAACGCCTGGCGGTATATACCCGCCTGGTGCGTAACAAT<br>CTTAAATCGTTCCTGGATCTGTGCTTCTCTGATTCCAGCCTGA<br>TGCTGGATCCCGCGCAATGGCAGGGCTGGCAGAATCGCTTT<br>CTGATCGAGGCACGCCCCGAAAGCCCGTTTTTTAATGATATT<br>CCGGCGCAGTTTCTTGCCTATTTAAACCGTTTGCCTGAACATG<br>ATCGTCCGTCAGAGAACATTCTTGCGATGATGGATTTCGAAAC<br>CGCTCTGTTGCATGCGGAGACGGCGCGCCAGCCTGATTCTG<br>ATGGCCGTTGGAGCGAGCATAGCGTCCTGAGCTGGGCCCCG<br>GCCGCACGTCTGCAGCAATATCCGTGTGATTTCGTGAGCTCC<br>GGCCTGGCACAGATCAACGCGGGCGCGTGTCACGTTCTGTC<br>CTGGCGTAACCGTCGCAACGAAGTCTATTATCGTATCGTGGA<br>AGATACCGATCTGTTTCTGCTGCAGCACTTTCAGTCACAGAAC<br>GACACTTTTGCGCGCCTGCTGGAAAGCCTGCAGTCCTTATTA<br>CCGGGTCAAGATATTGAAGGTCGTCTTAAAACGGCCGTCGGT<br>GGGTGGGTTGAAGCCGGCGTGCTGCTGACCGCGGAGTAACT<br>AGGCTGCTGCCACCGCTGAGCAATAACTAGCATAACCCCTTG<br>GGG	

Supplementary Table 10. Strains and plasmids used in this study.

Strain/plasmid name	Purpose
5alpha (NEB)	E. coli strain for cloning plasmids
BL21 (DE3)	E. coli strain for protein expression
pACYCDuet-1:His-ChrA	For expression of His-ChrA
pACYCDuet-1:His-DybA	For expression of His-DybA
pACYCDuet-1:His-ChsA	For expression of His-ChsA
pACYCDuet-1:His-HymA	For expression of His-HymA
pRSFDuet-1:His-ArlA	For expression of His-ArlA
pACYCDuet-1:His-MelA1	For expression of His-MelA1
pET28a:eGFP-CPACGMG-4aa-linker	For expression of His-GFP-CPACGMG-4aa-linker
pET28a:eGFP-CPACGMG-10aa-linker	For expression of His-GFP-CPACGMG-10aa-linker
pCDFDuet-1:ChrH	For expression of untagged ChrH
pCDFDuet-1:DybH	For expression of untagged DybH
pCDFDuet-1:ChsH	For expression of untagged ChsH
pCDFDuet-1:HymH	For expression of untagged HymH
pRSFDuet-1:MCS1_His-ArlA_MCS2_ArlH	For coexpression of His-ArlA with untagged ArlH
pCDFDuet-1:MelH	For expression of untagged MelH
pCDFDuet-1:MCS1_MelH_MCS2_MelHc	For coexpression of untagged MelH and MelHc
pCDFDuet-1:MCS1_His-MelH_MCS2_MelHc	For coexpression of His-MelH and untagged MelHc
pET28a:His-TEV-DybH	For expression of His-DybH
pRSFDuet-1:MCS1_His-DybA_MCS2_DybH	For coexpression of His-DybA and untagged DybH
pACYCDuet-1:His-DybA-A46P	For expression of His-DybA with mutated 'CPPCGMG' sequence
pACYCDuet-1:His-DybA-A47ins	For expression of His-DybA with mutated 'CPAACGMG' sequence
pRSFDuet-1:MCS1_DybA-His-embed_MCS2_DybH	For coexpression of DybA (His-tag embedded at residue 21) with untagged DybH
pRSFDuet-1:NedA-C-His	For expression of C-terminally His-tagged NedA
pCDFDuet-1: MCS1_NedB_MCS2_NedC	For expression of untagged NedB and NedC

References

1. Kenney, G. E.; Dassama, L. M. K.; Pandelia, M. E.; Gizzi, A. S.; Martinie, R. J.; Gao, P.; DeHart, C. J.; Schachner, L. F.; Skinner, O. S.; Ro, S. Y.; Zhu, X.; Sadek, M.; Thomas, P. M.; Almo, S. C.; Bollinger, J. M., Jr.; Krebs, C.; Kelleher, N. L.; Rosenzweig, A. C., The biosynthesis of methanobactin. *Science* **2018**, *359* (6382), 1411-1416.
2. Ting, C. P.; Funk, M. A.; Halaby, S. L.; Zhang, Z.; Gonen, T.; van der Donk, W. A., Use of a scaffold peptide in the biosynthesis of amino acid-derived natural products. *Science* **2019**, *365* (6450), 280-284.
3. Ayikpoe, R. S.; Zhu, L.; Chen, J. Y.; Ting, C. P.; van der Donk, W. A., Macrocyclization and backbone rearrangement during RiPP biosynthesis by a SAM-dependent domain-of-unknown-function 692. *ACS Cent. Sci.* **2023**, *9* (5), 1008-1018.
4. Nguyen, D. T.; Zhu, L.; Gray, D. L.; Woods, T. J.; Padhi, C.; Flatt, K. M.; Mitchell, D. A.; van der Donk, W. A., Biosynthesis of macrocyclic peptides with C-terminal β-amino-α-keto acid groups by three different metalloenzymes. *ACS Cent. Sci.* **2024**, *10* (5), 1022-1032.
5. Chioti, V. T.; Clark, K. A.; Ganley, J. G.; Han, E. J.; Seyedsayamdost, M. R., N-Cα bond cleavage catalyzed by a multinuclear iron oxygenase from a divergent methanobactin-like RiPP gene cluster. *J. Am. Chem. Soc.* **2024**, *146* (11), 7313-7323.
6. Manley, O. M.; Shriver, T. J.; Xu, T.; Melendrez, I. A.; Palacios, P.; Robson, S. A.; Guo, Y.; Kelleher, N. L.; Ziarek, J. J.; Rosenzweig, A. C., A multi-iron enzyme installs copper-binding oxazolone/thioamide pairs on a nontypeable *Haemophilus influenzae* virulence factor. *Proc. Natl. Acad. Sci. U. S. A.* **2024**, *121* (28), e2408092121.
7. Leprevost, L.; Jünger, S.; Lippens, G.; Guillaume, C.; Sicoli, G.; Oliveira, L.; Falcone, E.; de Santis, E.; Rivera-Millot, A.; Billon, G.; Stellato, F.; Henry, C.; Antoine, R.; Zirah, S.; Dubiley, S.; Li, Y.; Jacob-Dubuisson, F., A widespread family of ribosomal peptide metallophores involved in bacterial adaptation to metal stress. *Proc. Natl. Acad. Sci. U S A* **2024**, *121* (49), e2408304121.
8. Padhi, C.; Zhu, L.; Chen, J. Y.; Huang, C.; Moreira, R.; Challis, G. L.; Cryle, M. J.; van der Donk, W. A., Biosynthesis of biphenomycin-like macrocyclic peptides by formation and cross-linking of ortho-tyrosines. *J. Am. Chem. Soc.* **2025**, *147* (27), 23781-23796.
9. Teufel, F.; Almagro Armenteros, J. J.; Johansen, A. R.; Gíslason, M. H.; Pihl, S. I.; Tsirigos, K. D.; Winther, O.; Brunak, S.; von Heijne, G.; Nielsen, H., SignalP 6.0 predicts all five types of signal peptides using protein language models. *Nat. Biotechnol.* **2022**, *40* (7), 1023-1025.
10. Zhang, J.; Zhang, D.; Xu, Y.; Zhang, J.; Liu, R.; Gao, Y.; Shi, Y.; Cai, P.; Zhong, Z.; He, B.; Li, X.; Zhou, H.; Chen, M.; Li, Y.-X., Large-scale biosynthetic analysis of human microbiomes reveals diverse protective ribosomal peptides. *Nature Commun.* **2025**, *16* (1), 3054.
11. Oberg, N.; Zallot, R.; Gerlt, J. A., EFI-EST, EFI-GNT, and EFI-CGFP: Enzyme Function Initiative (EFI) web resource for genomic enzymology tools. *J. Mol. Biol.* **2023**, *435* (14), 168018.
